# The impact of lipidome on breast cancer: a Mendelian randomization study

**DOI:** 10.1186/s12944-024-02103-2

**Published:** 2024-04-15

**Authors:** Yuchen Cao, Meichen Ai, Chunjun Liu

**Affiliations:** 1 Plastic Surgery Hospital, Chinese Academy of Medical Sciences and Peking Union Medical College, No. 33 Badachu Road, Shijingshan, Beijing, 100144 China; 2 Southern Medical University, Guangzhou, 510515 China

**Keywords:** Lipidome, Breast cancer, Mendelian randomization, Bayesian model averaging mendelian randomization, Estrogen receptor

## Abstract

**Objective:**

This study aims to investigate the association between specific lipidomes and the risk of breast cancer (BC) using the Two-Sample Mendelian Randomization (TSMR) approach and Bayesian Model Averaging Mendelian Randomization (BMA-MR) method.

**Method:**

The study analyzed data from large-scale GWAS datasets of 179 lipidomes to assess the relationship between lipidomes and BC risk across different molecular subtypes. TSMR was employed to explore causal relationships, while the BMA-MR method was carried out to validate the results. The study assessed heterogeneity and horizontal pleiotropy through Cochran's Q, MR-Egger intercept tests, and MR-PRESSO. Moreover, a leave-one-out sensitivity analysis was performed to evaluate the impact of individual single nucleotide polymorphisms on the MR study.

**Results:**

By examining 179 lipidome traits as exposures and BC as the outcome, the study revealed significant causal effects of glycerophospholipids, sphingolipids, and glycerolipids on BC risk. Specifically, for estrogen receptor-positive BC (ER^+^ BC), phosphatidylcholine (*P* < 0.05) and phosphatidylinositol (OR: 0.916–0.966, *P* < 0.05) within glycerophospholipids play significant roles, along with the importance of glycerolipids (diacylglycerol (OR = 0.923, *P* < 0.001) and triacylglycerol, OR: 0.894–0.960, *P* < 0.05)). However, the study did not observe a noteworthy impact of sphingolipids on ER^+^BC. In the case of estrogen receptor-negative BC (ER^−^ BC), not only glycerophospholipids, sphingolipids (OR = 1.085, *P* = 0.008), and glycerolipids (OR = 0.909, *P* = 0.002) exerted an influence, but the protective effect of sterols (OR: 1.034–1.056, *P* < 0.05) was also discovered. The prominence of glycerolipids was minimal in ER-BC. Phosphatidylethanolamine (OR: 1.091–1.119, *P* < 0.05) was an important causal effect in ER^−^BC.

**Conclusions:**

The findings reveal that phosphatidylinositol and triglycerides levels decreased the risk of BC, indicating a potential protective role of these lipid molecules. Moreover, the study elucidates BC's intricate lipid metabolic pathways, highlighting diverse lipidome structural variations that may have varying effects in different molecular subtypes.

**Supplementary Information:**

The online version contains supplementary material available at 10.1186/s12944-024-02103-2.

## Introduction

Despite significant advancements in cancer research, breast cancer (BC) remains the most common malignancy among women worldwide, representing a leading cause of cancer-related deaths and ranking as one of the top three most prevalent cancers globally [1, 2]. The incidence of this aggressive disease affects approximately one in eight women in the United States, while in Asia, the alarming rate stands at one in every 35 women diagnosed with BC. The accumulated pieces of evidence underscore the ongoing need for intensified efforts in prevention, providing high-quality screening, diagnosis, and treatment to the population [3, 4]. Current clinical management of BC offers diverse options, including surgery, chemotherapy, radiotherapy, and targeted therapies [5]. Disparities in BC survival rates are substantial worldwide, with an estimated 5-year survival rate of 80% in developed countries contrasted with less than 40% in developing nations [6, 7]. The American Cancer Society projects a staggering 297,790 deaths due to BC in women by the year 2023 [8], highlighting the urgent imperative to develop more effective and safe treatment modalities tailored for different regions of varying developmental stages.

Lipidomes are spherical vesicles composed of one or more concentric phospholipid bilayers surrounding an aqueous core [9]. Classified within the realm of nanomedicine, lipidomes represent a prominent category of lipid-based nanocarriers, standing out as one of the most sophisticated systems in this domain [10]. This non-toxic and biodegradable nature makes lipidomes a versatile drug delivery platform for a plethora of therapeutic agents. By stabilizing compounds, surmounting barriers to cellular and tissue uptake, and enhancing the pharmacokinetics and biodistribution of drugs at targeted sites within the body, lipidomes enhance the therapeutic efficacy while minimizing systemic toxicity [11]. Their pivotal role spans across various health fields [12, 13], playing a critical role in drug delivery systems for small molecules, peptides, genes, and monoclonal antibodies, as extensively documented and recognized in plenty of research [14–16]. Lipidomes, benefiting from their self-assembly process with inherent thermodynamic advantages, have emerged as a versatile tool in cancer therapy. They have been harnessed to enhance tumor targeting efficacy, minimize off-target toxicity [17], and treat patients with severe infections or compromised immune function [10], including those afflicted with BC [18]. Amid the recognized physical and psychological toll of conventional treatments like surgery and chemotherapy on patients, there is a pressing need to explore safer and more effective therapeutic modalities. Leveraging the distinctive characteristics of lipidomes, there is a growing interest in unraveling their role in BC treatment, particularly in exploring lipidome-associated viable gene therapy strategies for BC [19]. This burgeoning attention underscores the potential for lipidomes to revolutionize BC management and potentially offer personalized treatment options with enhanced outcomes and diminished adverse effects, aligning with the evolving landscape of precision medicine and targeted therapies across oncology.

Mendelian randomization (MR) has emerged as a prevalent method in genetic epidemiological research, probing causal inferences between exposures and outcomes. While randomized controlled trials (RCTs) stand as the gold standard in clinical evidence, MR methodology serves as a vital alternative strategy when RCTs are unfeasible, furnishing robust evidence concerning causal relationships between exposures and disease risks [20–22]. Utilizing single nucleotide polymorphisms (SNPs) as instrumental variables (IVs), MR assesses the causal impact of exposures on outcomes [23]. Considering that genetic variants are randomly allocated during gametogenesis and genotypes are typically unaffected by external environments, a rigorously designed MR study can largely mitigate confounding and reverse causality concerns.

Given the limited understanding of the causal impact of liposomes on BC, this study aims to establish a causal relationship between lipidomes and BC by providing compelling evidence. Ultimately, this research endeavours to advance the early detection and treatment of BC. As a result, this study aims to employ Two-Sample Mendelian randomization (TSMR) to investigate the underlying relationship between lipidomes and BC, and bayesian model averaging multivariate mendelian randomization (BMA-MR) was applied to further verify the results.

## Method

### GWAS data for lipidome

Genome-Wide Association Study (GWAS) summary statistics for lipidome were acquired from the GWAS Catalog (https://www.ebi.ac.uk/gwas/, GCST90277238-GCST90277416). A total of 7174 unrelated Finnish individuals from the GeneRISK cohort were included, and SNPs for 179 lipid species (sTable 1) belonging to 13 lipid classes and 4 categories were tested [24].

### GWAS data for breast cancer

The SNPs linked to BC were collected from the Breast Cancer Association Consortium (BCAC) GWAS database, accessible at https://gwas.mrcieu.ac.uk/. The dataset comprised 228,951 samples (*N* = 122,977 BC cases and 105,974 controls) and 10,680,257 SNPs. Meanwhile, a total of 175,475 samples (*N* = 69,501 Estrogen receptor-positive BC (ER^+^ BC) cases; 105,974 controls) and 10,680,257 SNPs related to ER^+^BC were investigated. Additionally, 127,442 samples (*N* = 21,468.

Estrogen receptor-negative BC (ER^−^ BC) cases; 105,974 controls) and 10,680,257 SNPs associated with ER^−^BC were examined [25].

### Instrumental variable extraction

The selection of SNPs as IVs for assessing the impact of lipidome on BC requires meeting three key assumptions of MR. Firstly, there should be a significant association between genetic variations and lipidomes. Secondly, confounding factors should not influence the chosen IVs, which are known to affect the relationship between lipidomes and BC. Lastly, the IVs should only influence the incidence of BC through lipidomes. Certain criteria were employed to ensure the selection of appropriate SNPs. Firstly, SNPs were required to exhibit a strong correlation with lipidomes, with a significance level of *P* < 5 × 10^–6^. Furthermore, SNPs were chosen in a manner that ensured their independence from one another, thereby minimizing potential confounding effects resulting from linkage disequilibrium (LD). In cases where the LD r^2^ value was equal to or exceeded 0.001, one of the SNPs was excluded from further analysis. Additionally, a genetic distance of 10,000 kb, representative of the region's length, was set. SNPs with an r^2^ value greater than 0.001 and located within this 10,000 kb region were eliminated to remove any remaining LD. These stringent SNP selection criteria were implemented to satisfy the assumptions of MR and ensure robustness and validity in evaluating the correlated impact of lipidome on BC. Additionally, SNPs with palindromic intermediate allele frequencies were also excluded. To identify suitable IVs for this study, the study utilized the PhenoScanner website (http://www.phenoscanner.medschl.cam.ac.uk/). Any SNPs of exposure found to be directly associated with the outcomes (BC, ER^+^BC, and ER^−^BC) were also excluded ahead of the MR analysis, respectively. Furthermore, the study comprehensively assessed the selected SNPs to evaluate their potential pleiotropic effects, utilizing the MR Pleiotropy RESidual Sum and Outlier (MR-PRESSO) test [26]. Fortunately, none of the SNPs exhibited any indications for removal after undergoing this evaluation. Next, the study employed a two-sample MR analysis to investigate the relationship between the exposure and outcome variables initially.

### Two samples of MR analysis

The inverse variance weighting (IVW) technique is commonly employed to combine multiple random variables in order to effectively reduce the overall variance. This approach assigns weights to each random variable in the aggregate based on their level of divergence. With IVW, it becomes possible to integrate the findings from distinct investigations. The IVW method was employed as the principal analytical approach, yielding the most accurate and reliable estimations when all genetic variants are considered legitimate instruments [27]. *P* values acquired from IVW method were subjected to False Discovery Rate (FDR) adjustment to account for multiple testing and enhance the reliability of our findings. To ensure the accuracy of our results, the study also employed various methods, including MR-Egger, weighted median, simple mode, and weighted mode. These additional analyses were conducted as safeguards in order to provide robust and reliable outcomes. For instance, if multiple methods indicate the same causal direction, confidence in that causal relationship would be strengthened. Further, The study employed the MR-BMA to further verify the causal effects of the exposure on the outcome by reducing potential biases, which enables the modelling of multiple correlated risk factors together and identifies the true causal risk factors, particularly suitable for high-throughput and highly correlated data [28].

### Sensitivity analysis

Sensitivity analysis was conducted to ensure the robustness of our findings. First, the study examined the intercept term of the MR-Egger regression model to assess the potential presence of pleiotropic effects [29]. If the P-value of the intercept term exceeded 0.05, it suggested that any influence of genetic pleiotropy was minimal. In such cases, the study concluded that the IVs solely impacted the risk of BC through lipidomes. To investigate the heterogeneity of the IVs and its potential impact on the causal estimate, the study employed the Cochran's Q test [30]. Funnel plots were also utilized to visually represent any heterogeneity within the obtained data. Furthermore, The study conducted a leave-one-out analysis to assess the sensitivity of the results. This involved performing MR analysis repeatedly, gradually eliminating one SNP at a time. Subsequently, the MR-PRESSO test was used to evaluate the presence of any disparities between the outcomes of MR analysis before to and after correction. Conducting both MR-Egger intercept test and MR-PRESSO allows the study to conduct sensitivity analyses from different perspectives. If the MR-Egger intercept test indicates the presence of directional pleiotropy, MR-PRESSO can further identify and correct for outliers that may be contributing to this bias. 

## Results

### Causal effects of lipidomes and BC

**Fig. 1:**
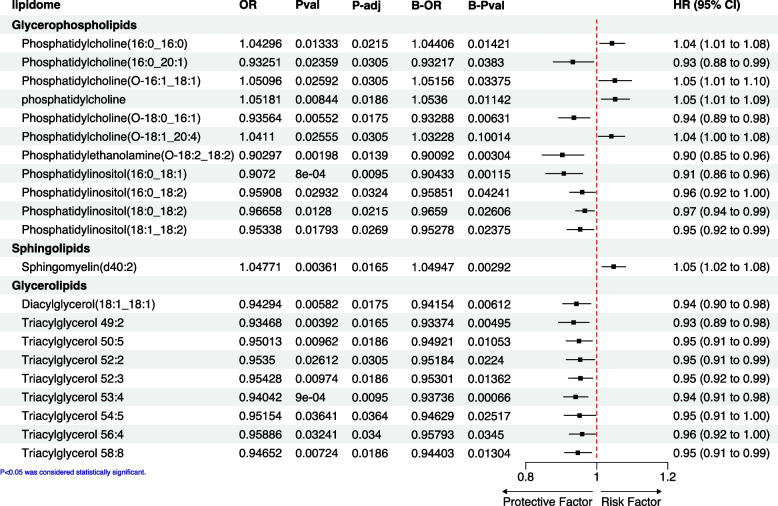
Forest plot of Mendelian randomization and Bayesian Model Averaging Mendelian Randomization method of the effect of lipidomes on BC. The OR, P-val, and 95% CI are results obtained from the IVW method, while P-adj represents the outcomes adjusted via the FDR method. The B-OR and B-Pval are results derived from the MR-BMA analysis, and the forest plot displays the IVW results. (Phosphatidylcholine(O-16:1/18:1) represents a specific type of phospholipid molecule with a defined composition. Here is a breakdown of its components: O-16:1: This notation refers to the fatty acid composition of the molecule. In this case, O-16:1 signifies that there is an unsaturated fatty acid with 16 carbon atoms and 1 double bond attached to the glycerol backbone at the first position; 18:1: Similarly, 18:1 denotes an 18-carbon atom unsaturated fatty acid with 1 double bond attached to the glycerol backbone at the second position. In essence, Phosphatidylcholine(O-16:1/18:1) specifies a phospholipid molecule consisting of a choline head group linked to a glycerol backbone, with a 16-carbon unsaturated fatty acid at the first position and an 18-carbon unsaturated fatty acid at the second position. The specific arrangement and composition of these components play a crucial role in the structure and function of the phospholipid within biological membranes. Phosphatidylcholine, Phosphatidylethanolamine, and Phosphatidylinositol have the same naming principles. Sphingomyelin(d40:2) is a specific type of sphingomyelin lipid molecule. The term "d40:2" refers to the fatty acid composition of the sphingomyelin molecule. In this case, "d40:2" indicates that the sphingomyelin molecule contains a 40-carbon backbone with 2 double bonds. Diacylglycerol(18:1/18:1) refers to a specific type of diacylglycerol lipid molecule. In this case, the notation "18:1/18:1" denotes the fatty acid composition of the two acyl chains attached to the glycerol backbone in the diacylglycerol molecule. Each "18:1" specifies that the fatty acid chain contains 18 carbon atoms and one double bond, also known as an oleic acid. In the context of lipid nomenclature, Triacylglycerol 50:5 is a specific way of expressing the composition of a triglyceride molecule. Here is a breakdown of what this designation means; "50:5" represents the fatty acid composition of the triglyceride molecule. In this case: "50" denotes the total number of carbon atoms in the three fatty acid chains. Therefore, the sum of carbon atoms in all three fatty acids is 50. "5" signifies the total number of double bonds present in the three fatty acid chains combined)

The causal effects of lipidomes on BC were evaluated through TSMR. The results indicated that glycerophospholipids, sphingolipids, and glycerolipids could potentially impact BC risk. Specifically, phosphatidylethanolamine and various phosphatidylinositol structures were found to be protective factors. In contrast, different forms of phosphatidylcholine displayed inconsistent effects on BC (Fig. 1). Notably, sphingomyelin, a member of the sphingolipids family, was associated with an increased risk of BC (OR = 1.05, 95%CI 1.02–1.08, *P* = 0.0036). On the other hand, diacylglycerol and several triacylglycerol variants within the glycerolipids family exhibited a protective role against BC (Fig. 1). The results of the different analysis methods are presented in Supplementary Table 2. The results from IVW are our primary reference metric. If the causal relationships determined by the other four analysis methods align with the direction of the IVW results and have a *P* < 0.05, it would greatly enhance our confidence in the establishment of the causal relationship.The results from both TSMR and BMA-MR analyses were generally consistent, except for phosphatidylcholine (O-18:1/20:4).

### Causal effects of lipidomes and ER^+^ BC

**Fig. 2:**
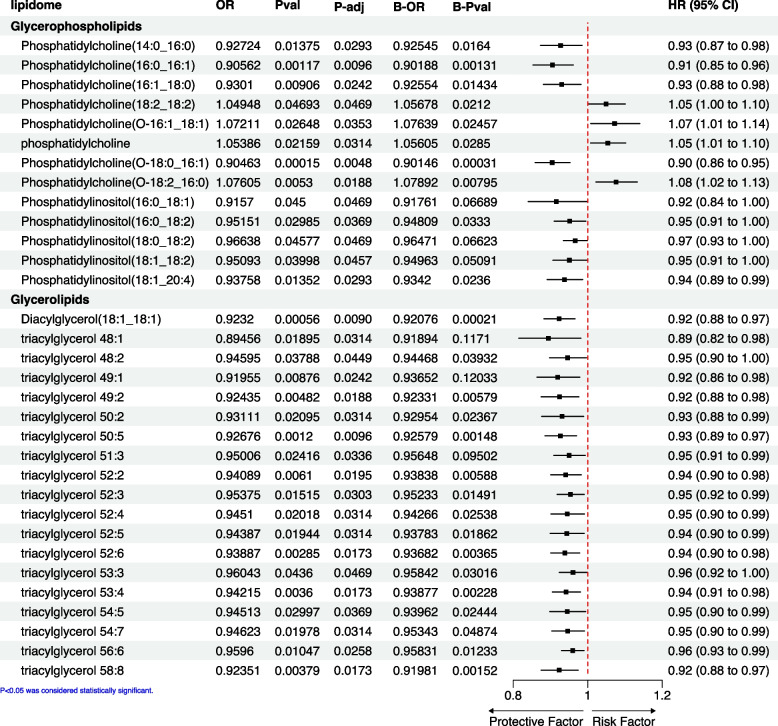
Forest plot of Mendelian randomization and Bayesian Model Averaging Mendelian Randomization method of the effect of lipidomes on ER^+^BC. The OR, P-val, and 95% CI are results obtained from the IVW method, while P-adj represents the outcomes adjusted via the FDR method. The B-OR and B-Pval are results derived from the MR-BMA analysis, and the forest plot displays the IVW results

The analysis of TSMR revealed potential causal effects of glycerophospholipids and glycerolipids on ER^+^BC. Specifically, phosphatidylinositols within the glycerophospholipids group continued to exhibit a protective role, while the impact of phosphatidylcholines varied depending on their structure (Fig. 2). Within the glycerolipids family, the diacylglycerol variant 18:1/18:1 and several triacylglycerols demonstrated a protective effect against ER^+^BC (Fig. 2). The results of the different analysis methods are presented in Supplementary Table 3. The results from IVW are our primary reference metric. If the causal relationships determined by the other four analysis methods align with the direction of the IVW results and have a *P* < 0.05, it would greatly enhance our confidence in the establishment of the causal relationship. The overall findings were consistent between TSMR and BMA-MR analyses, with a few exceptions that included specific phosphatidylinositols (16:0/18:0 and 18:0/18:2), as well as triacylglycerols (48:1, 49:1, and 53:3) (Fig. 2).

### Causal effects of lipidomes and ER^−^ BC

**Fig. 3:**
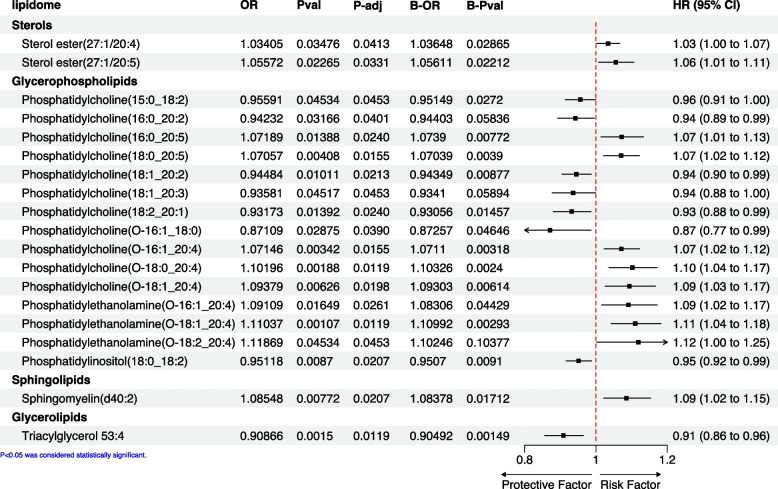
Forest plot of Mendelian randomization and Bayesian Model Averaging Mendelian Randomization method of the effect of lipidomes on ER.^−^BC. The OR, P-val, and 95% CI are results obtained from the IVW method, while P-adj represents the outcomes adjusted via the FDR method. The B-OR and B-Pval are results derived from the MR-BMA analysis, and the forest plot displays the IVW results. (Sterol ester (27:1/20:4) refers to a specific type of sterol ester lipid molecule. In this case, the notation "27:1/20:4" denotes the fatty acid composition of the two acyl chains attached to the sterol backbone in the sterol ester molecule. For the "27:1" component, it indicates that one of the fatty acid chains contains 27 carbon atoms and one double bond, which is commonly seen in monounsaturated fatty acids like oleic acid. For the "20:4" component, it specifies that the other fatty acid chain contains 20 carbon atoms and four double bonds, which is characteristic of polyunsaturated fatty acids like arachidonic acid)

The TSMR analysis indicated that glycerophospholipids, sphingolipids, glycerolipids, and sterols may have a significant impact on ER^−^BC. Among the glycerophospholipids, an increased incidence of ER^−^BC was associated with specific phosphatidylcholines, with the risk and protective effects varying depending on their molecular structure (Fig. 3). Furthermore, phosphatidylethanolamine emerged as a clear risk factor for ER^−^BC, while phosphatidylinositols exhibited a protective role. In the sphingolipids category, the presence of sphingomyelin was found to promote the development of ER^−^BC. Conversely, within the glycerolipids family, the triacylglycerol variant 53:4 demonstrated a protective effect against ER^−^BC. Notably, the sterols category showed a consistent protective effect against ER^−^BC incidence. The results of the different analysis methods are presented in Supplementary Table 4. The results from IVW are our primary reference metric. If the causal relationships determined by the other four analysis methods align with the direction of the IVW results and have a *P* < 0.05, it would greatly enhance our confidence in the establishment of the causal relationship. Overall, the findings from both the TSMR and BMA-MR analyses were largely in agreement, with a few exceptions, such as phosphatidylethanolamine (O-18:2/20:4) (Fig. 3).

### Sensitivity analysis

A sensitivity analysis was performed to assess the robustness of the findings, and the results are presented in Supplementary Tables 2–4. In the context of BC, neither the IVW test nor the MR-Egger test indicated any heterogeneity. The absence of substantial heterogeneity was similarly observed for ER^−^ and ER^+^ BC. The MR-Egger intercept test yielded a p-value greater than 0.05, suggesting the absence of horizontal pleiotropy in the results. Furthermore, the MR-PRESSO test did not detect pleiotropy, thus confirming the accuracy of the findings. The scatter plots depicting the MR analyses are in Supplementary Figs. 1, 2 and 3. Examination of the funnel plot showed no significant heterogeneity (Supplementary Figs. 1, 2 and 3). Additionally, no single SNP significantly impacted the MR estimates, as demonstrated by the leave-one-out analysis. While a few SNPs were found to potentially influence the results for specific ER status subgroups, their odds ratio values consistently remained on the same side of the zero line (Supplementary Figs. 1, 2 and 3).

## Discussion

This is the first study to comprehensively and meticulously explore the impact of various structurally different lipidomes on BC incidence, presenting a panoramic view of the research on 179 lipidomes. By examining 179 lipidome traits as exposures and BC as the outcome, the study delved into the intricate causal associations between lipidomes and BC occurrence. Our findings revealed significant effects of glycerophospholipids, sphingolipids, and glycerolipids on BC risk. Specifically, for ER^+^BC, phosphatidylcholine and phosphatidylinositol within glycerophospholipids continued to play significant roles, along with the importance of glycerolipids. However, The study did not observe a noteworthy impact of sphingolipids on ER^+^BC. In the case of ER^−^BC, not only glycerophospholipids, sphingolipids, and glycerolipids exerted an influence, but the protective effect of sterols was also discovered. It is worth noting that the prominence of glycerolipids (diglycerides and triglycerides), which played a vital role in ER^+^BC, was minimal in ER^−^BC. Conversely, phosphatidylethanolamine within the glycerophospholipid family played an important role in ER^−^BC.

The findings of our study highlight the consistent protective role of phosphatidylinositol within glycerophospholipids for overall BC, ER^+^BC, and ER^−^BC. Our results demonstrate that various structurally diverse forms of phosphatidylinositol significantly decrease the risk of BC and ER^+^BC, whereas only Phosphatidylinositol (18:0/18:2) significantly affects ER^−^BC. Phosphatidylinositols are well-known for their involvement in intracellular signaling pathways [31, 32], particularly through the Phosphatidylinositol 3-kinase (PI3K) pathway, which regulates various cellular functions, including lipid metabolism. It is noteworthy that this pathway is frequently mutated or activated in BC tissues [33, 34]. Phosphatidylinositol stands out among other phospholipids due to its limited fatty acid composition and characteristic patterns in mammalian cells [35, 36]. Changes in the composition of phosphatidylinositol can be induced by specific stimuli, thereby regulating the PI3K signaling pathway [37]. Previous studies have shown that the matrix composition of BC tissues is predominantly occupied by phosphatidylinositol (18:0/20:4), with no significant inter-individual variation. Phosphatidylinositol (18:0/20:3) tends to distribute in the adjacent stromal area, while phosphatidylinositol (18:0/18:1) tends to cluster in the central region of tumor cell populations. Discrimination between these two distinct tissue areas can be achieved by the expression of either phosphatidylinositol (18:0/18:1) or phosphatidylinositol (18:0/20:3). The association between the accumulation of phosphatidylinositol (18:0/20:3) and stromal contact, as well as nodal status, suggests a plausible hypothesis that this accumulation in BC cells may contribute to their invasion capacity [38]. This hypothesis is supported by evidence indicating that the accumulation of phosphatidylinositol (18:0/20:3) not only affects cellular membrane fluidity but also influences the activity of the PI3K signaling pathway [39–42]. In previous studies, higher levels of phosphatidylinositol (16:0/18:1) were found in the epithelial region compared to the stromal area, and its distribution in malignant BC tissues was significantly higher than in benign breast tumors [38]. In our study, we observed that phosphatidylinositol (16:0/18:1) exhibited a significant protective effect on overall BC and ER^+^BC. This discrepancy underscores a potentially multifaceted role of phosphatidylinositol (16:0/18:1) in BC, suggesting that its impact may not be uniformly pro-tumorigenic and could vary depending on the context or subtype of BC. Notably, phosphatidylinositol (18:0/18:2), which showed no significant difference in distribution within the stroma and epithelial tissues according to previous literature, did not demonstrate any significant differences between BC and benign tumors [38]. However, in our study, phosphatidylinositol (18:0/18:2) displayed a significant protective effect among overall BC, ER^+^BC, and ER^−^BC. Regarding phosphatidylethanolamine(O-18:2/18:2), it played a protective role in overall BC, while in ER^−^BC, specific subtypes (O-16:1/20:4, O-18:1/20:4, and O-18:2/20:4) exerted clear promoting effects. Phosphatidylethanolamine, an essential component for ferroptosis, has been found to play an important role in the heterogeneity of tumors in triple-negative BC (TNBC) [43, 44], which might an essential breakthrough for further investigation to explore the potential dual effects of phosphatidylethanolamine on BC. Additionally, the downregulation of RARRES2, which regulates lipid metabolism reprogramming and mediates the development of brain metastasis in TNBC, has been found to lead to an increase in phosphatidylethanolamine and phosphatidylcholine levels. This highlights the significance of investigating the role of RARRES2 and its impact on phosphatidylethanolamine in BC [45]. Our findings shed light on the distinct effects of different phospholipid subtypes, such as phosphatidylinositol and phosphatidylethanolamine, on BC. The observed protective effects of certain phospholipids in specific BC subtypes provide valuable insights into the underlying mechanisms and potential therapeutic targets. The relationship between lipid metabolism and BC progression, merits further investigation. One noteworthy example is the inconsistent effects observed with phosphatidylcholine, as different structural forms of phosphatidylcholine exert entirely opposing roles. Further research is warranted to unravel the molecular mechanisms involved and to explore the clinical implications of these findings.

The randomized results from Mendelian randomization have also presented us with some questions and challenges. In our traditional understanding, diacylglycerols and triglycerides increase the risk of various diseases, with a steady state existing between monoglycerides, diacylglycerols, and triglycerides synthesis. Previous cohort studies have indicated an increased risk of BC with elevated triglyceride levels [46]. However, evidence from evidence-based medicine suggests that high triglyceride levels do not lead to an increased risk of BC [47, 48]. Our research findings suggest that triglycerides may be a protective factor for ER^+^BC, but this phenomenon is not clearly evident in ER^−^BC. This seemingly novel perspective is supported by existing evidence as well. Similarly, other Mendelian randomization studies focusing on lipid metabolism and tumor risk also indicate triglycerides as a protective factor for BC, particularly ER^+^BC [49–54], aligning with our research results. However, as with most complex diseases, common variants identified in GWAS can only explain a small portion of the total heritability of the disease, especially in the case of cancer. Rare variants throughout the genome may also play a significant role in disease development. Therefore, a combination of basic and clinical research is needed to further clarify causal relationships [55]. Some scholars have suggested that these intriguing findings may indicate a complex relationship between lipid metabolism, estrogen, and BC [51]. The consistency of these results across multiple studies from different data sources further enhances our confidence in the reliability of our findings. High mammographic breast density (MBD) has been widely recognized as a strong risk factor for the development of BC. Volumetric percent density (VPD) has emerged as a quantitative standard for assessing MBD. Previous study revealed that lipid species inversely associated with VPD predominantly belonged to the triacylglycerol (*N* = 43) and diacylglycerol (*N* = 7) sub-pathways [56]. These lipid species align with the protective effects observed in our research. Furthermore, investigations into the lipid profiles of plasma-derived extracellular vesicles revealed a higher concentration of triglycerides in TNBC [57], which is consistent with the low prominence of triglycerides as protective factors in our findings for ER-BC. However, it is worth noting that triglycerides and diglycerides exhibit diverse structural variations. Previous studies have predominantly explored triglycerides as a whole without specific subdivisions based on their structural forms. Hence, further research is warranted to identify which specific structural forms of triglycerides can exert preventive effects [56]. Our study highlights the potential importance of triglyceride subtypes in modulating BC risk, particularly in different molecular subtypes. These findings underscore the complexity of lipid metabolism in BC aetiology, and further investigation is essential to unravel the precise mechanisms involved.

### Strengths and limitations

This study has several strengths. Firstly, we are the first to utilize the TSMR approach to thoroughly investigate the association between lipidomes and BC. This study encompasses a collection of genetic data and minimizes potential confounding factors to the greatest extent possible. Secondly, we applied the BMA-MR method to replicate our results, enhancing the accuracy of our findings. Thirdly, we conducted analyses based on different ER statuses to examine overall BC and specific subgroups, allowing us to apply our conclusions to the population more precisely.

However, there are certain limitations to our study. Firstly, during screening SNPs of lipidomes, a less stringent threshold of *P* < 5*10^–6^ was utilized instead of the conventional threshold of 5*10^–8^ to acquire enough SNPs. Secondly, the majority of participants in this study were of European descent, which restricts the generalizability of our findings to other populations.Thirdly, due to the challenge of securing sufficiently high-confidence and quality SNPs necessary for multivariable Mendelian randomization analysis (MVMR), our study does not currently present MVMR results at present. We aim to refine our algorithms in future research to achieve these outcomes. Meanwhile, MR analysis assumes that the impact of genetic variants on the outcome is entirely mediated by the exposure [58]. Further, the inconsistency in significance assessment between TSMR and BMA-MR could be attributed to the differing algorithms used by the two analysis methods. TSMR typically employs frequentist statistical methods, whereas BMA-MR utilizes Bayesian methods. Bayesian methods estimate parameters by introducing prior distributions and calculating posterior distributions, offering unique advantages in handling uncertainty and integrating external information. However, this may also result in associations that are significant in frequentist analyses becoming non-significant in Bayesian analyses [59, 60]. BMA-MR analysis may be more stringent than traditional frequentist methods in some cases, requiring stronger evidence to support causal inference. This means that results deemed significant in TSMR analysis may not remain significant in MR-BMA analysis when the data support is insufficient. Therefore, MR techniques can only assess causal relationships and cannot provide deeper insights into the underlying mechanisms of how lipidomes contribute to breast cancer prevention. Therefore, further research is needed to elucidate the potential processes underlying the novel insights generated by our study.

## Conclusion

Our study provide significant insights into the association between specific lipidomes and the risk of BC. The findings indicate thatphosphatidylinositol and triglycerides would decrease the incidence of BC, suggesting their potential protective role. Furthermore, the study highlights the complexity of lipid metabolism in BC by uncovering the diverse structural variations of lipidomes and their potential differential effects in different molecular subtypes. These findings contribute to our understanding of the role of lipidomes, such as phosphatidylinositol and triglycerides, in modulating BC risk and emphasize the need for further research to elucidate the underlying mechanisms.

### Supplementary Information

**Supplementary Material 1.**

**Supplementary Material 2.**

**Supplementary Material 3.**

**Supplementary Material 4.**

**Supplementary Material 5.**

## Data Availability

No datasets were generated or analysed during the current study.

## Abbreviations

BC: Breast cancer
ER^+^ BC: Estrogen receptor-positive BC
ER^−^ BC: Estrogen receptor-negative BC
MR: Mendelian randomization
RCTs: Randomized controlled trials
SNPs: Single nucleotide polymorphisms
IVs: Instrumental variables
TSMR: Two-Sample Mendelian randomization
FDR: False Discovery Rate
BMA-MR: Bayesian model averaging multivariate mendelian randomization
GWAS: Genome-Wide Association Study
BCAC: Breast Cancer Association Consortium
LD: Linkage disequilibrium
MR-PRESSO: MR Pleiotropy RESidual Sum and Outlier
IVW: Inverse variance weighting
