# Supplementary figures and images for: The impact of lipidome on breast cancer: a Mendelian randomization study (part 1 of 3)
Source: Lipids Health Dis. 2024 Apr 15;23:109. doi: 10.1186/s12944-024-02103-2 (PMC11017498; doi:10.1186/s12944-024-02103-2)

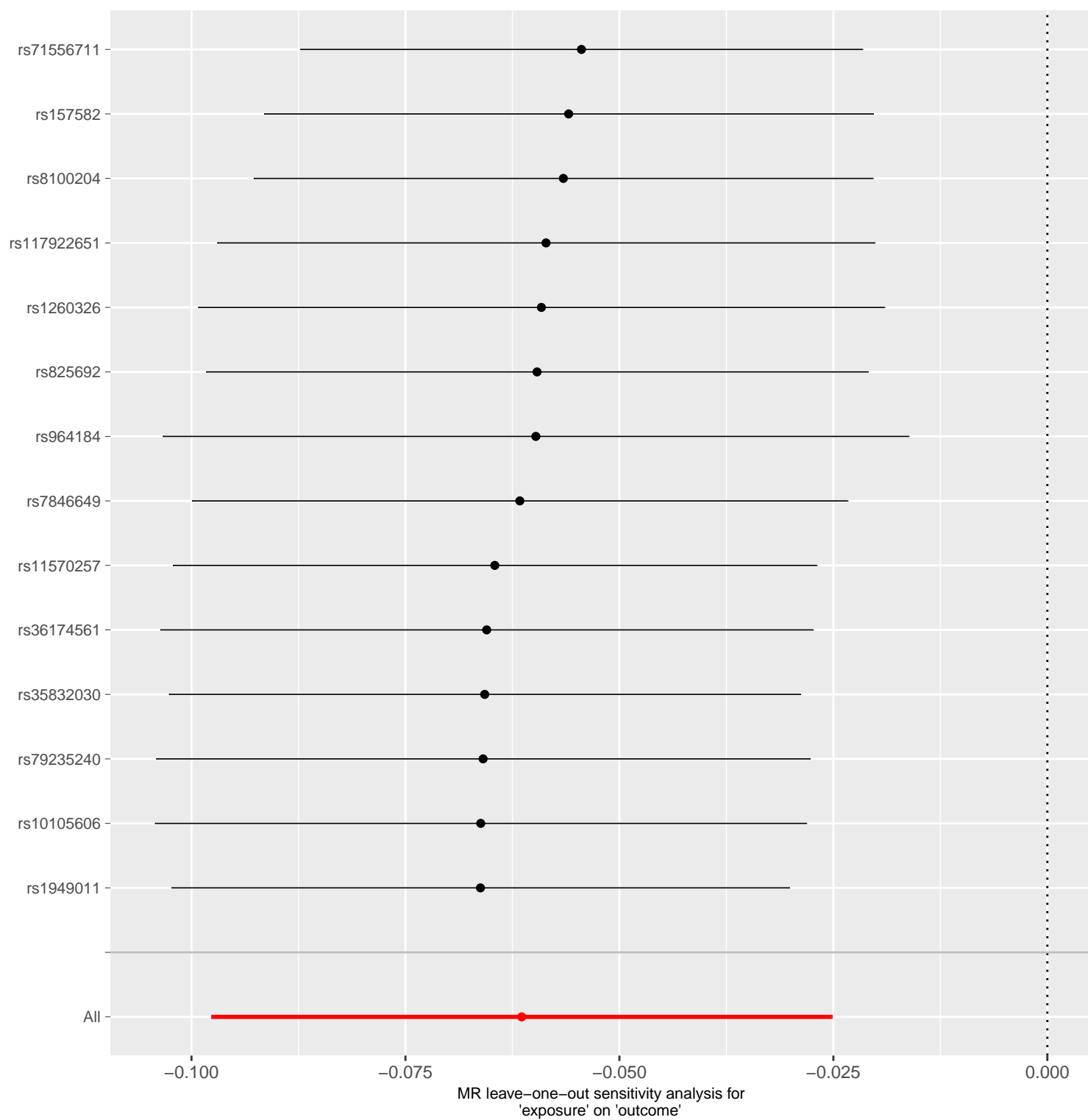

Supplement: Supplementary file 2 — Supplementary Material 2. [file 12944_2024_2103_MOESM2_ESM.zip › sFigure1∩╝êlipidomes-BC∩╝ë/GCST90277403/sensitivity-analysis.pdf]

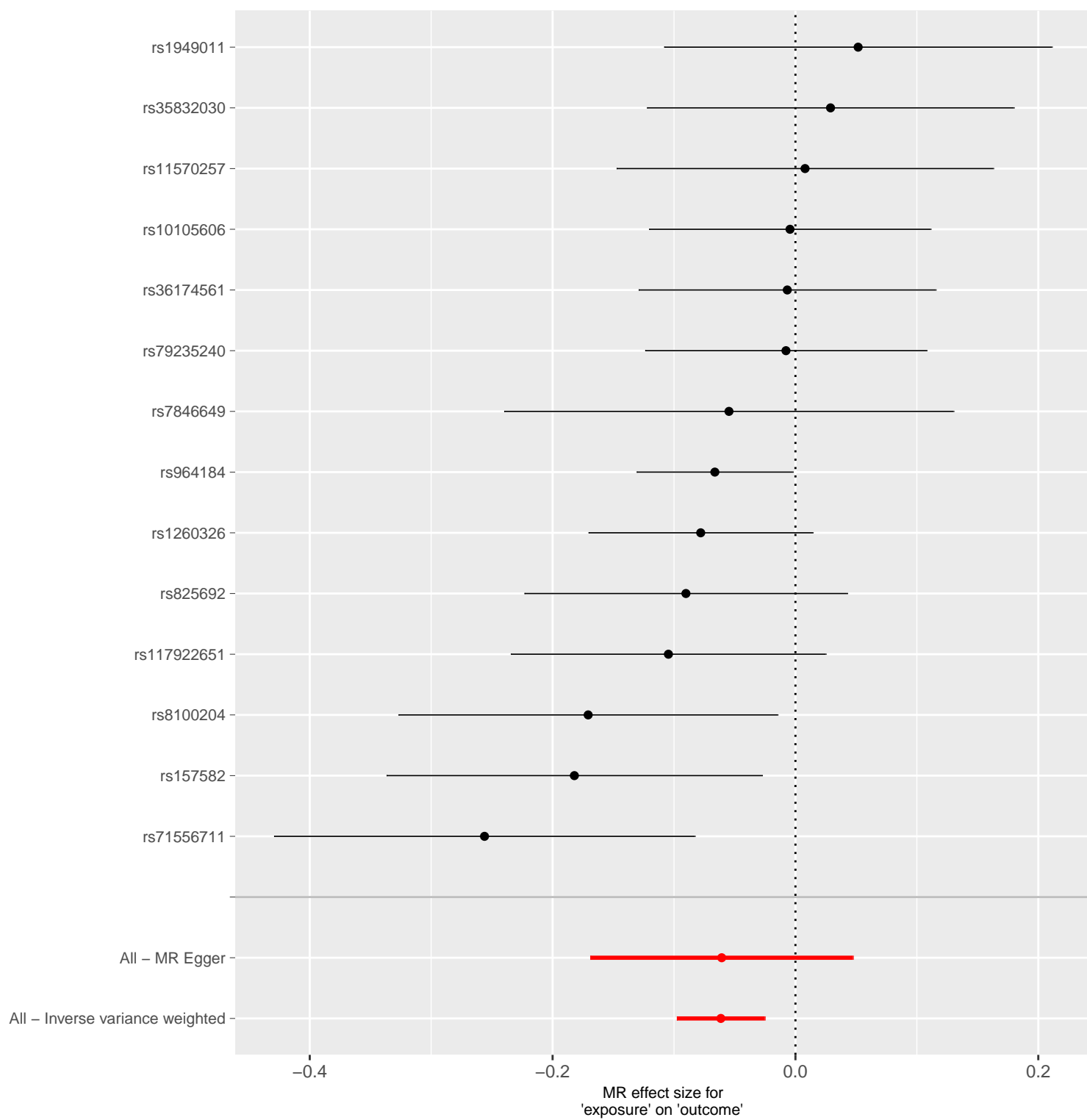

Supplement: Supplementary file 2 — Supplementary Material 2. [file 12944_2024_2103_MOESM2_ESM.zip › sFigure1∩╝êlipidomes-BC∩╝ë/GCST90277403/forest.pdf]

# MR Method

- Inverse variance weighted
- MR Egger

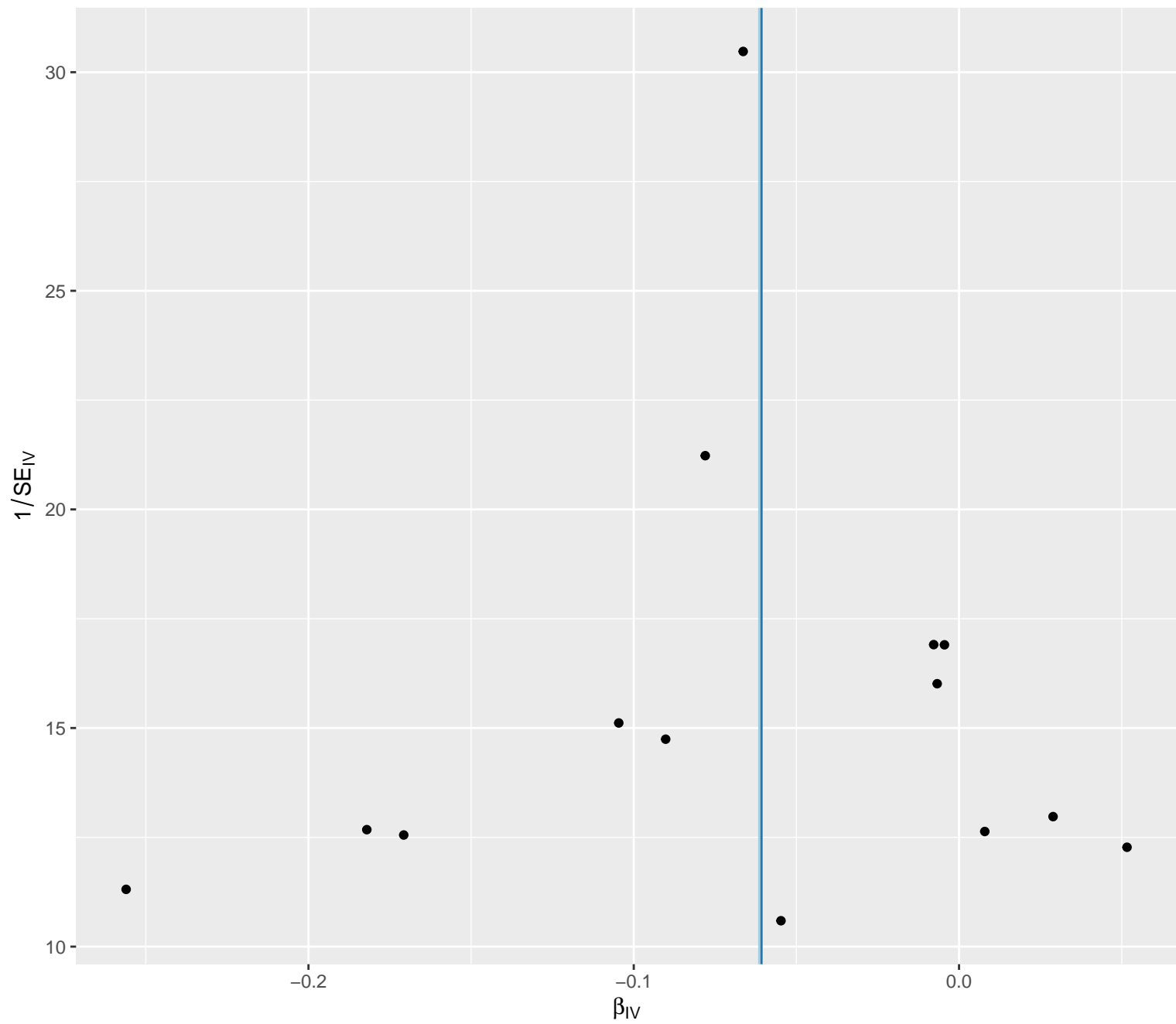

Supplement: Supplementary file 2 — Supplementary Material 2. [file 12944_2024_2103_MOESM2_ESM.zip › sFigure1∩╝êlipidomes-BC∩╝ë/GCST90277403/funnelplot.pdf]

# MR Test

- Inverse variance weighted
- MR Egger
- Simple mode
- Weighted median
- Weighted mode

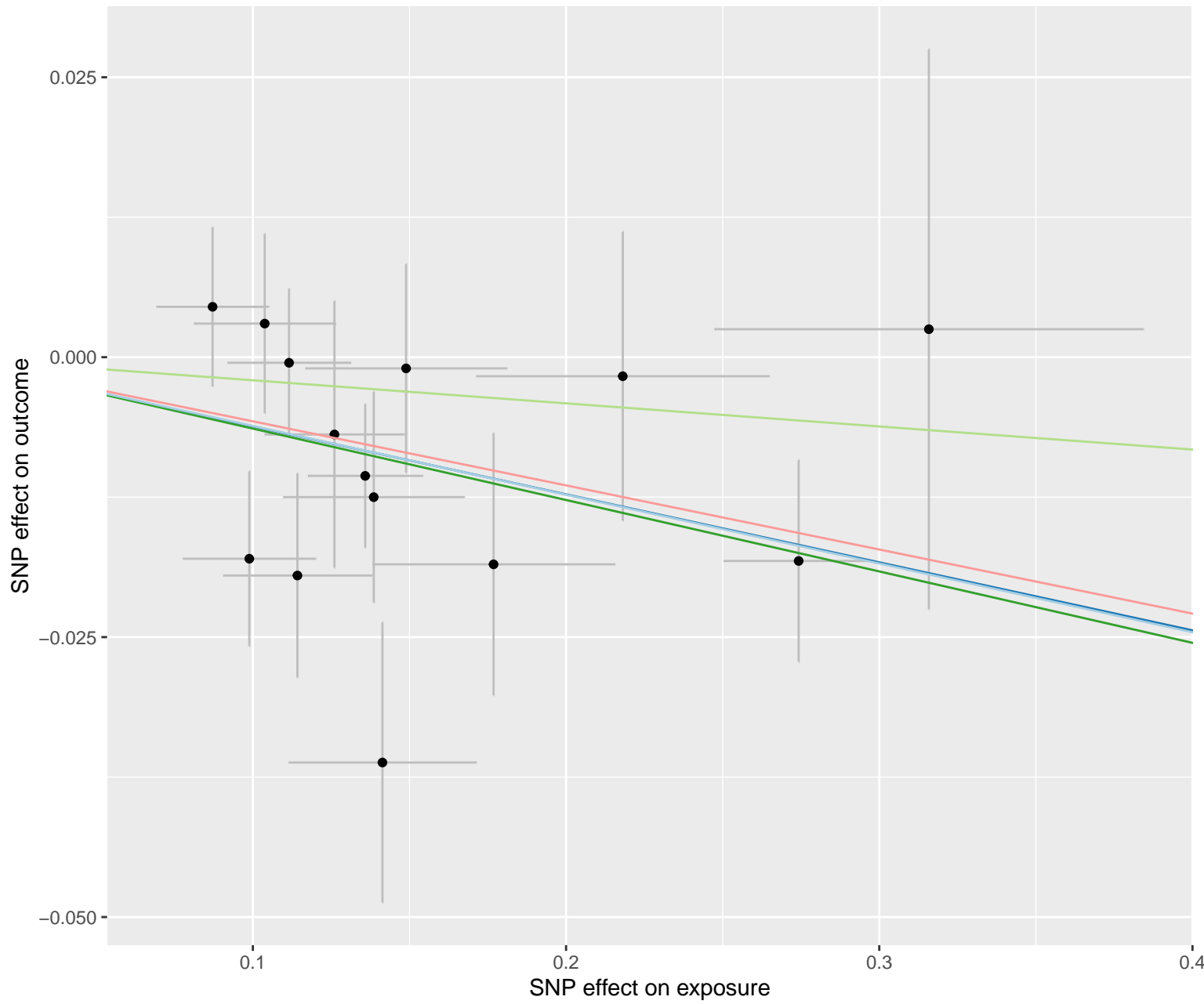

Supplement: Supplementary file 2 — Supplementary Material 2. [file 12944_2024_2103_MOESM2_ESM.zip › sFigure1∩╝êlipidomes-BC∩╝ë/GCST90277403/scatter.pdf]

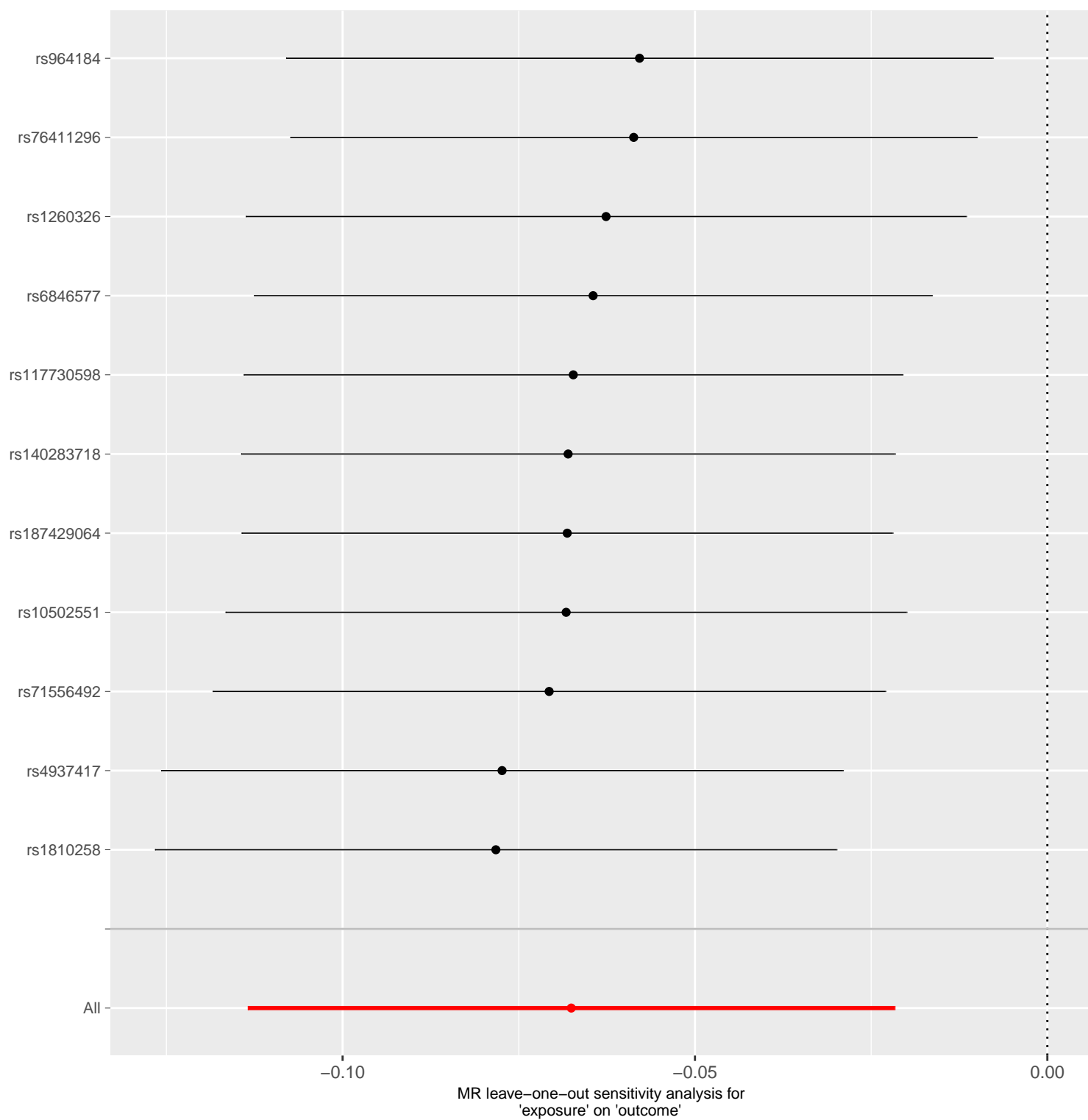

Supplement: Supplementary file 2 — Supplementary Material 2. [file 12944_2024_2103_MOESM2_ESM.zip › sFigure1∩╝êlipidomes-BC∩╝ë/GCST90277386/sensitivity-analysis.pdf]

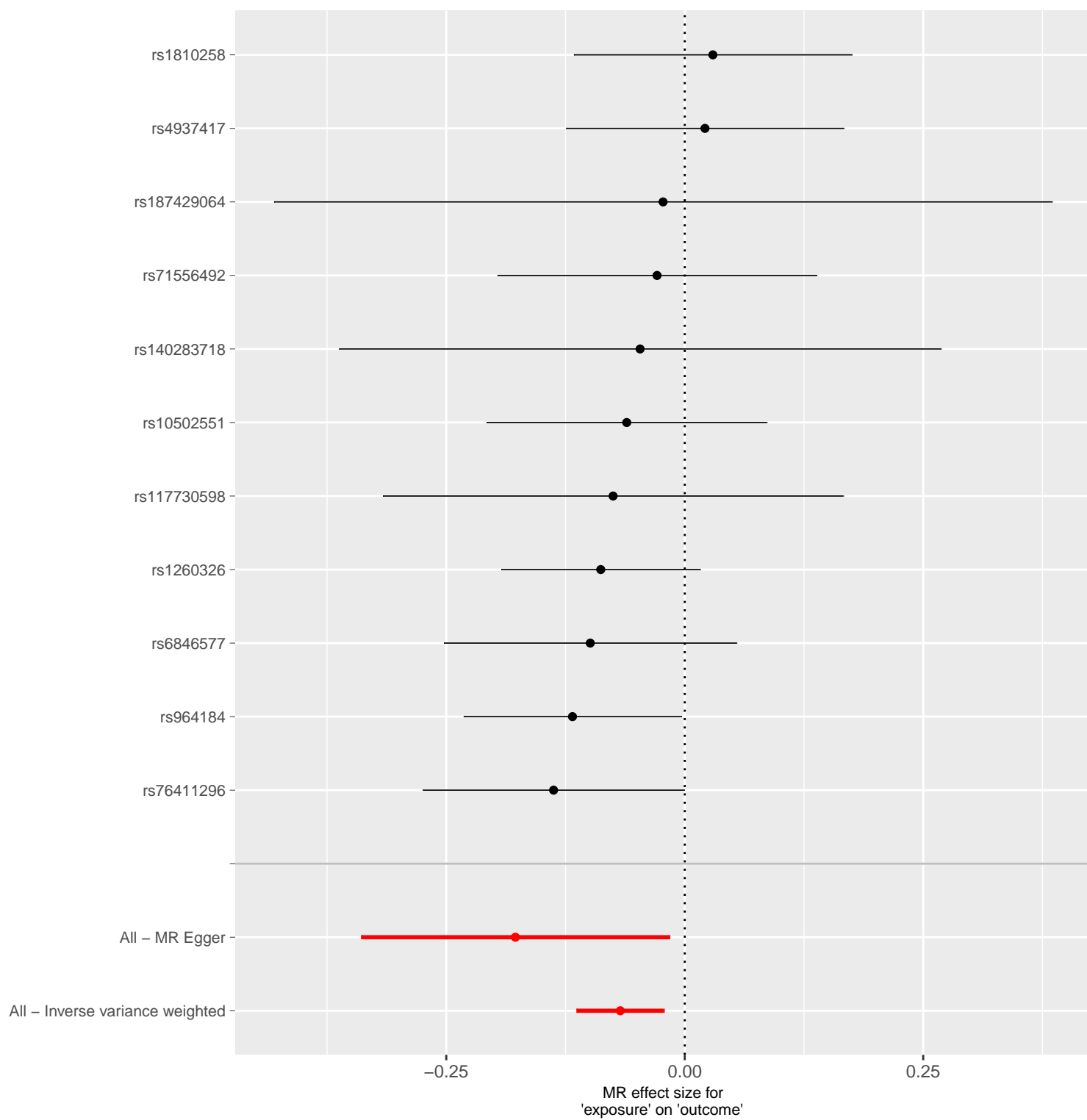

Supplement: Supplementary file 2 — Supplementary Material 2. [file 12944_2024_2103_MOESM2_ESM.zip › sFigure1∩╝êlipidomes-BC∩╝ë/GCST90277386/forest.pdf]

# MR Method

- Inverse variance weighted
- MR Egger

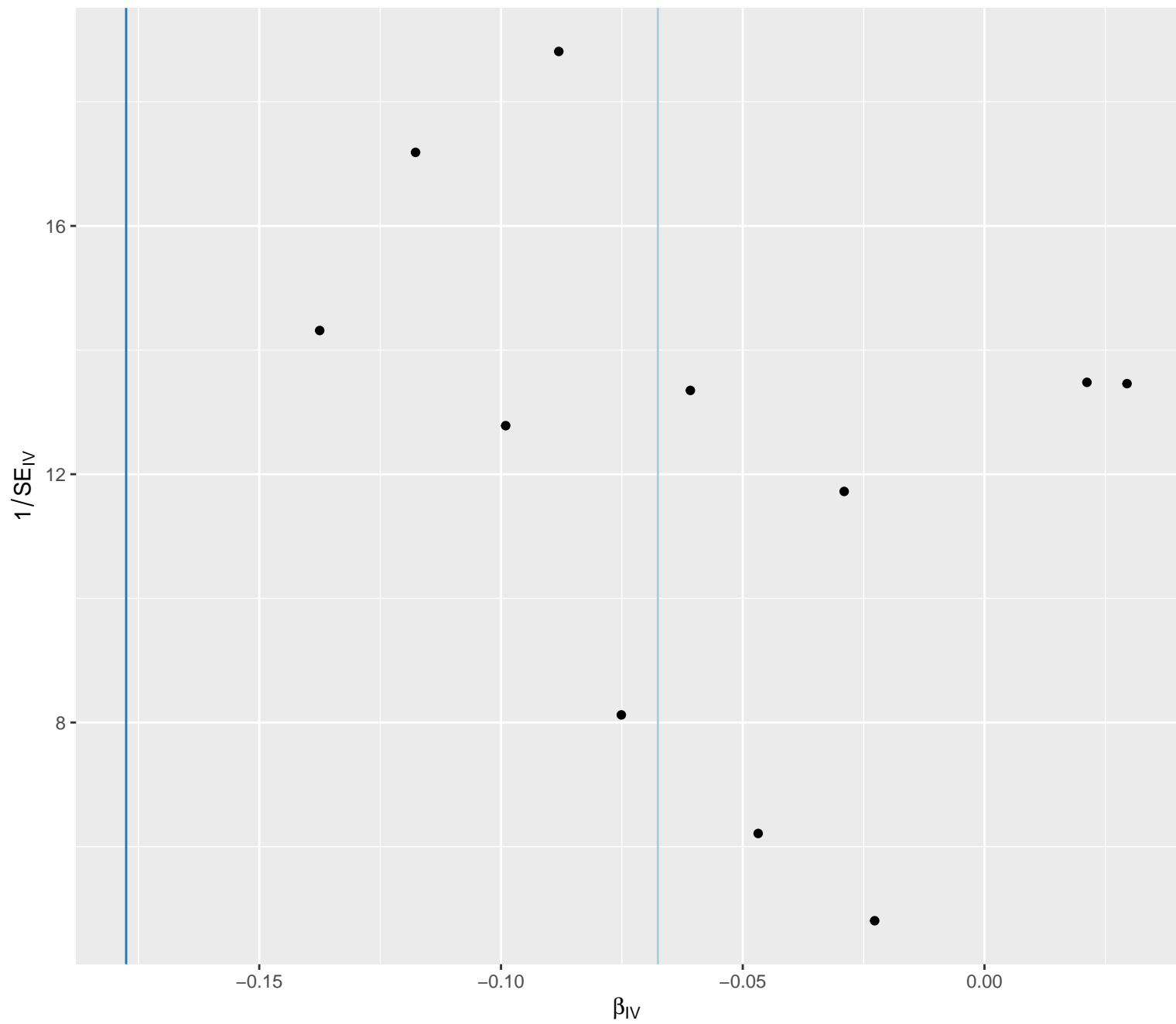

Supplement: Supplementary file 2 — Supplementary Material 2. [file 12944_2024_2103_MOESM2_ESM.zip › sFigure1∩╝êlipidomes-BC∩╝ë/GCST90277386/funnelplot.pdf]

# MR Test

- Inverse variance weighted
- MR Egger
- Simple mode
- Weighted median
- Weighted mode

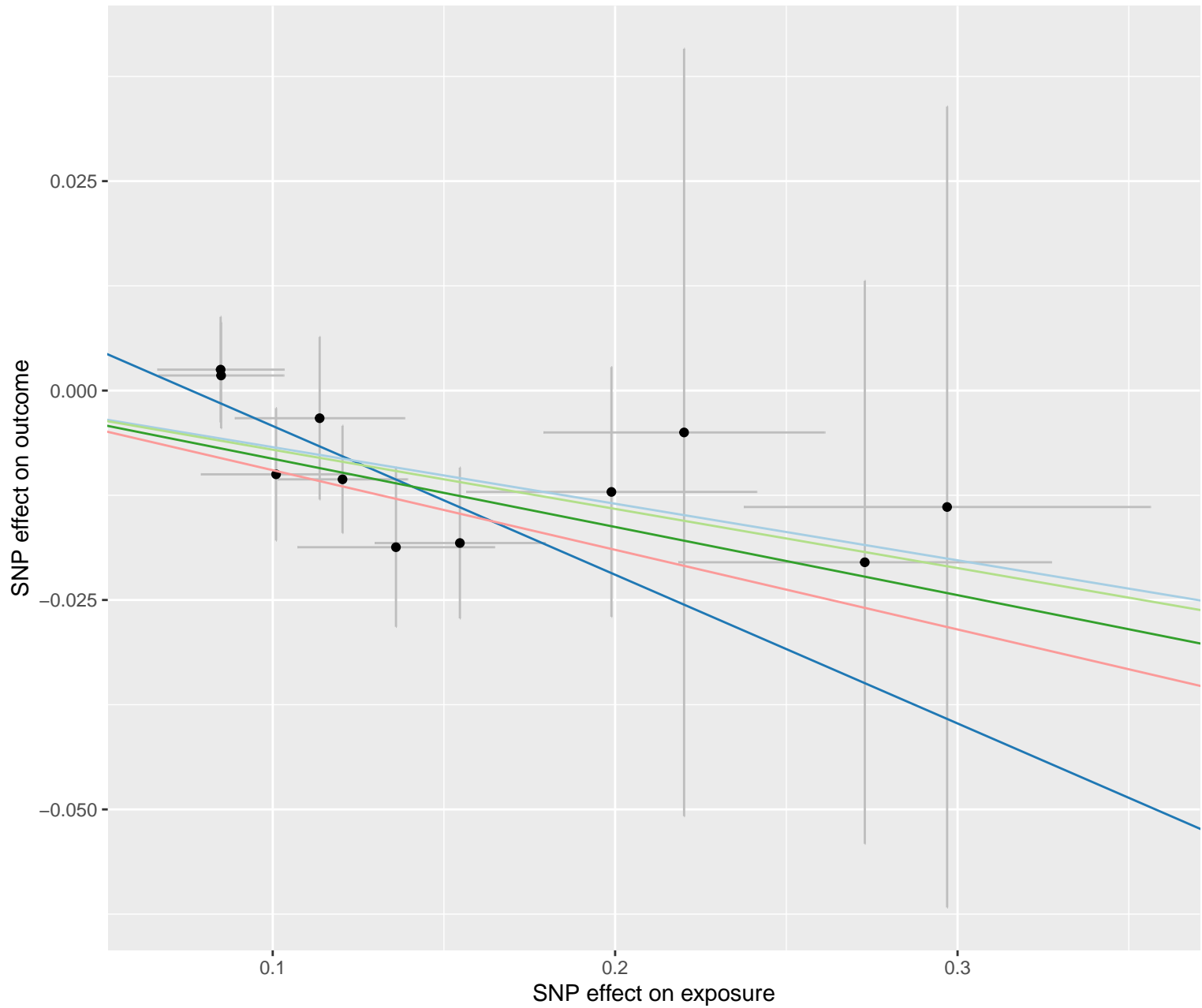

Supplement: Supplementary file 2 — Supplementary Material 2. [file 12944_2024_2103_MOESM2_ESM.zip › sFigure1∩╝êlipidomes-BC∩╝ë/GCST90277386/scatter.pdf]

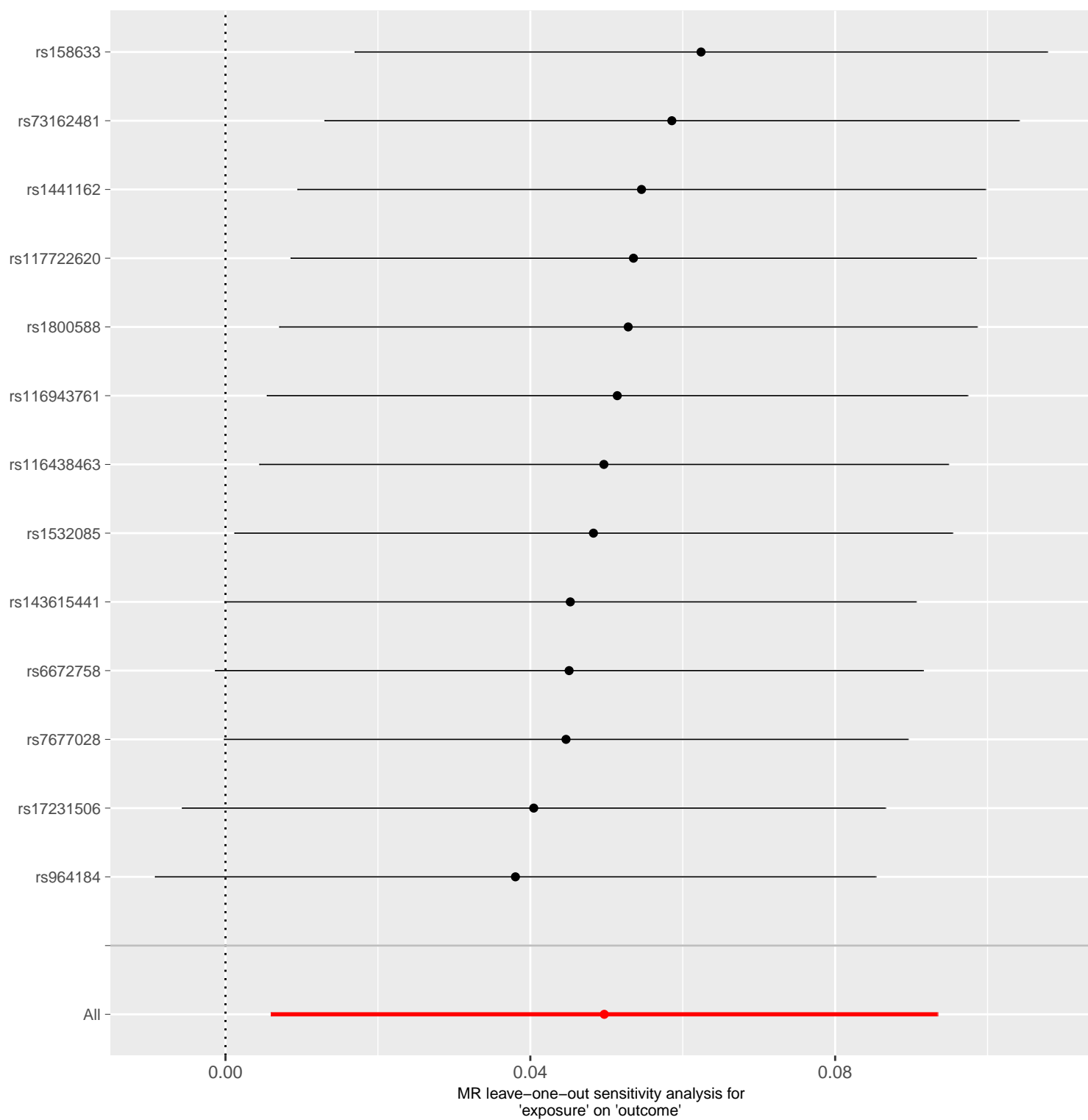

Supplement: Supplementary file 2 — Supplementary Material 2. [file 12944_2024_2103_MOESM2_ESM.zip › sFigure1∩╝êlipidomes-BC∩╝ë/GCST90277327/sensitivity-analysis.pdf]

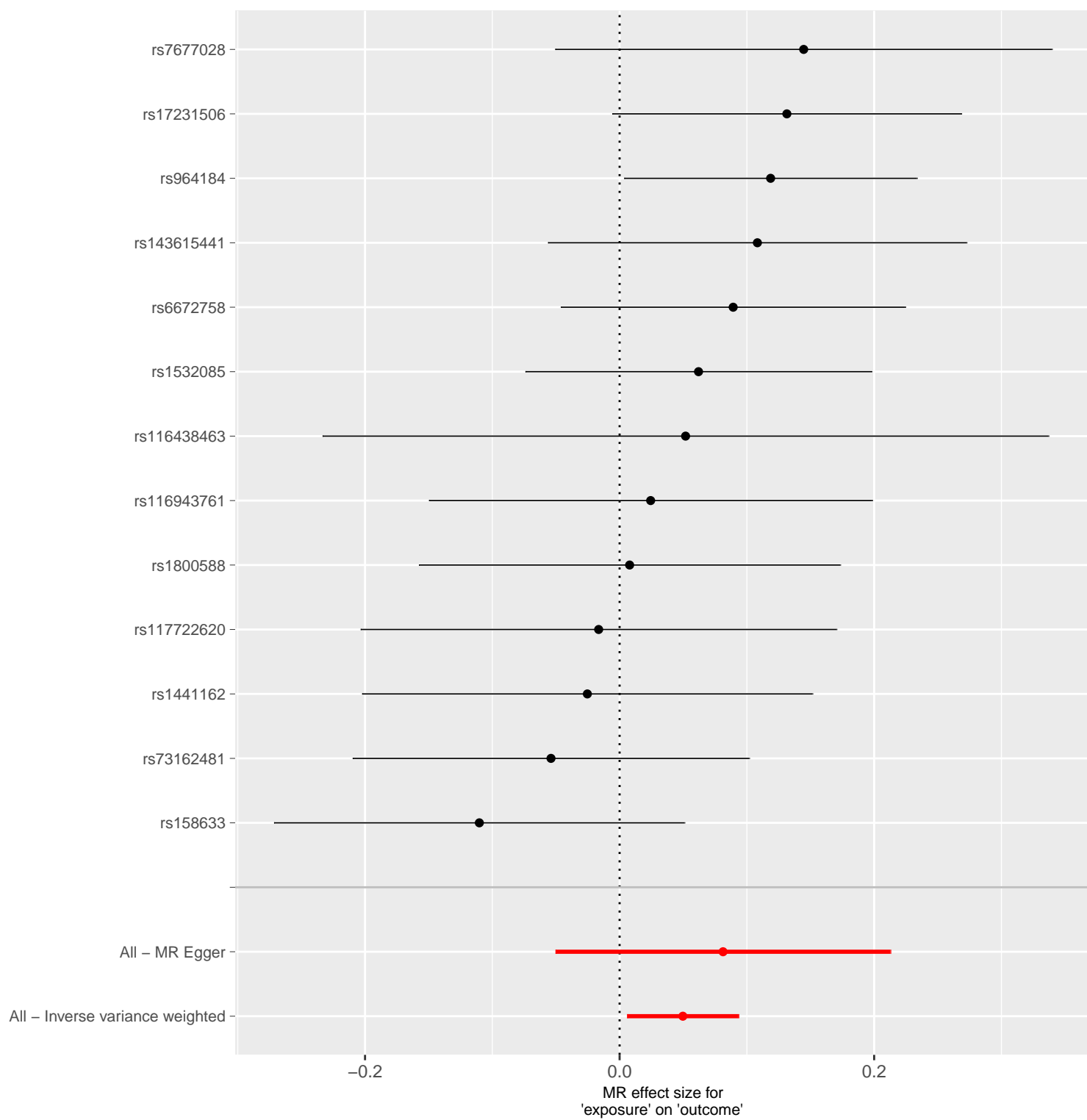

Supplement: Supplementary file 2 — Supplementary Material 2. [file 12944_2024_2103_MOESM2_ESM.zip › sFigure1∩╝êlipidomes-BC∩╝ë/GCST90277327/forest.pdf]

# MR Method

- Inverse variance weighted
- MR Egger

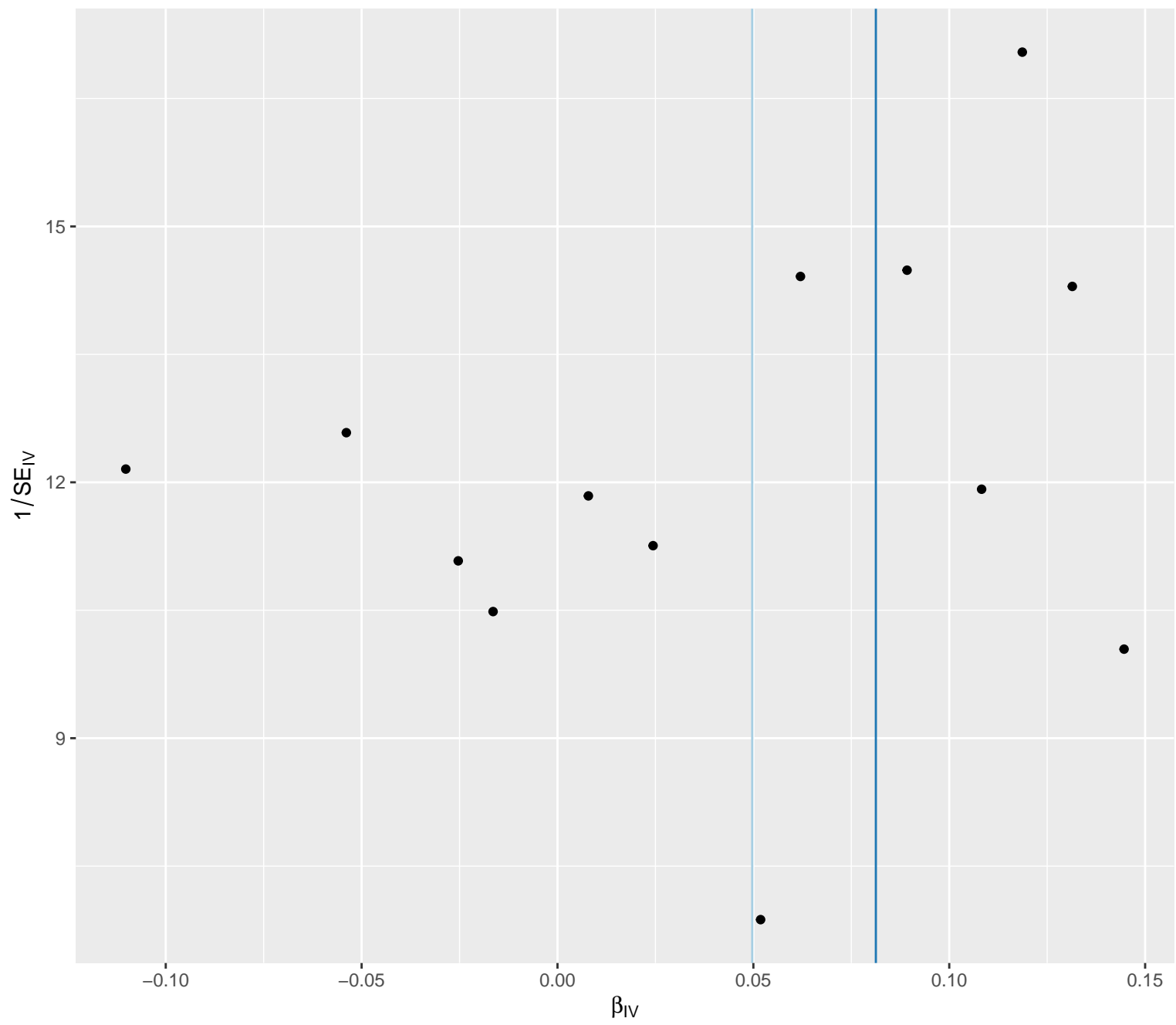

Supplement: Supplementary file 2 — Supplementary Material 2. [file 12944_2024_2103_MOESM2_ESM.zip › sFigure1∩╝êlipidomes-BC∩╝ë/GCST90277327/funnelplot.pdf]

# MR Test

- Inverse variance weighted
- MR Egger
- Simple mode
- Weighted median
- Weighted mode

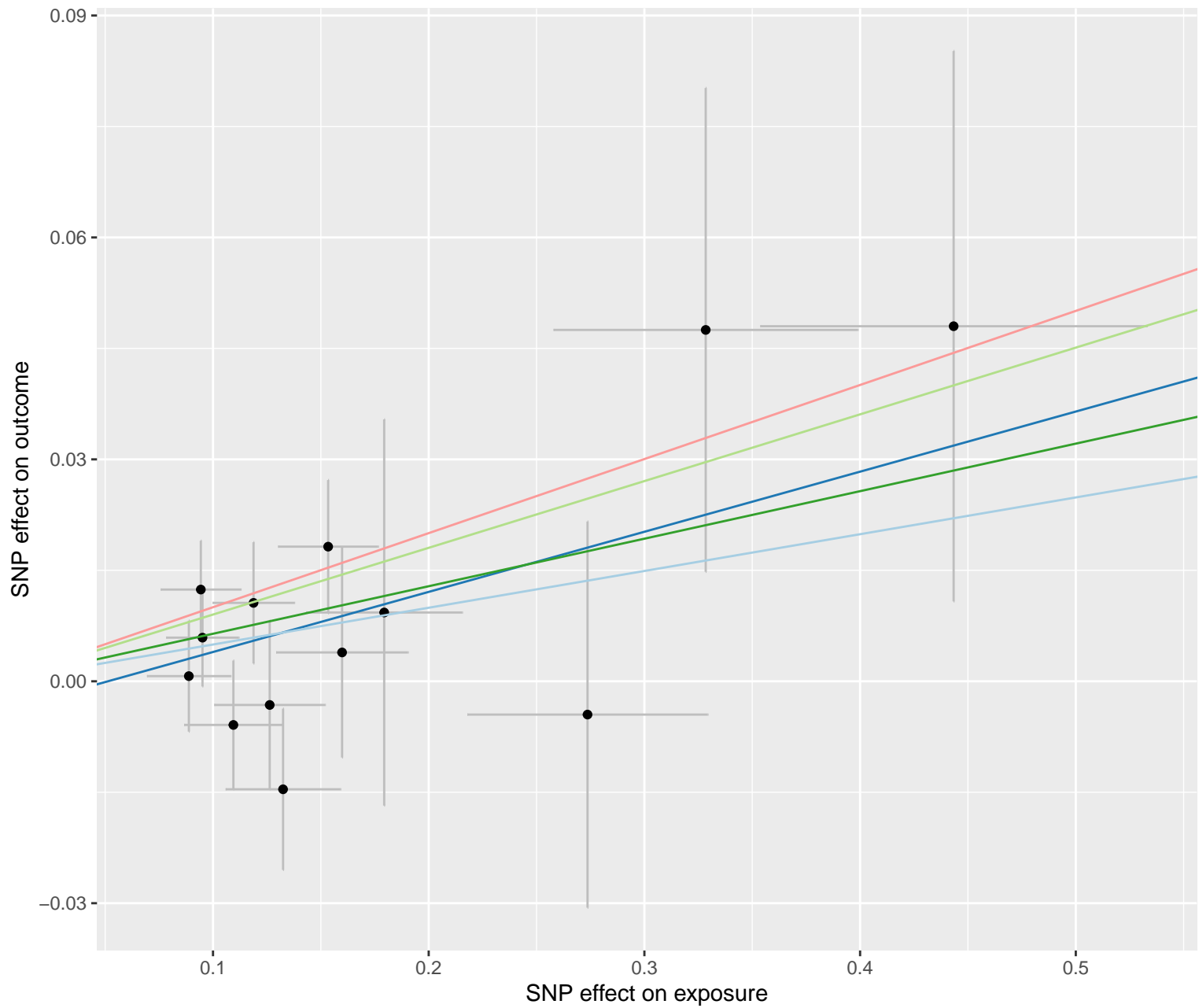

Supplement: Supplementary file 2 — Supplementary Material 2. [file 12944_2024_2103_MOESM2_ESM.zip › sFigure1∩╝êlipidomes-BC∩╝ë/GCST90277327/scatter.pdf]

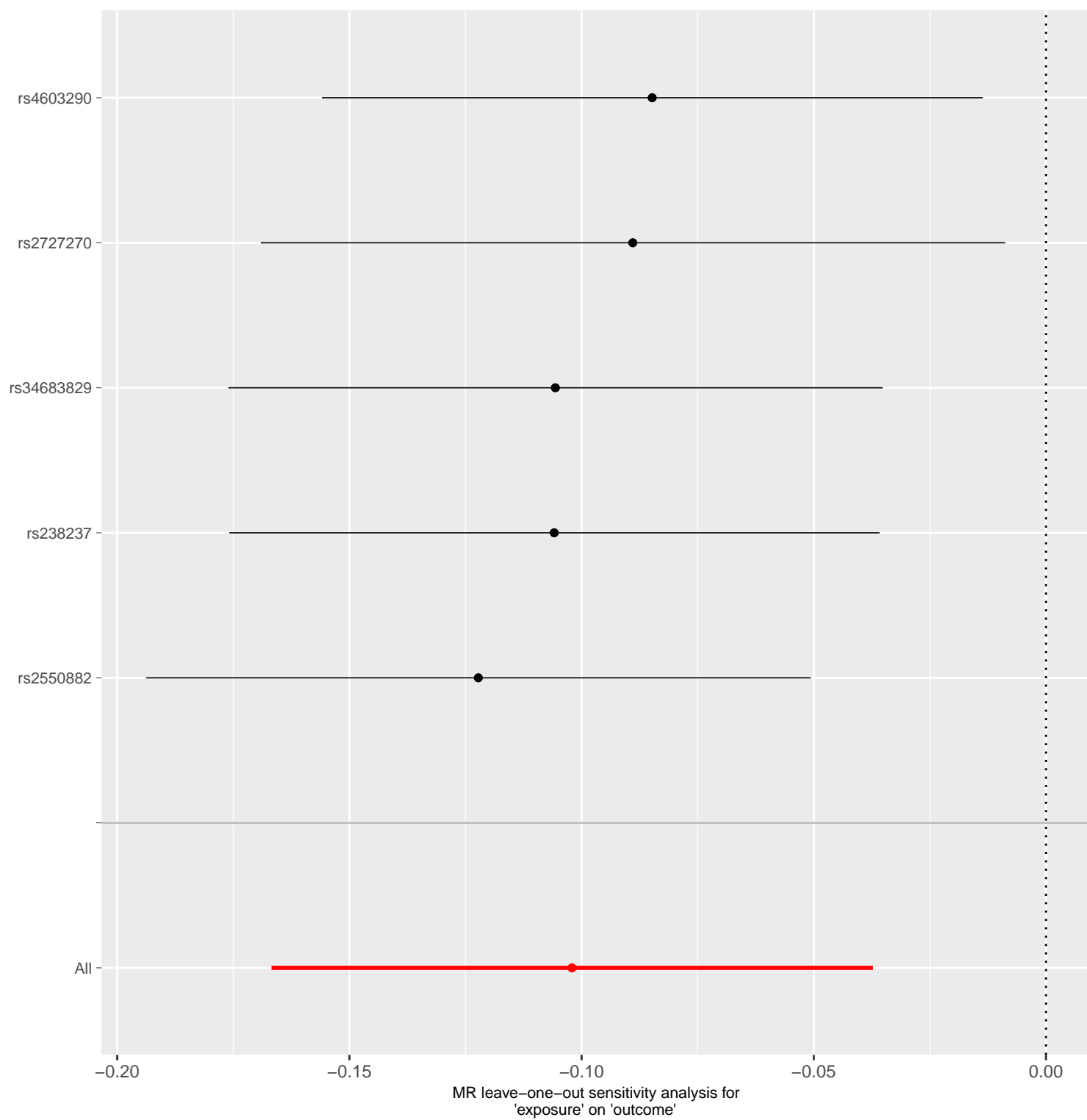

Supplement: Supplementary file 2 — Supplementary Material 2. [file 12944_2024_2103_MOESM2_ESM.zip › sFigure1∩╝êlipidomes-BC∩╝ë/GCST90277356/sensitivity-analysis.pdf]

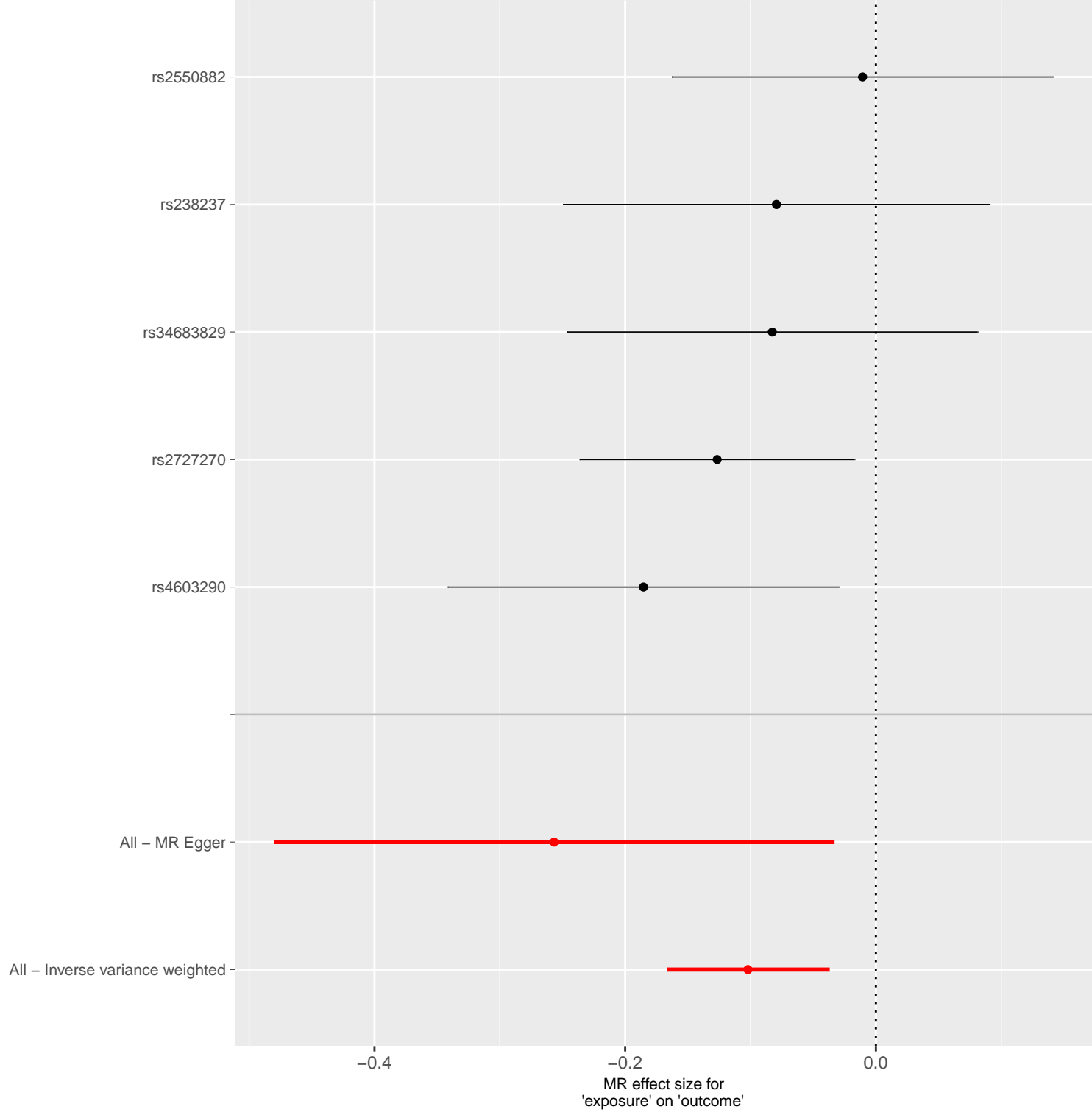

Supplement: Supplementary file 2 — Supplementary Material 2. [file 12944_2024_2103_MOESM2_ESM.zip › sFigure1∩╝êlipidomes-BC∩╝ë/GCST90277356/forest.pdf]

# MR Method

- Inverse variance weighted
- MR Egger

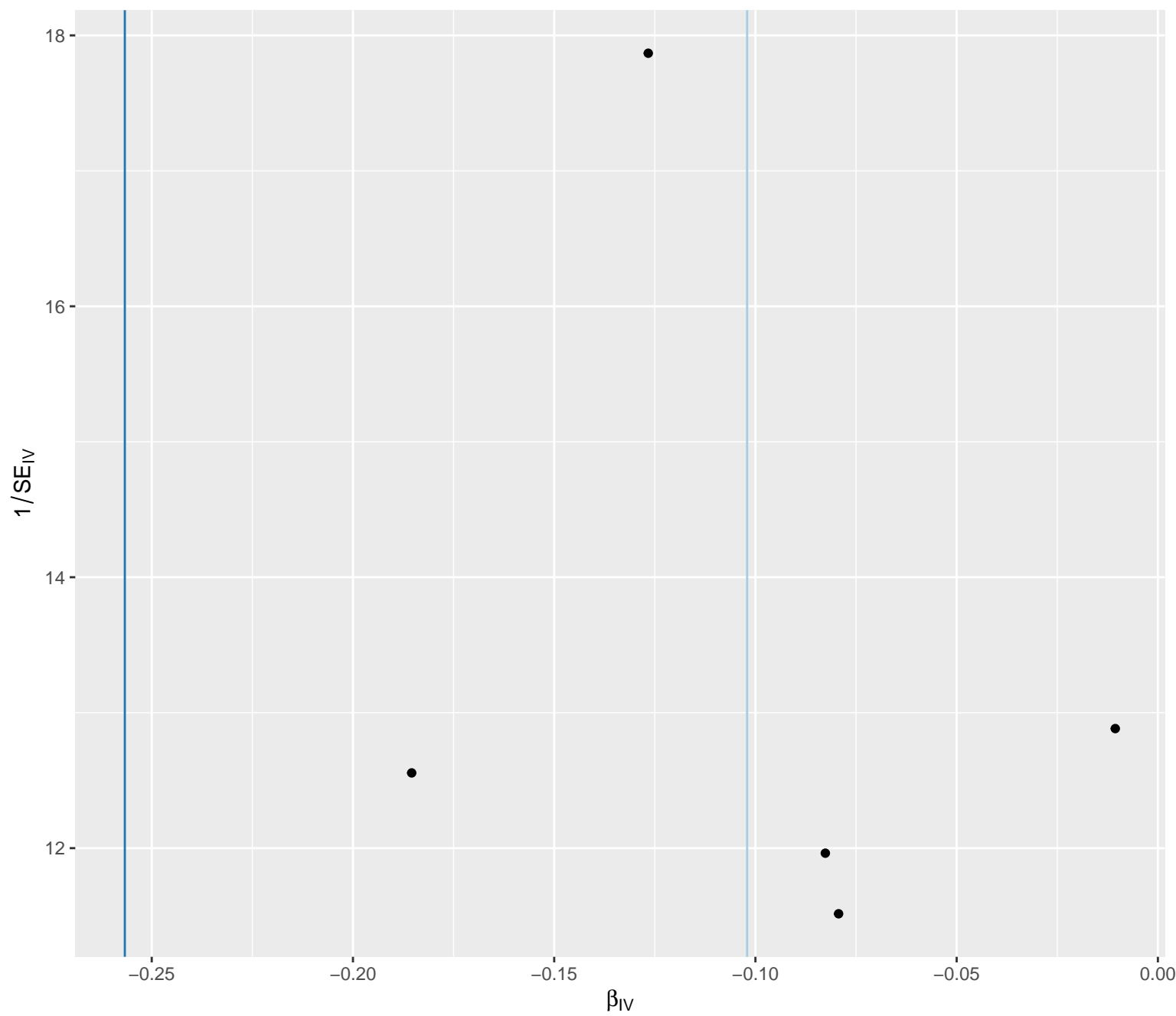

Supplement: Supplementary file 2 — Supplementary Material 2. [file 12944_2024_2103_MOESM2_ESM.zip › sFigure1∩╝êlipidomes-BC∩╝ë/GCST90277356/funnelplot.pdf]

# MR Test

- Inverse variance weighted
- MR Egger
- Simple mode
- Weighted median
- Weighted mode

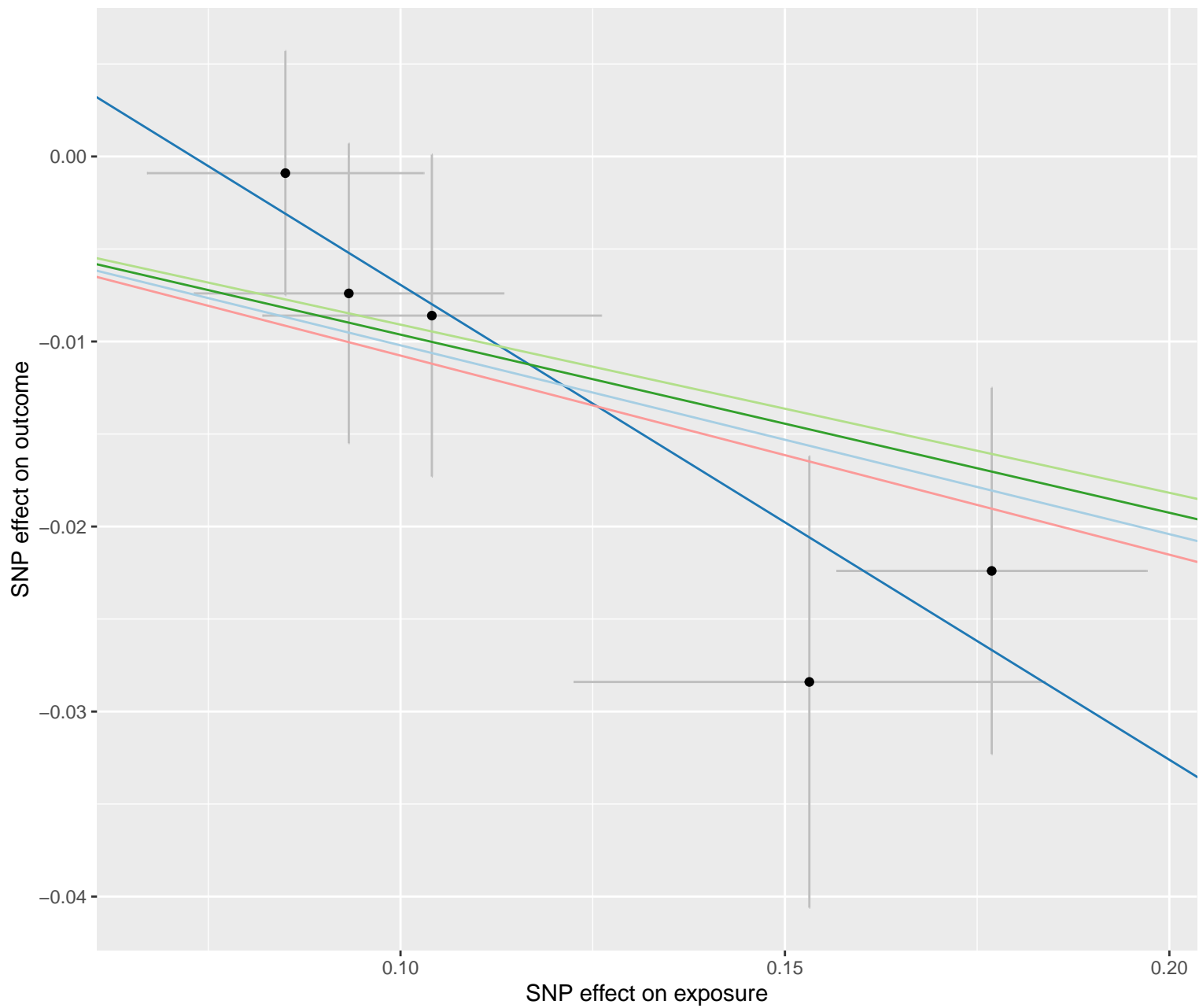

Supplement: Supplementary file 2 — Supplementary Material 2. [file 12944_2024_2103_MOESM2_ESM.zip › sFigure1∩╝êlipidomes-BC∩╝ë/GCST90277356/scatter.pdf]

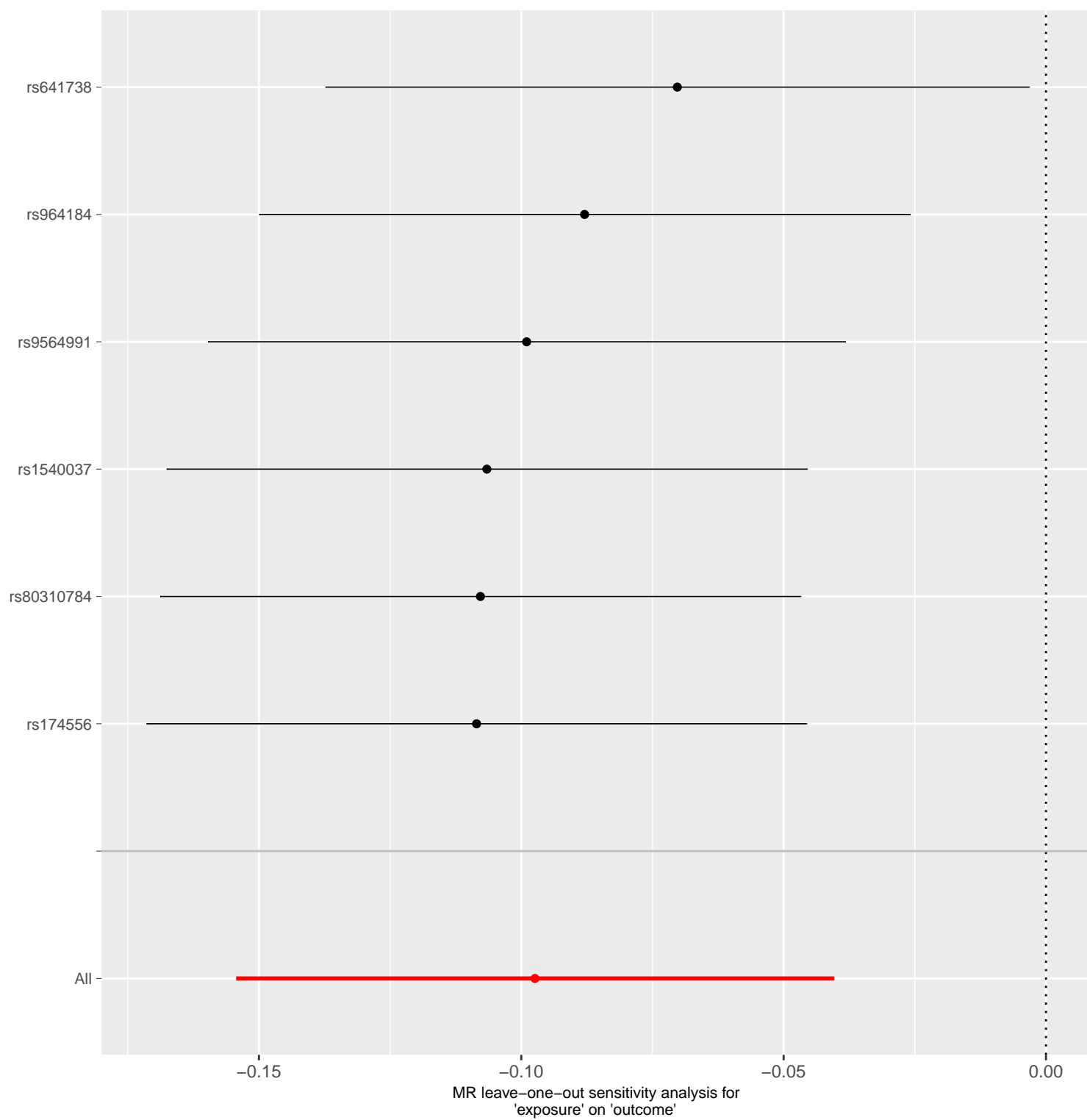

Supplement: Supplementary file 2 — Supplementary Material 2. [file 12944_2024_2103_MOESM2_ESM.zip › sFigure1∩╝êlipidomes-BC∩╝ë/GCST90277358/sensitivity-analysis.pdf]

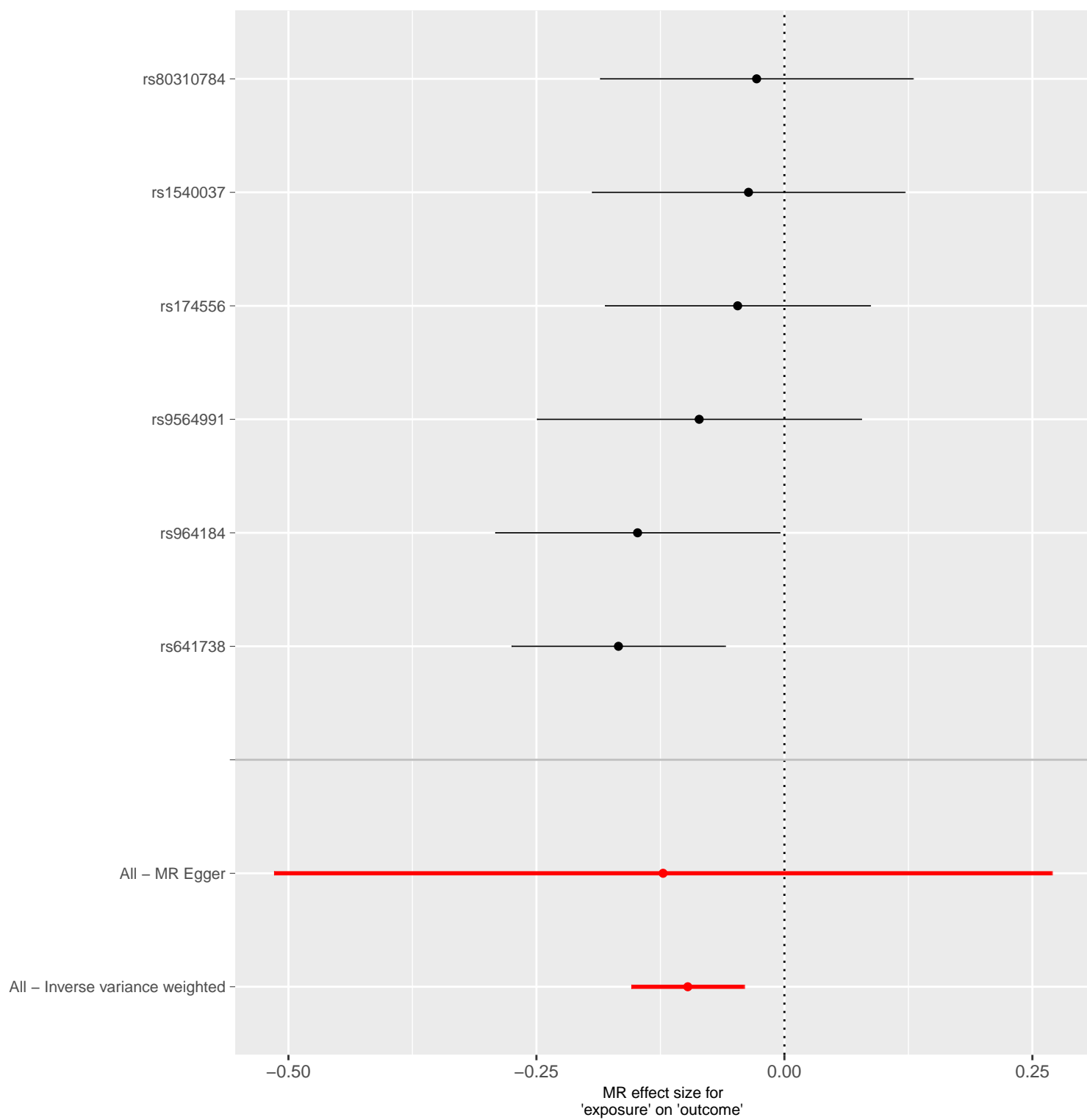

Supplement: Supplementary file 2 — Supplementary Material 2. [file 12944_2024_2103_MOESM2_ESM.zip › sFigure1∩╝êlipidomes-BC∩╝ë/GCST90277358/forest.pdf]

# MR Method

- Inverse variance weighted
- MR Egger

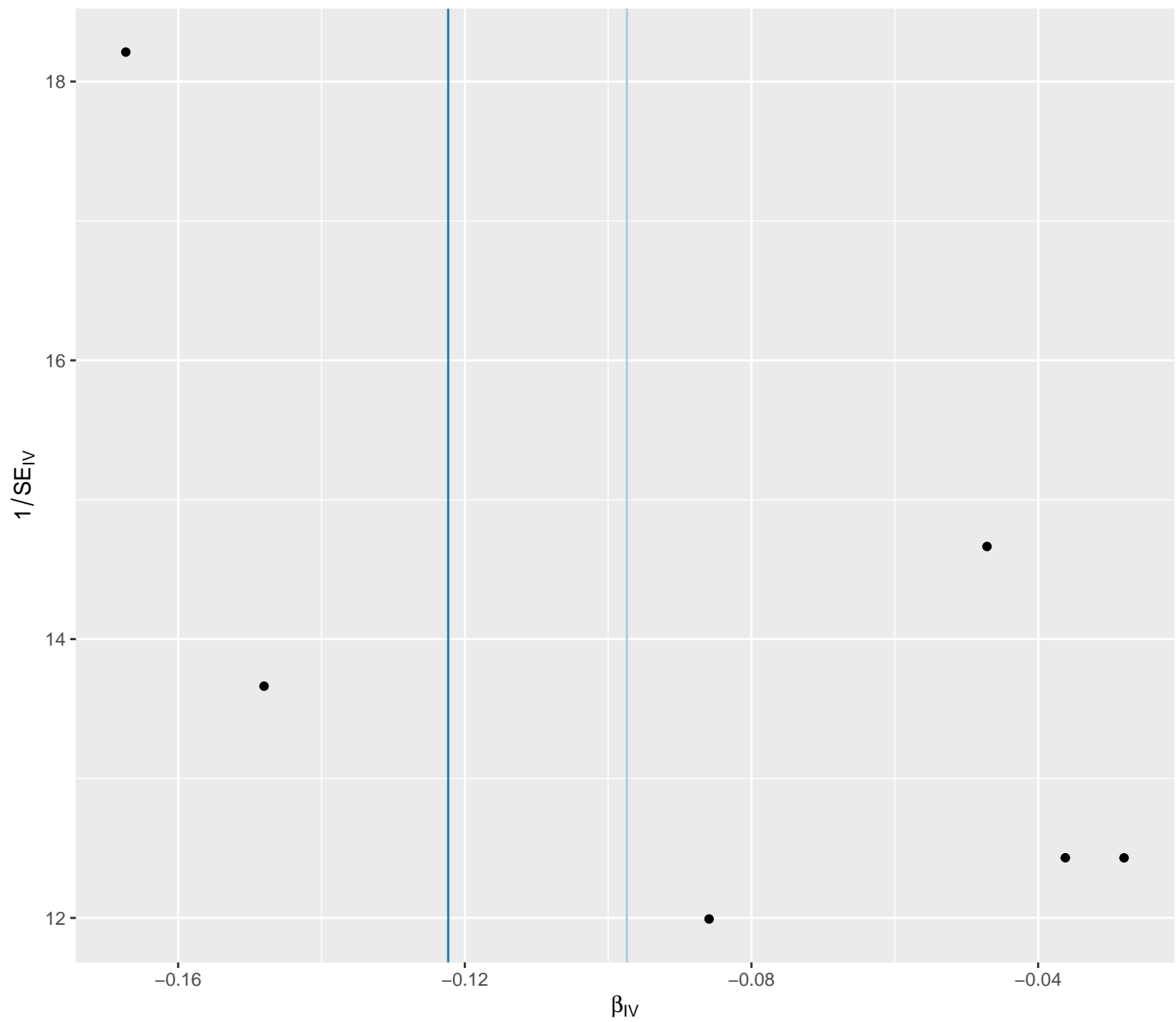

Supplement: Supplementary file 2 — Supplementary Material 2. [file 12944_2024_2103_MOESM2_ESM.zip › sFigure1∩╝êlipidomes-BC∩╝ë/GCST90277358/funnelplot.pdf]

# MR Test

- Inverse variance weighted
- MR Egger
- Simple mode
- Weighted median
- Weighted mode

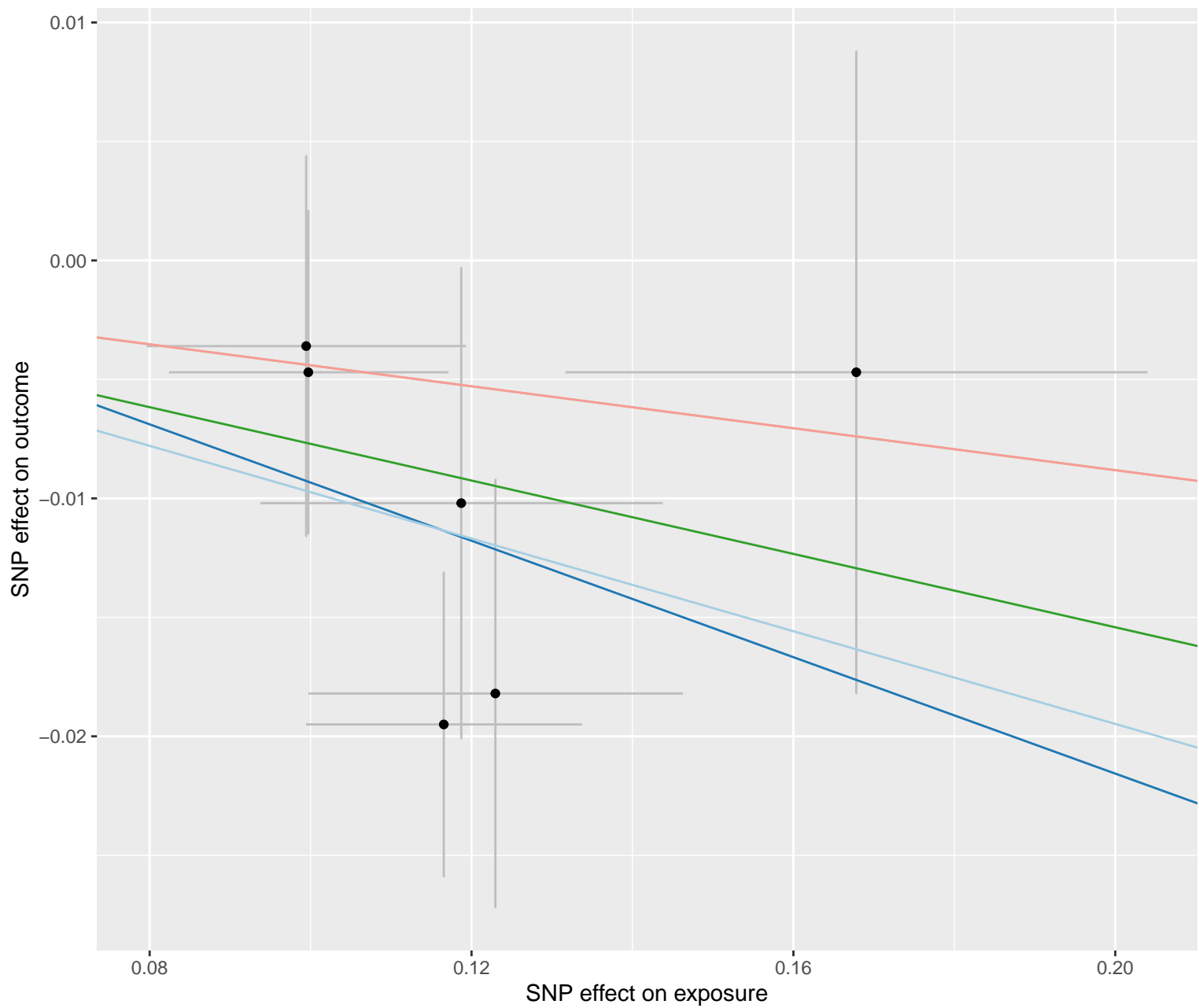

Supplement: Supplementary file 2 — Supplementary Material 2. [file 12944_2024_2103_MOESM2_ESM.zip › sFigure1∩╝êlipidomes-BC∩╝ë/GCST90277358/scatter.pdf]

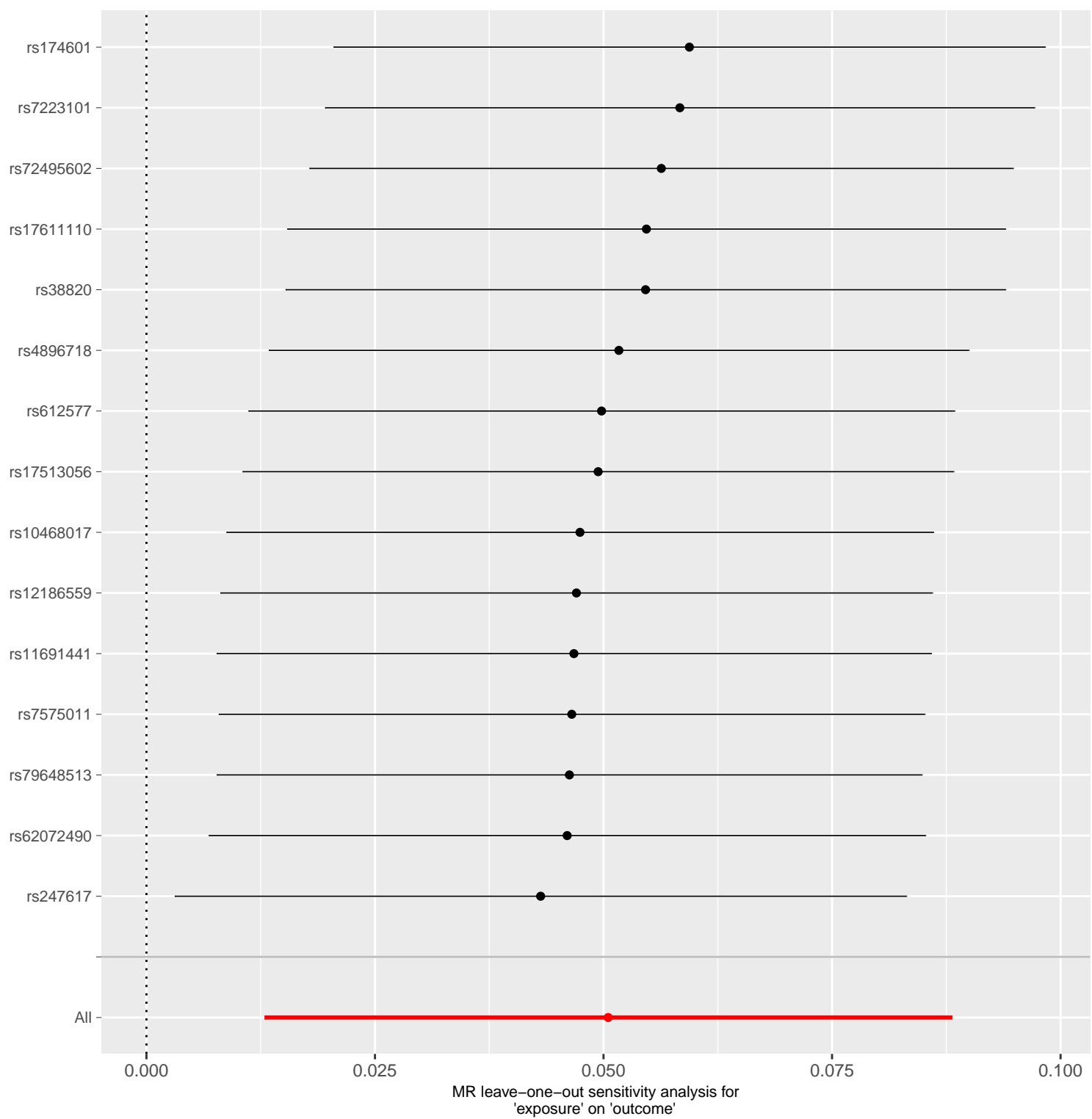

Supplement: Supplementary file 2 — Supplementary Material 2. [file 12944_2024_2103_MOESM2_ESM.zip › sFigure1∩╝êlipidomes-BC∩╝ë/GCST90277333/sensitivity-analysis.pdf]

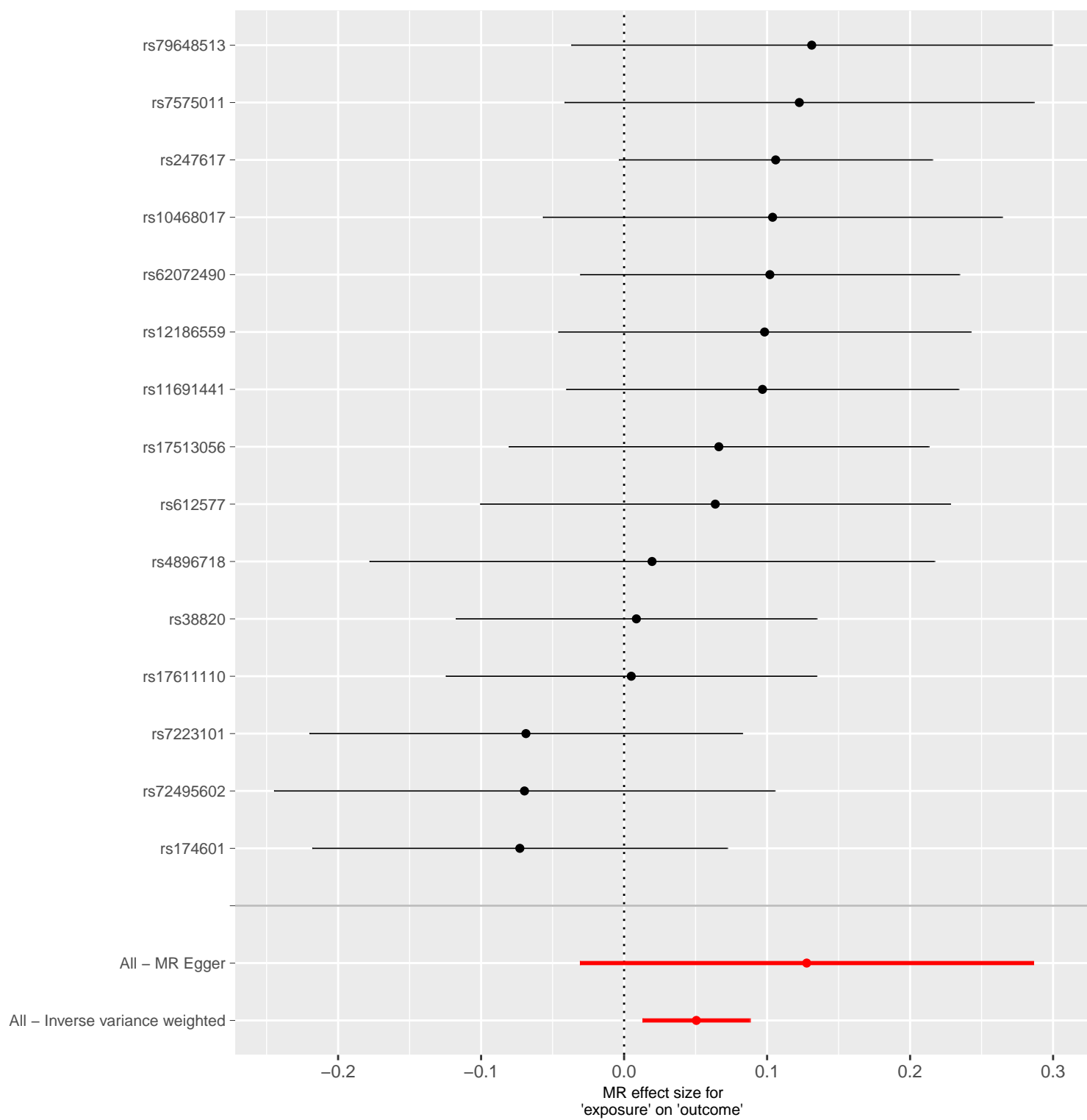

Supplement: Supplementary file 2 — Supplementary Material 2. [file 12944_2024_2103_MOESM2_ESM.zip › sFigure1∩╝êlipidomes-BC∩╝ë/GCST90277333/forest.pdf]

# MR Method

- Inverse variance weighted
- MR Egger

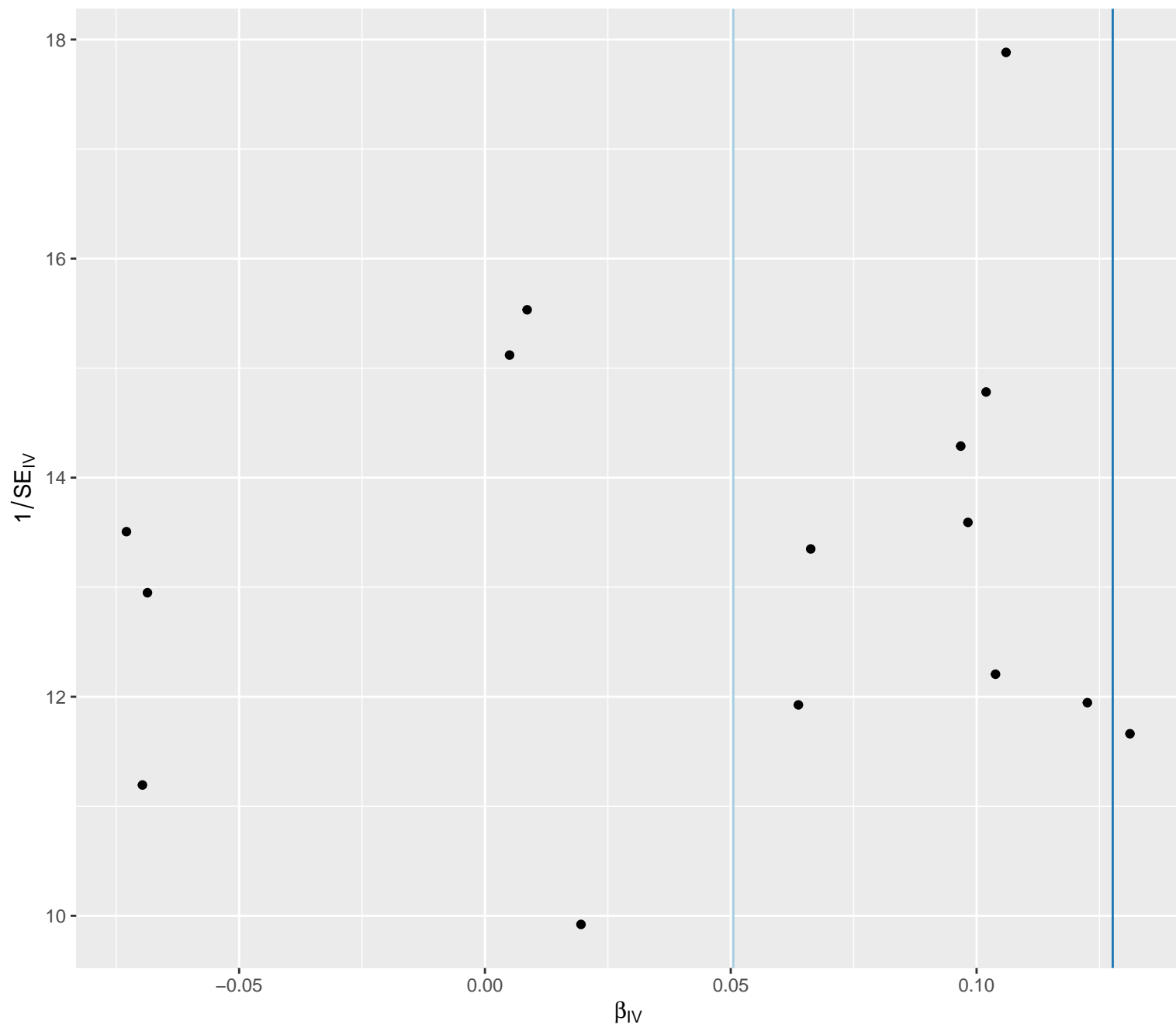

Supplement: Supplementary file 2 — Supplementary Material 2. [file 12944_2024_2103_MOESM2_ESM.zip › sFigure1∩╝êlipidomes-BC∩╝ë/GCST90277333/funnelplot.pdf]

# MR Test

- Inverse variance weighted
- MR Egger
- Simple mode
- Weighted median
- Weighted mode

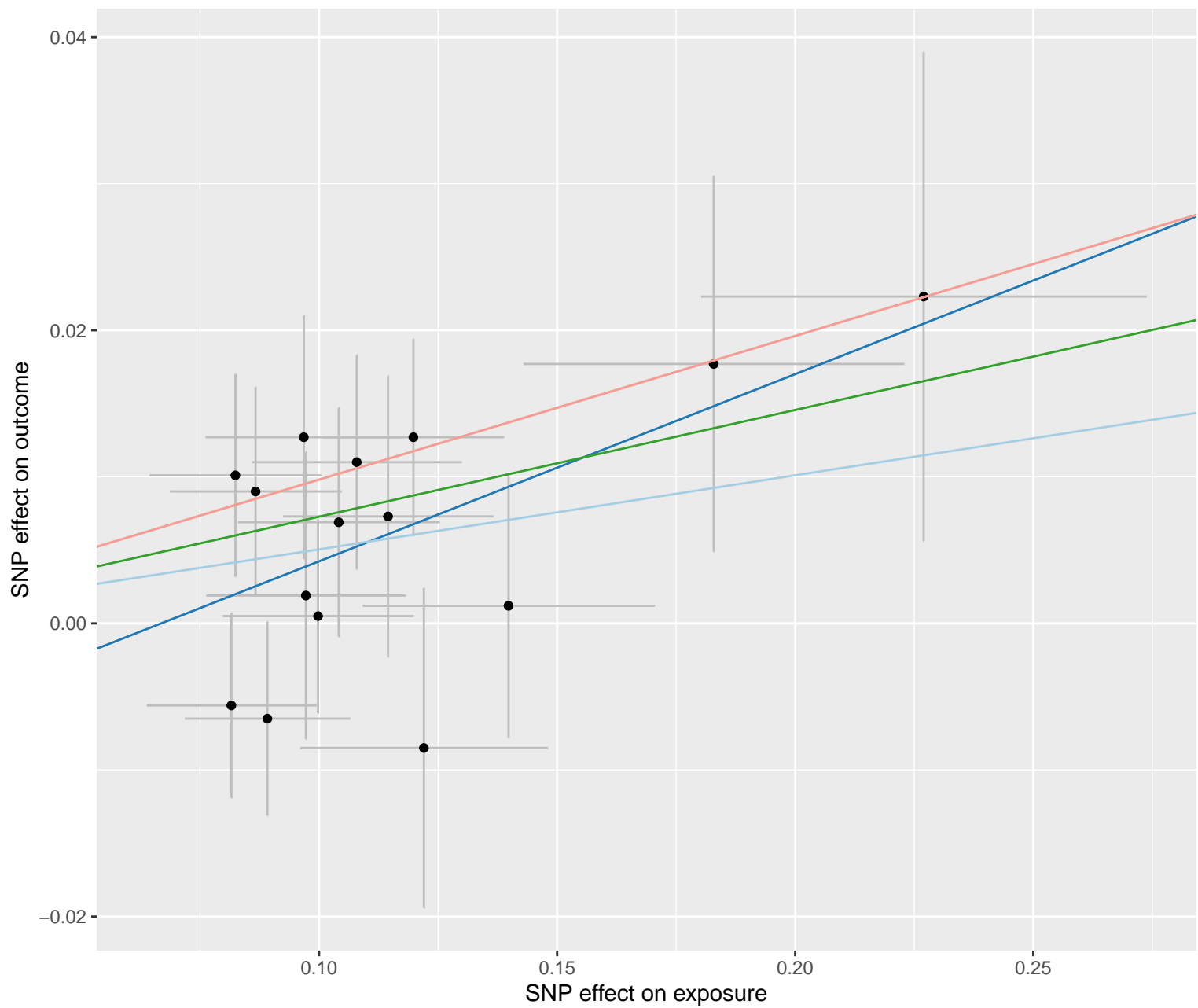

Supplement: Supplementary file 2 — Supplementary Material 2. [file 12944_2024_2103_MOESM2_ESM.zip › sFigure1∩╝êlipidomes-BC∩╝ë/GCST90277333/scatter.pdf]

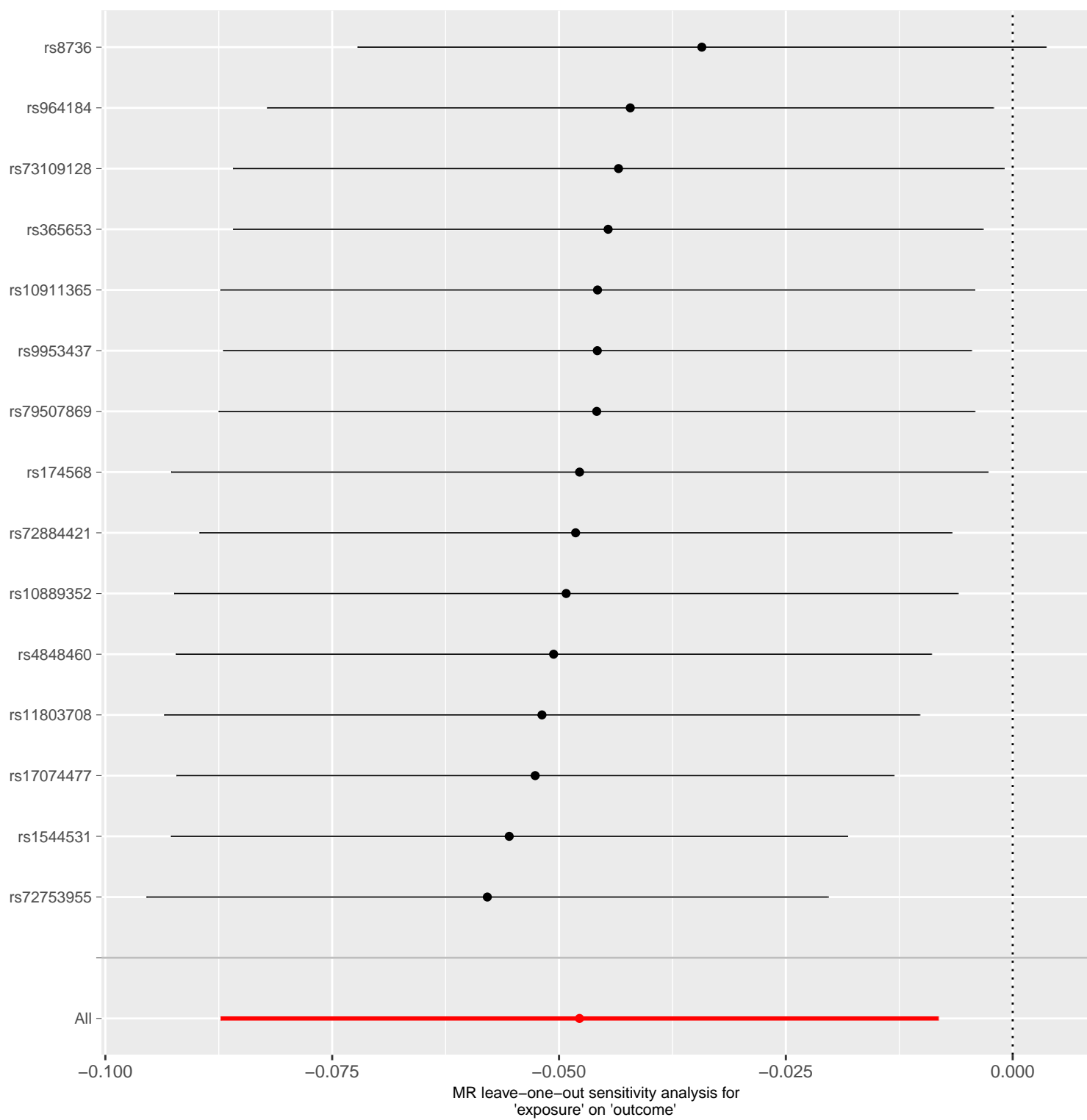

Supplement: Supplementary file 2 — Supplementary Material 2. [file 12944_2024_2103_MOESM2_ESM.zip › sFigure1∩╝êlipidomes-BC∩╝ë/GCST90277366/sensitivity-analysis.pdf]

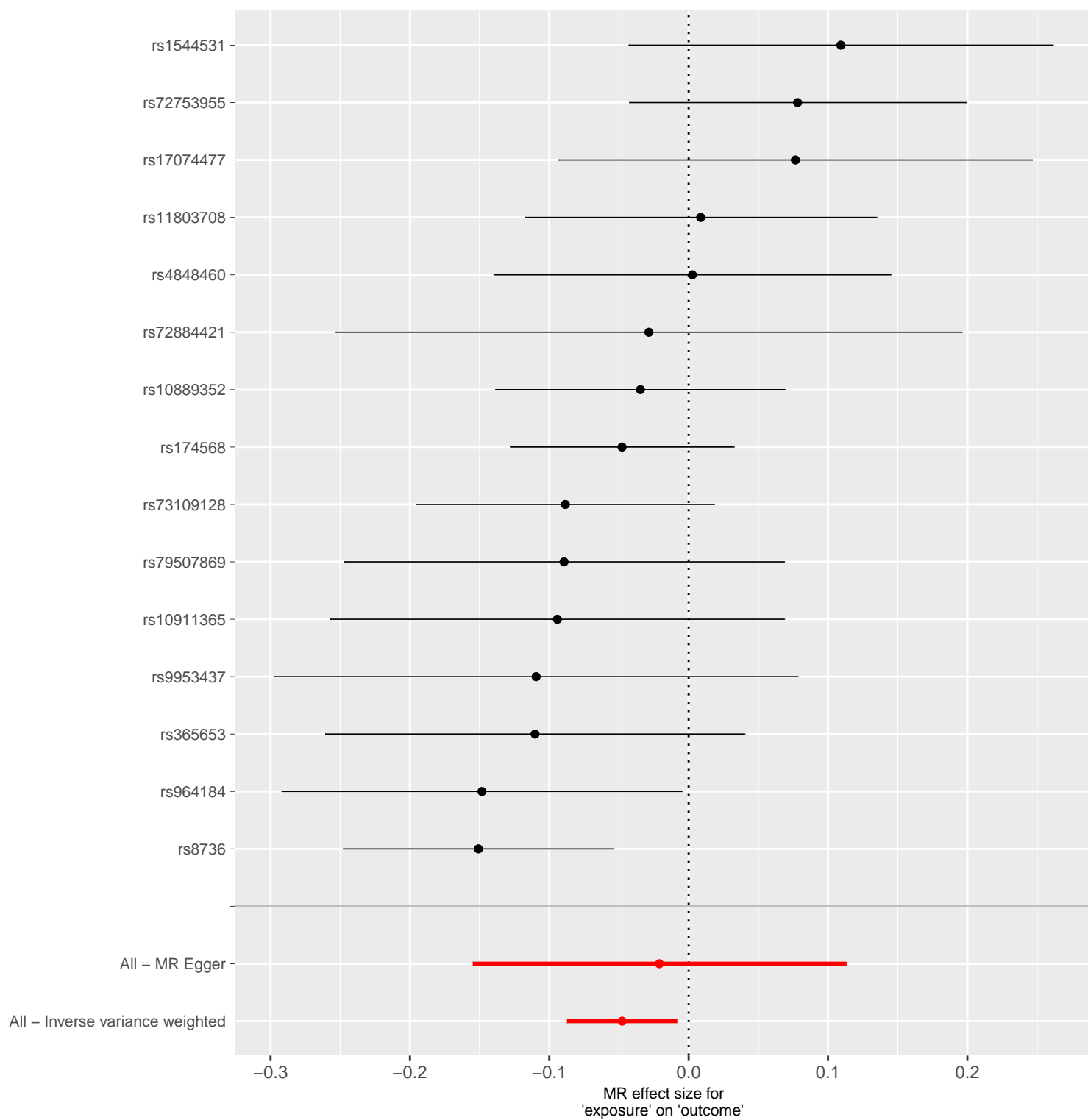

Supplement: Supplementary file 2 — Supplementary Material 2. [file 12944_2024_2103_MOESM2_ESM.zip › sFigure1∩╝êlipidomes-BC∩╝ë/GCST90277366/forest.pdf]

# MR Method

- Inverse variance weighted
- MR Egger

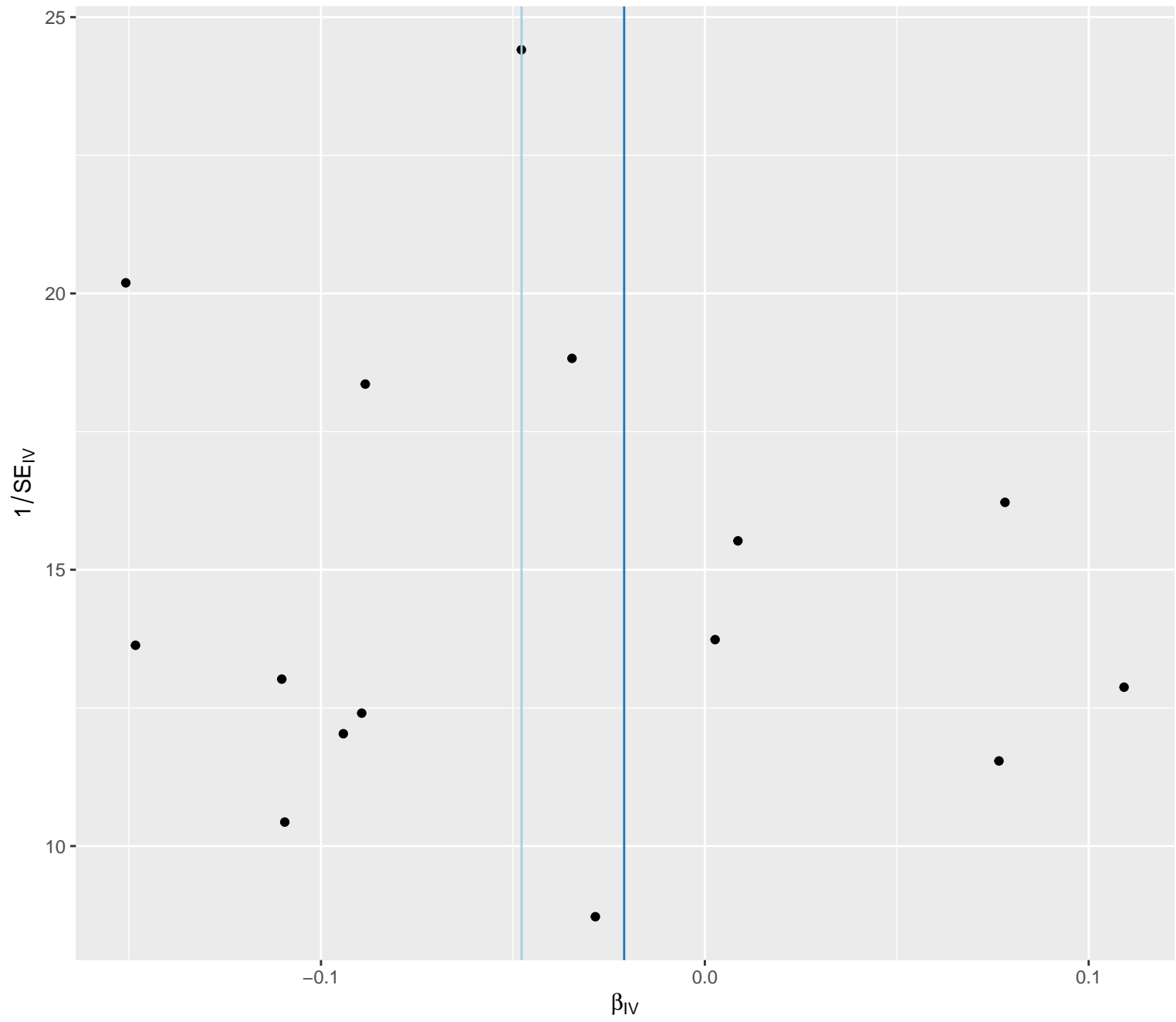

Supplement: Supplementary file 2 — Supplementary Material 2. [file 12944_2024_2103_MOESM2_ESM.zip › sFigure1∩╝êlipidomes-BC∩╝ë/GCST90277366/funnelplot.pdf]

# MR Test

- Inverse variance weighted
- MR Egger
- Simple mode
- Weighted median
- Weighted mode

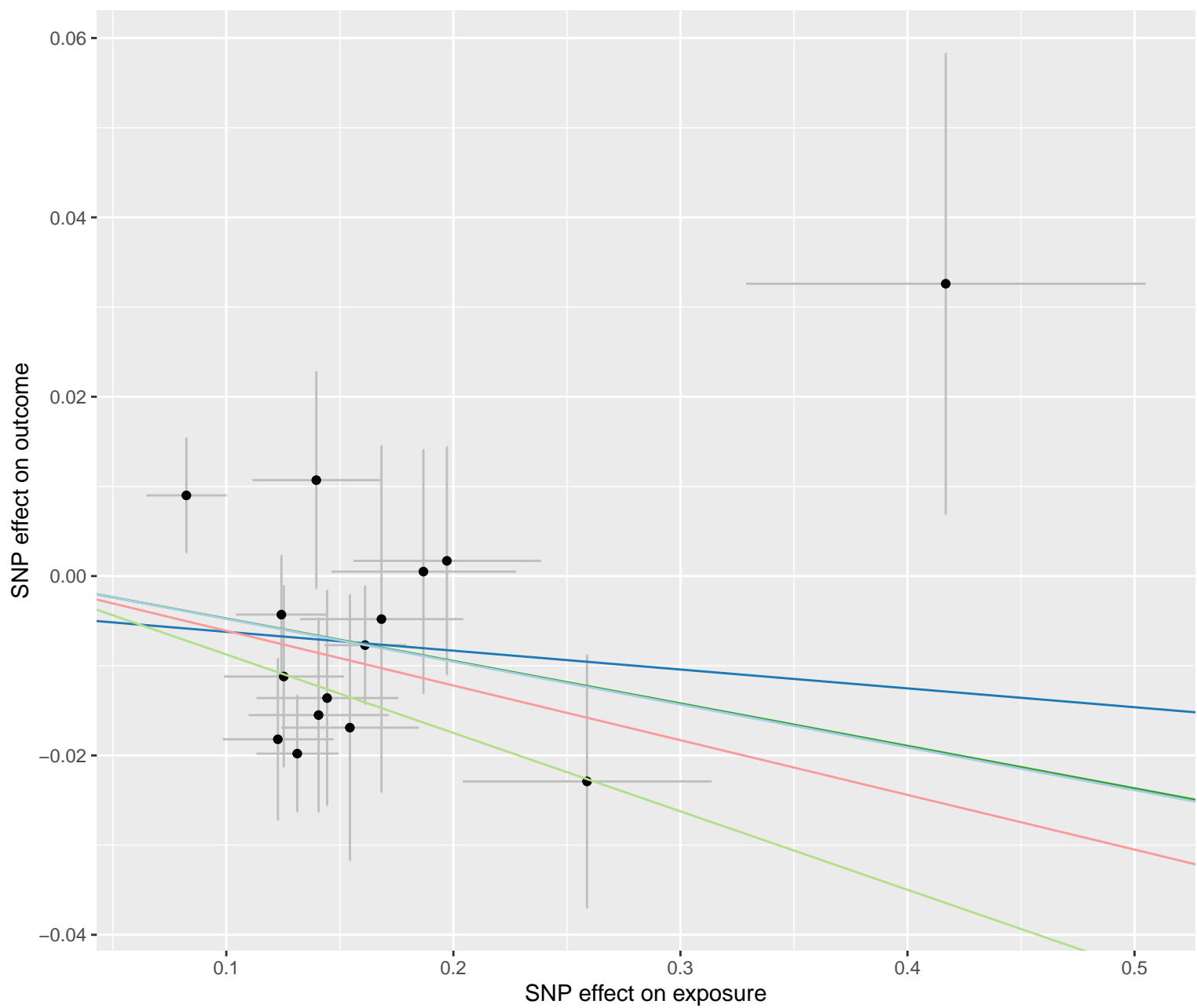

Supplement: Supplementary file 2 — Supplementary Material 2. [file 12944_2024_2103_MOESM2_ESM.zip › sFigure1∩╝êlipidomes-BC∩╝ë/GCST90277366/scatter.pdf]

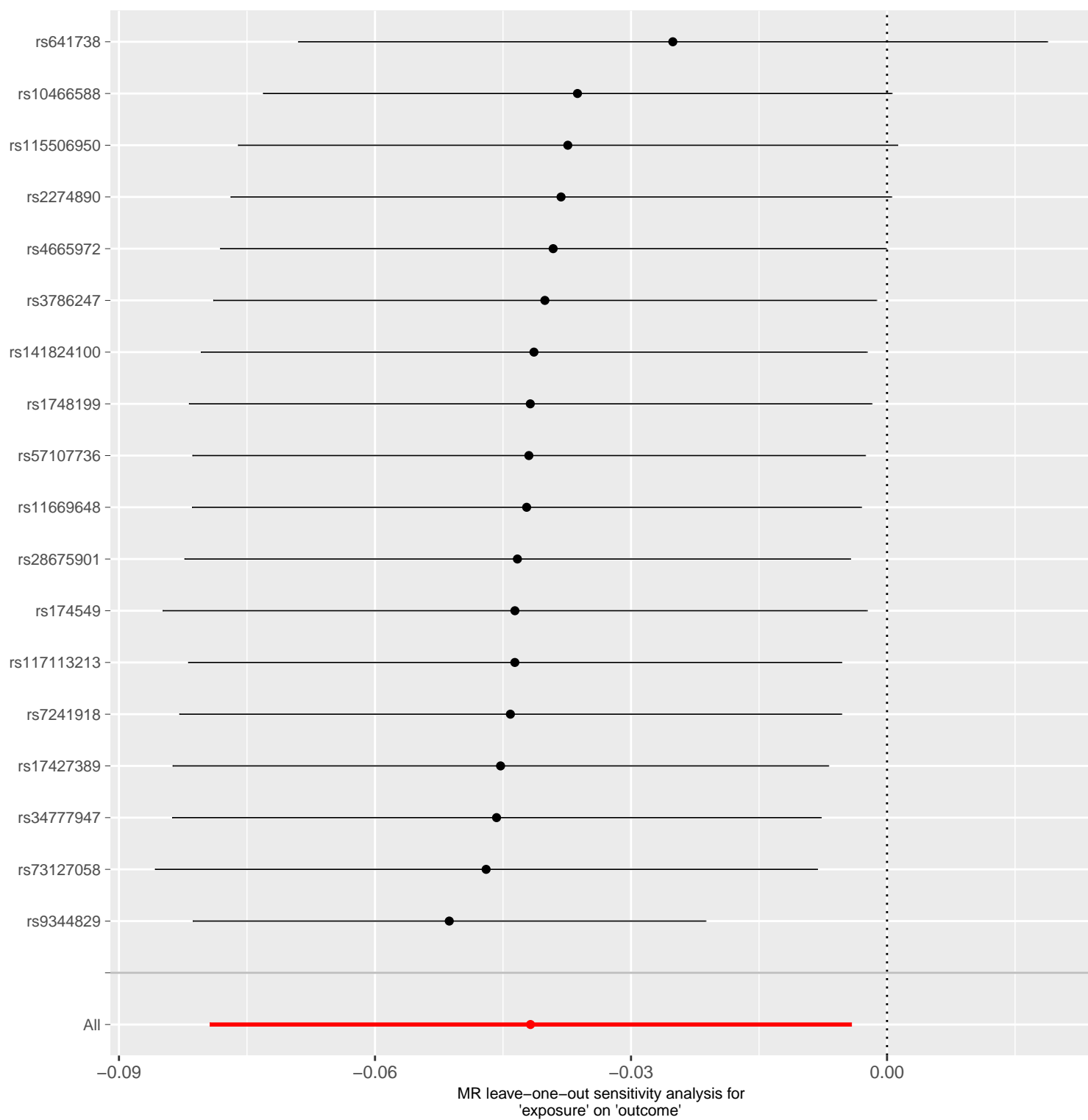

Supplement: Supplementary file 2 — Supplementary Material 2. [file 12944_2024_2103_MOESM2_ESM.zip › sFigure1∩╝êlipidomes-BC∩╝ë/GCST90277359/sensitivity-analysis.pdf]

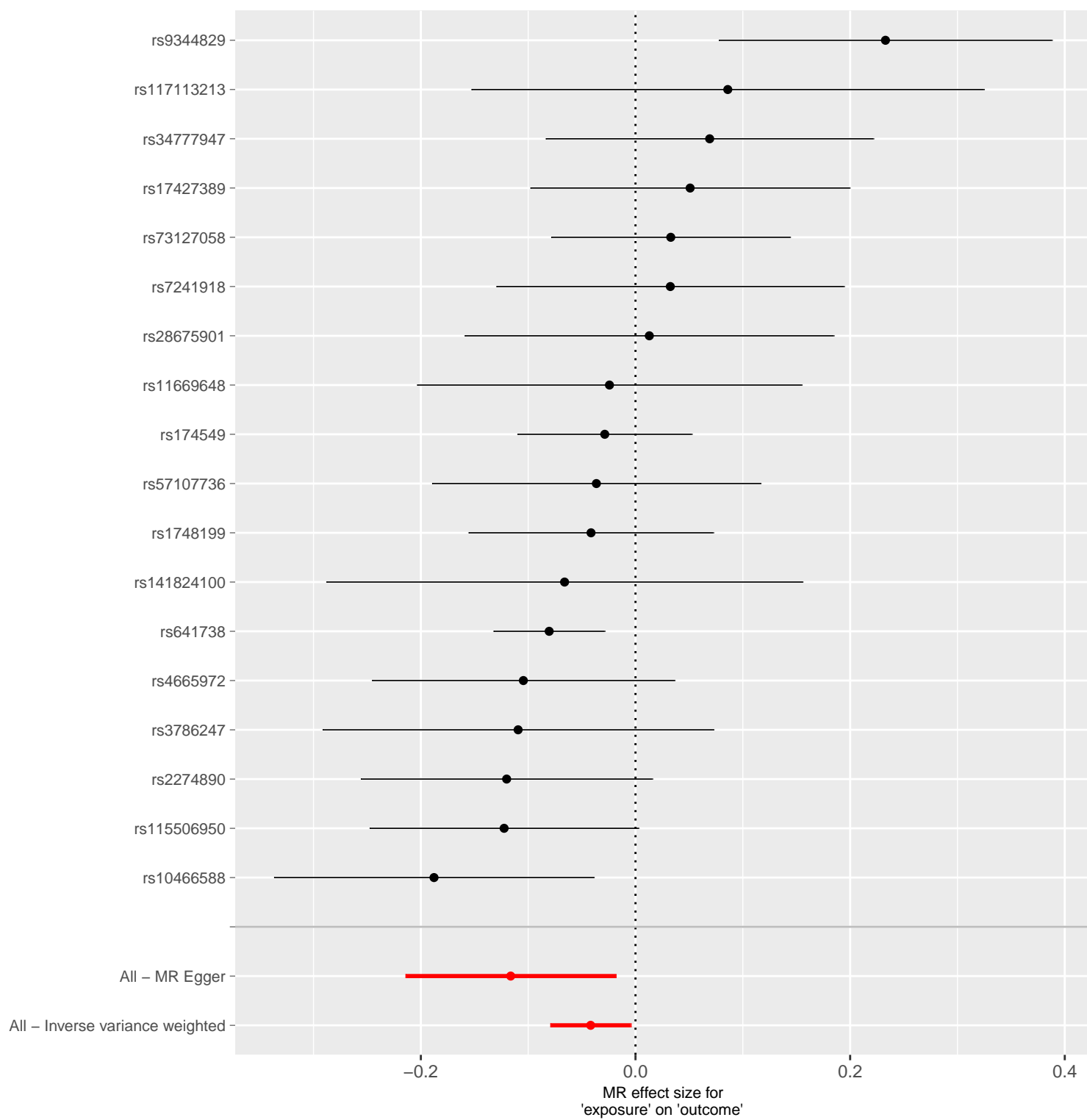

Supplement: Supplementary file 2 — Supplementary Material 2. [file 12944_2024_2103_MOESM2_ESM.zip › sFigure1∩╝êlipidomes-BC∩╝ë/GCST90277359/forest.pdf]

# MR Method

- Inverse variance weighted
- MR Egger

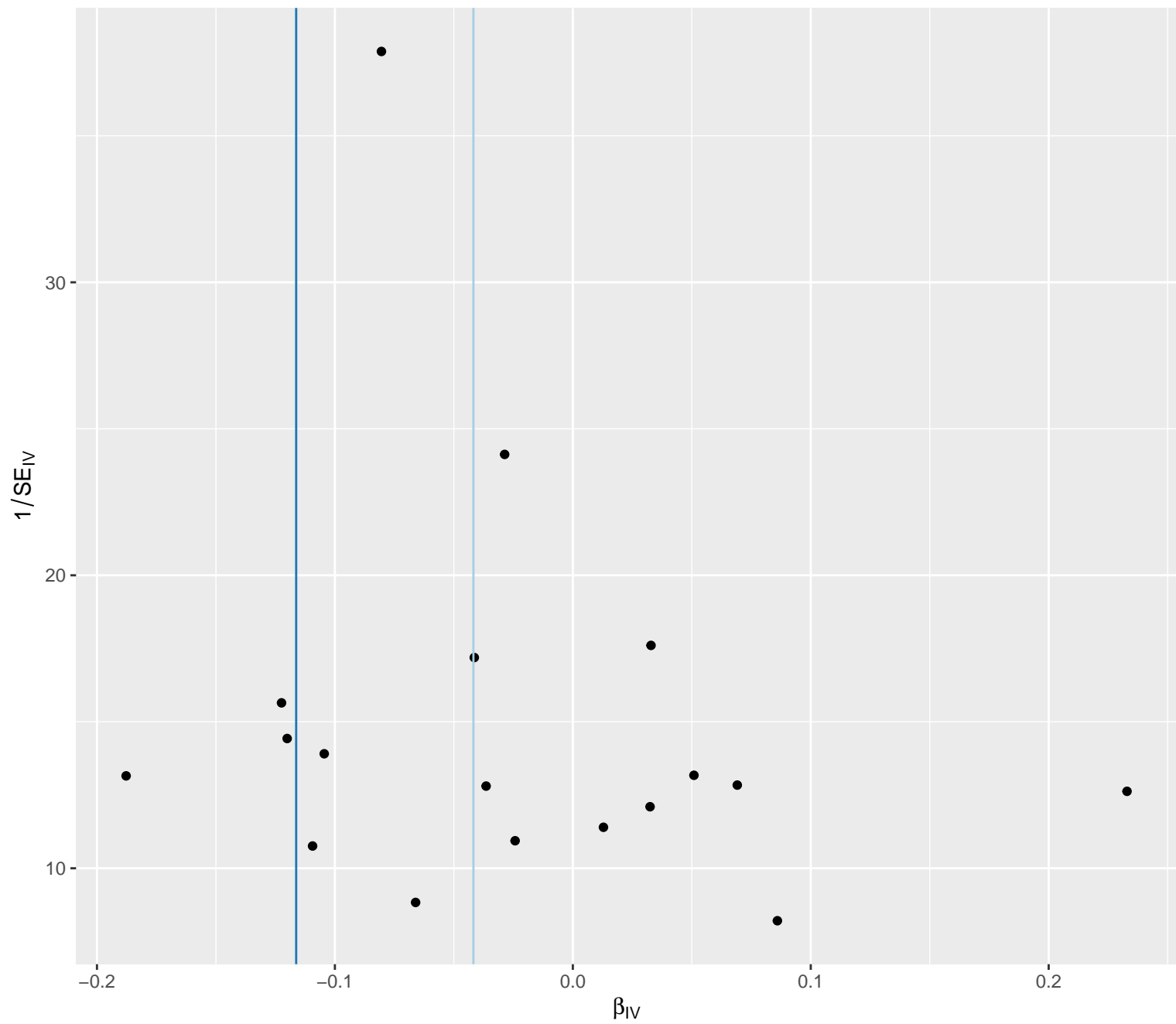

Supplement: Supplementary file 2 — Supplementary Material 2. [file 12944_2024_2103_MOESM2_ESM.zip › sFigure1∩╝êlipidomes-BC∩╝ë/GCST90277359/funnelplot.pdf]

# MR Test

- Inverse variance weighted
- MR Egger
- Simple mode
- Weighted median
- Weighted mode

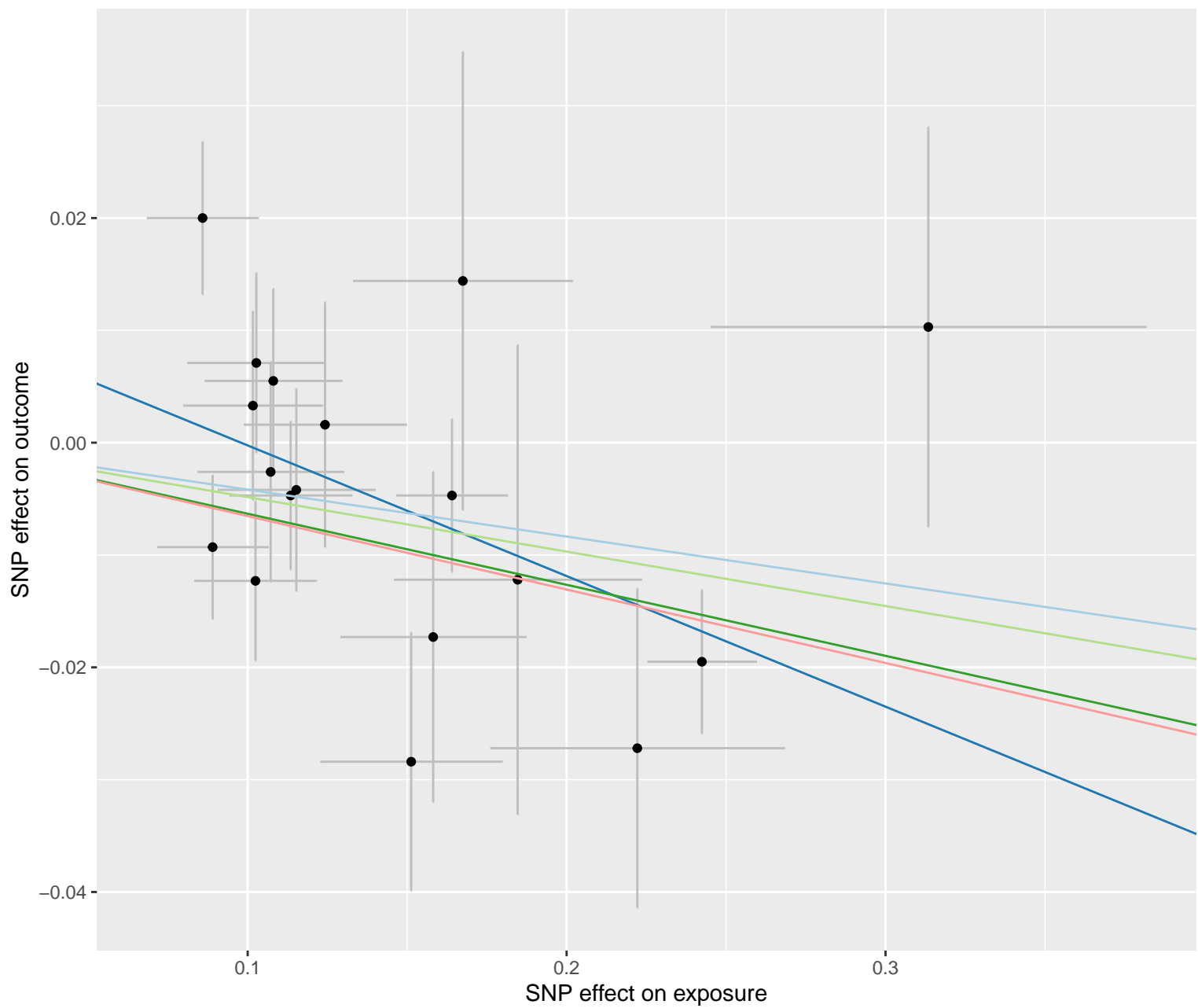

Supplement: Supplementary file 2 — Supplementary Material 2. [file 12944_2024_2103_MOESM2_ESM.zip › sFigure1∩╝êlipidomes-BC∩╝ë/GCST90277359/scatter.pdf]

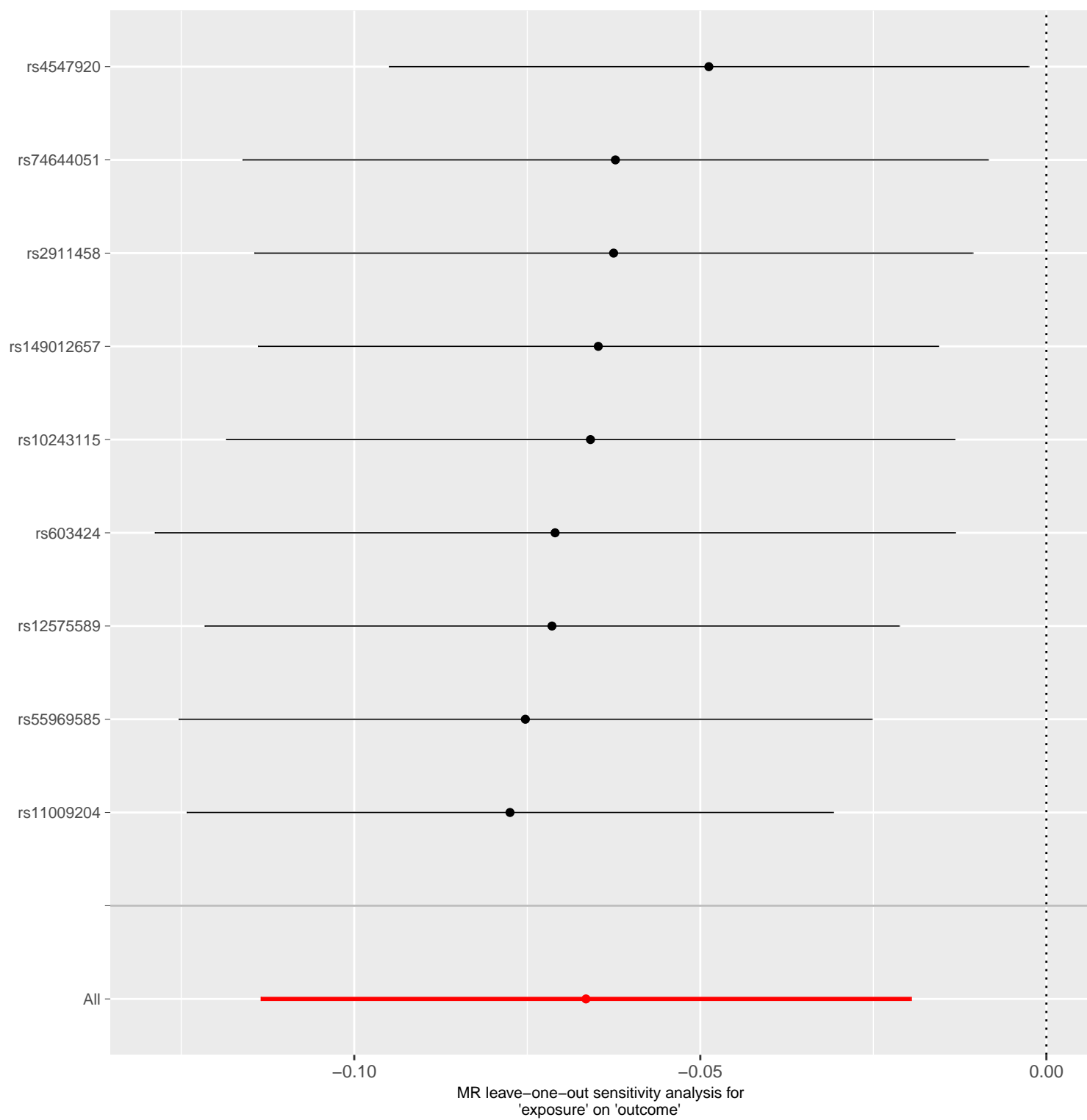

Supplement: Supplementary file 2 — Supplementary Material 2. [file 12944_2024_2103_MOESM2_ESM.zip › sFigure1∩╝êlipidomes-BC∩╝ë/GCST90277335/sensitivity-analysis.pdf]

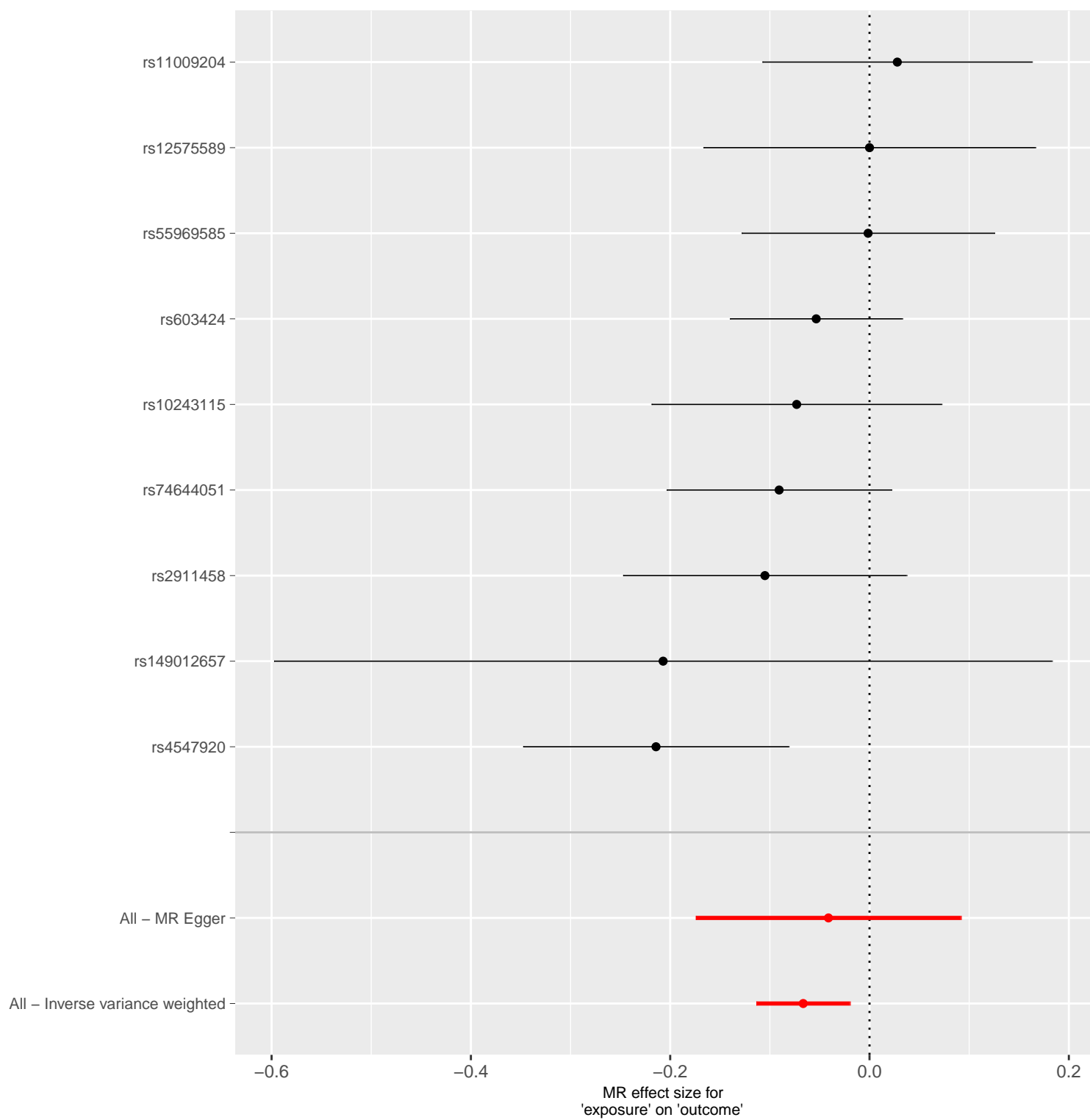

Supplement: Supplementary file 2 — Supplementary Material 2. [file 12944_2024_2103_MOESM2_ESM.zip › sFigure1∩╝êlipidomes-BC∩╝ë/GCST90277335/forest.pdf]

# MR Method

- Inverse variance weighted
- MR Egger

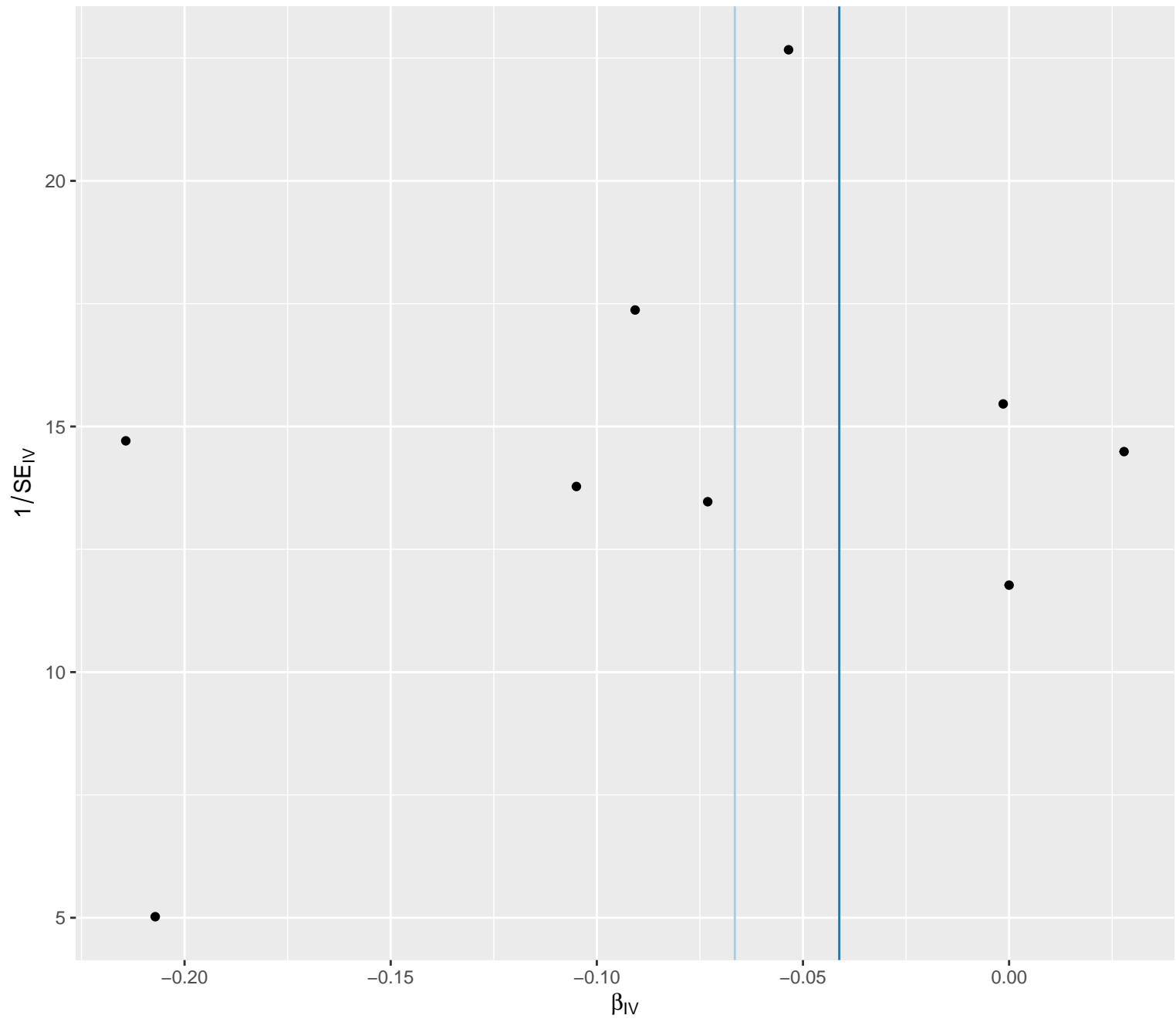

Supplement: Supplementary file 2 — Supplementary Material 2. [file 12944_2024_2103_MOESM2_ESM.zip › sFigure1∩╝êlipidomes-BC∩╝ë/GCST90277335/funnelplot.pdf]

# MR Test

- Inverse variance weighted
- MR Egger
- Simple mode
- Weighted median
- Weighted mode

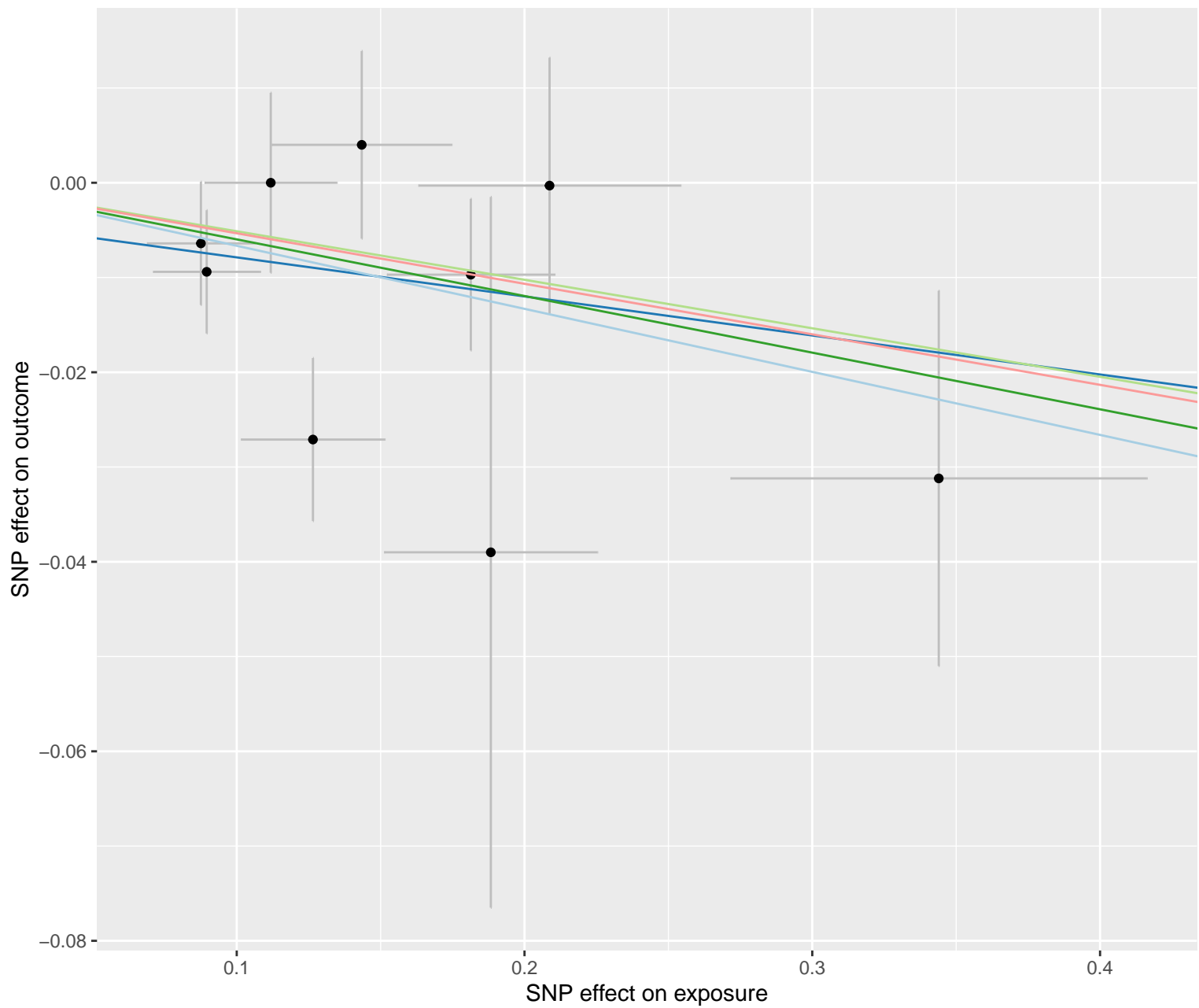

Supplement: Supplementary file 2 — Supplementary Material 2. [file 12944_2024_2103_MOESM2_ESM.zip › sFigure1∩╝êlipidomes-BC∩╝ë/GCST90277335/scatter.pdf]

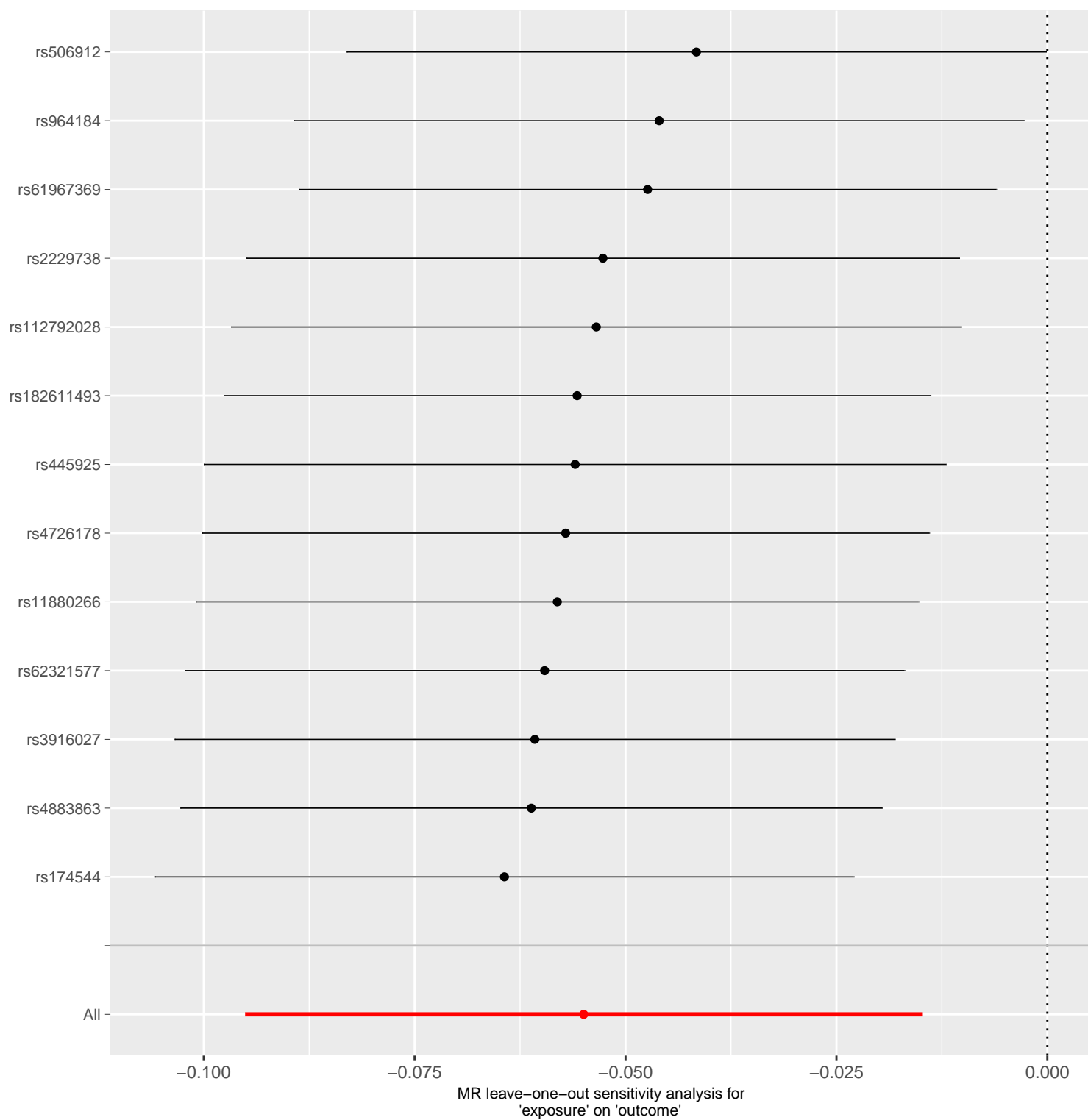

Supplement: Supplementary file 2 — Supplementary Material 2. [file 12944_2024_2103_MOESM2_ESM.zip › sFigure1∩╝êlipidomes-BC∩╝ë/GCST90277416/sensitivity-analysis.pdf]

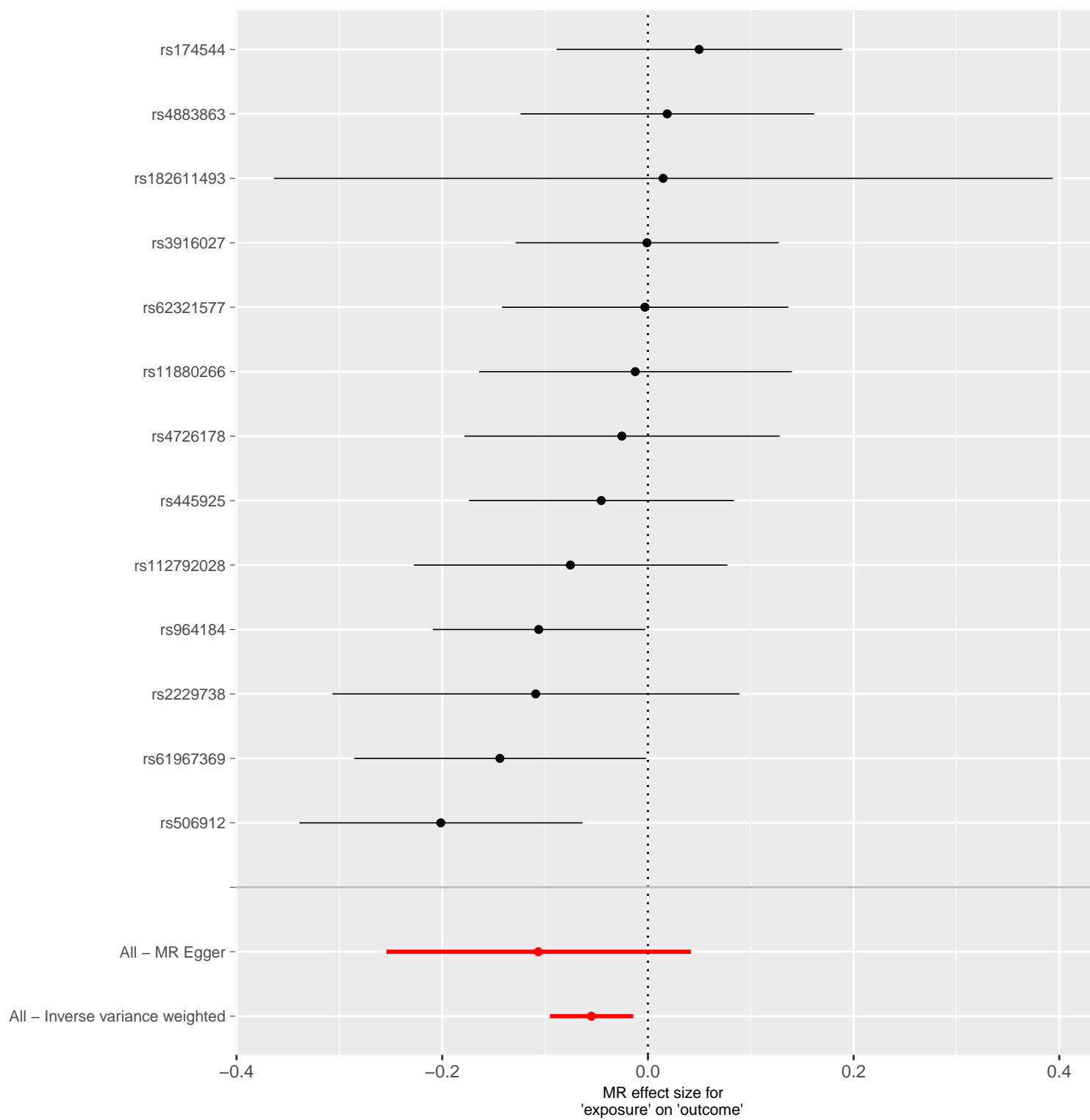

Supplement: Supplementary file 2 — Supplementary Material 2. [file 12944_2024_2103_MOESM2_ESM.zip › sFigure1∩╝êlipidomes-BC∩╝ë/GCST90277416/forest.pdf]

# MR Method

- Inverse variance weighted
- MR Egger

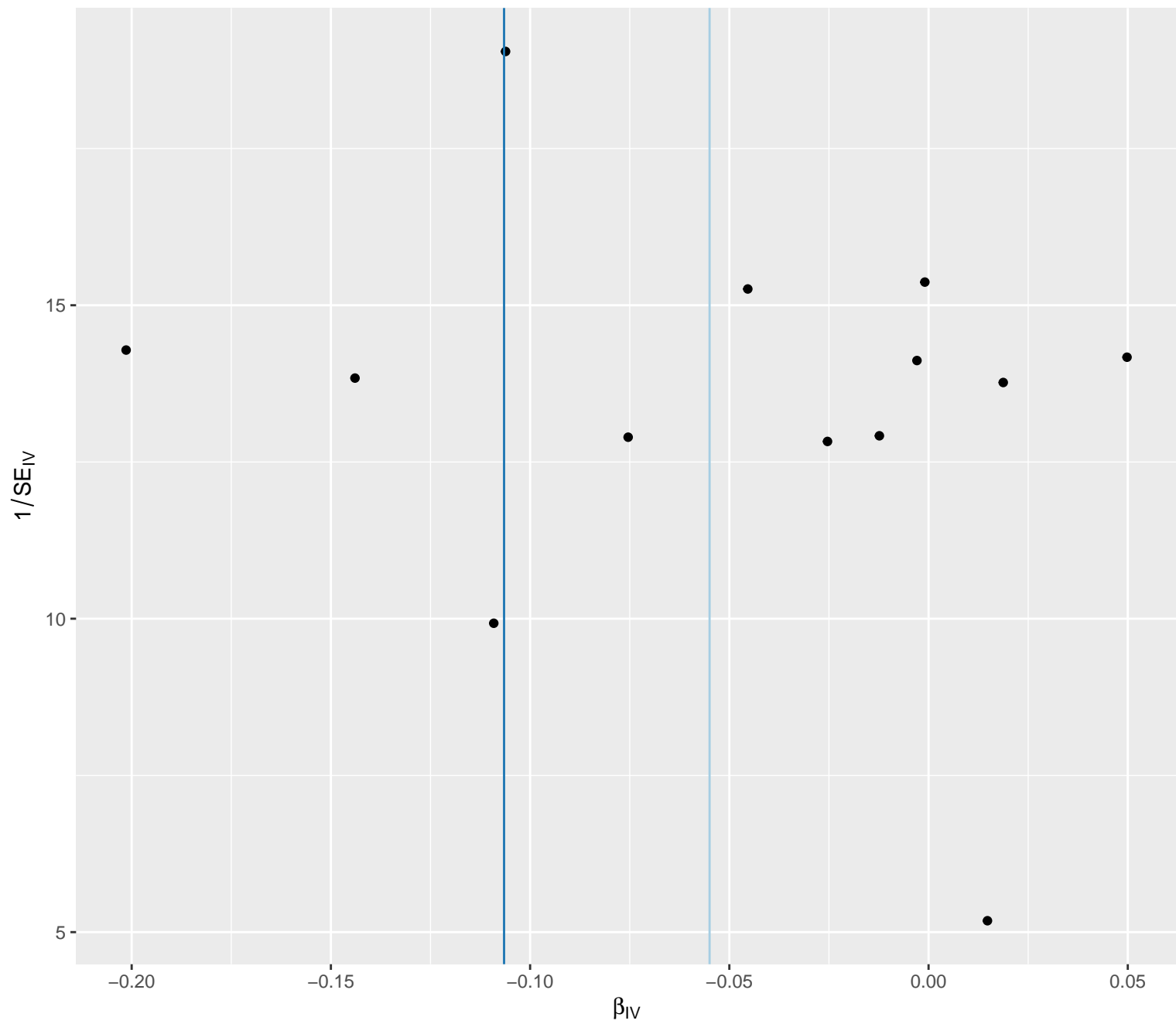

Supplement: Supplementary file 2 — Supplementary Material 2. [file 12944_2024_2103_MOESM2_ESM.zip › sFigure1∩╝êlipidomes-BC∩╝ë/GCST90277416/funnelplot.pdf]

# MR Test

- Inverse variance weighted
- MR Egger
- Simple mode
- Weighted median
- Weighted mode

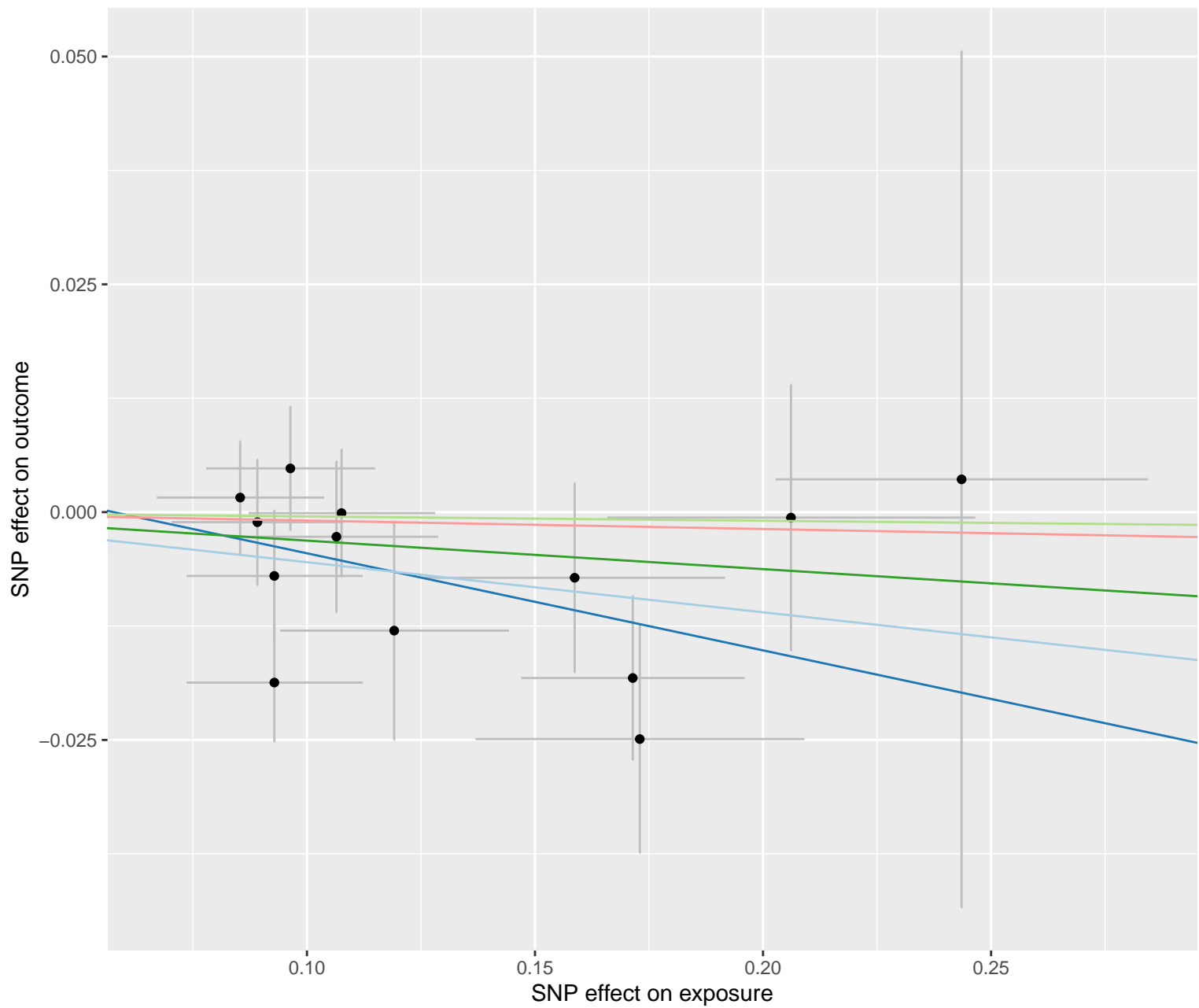

Supplement: Supplementary file 2 — Supplementary Material 2. [file 12944_2024_2103_MOESM2_ESM.zip › sFigure1∩╝êlipidomes-BC∩╝ë/GCST90277416/scatter.pdf]

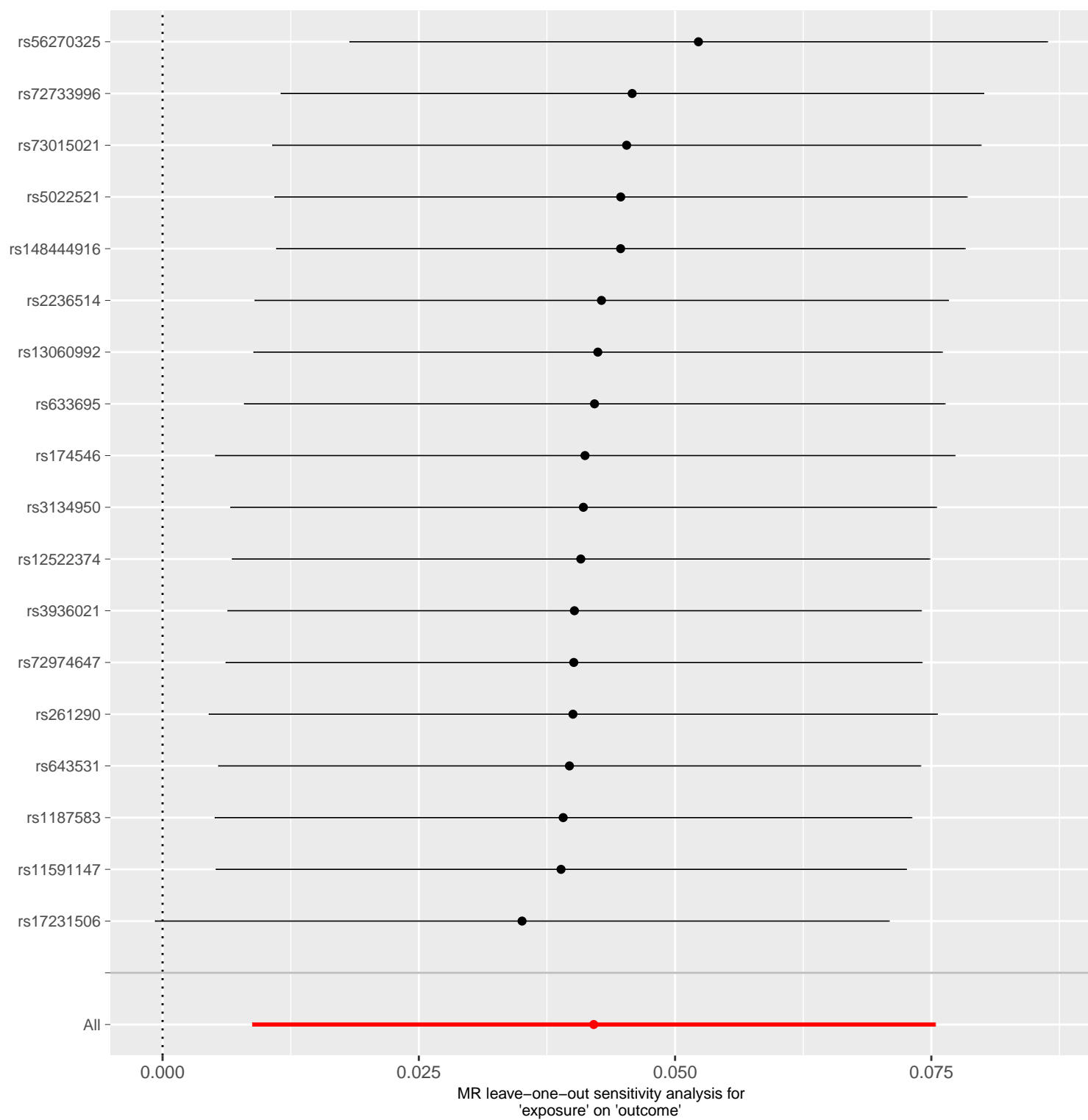

Supplement: Supplementary file 2 — Supplementary Material 2. [file 12944_2024_2103_MOESM2_ESM.zip › sFigure1∩╝êlipidomes-BC∩╝ë/GCST90277277/sensitivity-analysis.pdf]

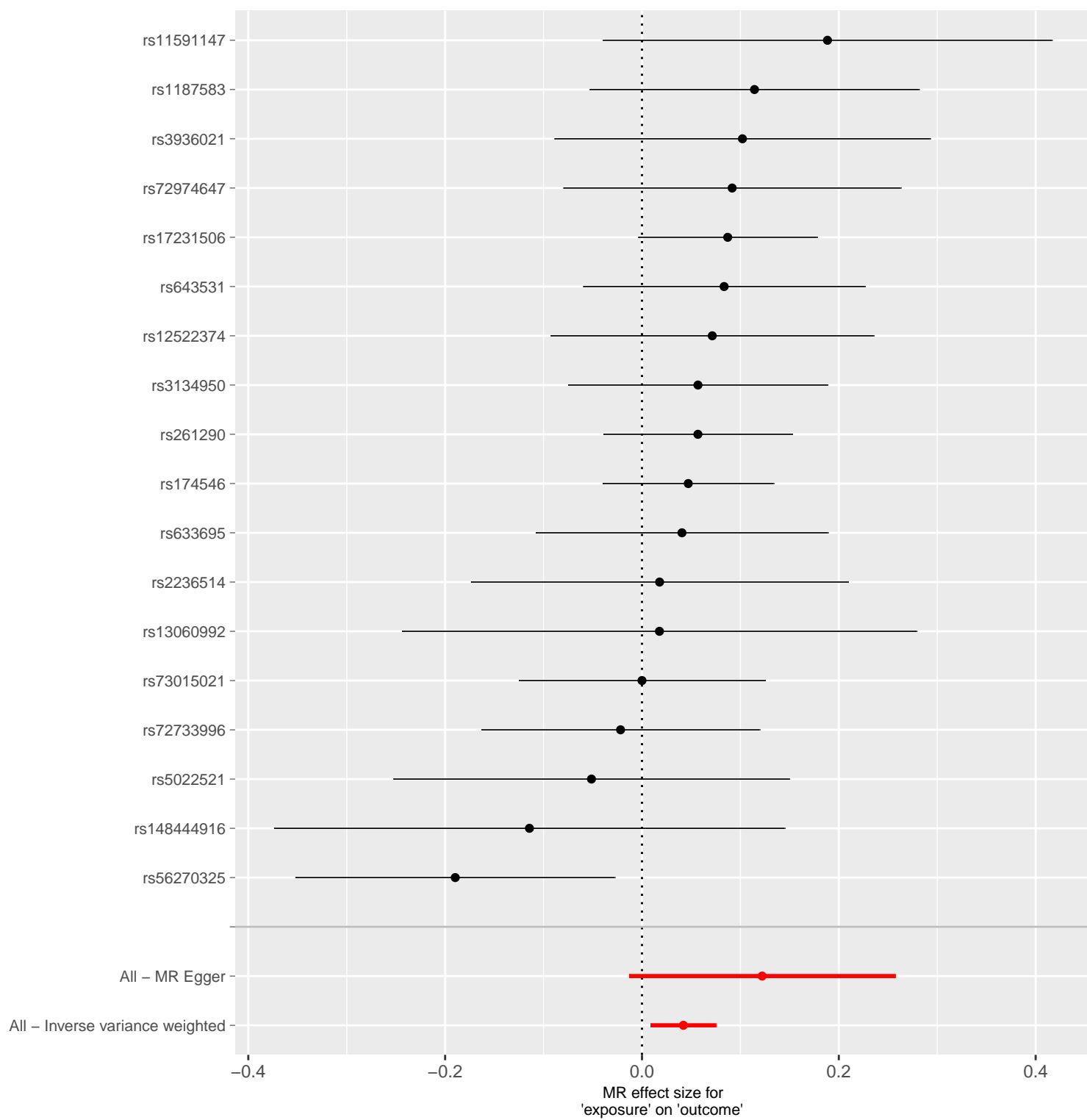

Supplement: Supplementary file 2 — Supplementary Material 2. [file 12944_2024_2103_MOESM2_ESM.zip › sFigure1∩╝êlipidomes-BC∩╝ë/GCST90277277/forest.pdf]

# MR Method

- Inverse variance weighted
- MR Egger

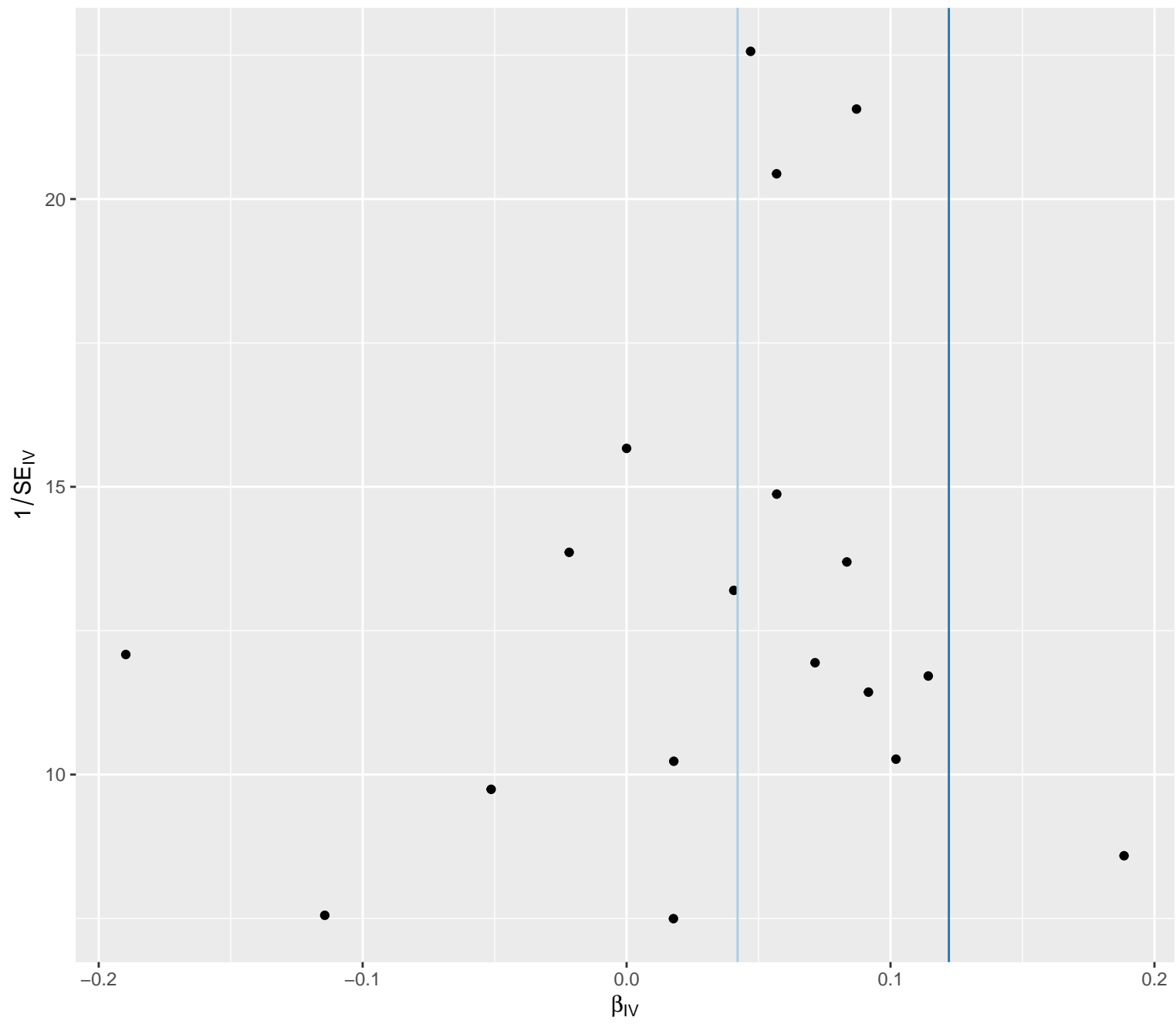

Supplement: Supplementary file 2 — Supplementary Material 2. [file 12944_2024_2103_MOESM2_ESM.zip › sFigure1∩╝êlipidomes-BC∩╝ë/GCST90277277/funnelplot.pdf]

## MR Test

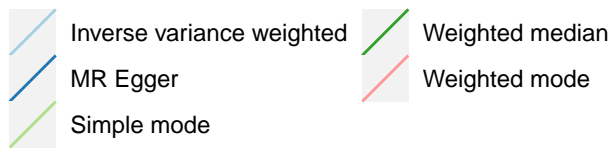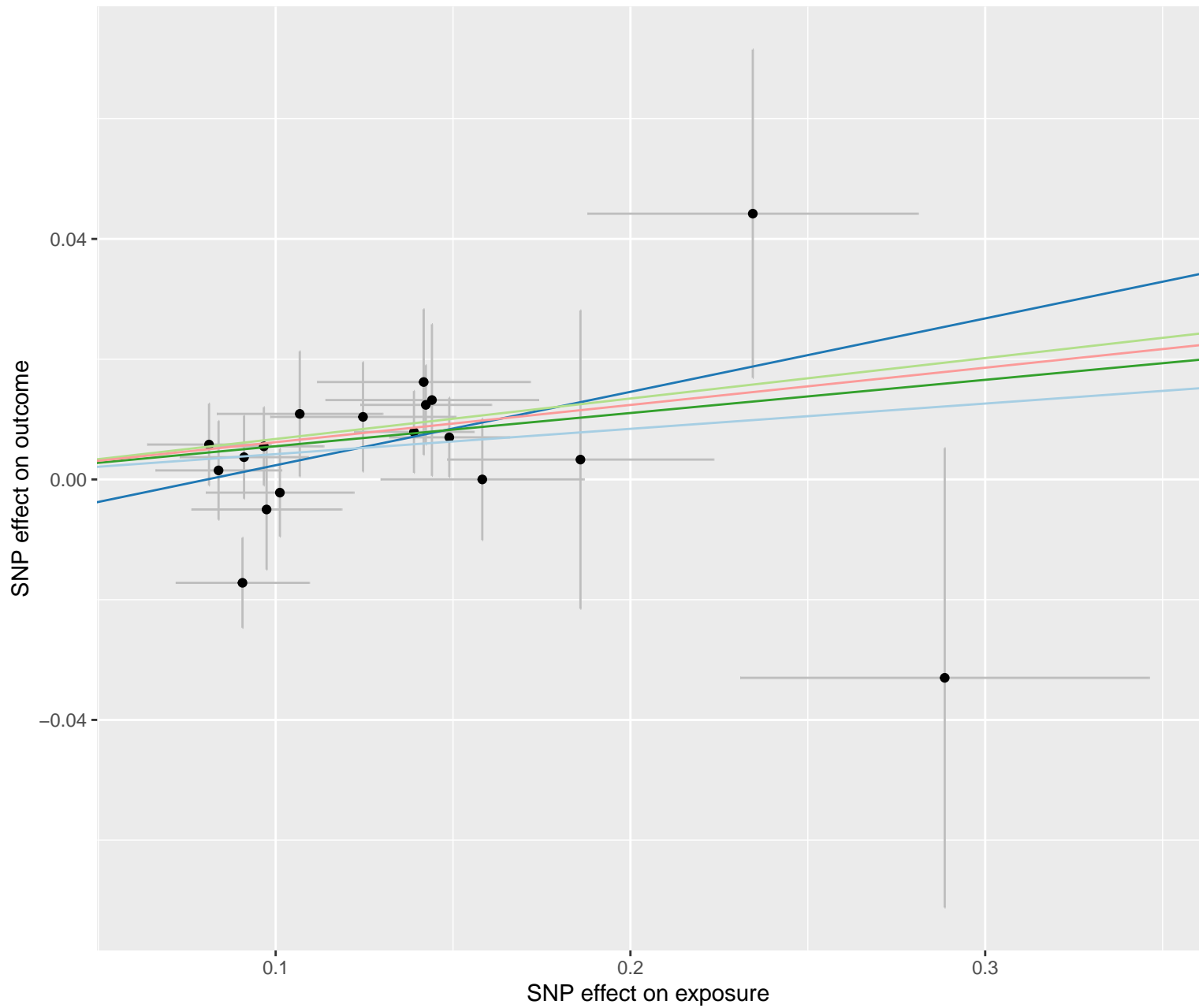

Supplement: Supplementary file 2 — Supplementary Material 2. [file 12944_2024_2103_MOESM2_ESM.zip › sFigure1∩╝êlipidomes-BC∩╝ë/GCST90277277/scatter.pdf]

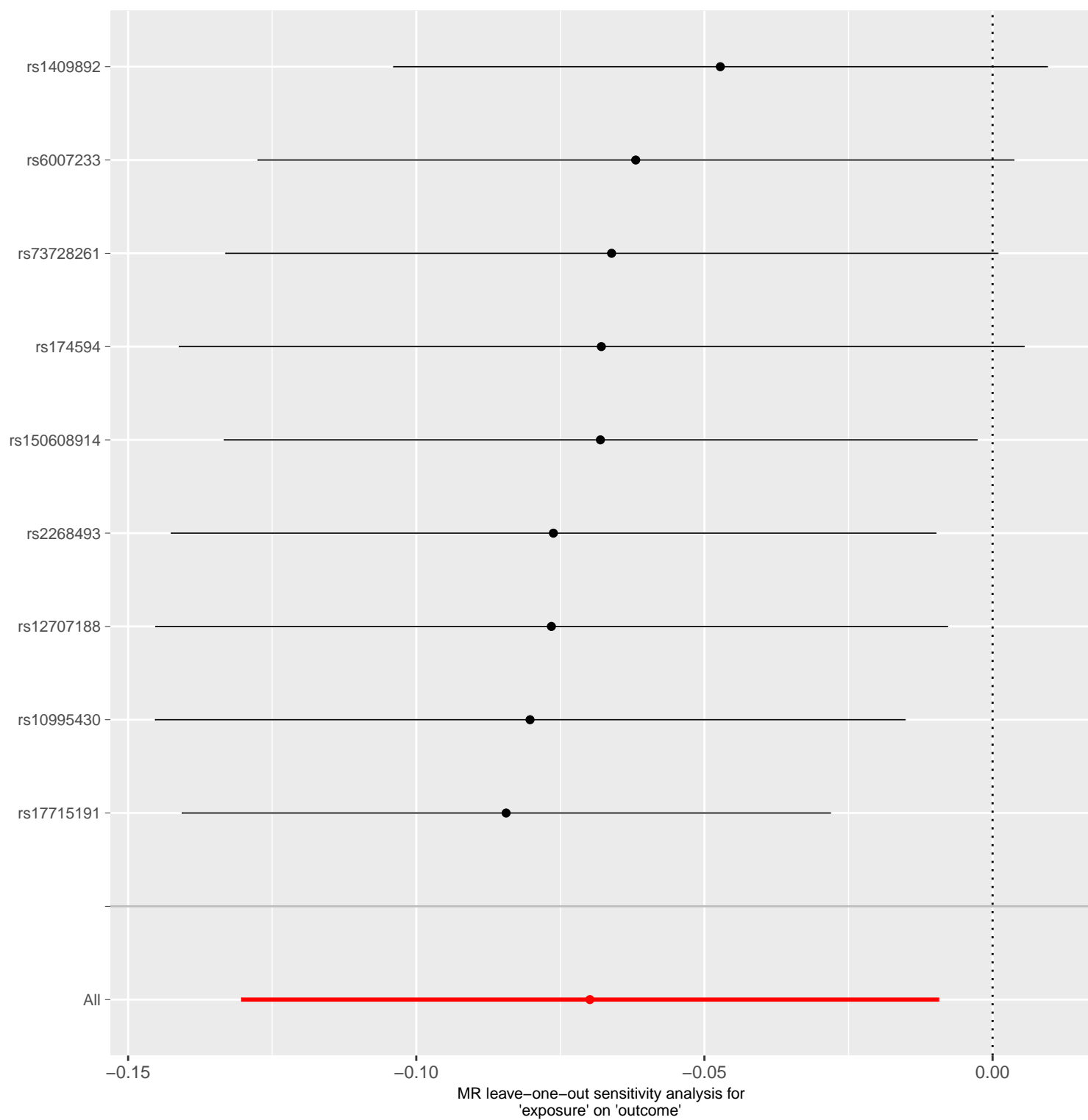

Supplement: Supplementary file 2 — Supplementary Material 2. [file 12944_2024_2103_MOESM2_ESM.zip › sFigure1∩╝êlipidomes-BC∩╝ë/GCST90277284/sensitivity-analysis.pdf]

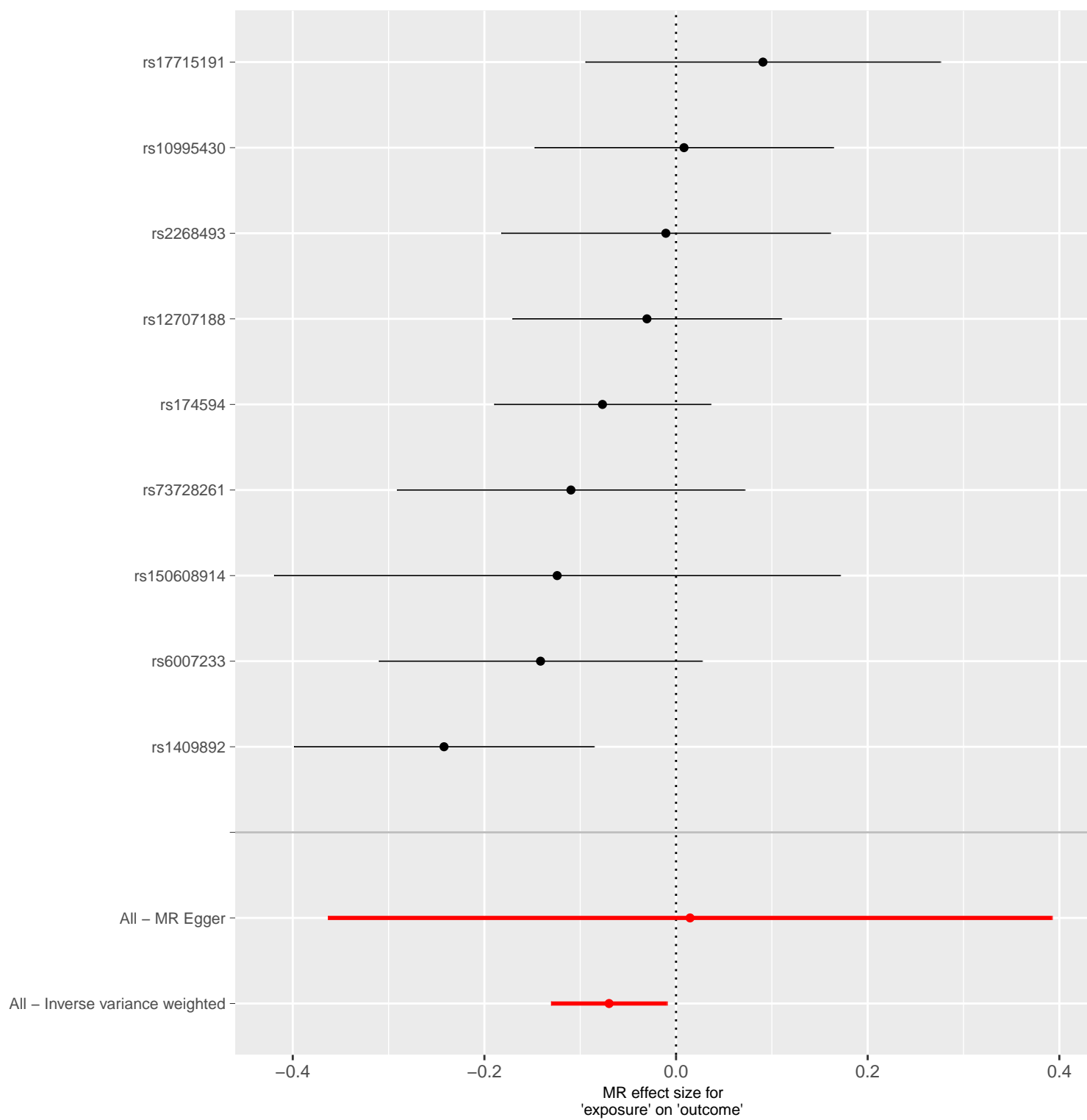

Supplement: Supplementary file 2 — Supplementary Material 2. [file 12944_2024_2103_MOESM2_ESM.zip › sFigure1∩╝êlipidomes-BC∩╝ë/GCST90277284/forest.pdf]

# MR Method

- Inverse variance weighted
- MR Egger

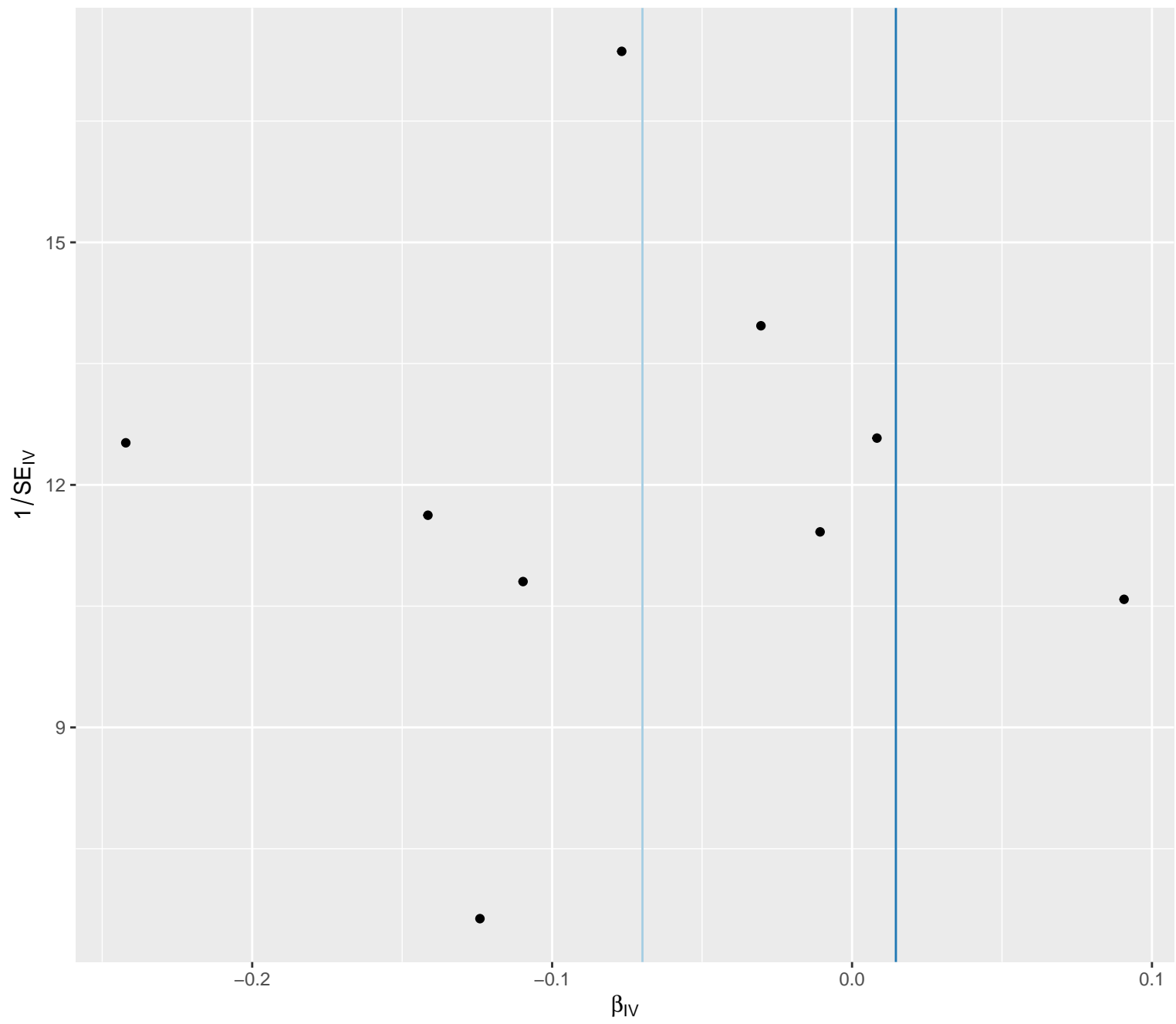

Supplement: Supplementary file 2 — Supplementary Material 2. [file 12944_2024_2103_MOESM2_ESM.zip › sFigure1∩╝êlipidomes-BC∩╝ë/GCST90277284/funnelplot.pdf]

# MR Test

- Inverse variance weighted
- MR Egger
- Simple mode
- Weighted median
- Weighted mode

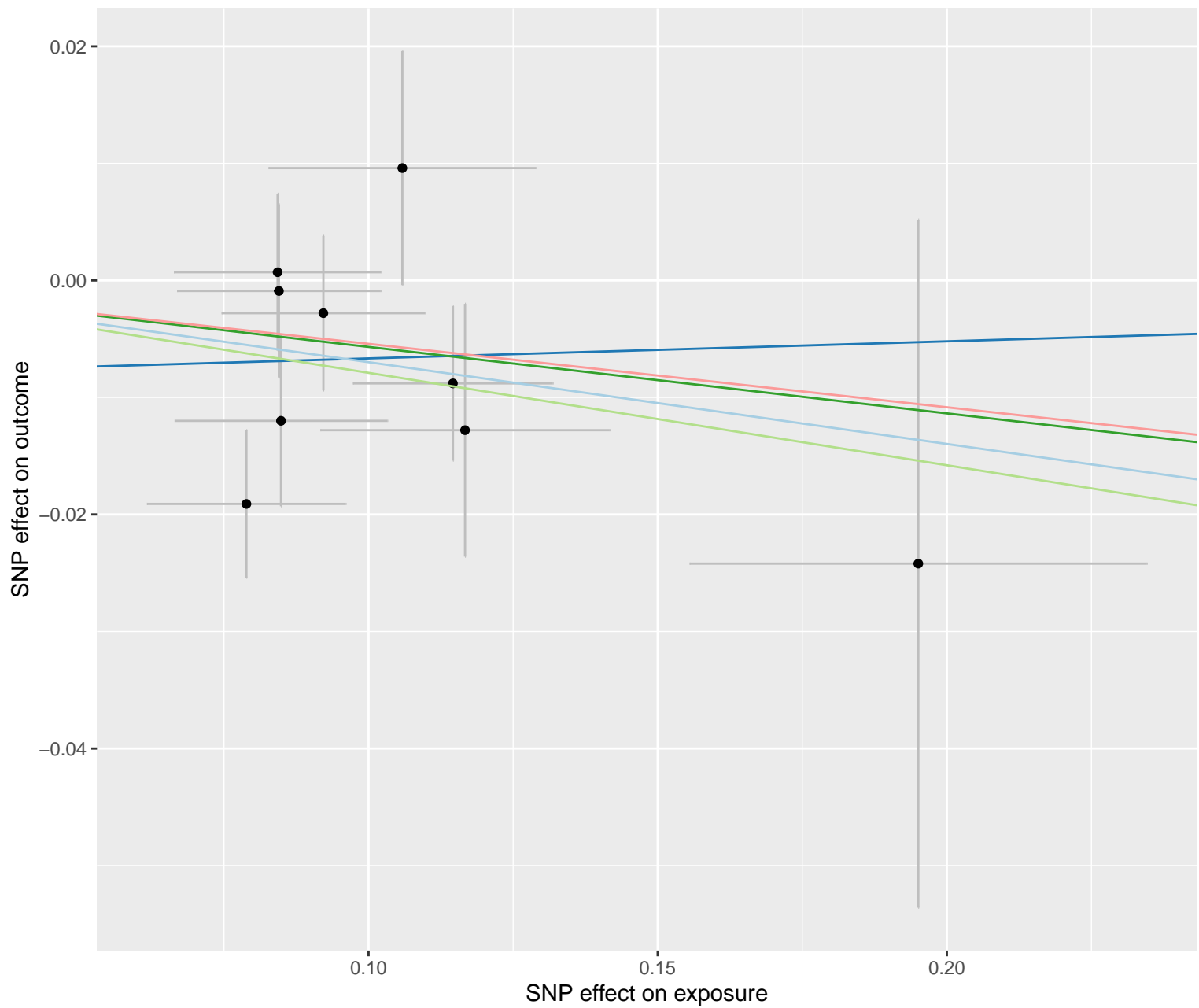

Supplement: Supplementary file 2 — Supplementary Material 2. [file 12944_2024_2103_MOESM2_ESM.zip › sFigure1∩╝êlipidomes-BC∩╝ë/GCST90277284/scatter.pdf]

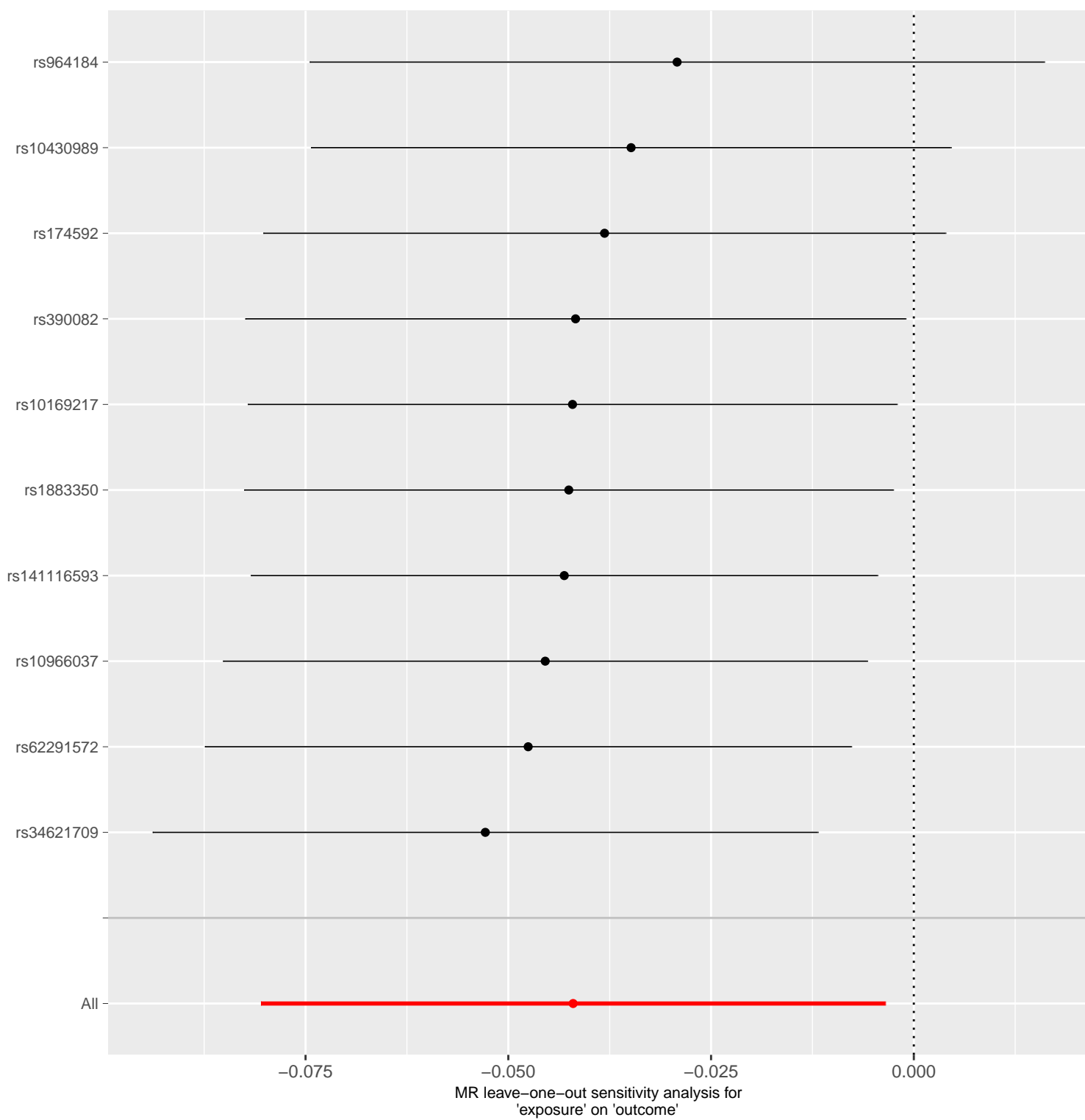

Supplement: Supplementary file 2 — Supplementary Material 2. [file 12944_2024_2103_MOESM2_ESM.zip › sFigure1∩╝êlipidomes-BC∩╝ë/GCST90277410/sensitivity-analysis.pdf]

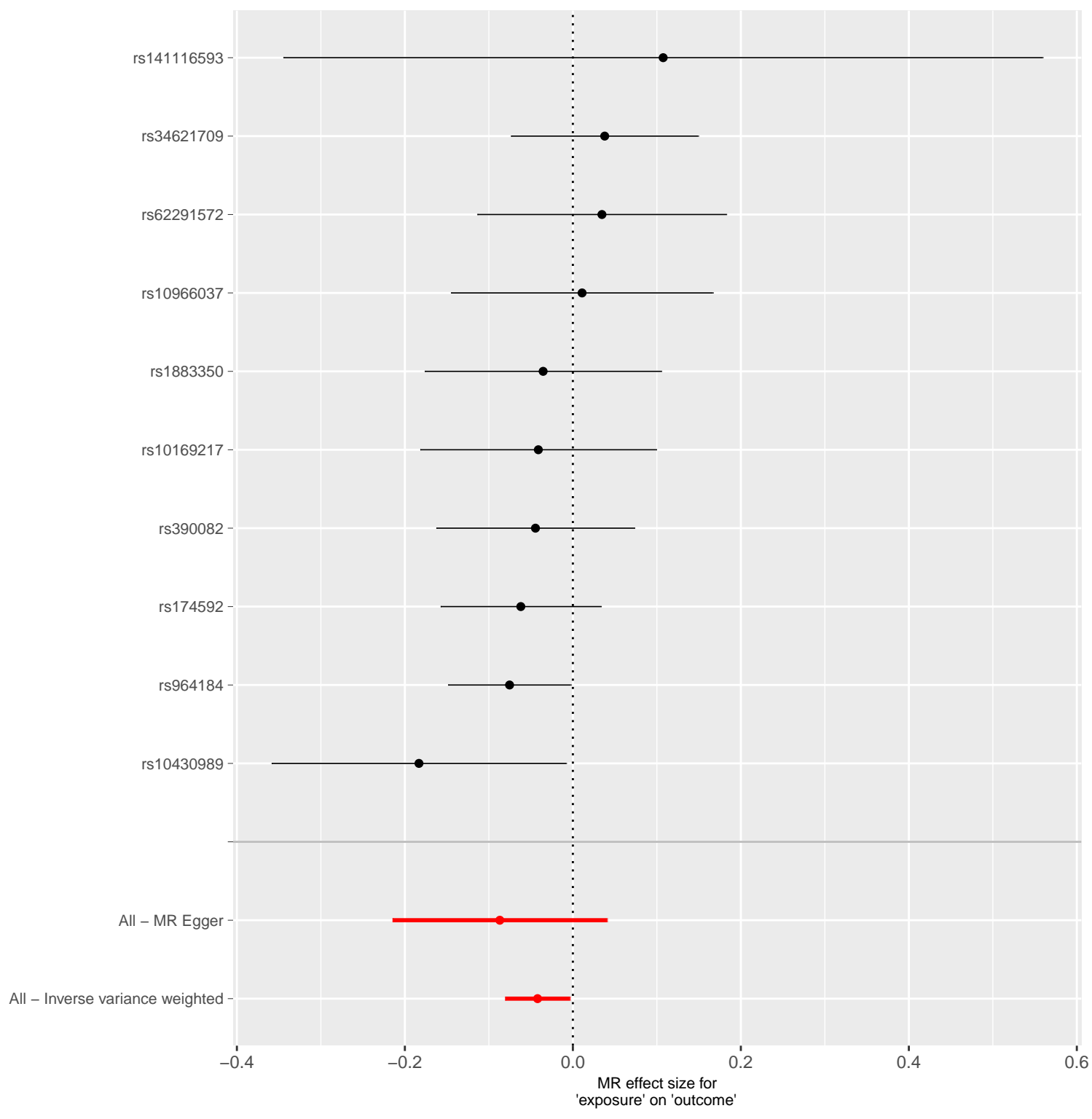

Supplement: Supplementary file 2 — Supplementary Material 2. [file 12944_2024_2103_MOESM2_ESM.zip › sFigure1∩╝êlipidomes-BC∩╝ë/GCST90277410/forest.pdf]

# MR Method

- Inverse variance weighted
- MR Egger

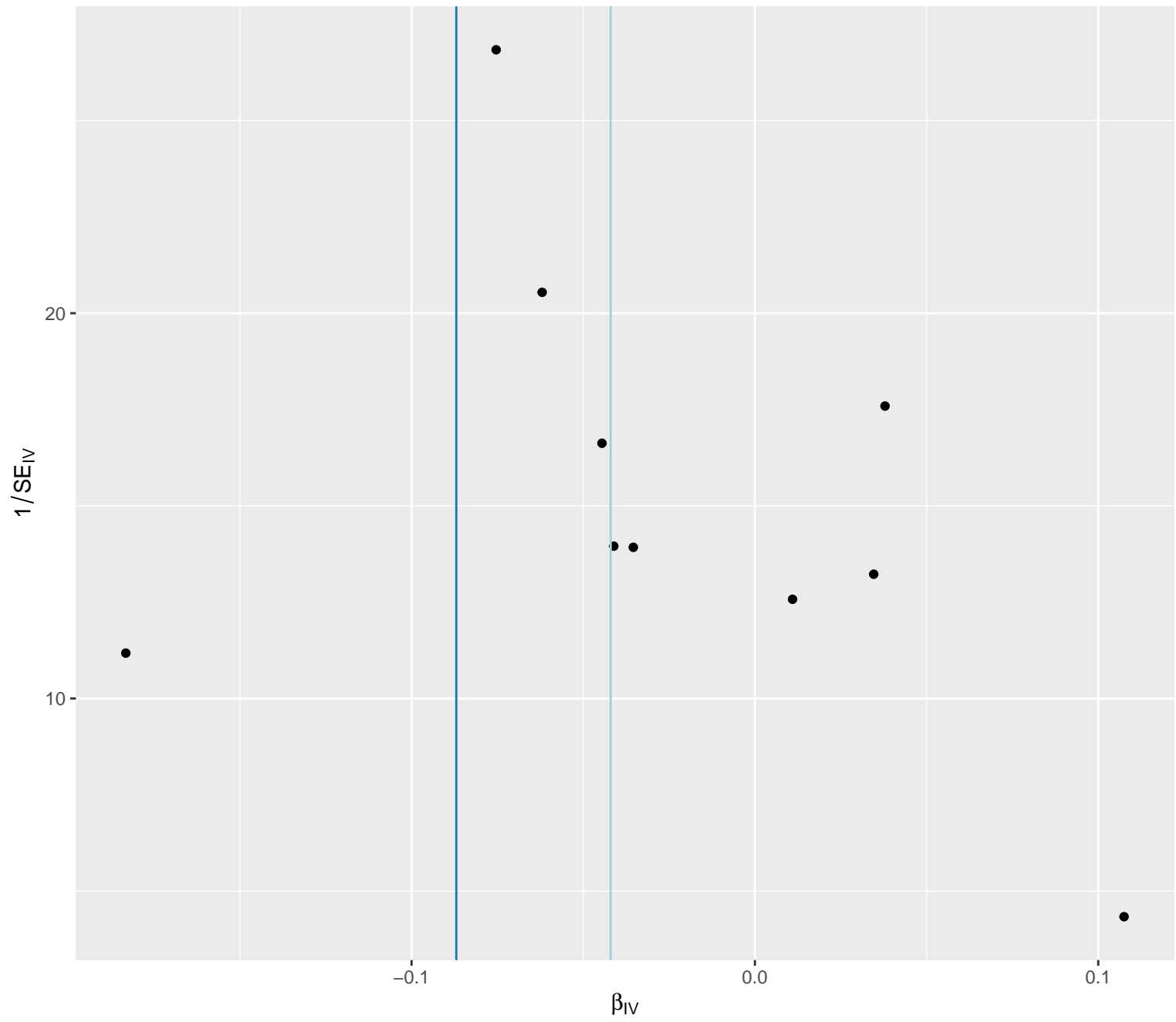

Supplement: Supplementary file 2 — Supplementary Material 2. [file 12944_2024_2103_MOESM2_ESM.zip › sFigure1∩╝êlipidomes-BC∩╝ë/GCST90277410/funnelplot.pdf]

# MR Test

- Inverse variance weighted
- MR Egger
- Simple mode
- Weighted median
- Weighted mode

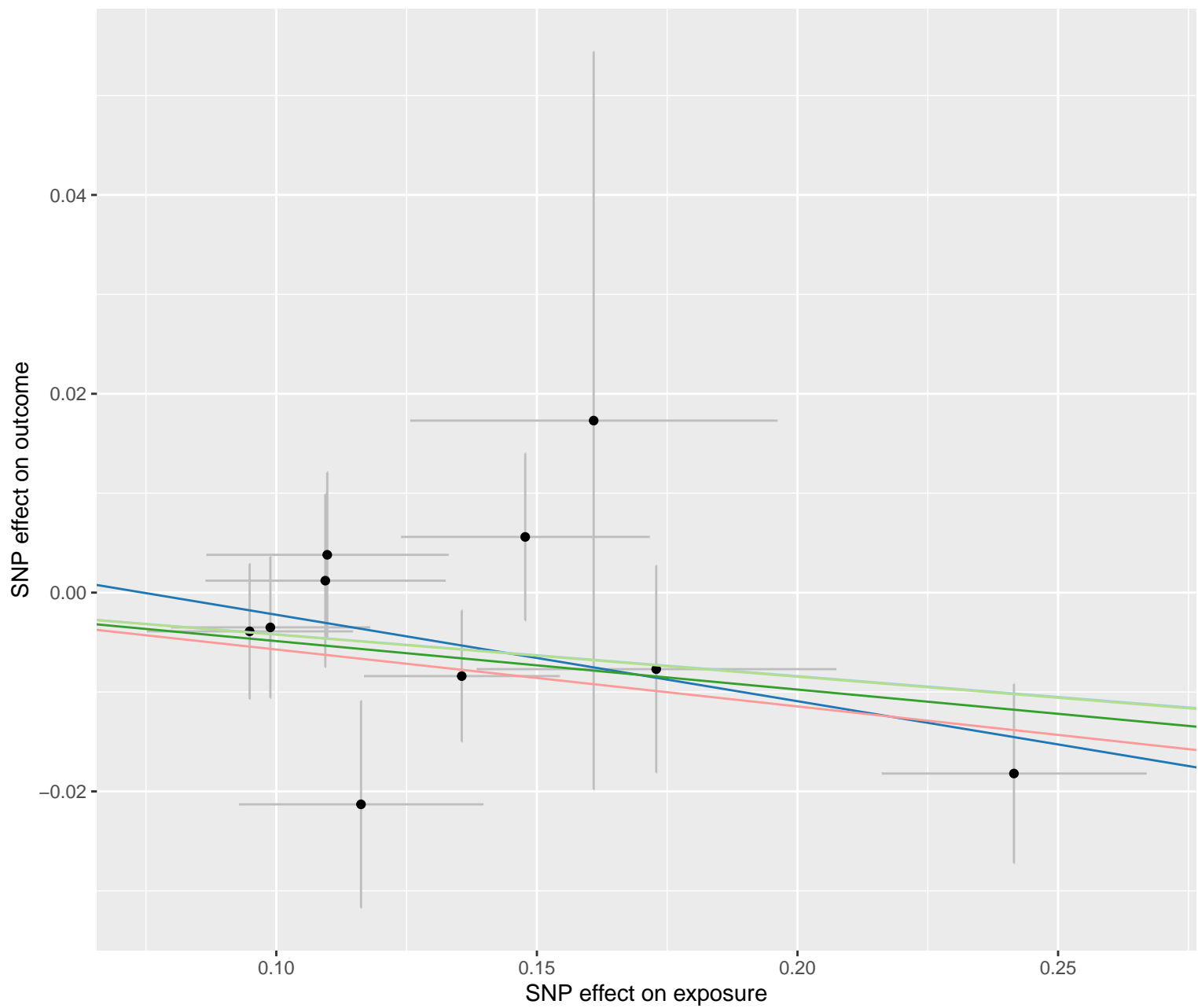

Supplement: Supplementary file 2 — Supplementary Material 2. [file 12944_2024_2103_MOESM2_ESM.zip › sFigure1∩╝êlipidomes-BC∩╝ë/GCST90277410/scatter.pdf]

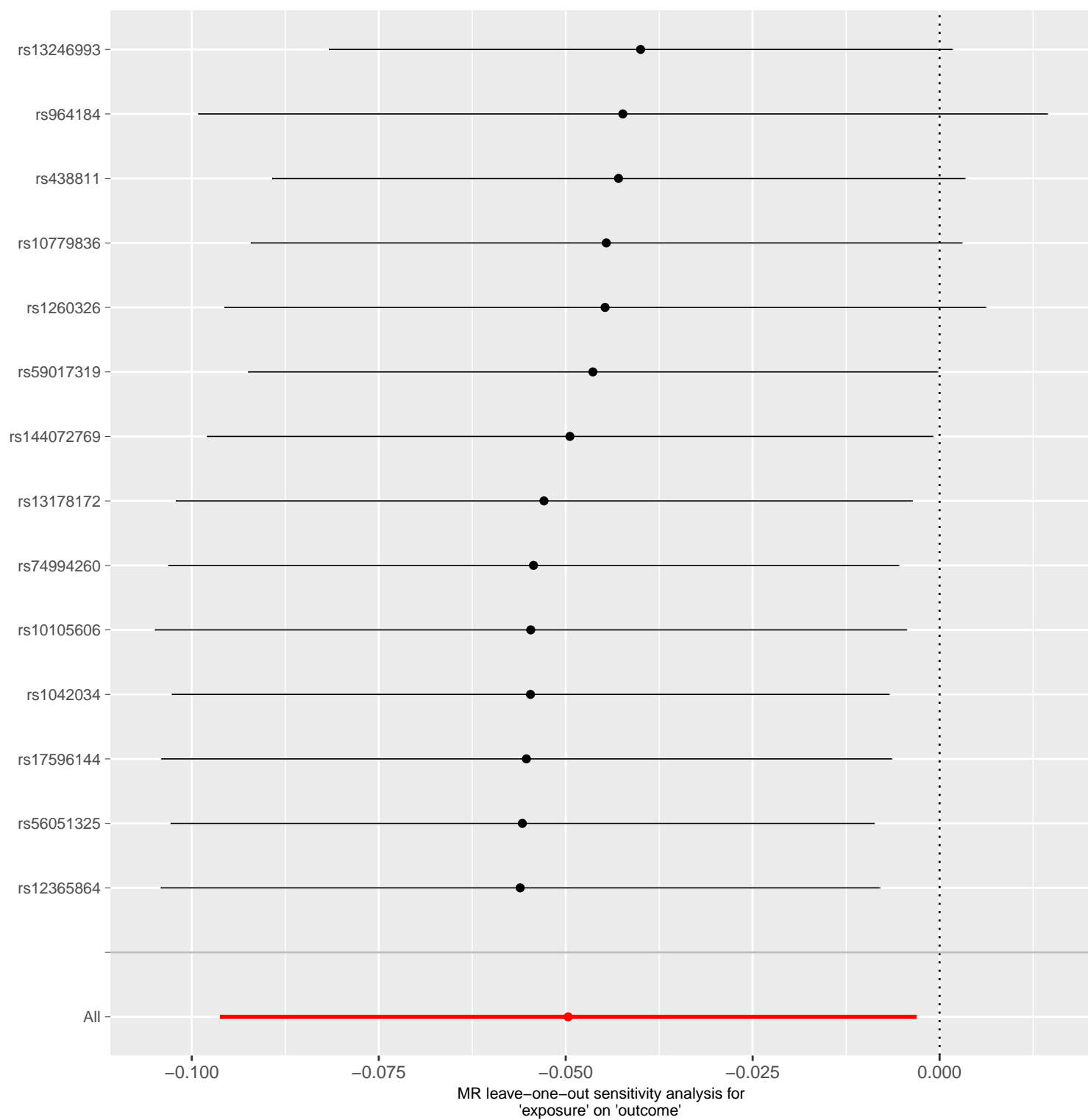

Supplement: Supplementary file 2 — Supplementary Material 2. [file 12944_2024_2103_MOESM2_ESM.zip › sFigure1∩╝êlipidomes-BC∩╝ë/GCST90277406/sensitivity-analysis.pdf]

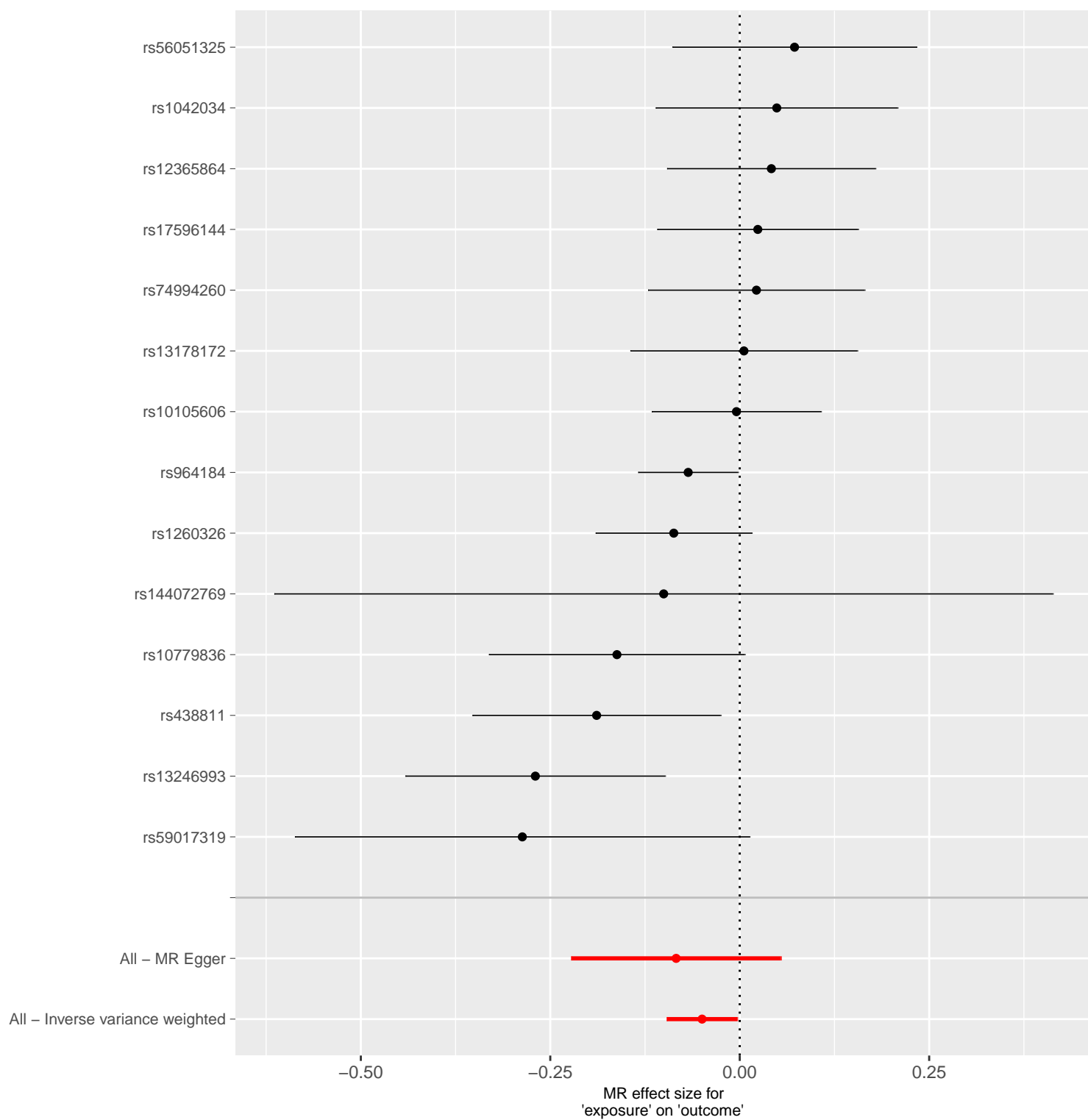

Supplement: Supplementary file 2 — Supplementary Material 2. [file 12944_2024_2103_MOESM2_ESM.zip › sFigure1∩╝êlipidomes-BC∩╝ë/GCST90277406/forest.pdf]

# MR Method

- Inverse variance weighted
- MR Egger

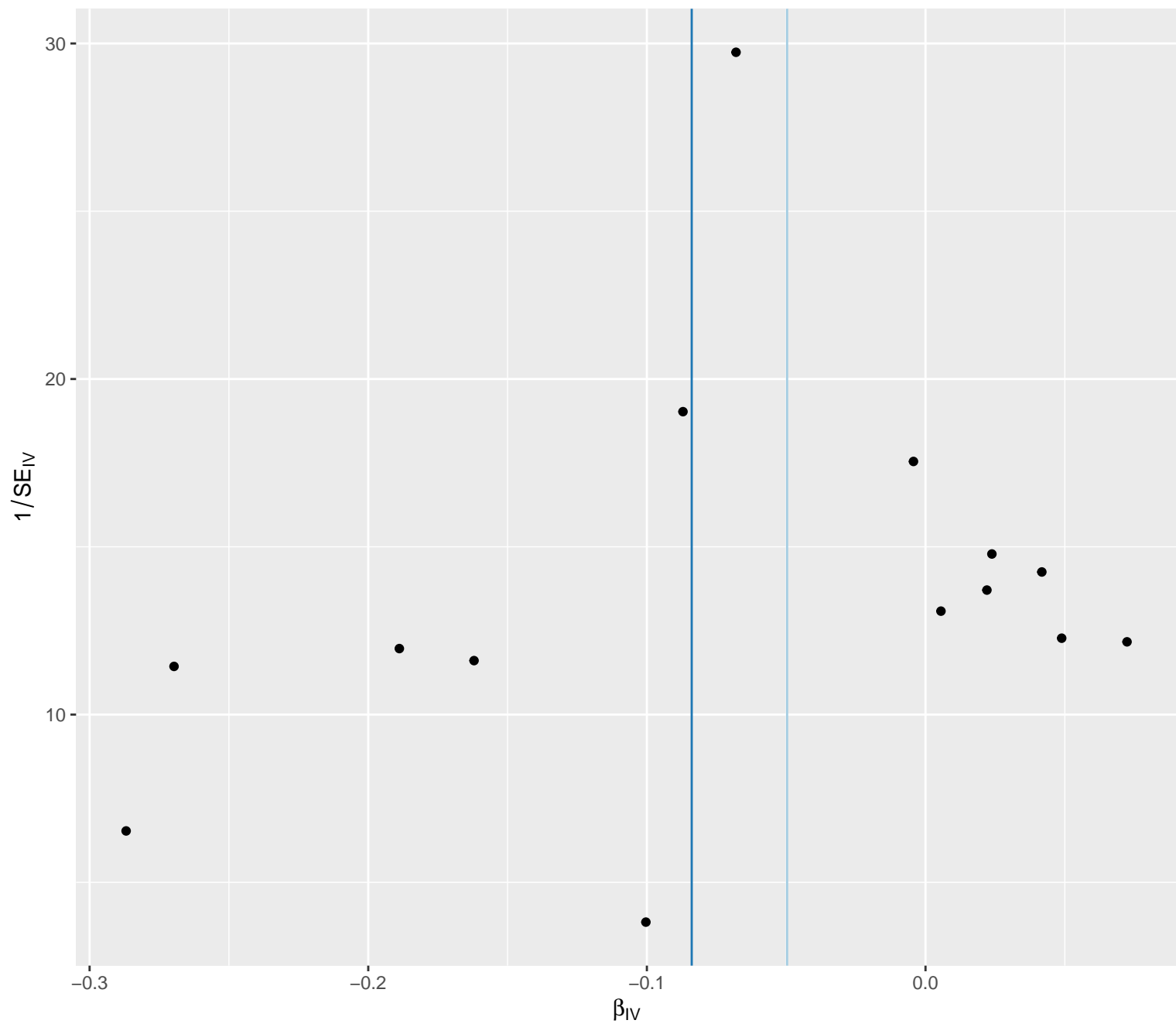

Supplement: Supplementary file 2 — Supplementary Material 2. [file 12944_2024_2103_MOESM2_ESM.zip › sFigure1∩╝êlipidomes-BC∩╝ë/GCST90277406/funnelplot.pdf]

# MR Test

- Inverse variance weighted
- MR Egger
- Simple mode
- Weighted median
- Weighted mode

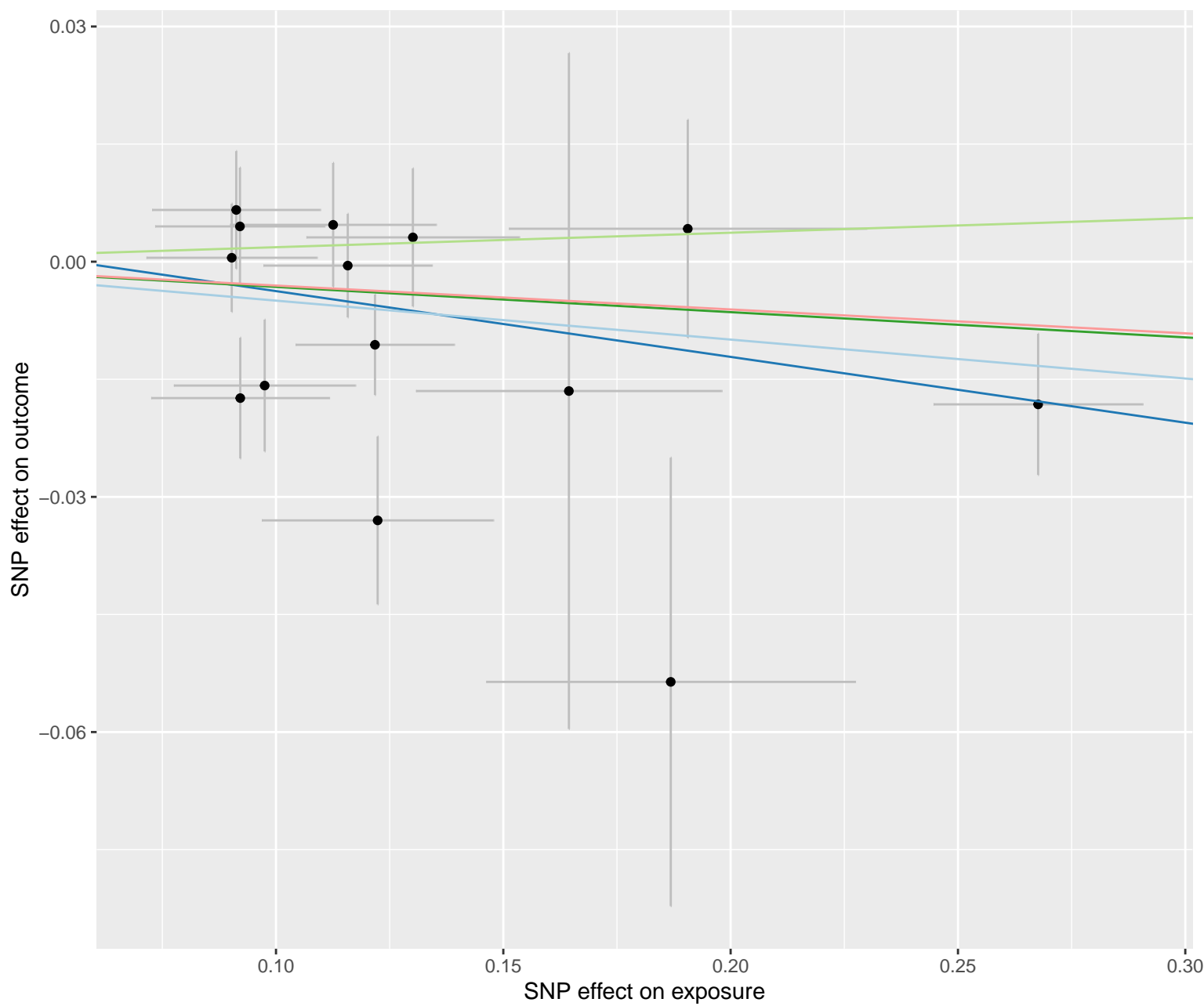

Supplement: Supplementary file 2 — Supplementary Material 2. [file 12944_2024_2103_MOESM2_ESM.zip › sFigure1∩╝êlipidomes-BC∩╝ë/GCST90277406/scatter.pdf]

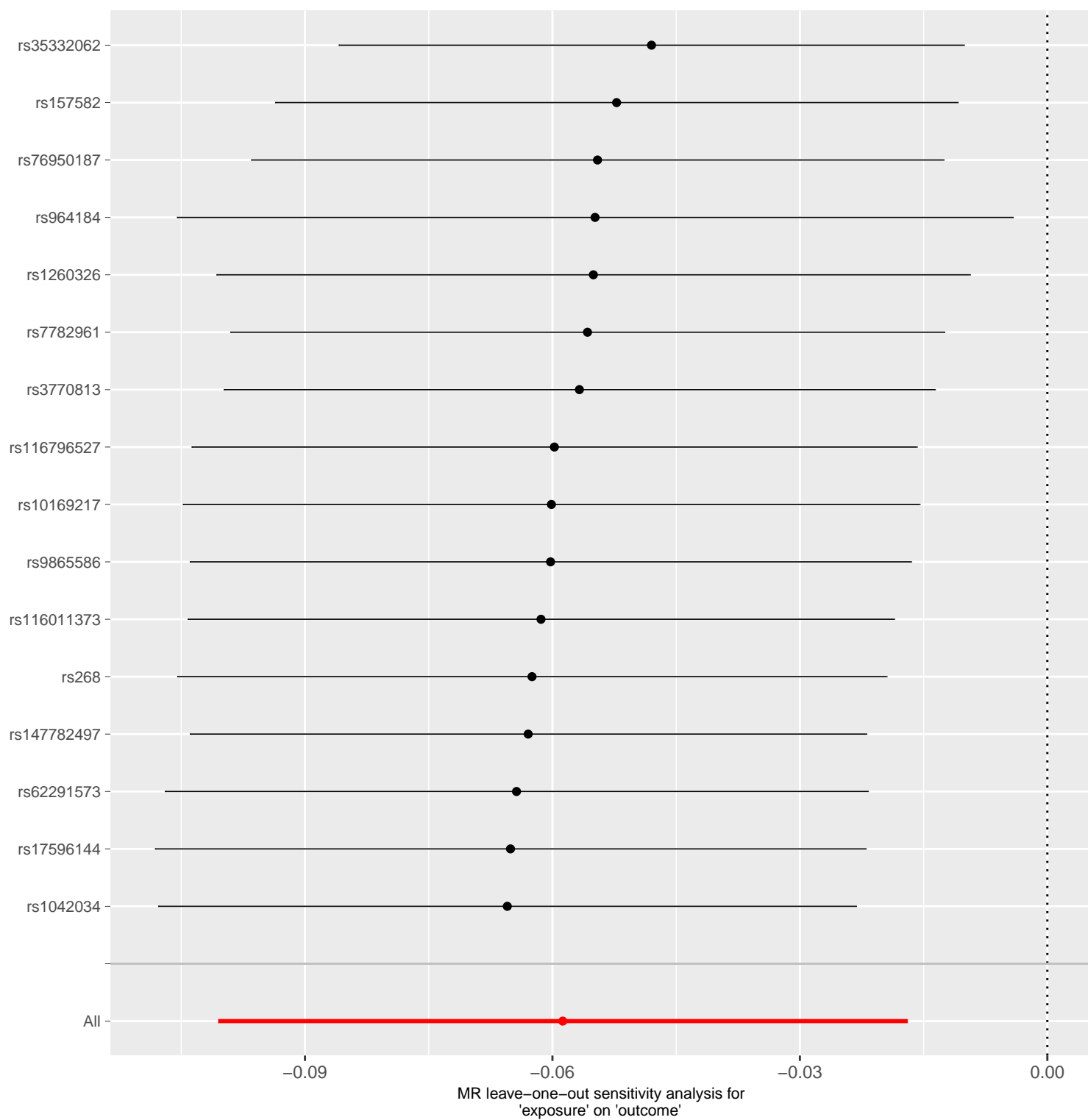

Supplement: Supplementary file 2 — Supplementary Material 2. [file 12944_2024_2103_MOESM2_ESM.zip › sFigure1∩╝êlipidomes-BC∩╝ë/GCST90277261/sensitivity-analysis.pdf]

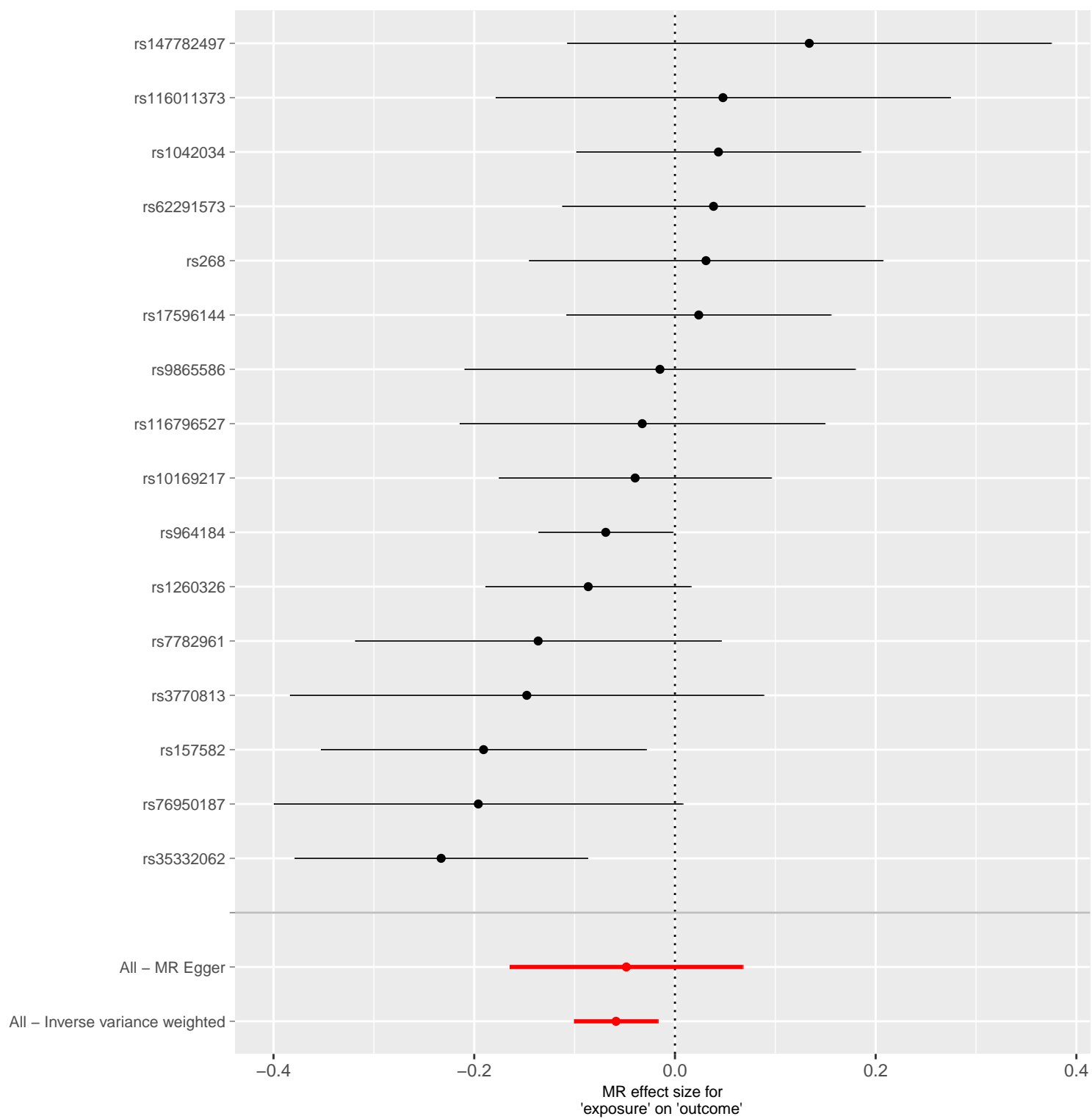

Supplement: Supplementary file 2 — Supplementary Material 2. [file 12944_2024_2103_MOESM2_ESM.zip › sFigure1∩╝êlipidomes-BC∩╝ë/GCST90277261/forest.pdf]

# MR Method

- Inverse variance weighted
- MR Egger

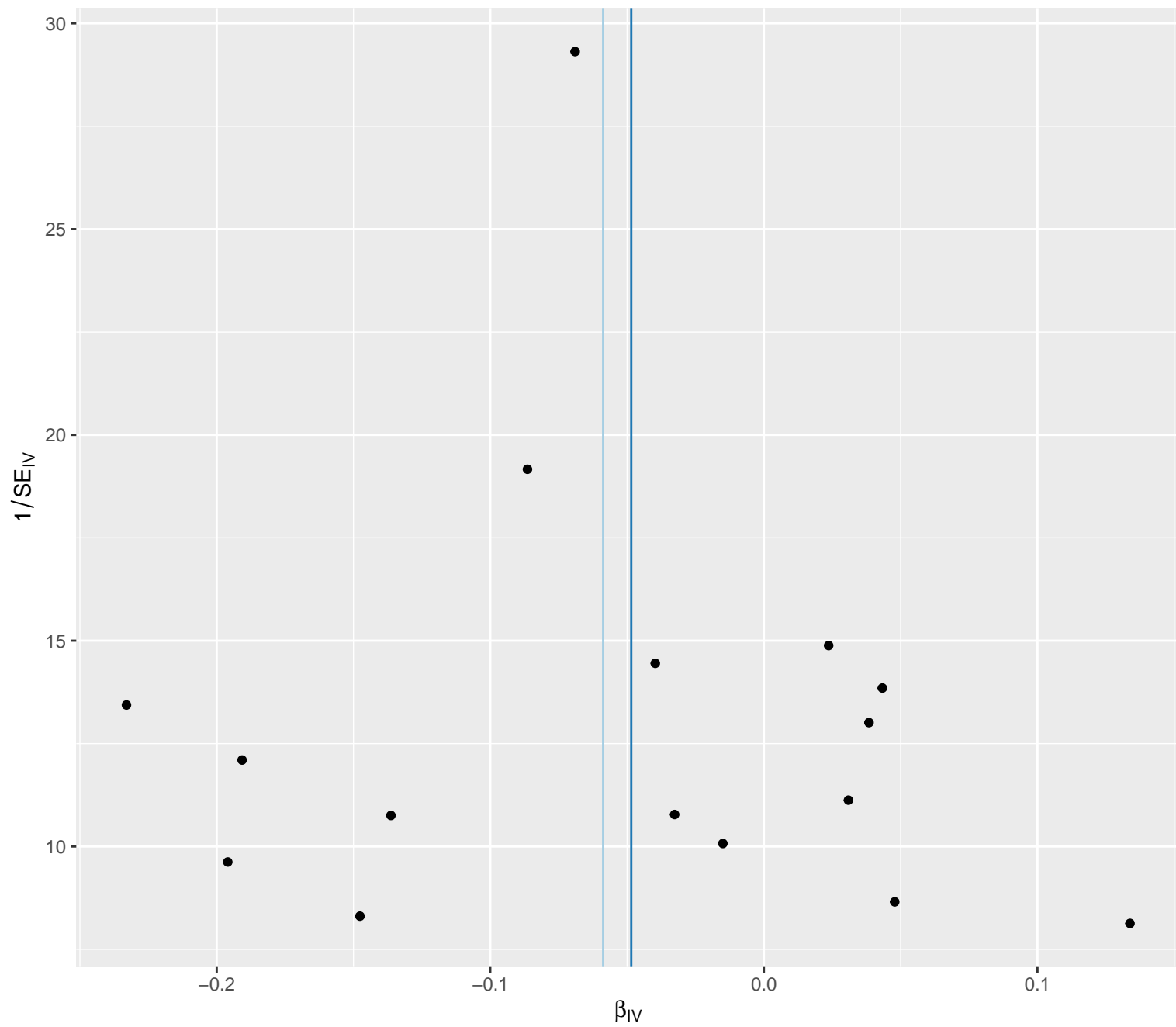

Supplement: Supplementary file 2 — Supplementary Material 2. [file 12944_2024_2103_MOESM2_ESM.zip › sFigure1∩╝êlipidomes-BC∩╝ë/GCST90277261/funnelplot.pdf]

# MR Test

- Inverse variance weighted
- MR Egger
- Simple mode
- Weighted median
- Weighted mode

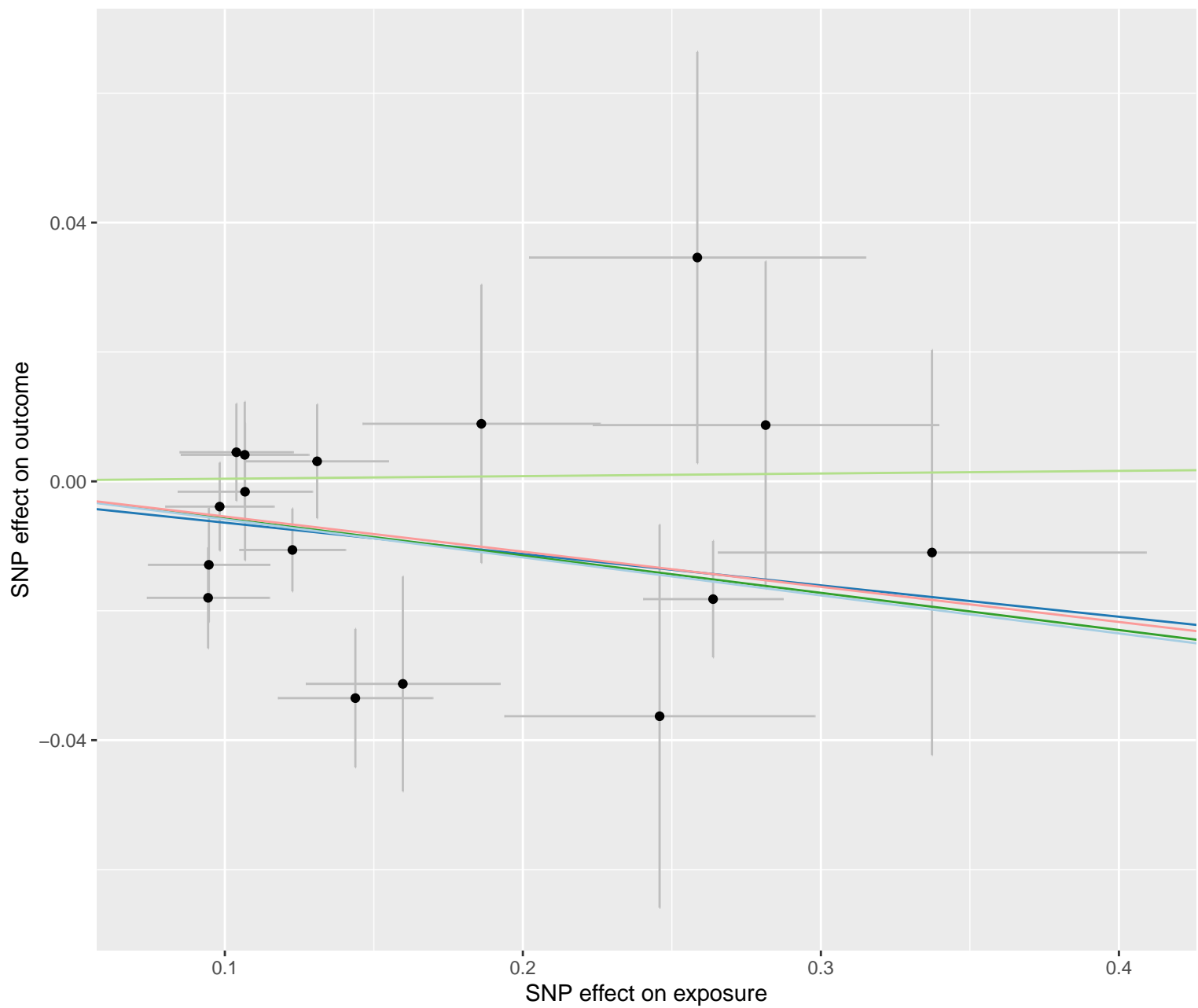

Supplement: Supplementary file 2 — Supplementary Material 2. [file 12944_2024_2103_MOESM2_ESM.zip › sFigure1∩╝êlipidomes-BC∩╝ë/GCST90277261/scatter.pdf]

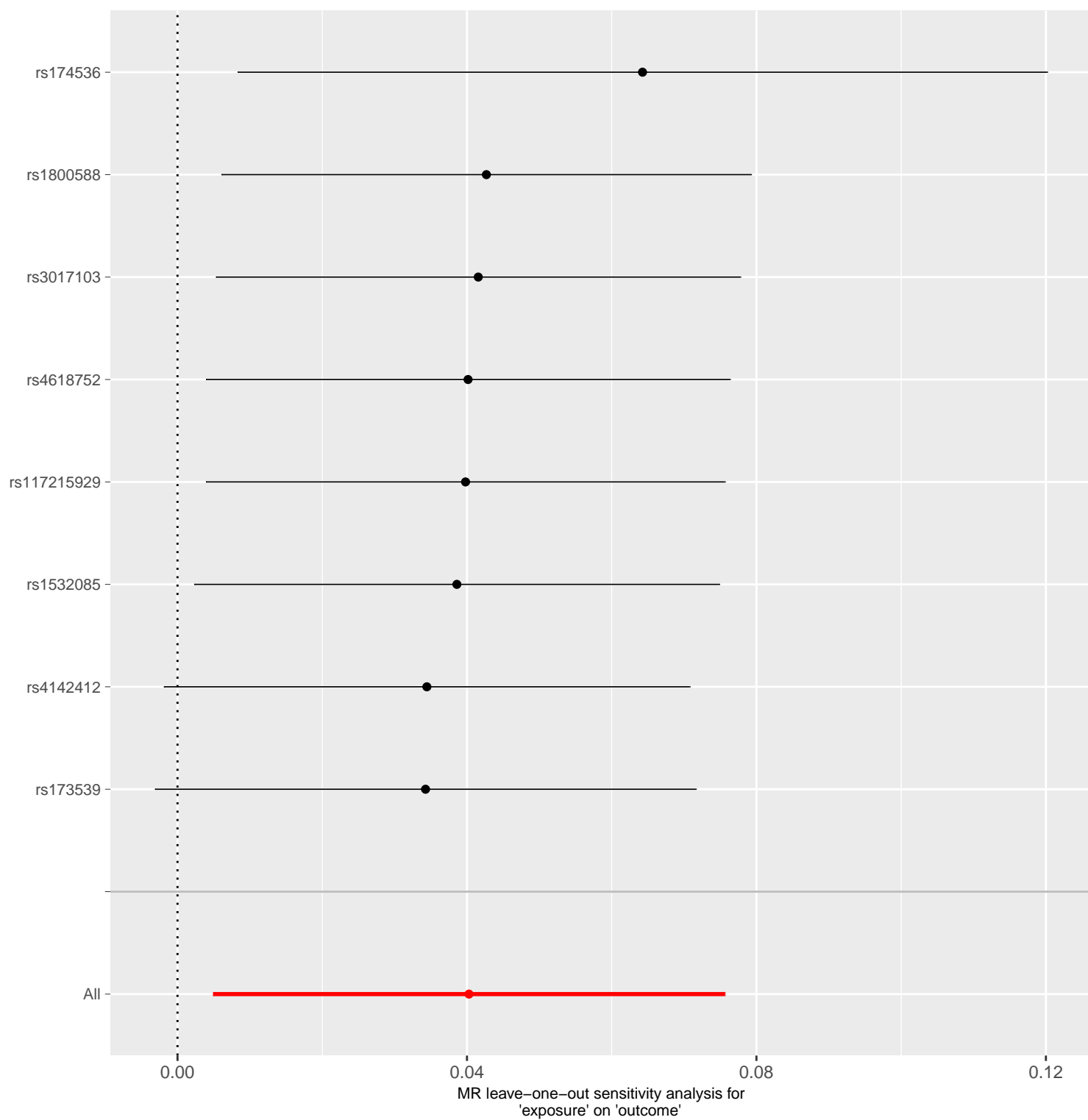

Supplement: Supplementary file 2 — Supplementary Material 2. [file 12944_2024_2103_MOESM2_ESM.zip › sFigure1∩╝êlipidomes-BC∩╝ë/GCST90277340/sensitivity-analysis.pdf]

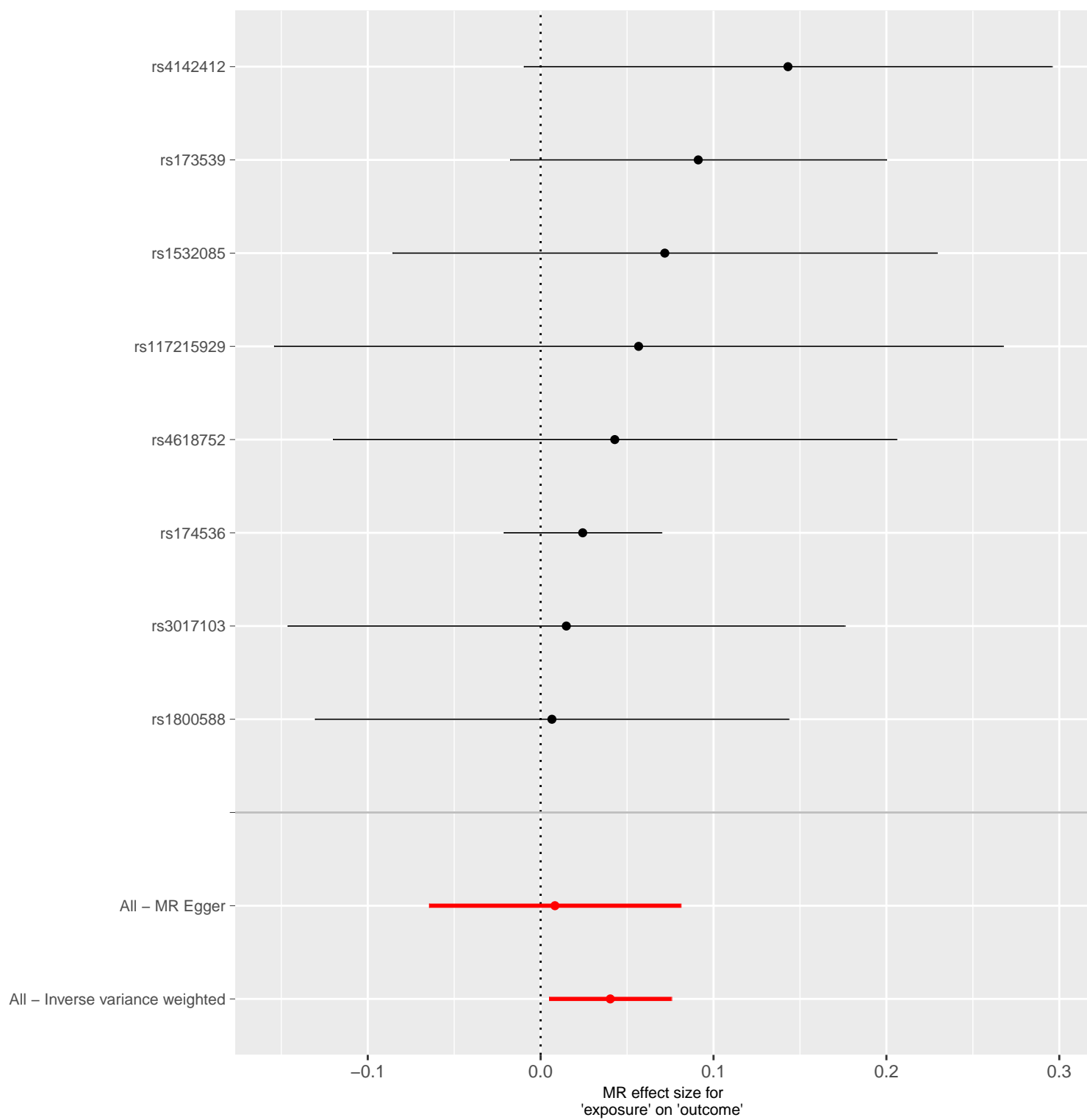

Supplement: Supplementary file 2 — Supplementary Material 2. [file 12944_2024_2103_MOESM2_ESM.zip › sFigure1∩╝êlipidomes-BC∩╝ë/GCST90277340/forest.pdf]

# MR Method

- Inverse variance weighted
- MR Egger

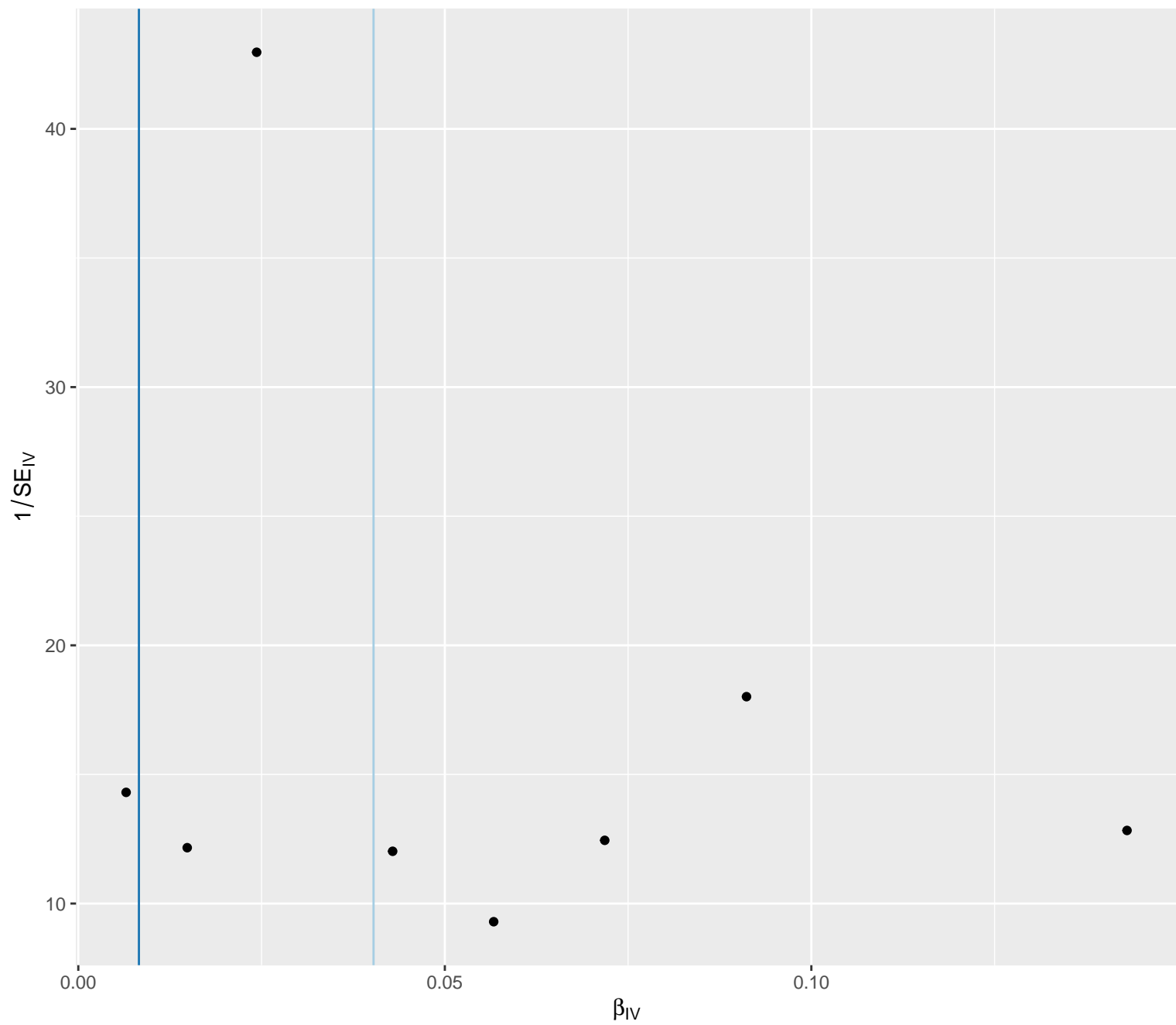

Supplement: Supplementary file 2 — Supplementary Material 2. [file 12944_2024_2103_MOESM2_ESM.zip › sFigure1∩╝êlipidomes-BC∩╝ë/GCST90277340/funnelplot.pdf]

# MR Test

- Inverse variance weighted
- MR Egger
- Simple mode
- Weighted median
- Weighted mode

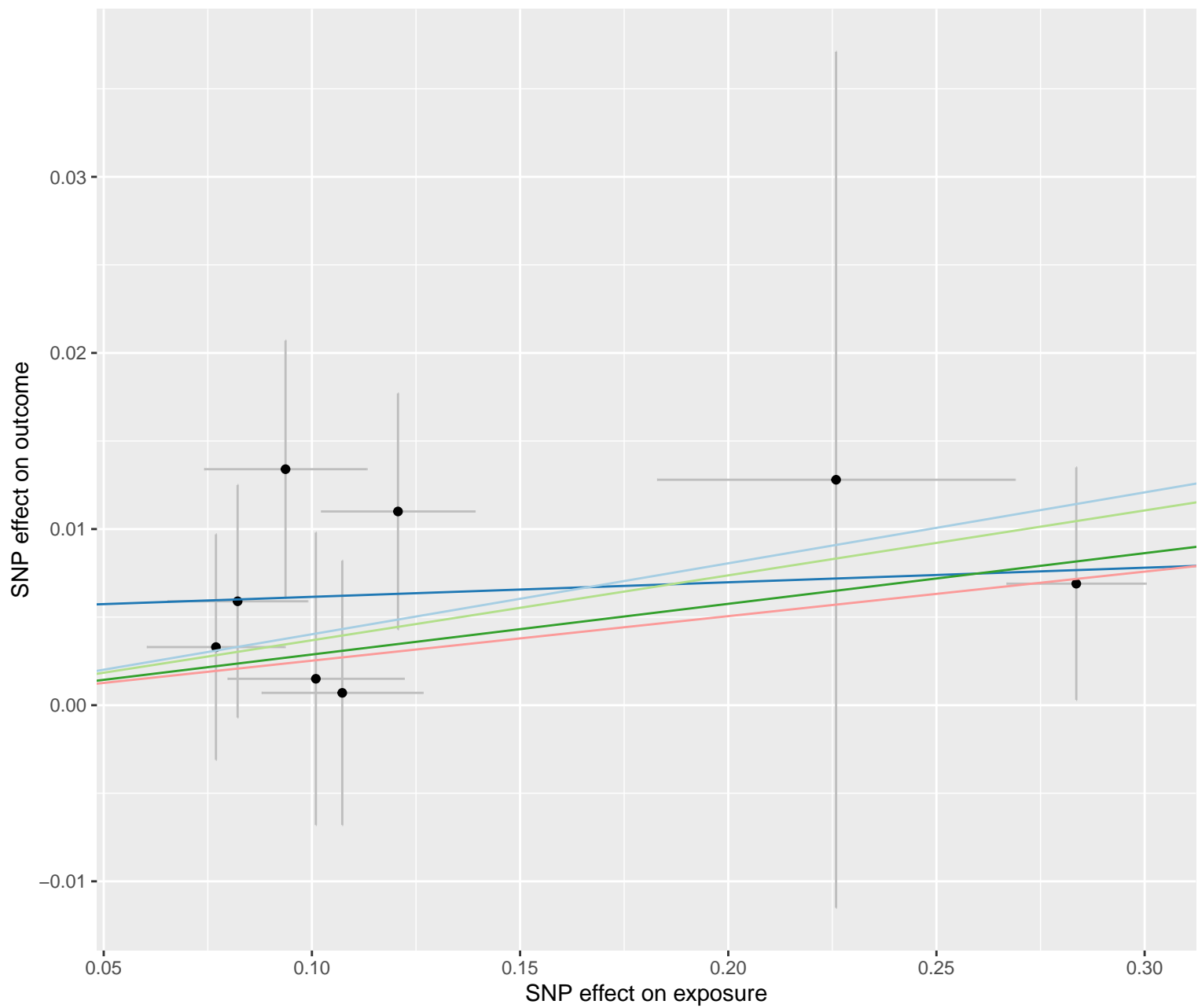

Supplement: Supplementary file 2 — Supplementary Material 2. [file 12944_2024_2103_MOESM2_ESM.zip › sFigure1∩╝êlipidomes-BC∩╝ë/GCST90277340/scatter.pdf]

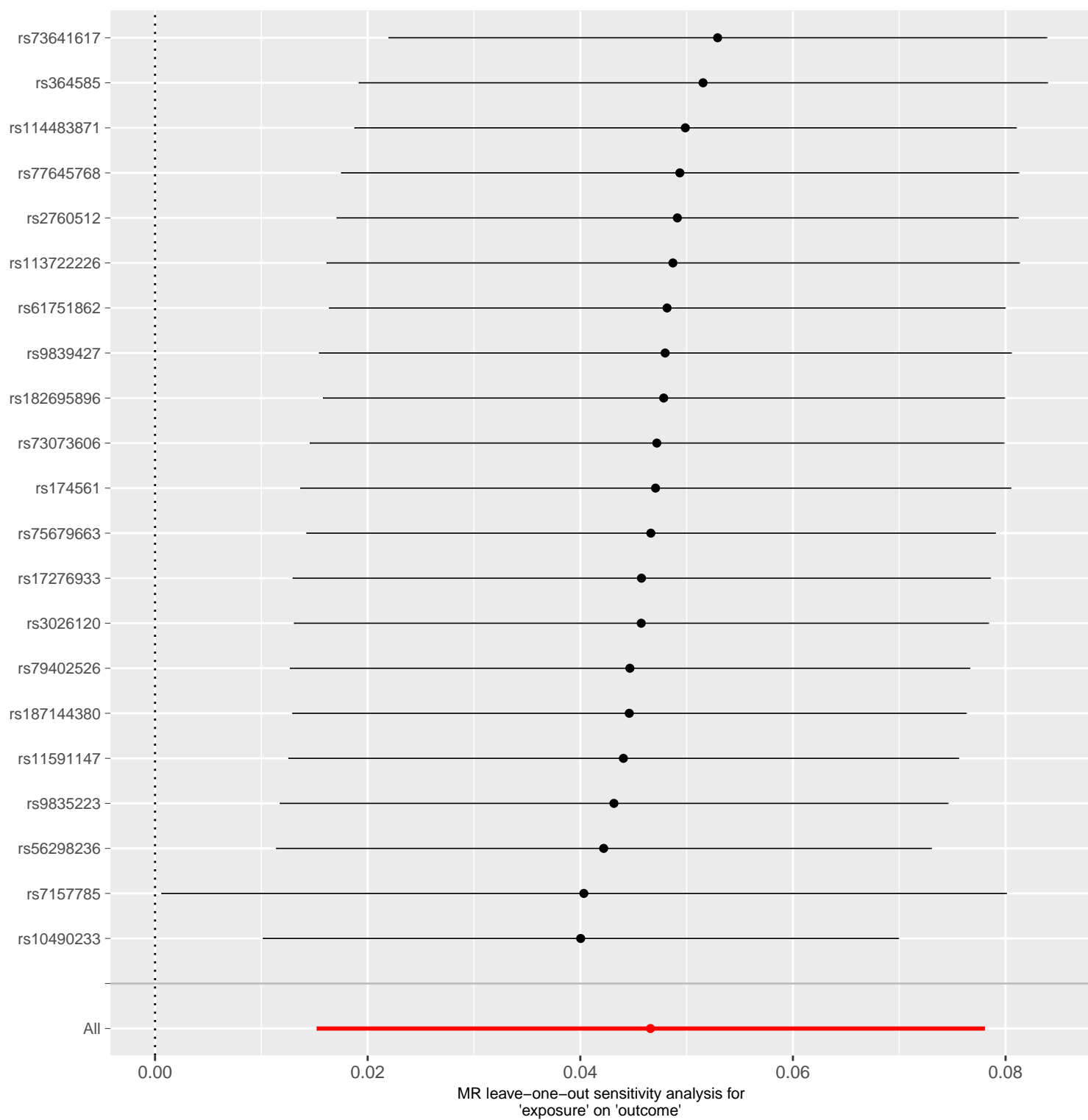

Supplement: Supplementary file 2 — Supplementary Material 2. [file 12944_2024_2103_MOESM2_ESM.zip › sFigure1∩╝êlipidomes-BC∩╝ë/GCST90277377/sensitivity-analysis.pdf]

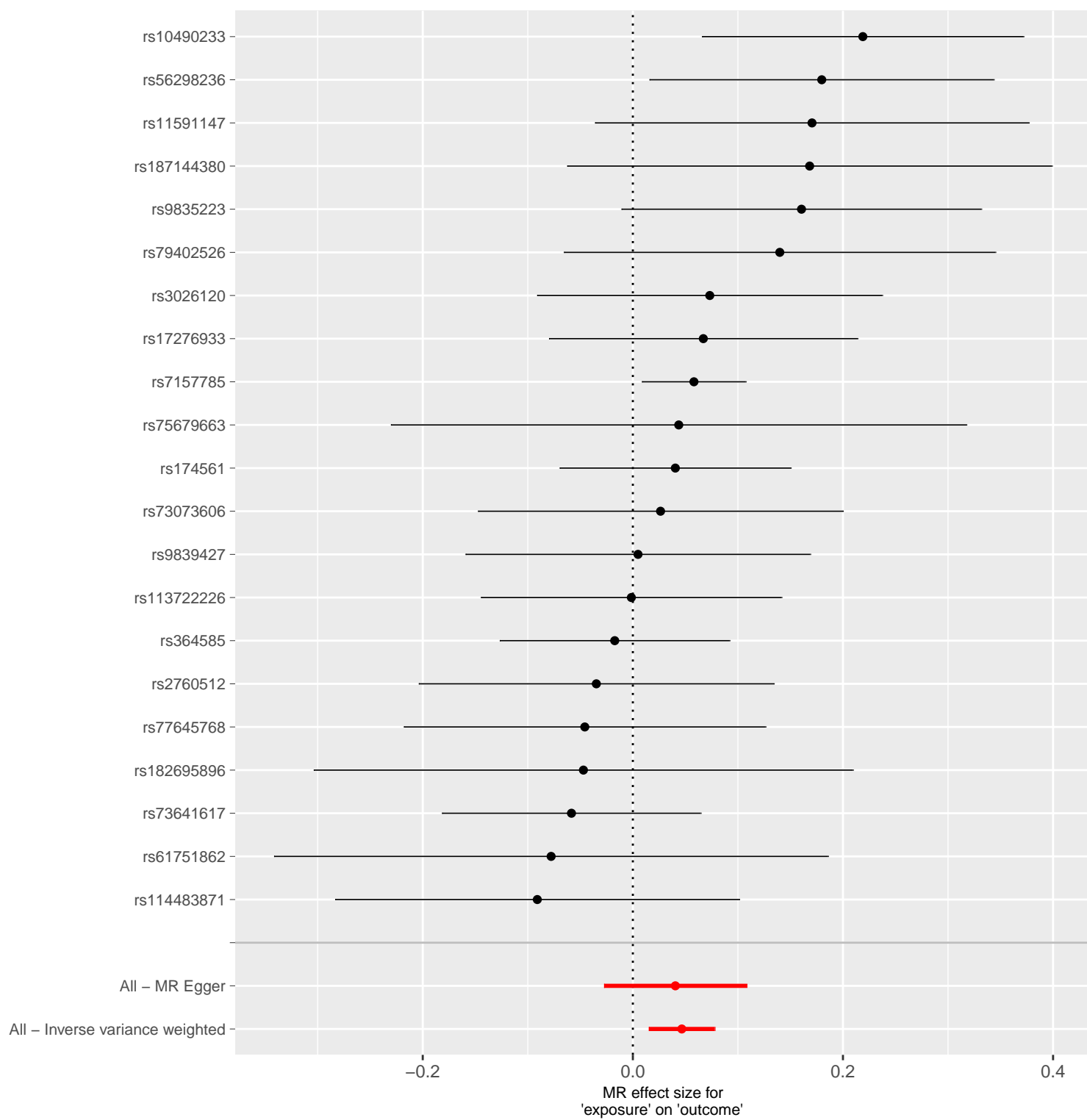

Supplement: Supplementary file 2 — Supplementary Material 2. [file 12944_2024_2103_MOESM2_ESM.zip › sFigure1∩╝êlipidomes-BC∩╝ë/GCST90277377/forest.pdf]

# MR Method

- Inverse variance weighted
- MR Egger

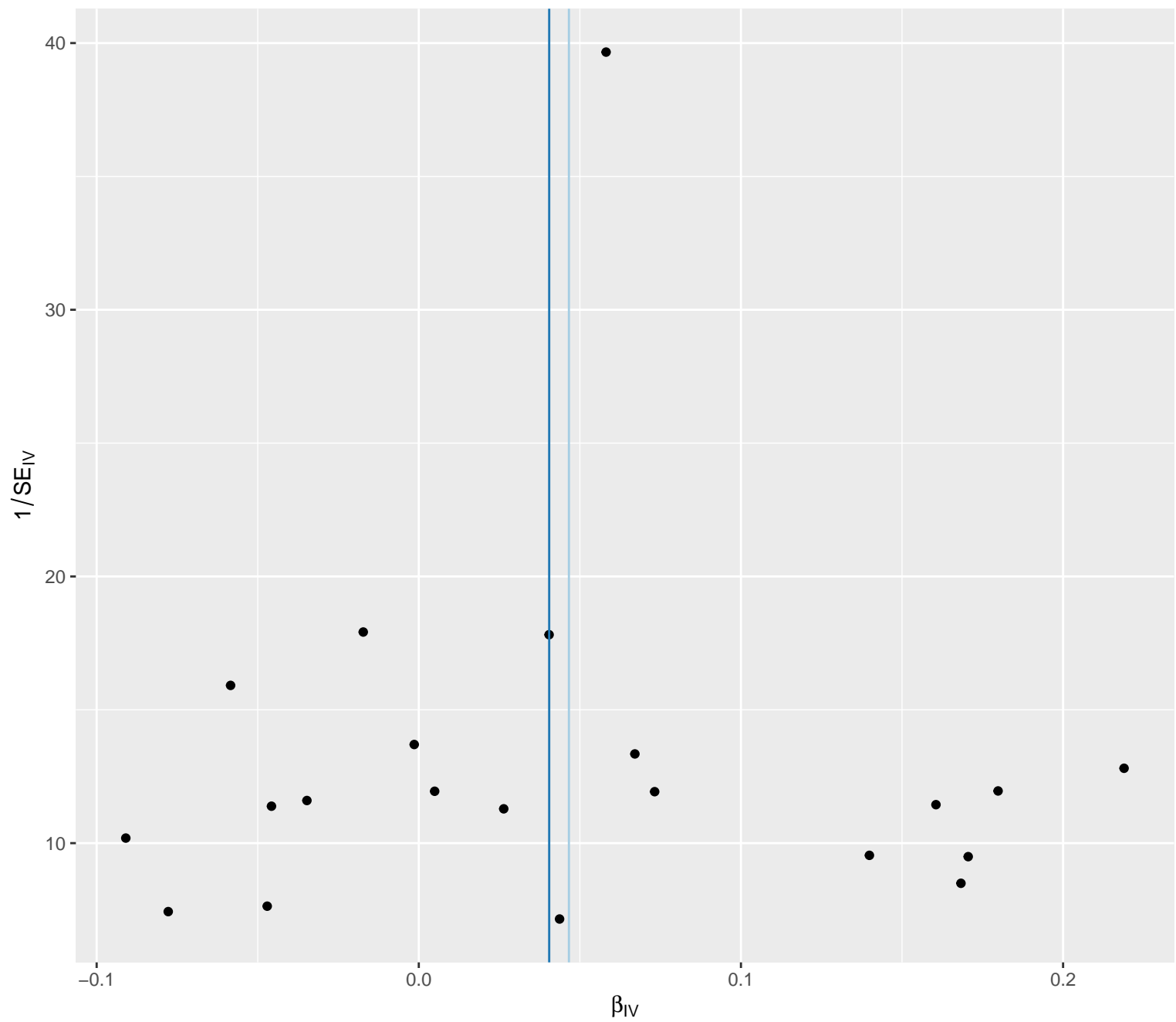

Supplement: Supplementary file 2 — Supplementary Material 2. [file 12944_2024_2103_MOESM2_ESM.zip › sFigure1∩╝êlipidomes-BC∩╝ë/GCST90277377/funnelplot.pdf]

# MR Test

- Inverse variance weighted
- MR Egger
- Simple mode
- Weighted median
- Weighted mode

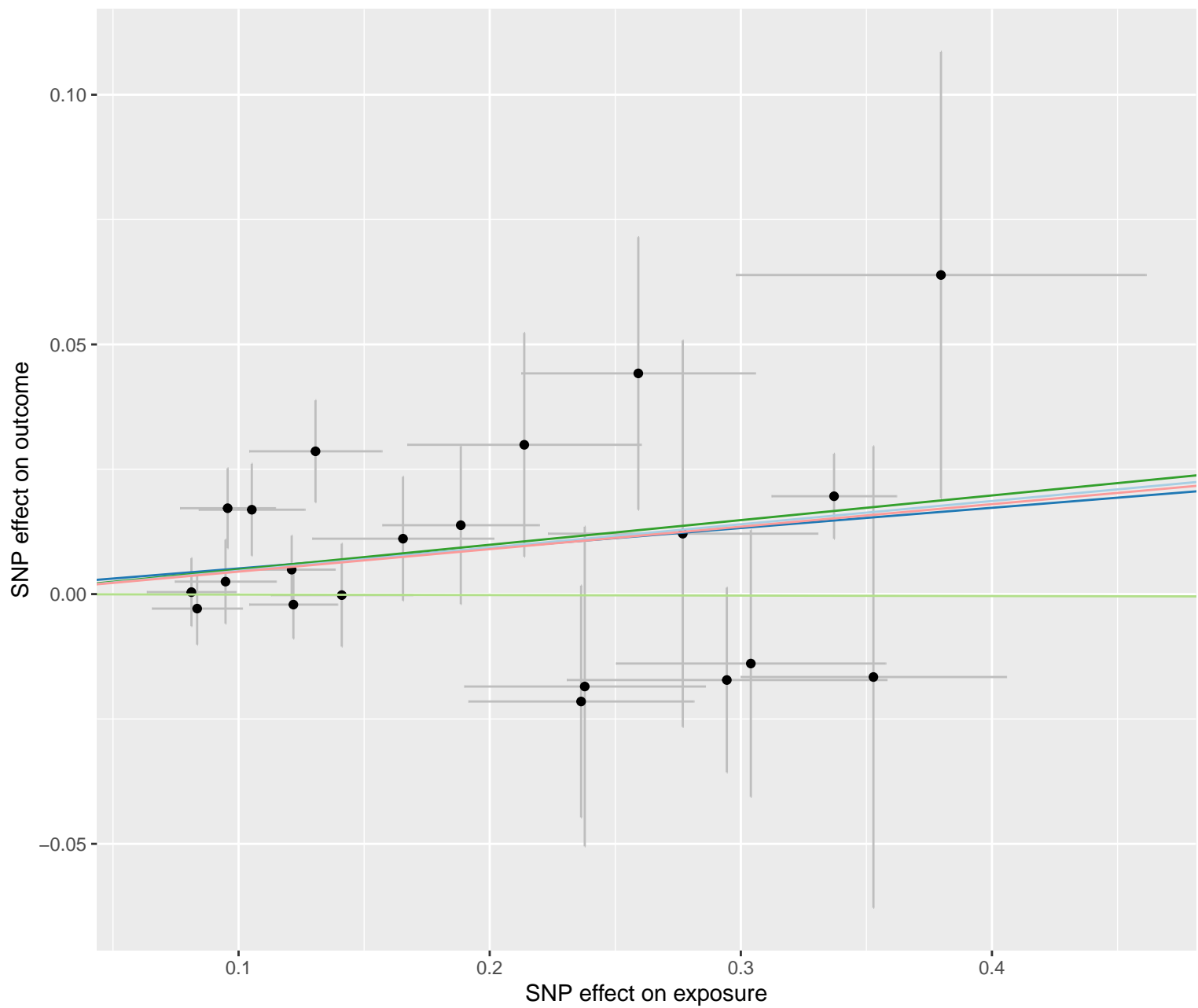

Supplement: Supplementary file 2 — Supplementary Material 2. [file 12944_2024_2103_MOESM2_ESM.zip › sFigure1∩╝êlipidomes-BC∩╝ë/GCST90277377/scatter.pdf]

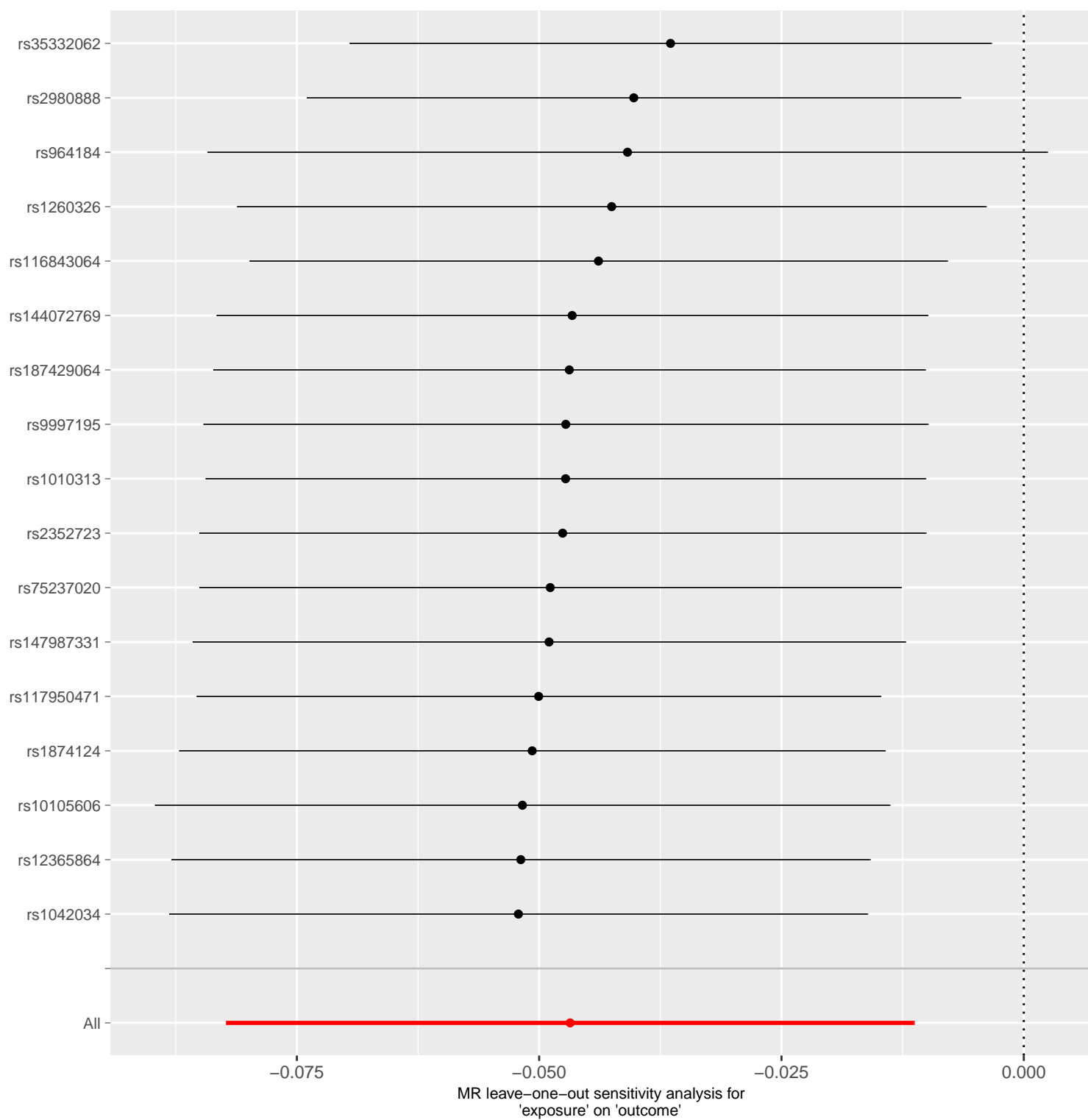

Supplement: Supplementary file 2 — Supplementary Material 2. [file 12944_2024_2103_MOESM2_ESM.zip › sFigure1∩╝êlipidomes-BC∩╝ë/GCST90277397/sensitivity-analysis.pdf]

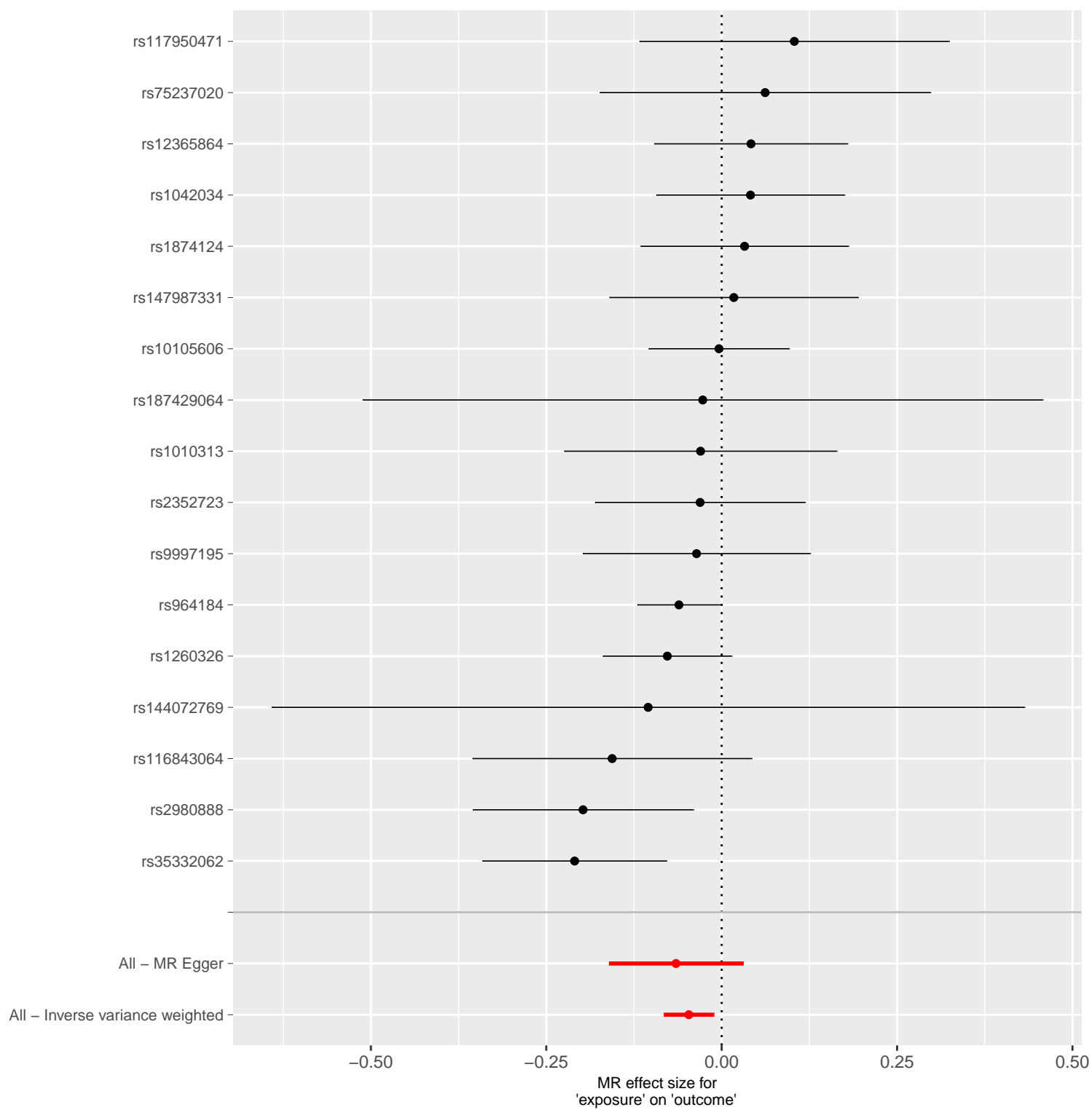

Supplement: Supplementary file 2 — Supplementary Material 2. [file 12944_2024_2103_MOESM2_ESM.zip › sFigure1∩╝êlipidomes-BC∩╝ë/GCST90277397/forest.pdf]

# MR Method

- Inverse variance weighted
- MR Egger

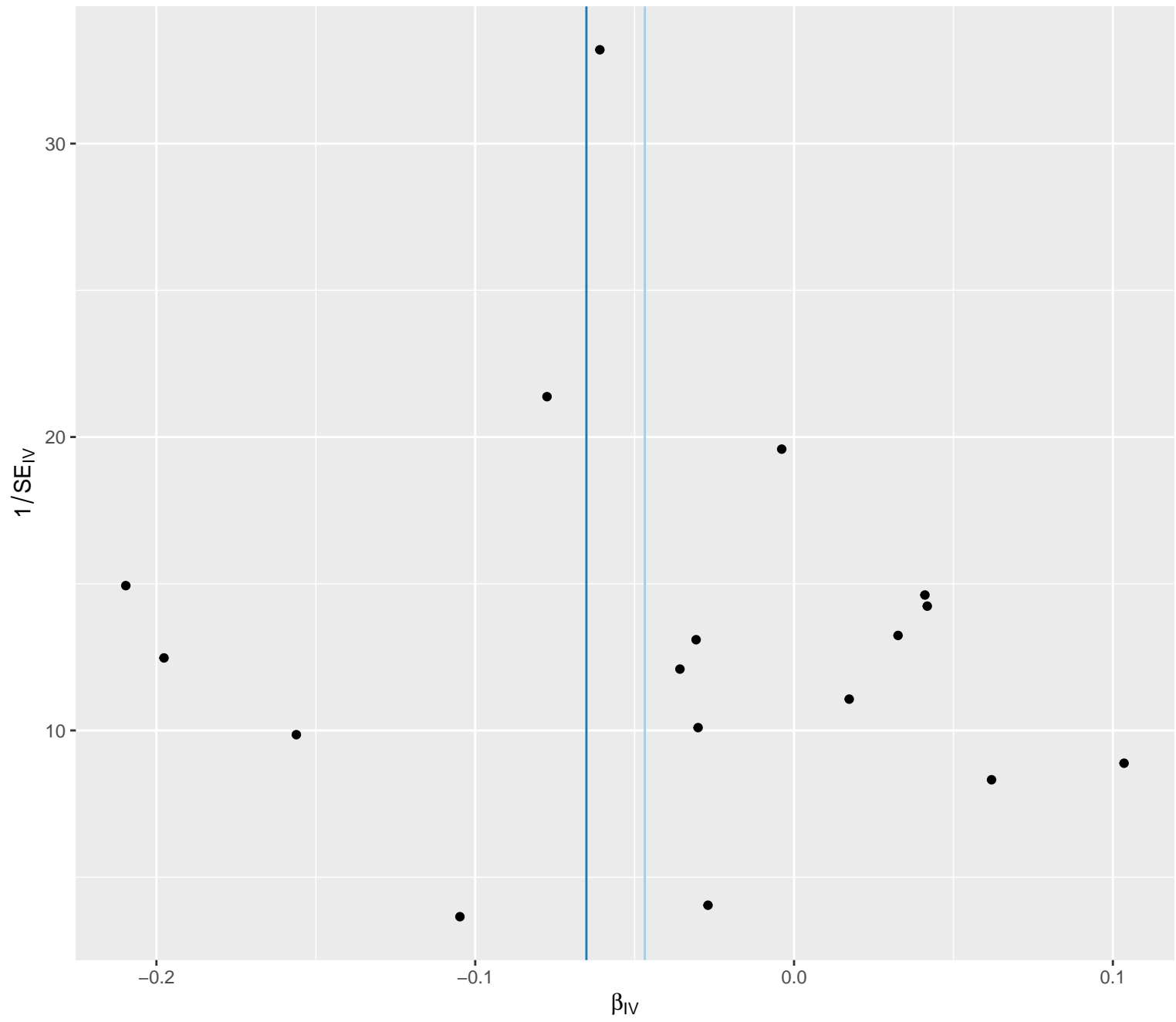

Supplement: Supplementary file 2 — Supplementary Material 2. [file 12944_2024_2103_MOESM2_ESM.zip › sFigure1∩╝êlipidomes-BC∩╝ë/GCST90277397/funnelplot.pdf]

# MR Test

- Inverse variance weighted
- MR Egger
- Simple mode
- Weighted median
- Weighted mode

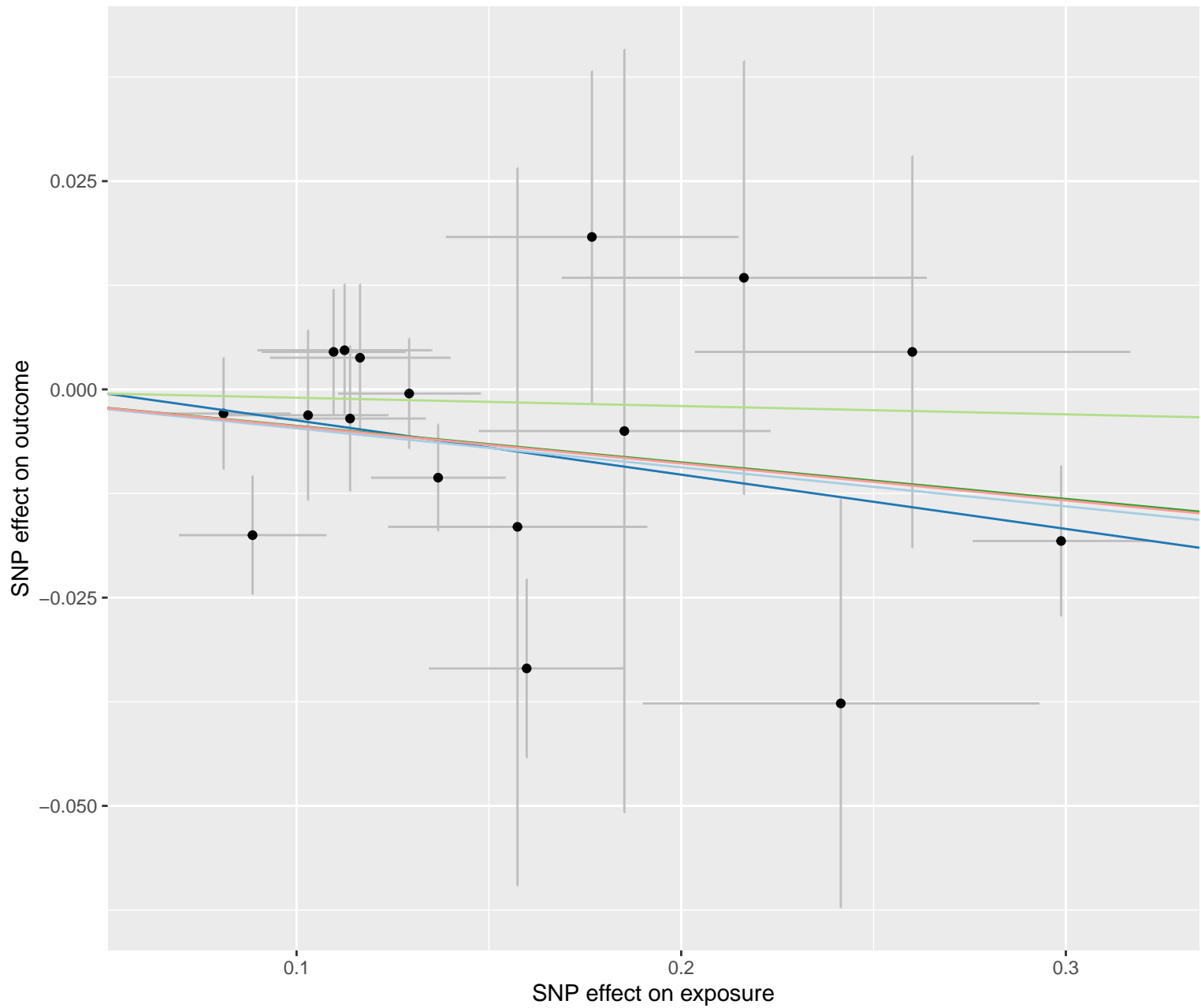

Supplement: Supplementary file 2 — Supplementary Material 2. [file 12944_2024_2103_MOESM2_ESM.zip › sFigure1∩╝êlipidomes-BC∩╝ë/GCST90277397/scatter.pdf]

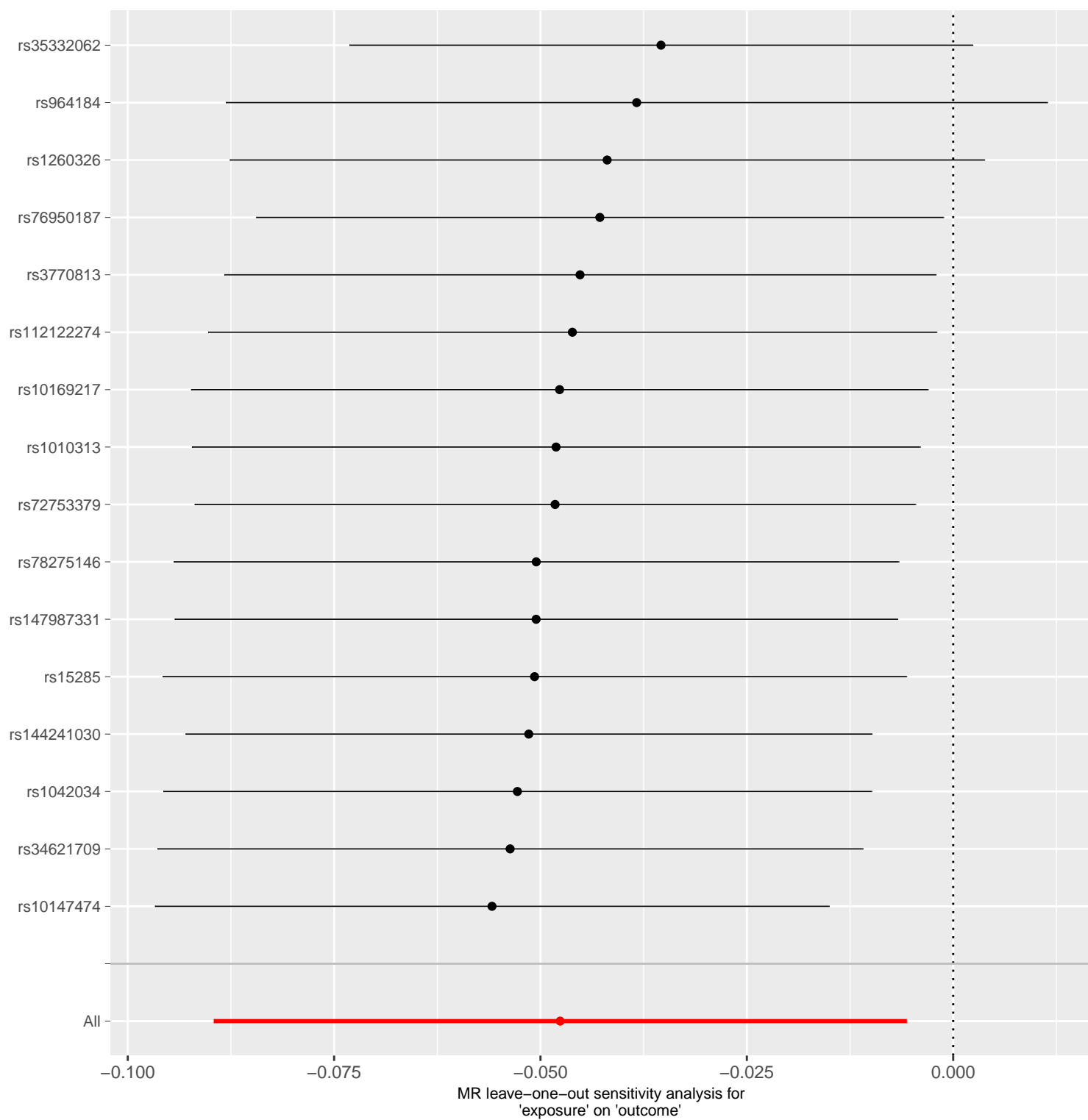

Supplement: Supplementary file 2 — Supplementary Material 2. [file 12944_2024_2103_MOESM2_ESM.zip › sFigure1∩╝êlipidomes-BC∩╝ë/GCST90277396/sensitivity-analysis.pdf]

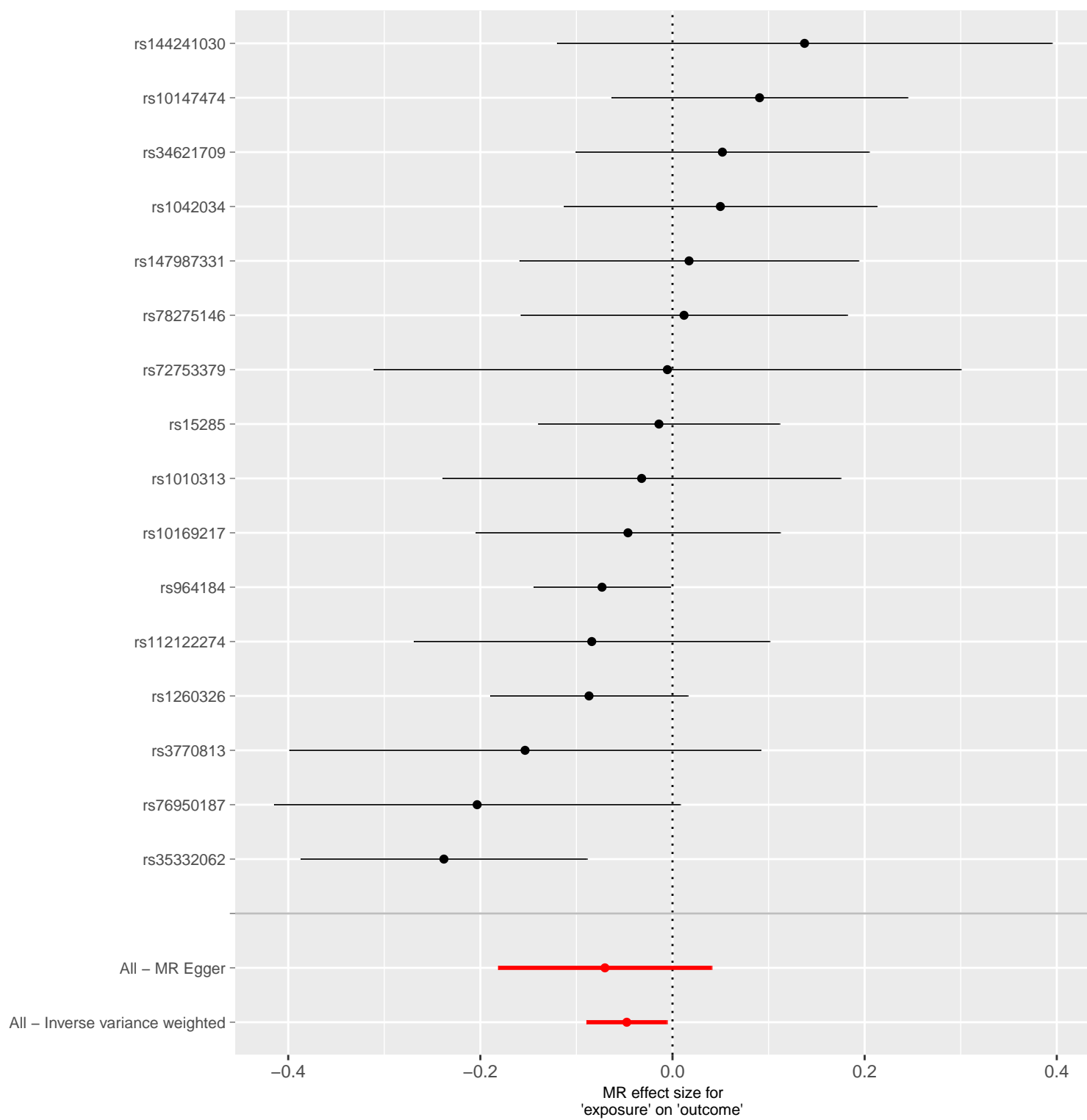

Supplement: Supplementary file 2 — Supplementary Material 2. [file 12944_2024_2103_MOESM2_ESM.zip › sFigure1∩╝êlipidomes-BC∩╝ë/GCST90277396/forest.pdf]

# MR Method

- Inverse variance weighted
- MR Egger

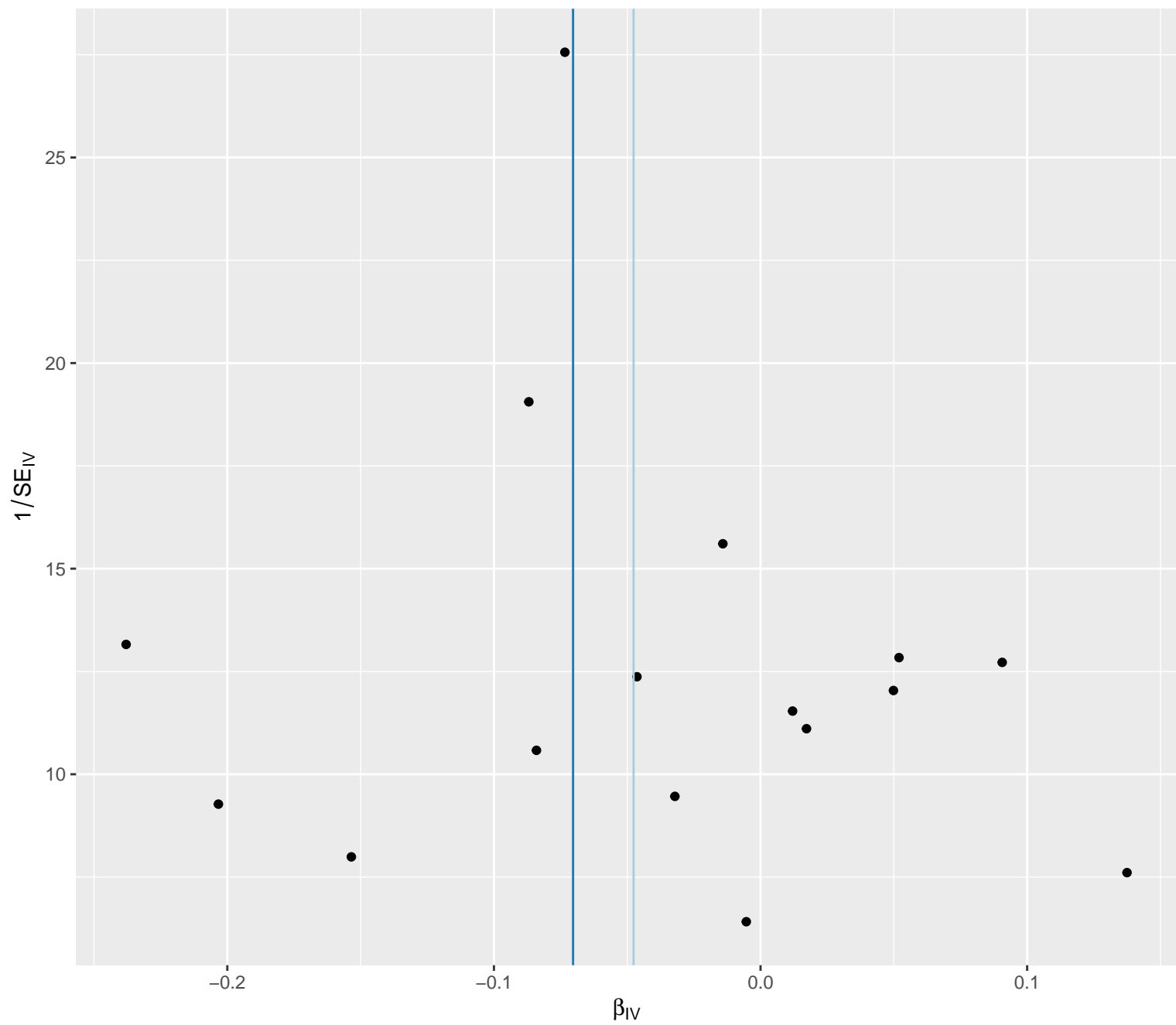

Supplement: Supplementary file 2 — Supplementary Material 2. [file 12944_2024_2103_MOESM2_ESM.zip › sFigure1∩╝êlipidomes-BC∩╝ë/GCST90277396/funnelplot.pdf]

# MR Test

- Inverse variance weighted
- MR Egger
- Simple mode
- Weighted median
- Weighted mode

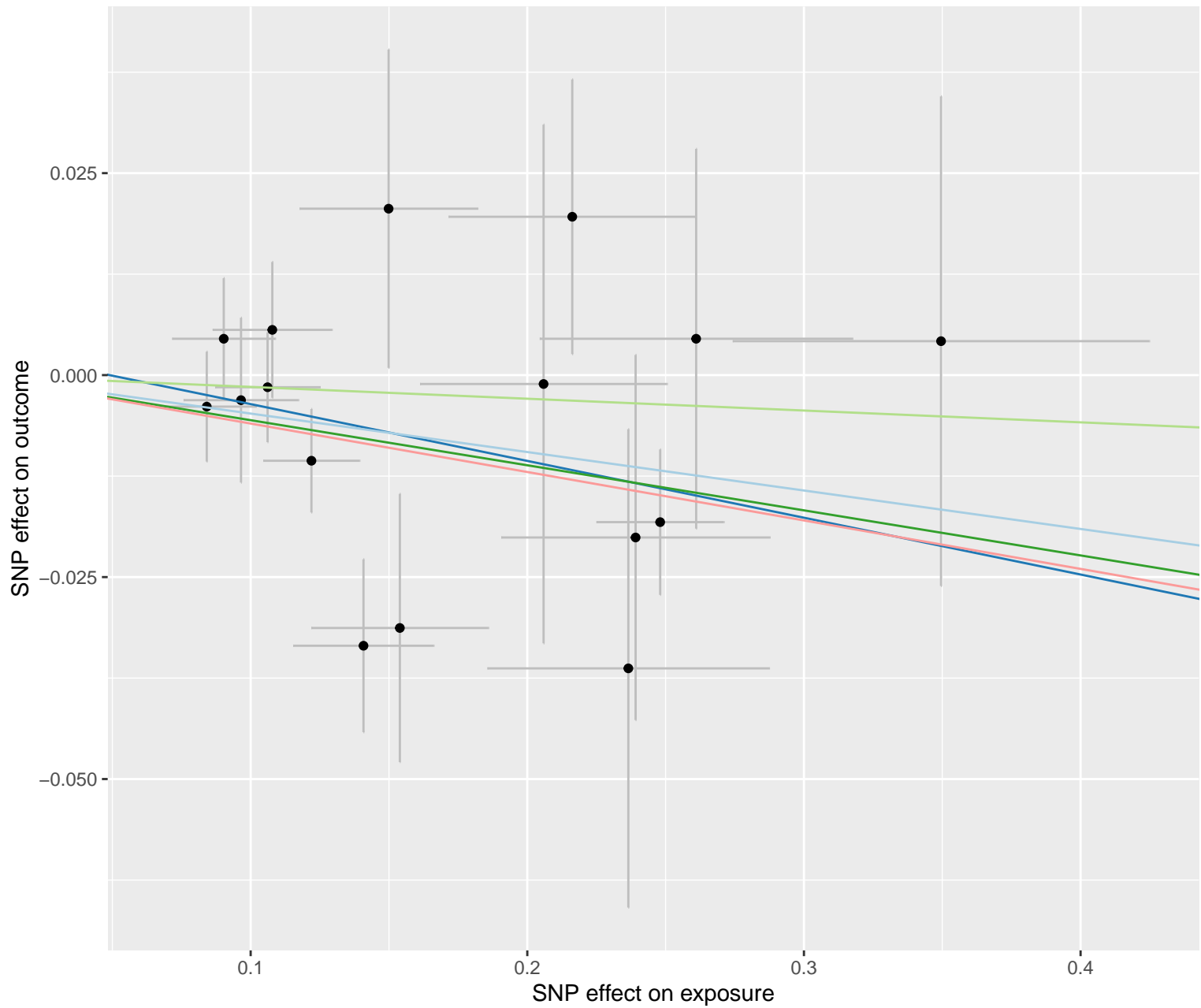

Supplement: Supplementary file 2 — Supplementary Material 2. [file 12944_2024_2103_MOESM2_ESM.zip › sFigure1∩╝êlipidomes-BC∩╝ë/GCST90277396/scatter.pdf]

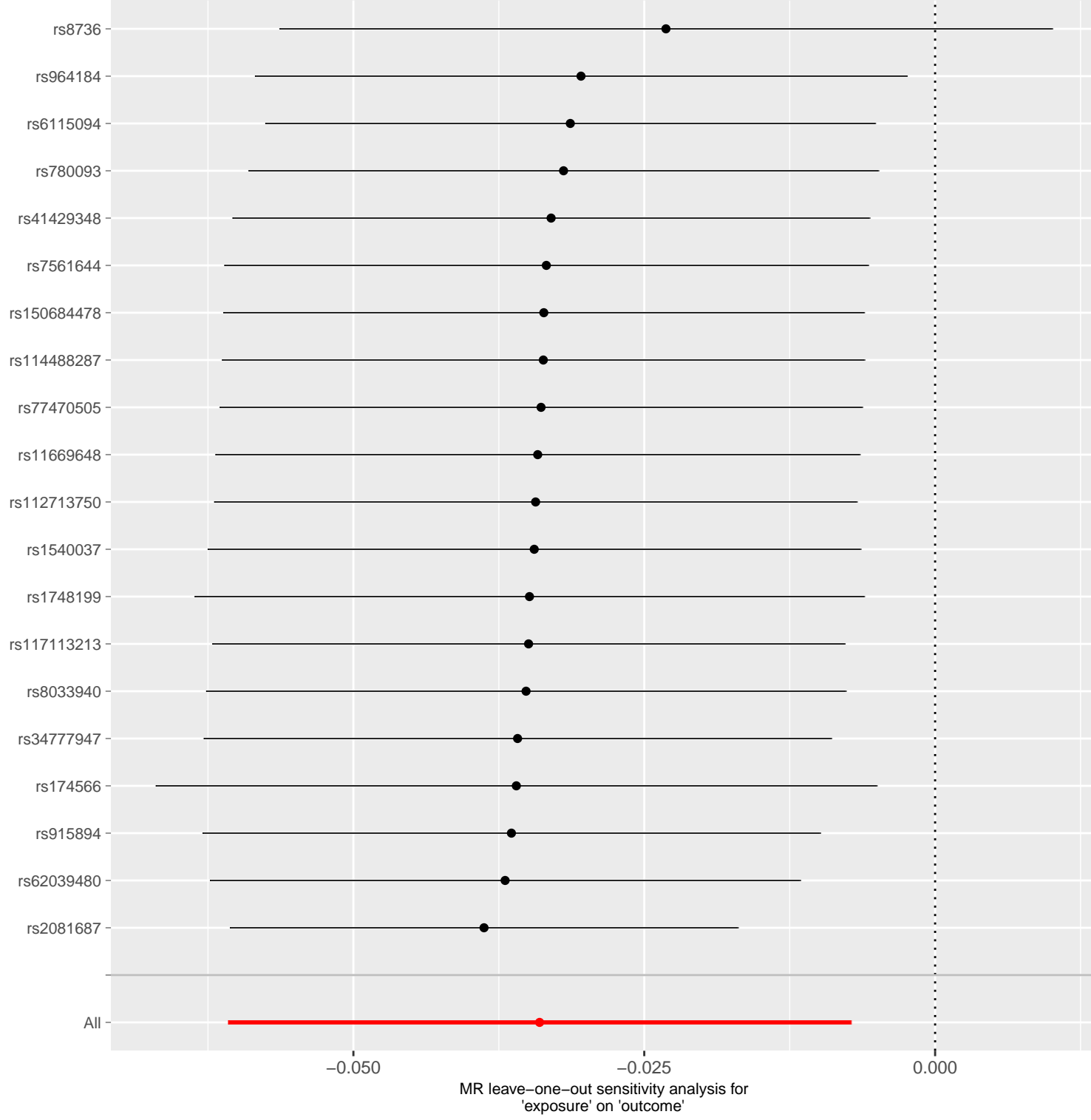

Supplement: Supplementary file 2 — Supplementary Material 2. [file 12944_2024_2103_MOESM2_ESM.zip › sFigure1∩╝êlipidomes-BC∩╝ë/GCST90277362/sensitivity-analysis.pdf]

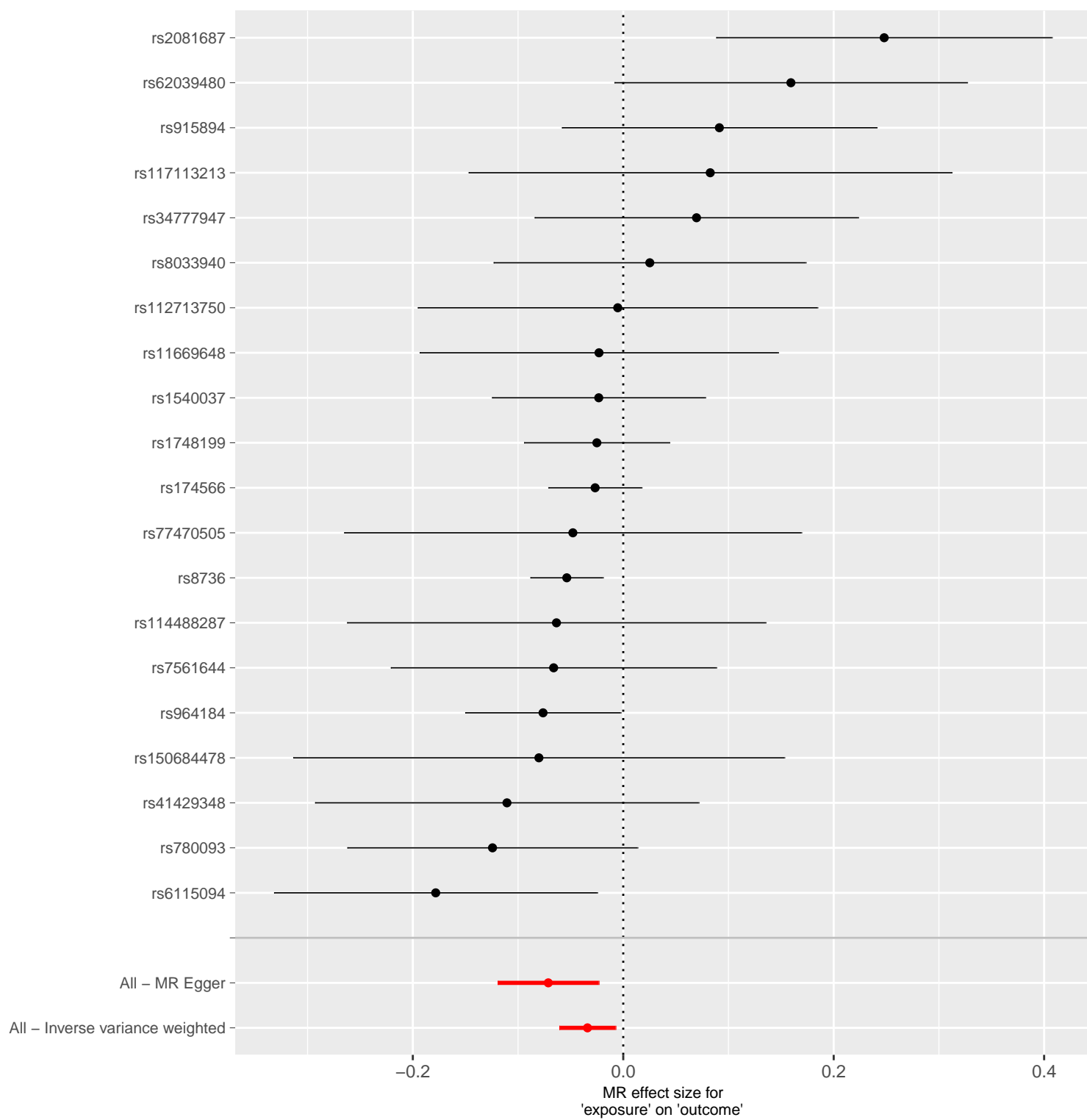

Supplement: Supplementary file 2 — Supplementary Material 2. [file 12944_2024_2103_MOESM2_ESM.zip › sFigure1∩╝êlipidomes-BC∩╝ë/GCST90277362/forest.pdf]

# MR Method

- Inverse variance weighted
- MR Egger

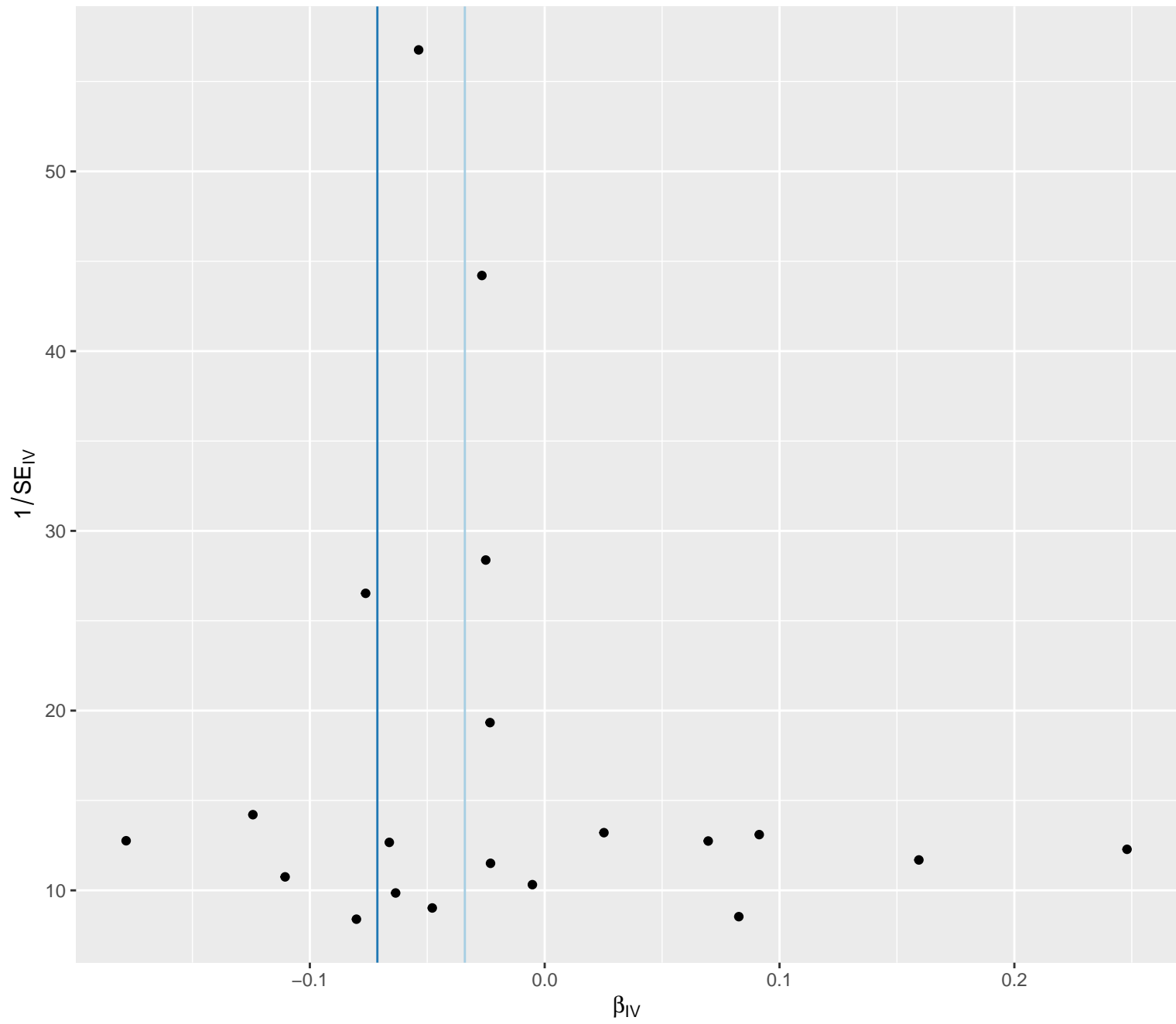

Supplement: Supplementary file 2 — Supplementary Material 2. [file 12944_2024_2103_MOESM2_ESM.zip › sFigure1∩╝êlipidomes-BC∩╝ë/GCST90277362/funnelplot.pdf]

# MR Test

- Inverse variance weighted
- MR Egger
- Simple mode
- Weighted median
- Weighted mode

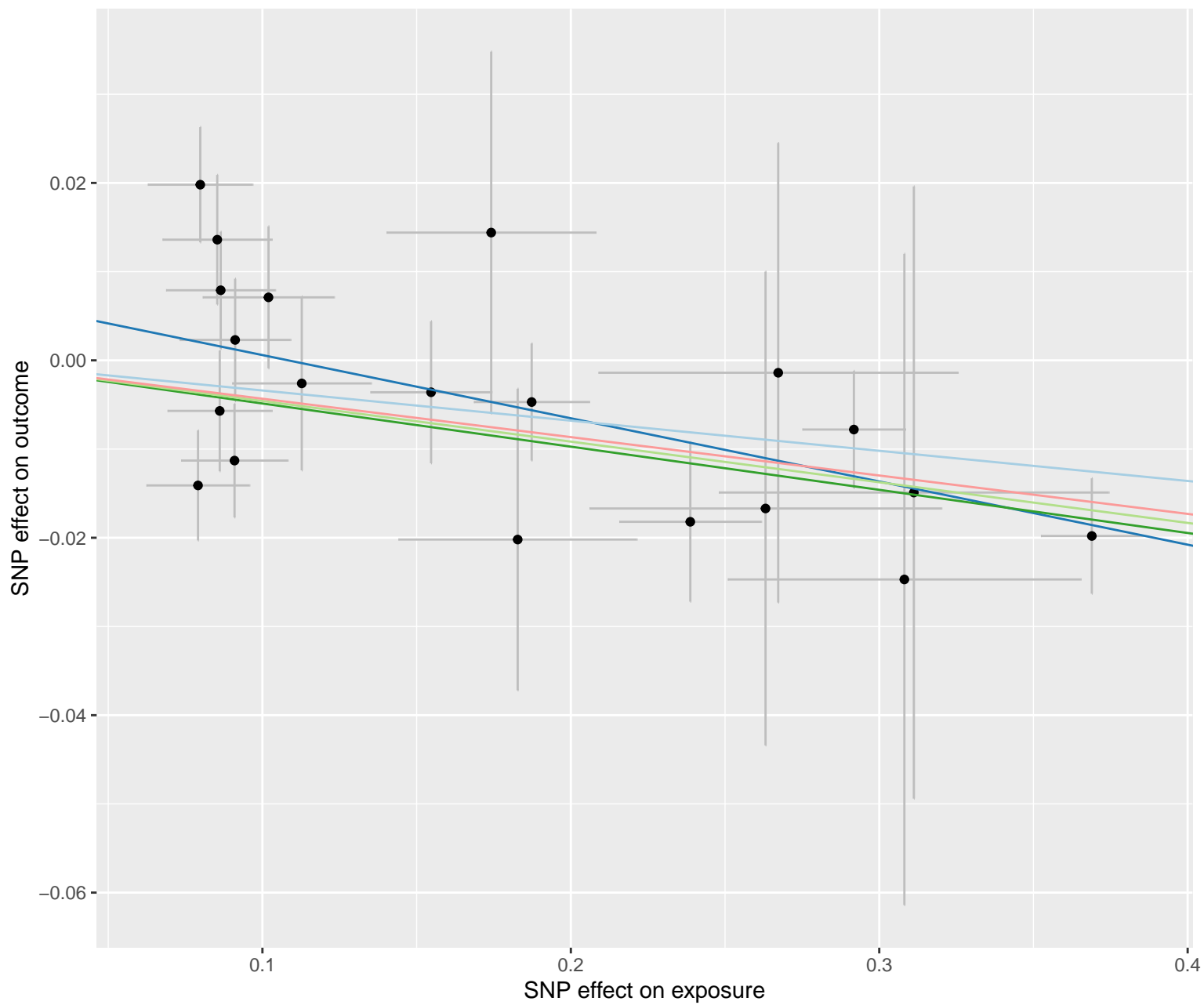

Supplement: Supplementary file 2 — Supplementary Material 2. [file 12944_2024_2103_MOESM2_ESM.zip › sFigure1∩╝êlipidomes-BC∩╝ë/GCST90277362/scatter.pdf]

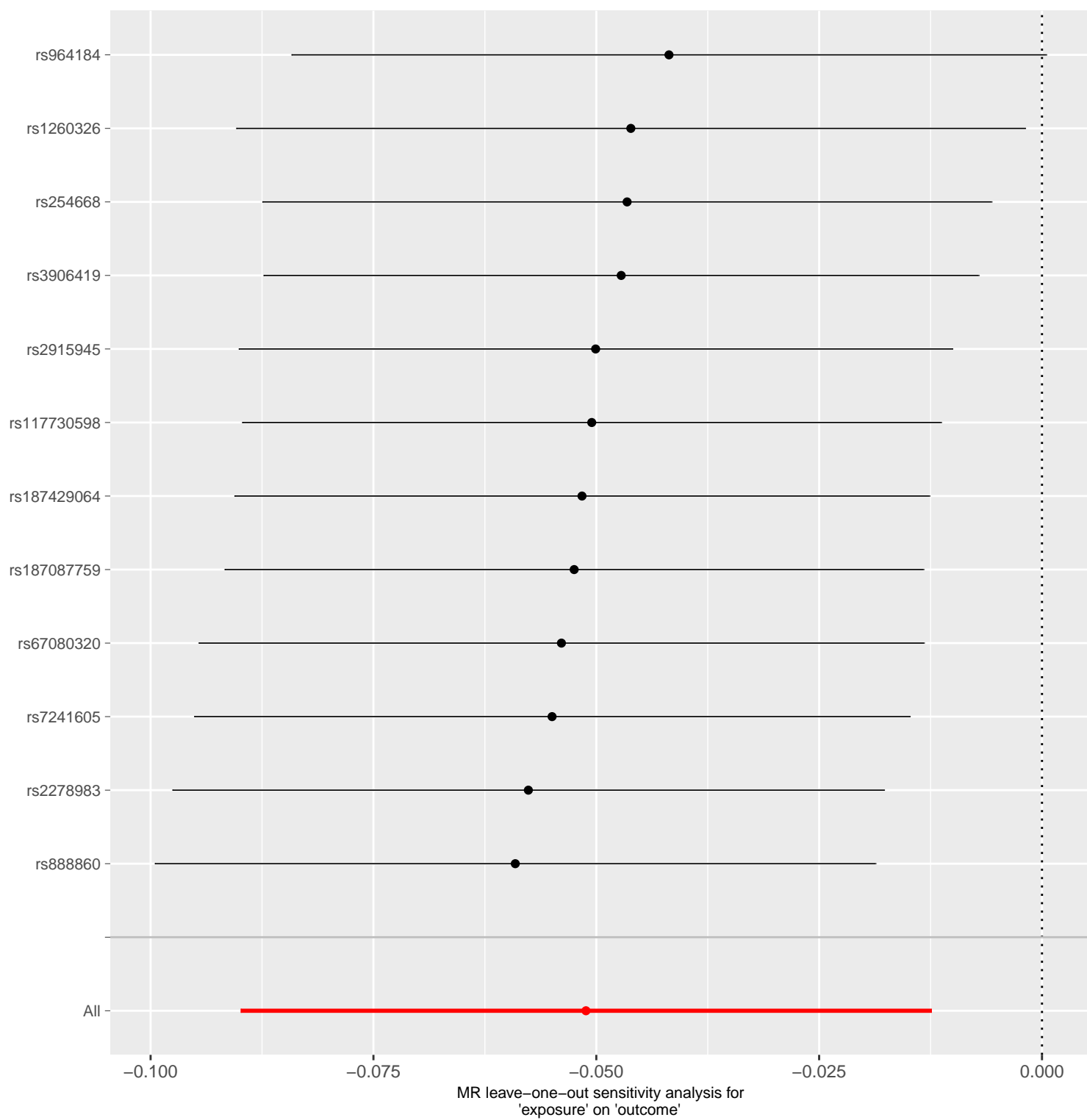

Supplement: Supplementary file 2 — Supplementary Material 2. [file 12944_2024_2103_MOESM2_ESM.zip › sFigure1∩╝êlipidomes-BC∩╝ë/GCST90277391/sensitivity-analysis.pdf]

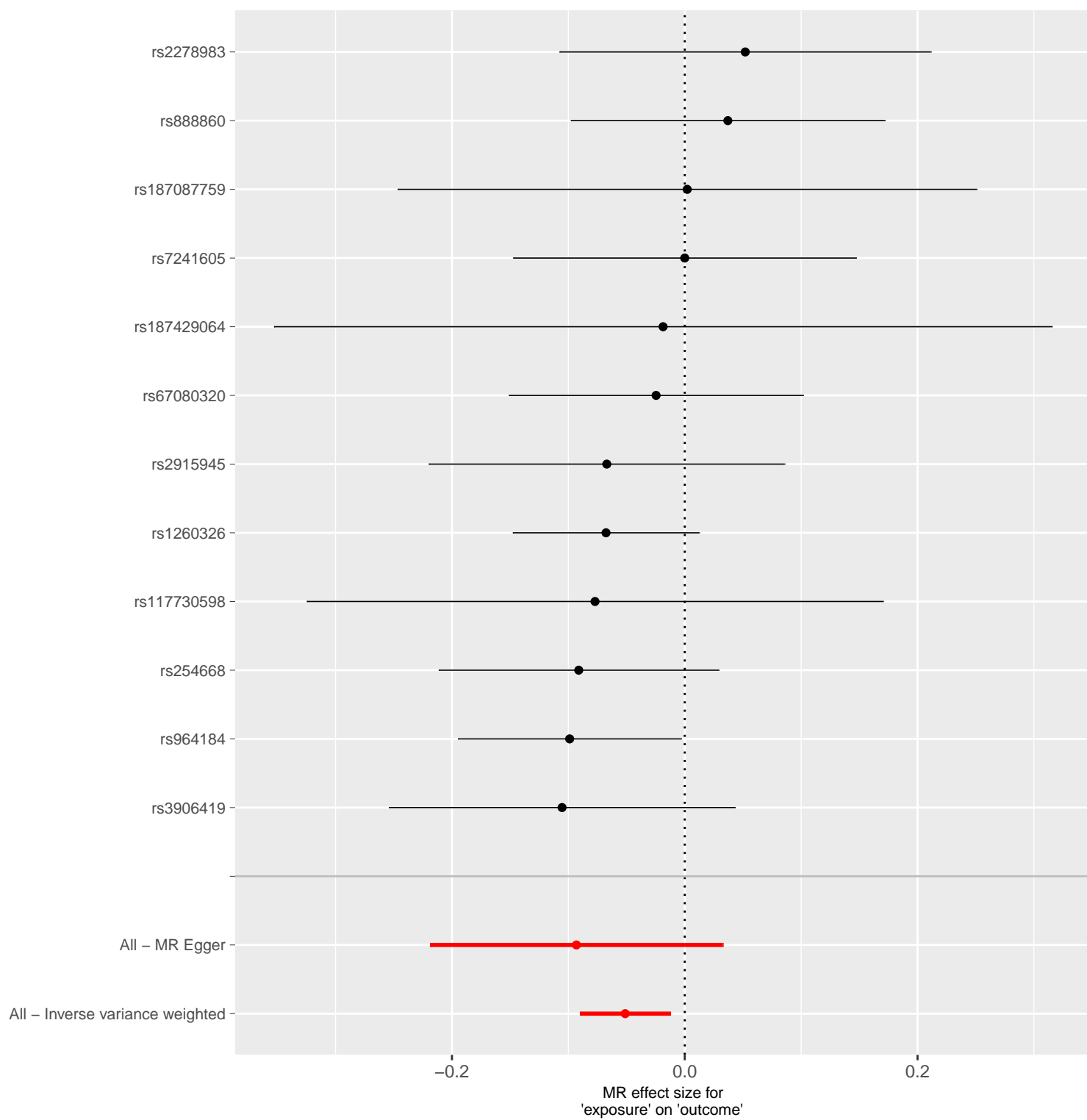

Supplement: Supplementary file 2 — Supplementary Material 2. [file 12944_2024_2103_MOESM2_ESM.zip › sFigure1∩╝êlipidomes-BC∩╝ë/GCST90277391/forest.pdf]

# MR Method

- Inverse variance weighted
- MR Egger

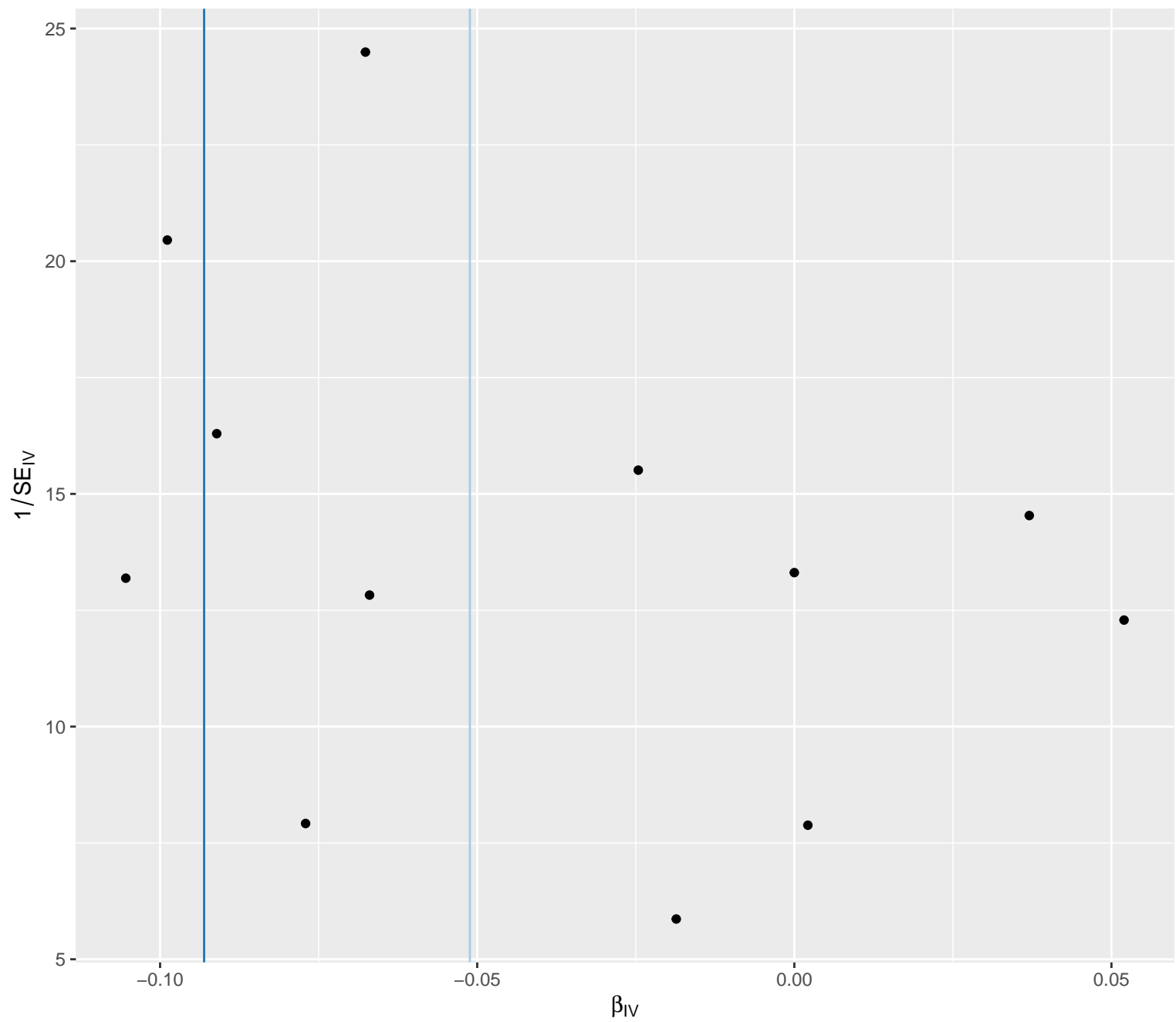

Supplement: Supplementary file 2 — Supplementary Material 2. [file 12944_2024_2103_MOESM2_ESM.zip › sFigure1∩╝êlipidomes-BC∩╝ë/GCST90277391/funnelplot.pdf]

# MR Test

- Inverse variance weighted
- MR Egger
- Simple mode
- Weighted median
- Weighted mode

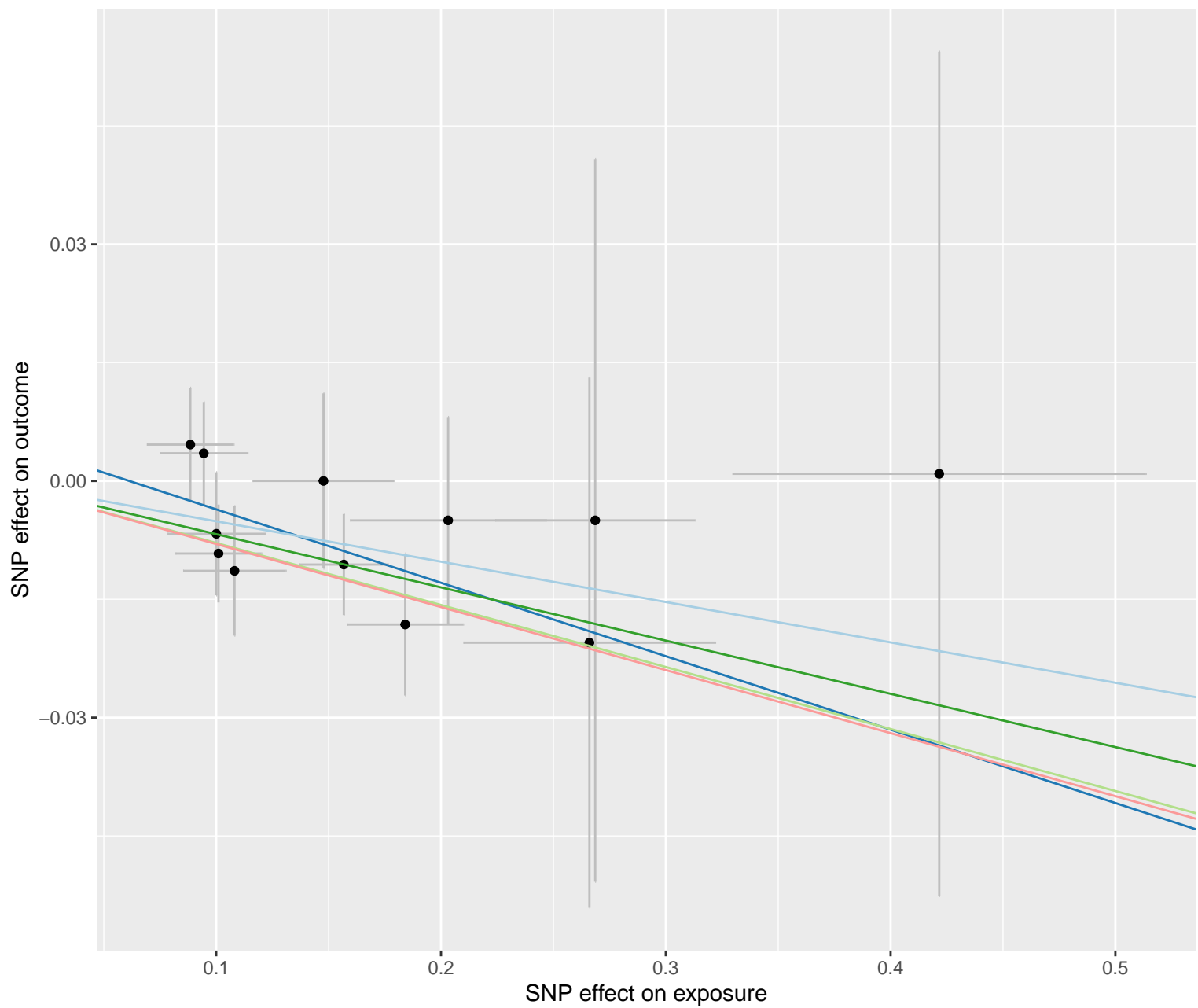

Supplement: Supplementary file 2 — Supplementary Material 2. [file 12944_2024_2103_MOESM2_ESM.zip › sFigure1∩╝êlipidomes-BC∩╝ë/GCST90277391/scatter.pdf]

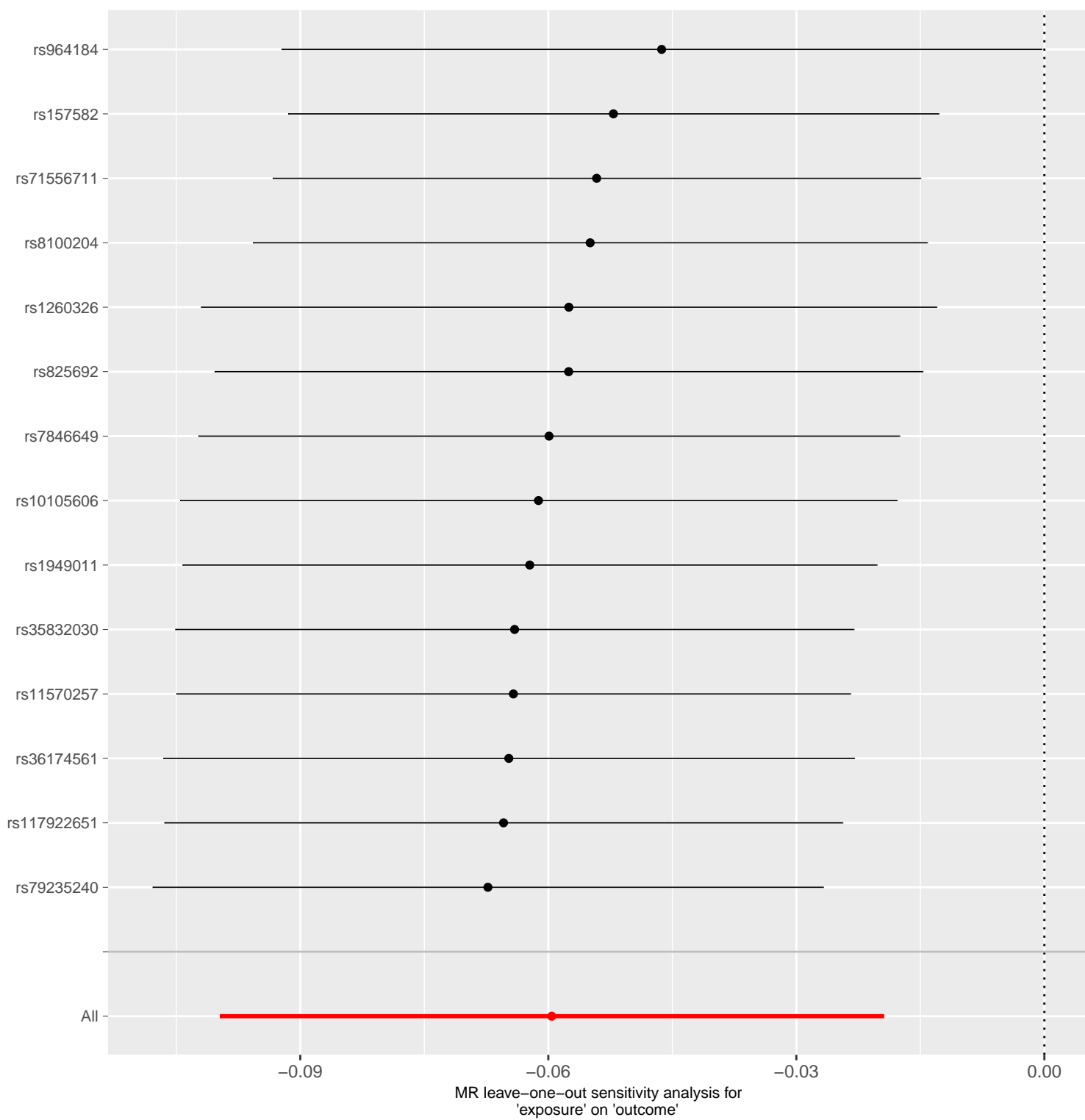

Supplement: Supplementary file 3 — Supplementary Material 3. [file 12944_2024_2103_MOESM3_ESM.zip › sFigure2∩╝êlipidomes-ER+BC∩╝ë/GCST90277403/sensitivity-analysis.pdf]

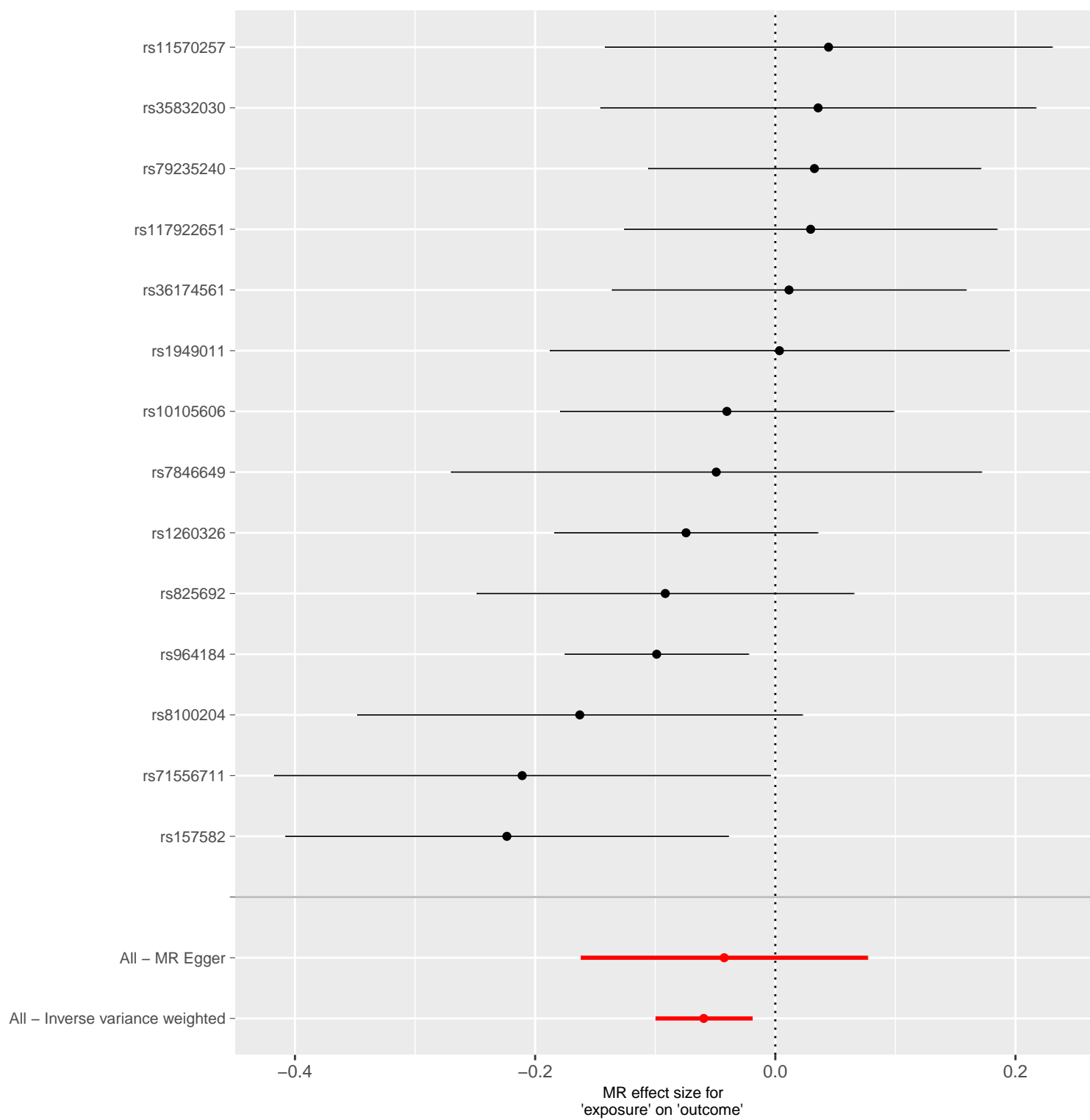

Supplement: Supplementary file 3 — Supplementary Material 3. [file 12944_2024_2103_MOESM3_ESM.zip › sFigure2∩╝êlipidomes-ER+BC∩╝ë/GCST90277403/forest.pdf]

# MR Method

- Inverse variance weighted
- MR Egger

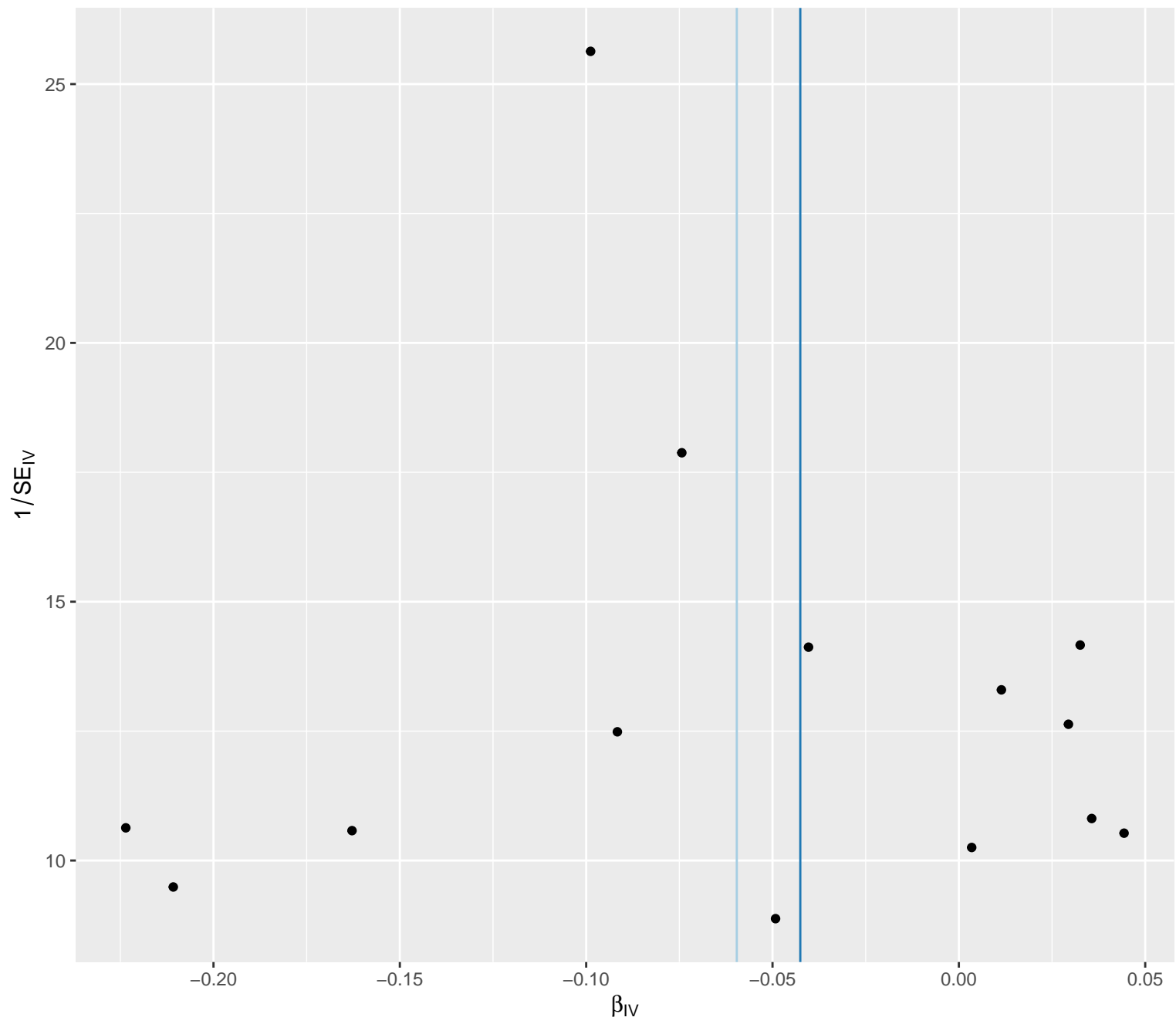

Supplement: Supplementary file 3 — Supplementary Material 3. [file 12944_2024_2103_MOESM3_ESM.zip › sFigure2∩╝êlipidomes-ER+BC∩╝ë/GCST90277403/funnelplot.pdf]

# MR Test

- Inverse variance weighted
- MR Egger
- Simple mode
- Weighted median
- Weighted mode

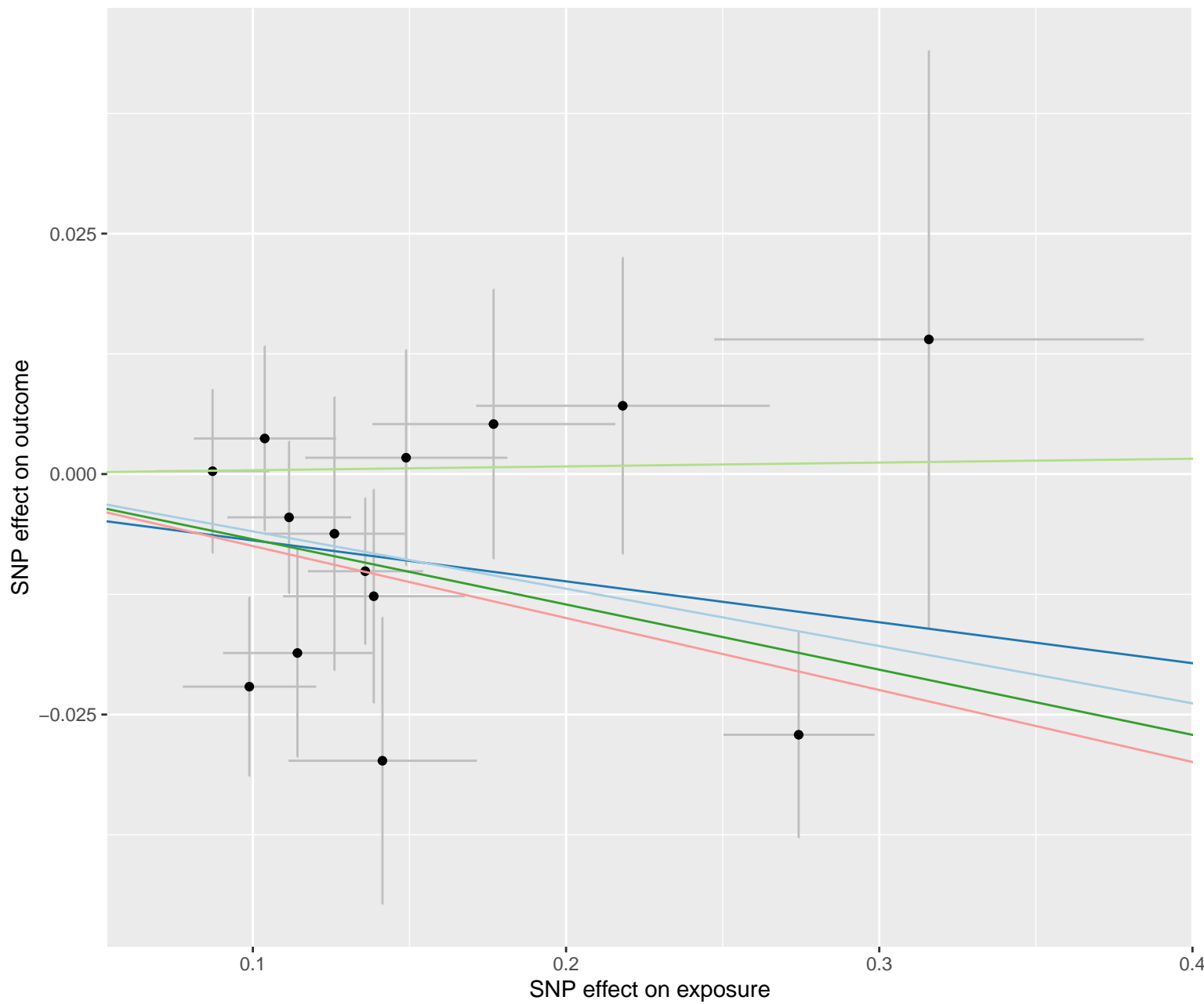

Supplement: Supplementary file 3 — Supplementary Material 3. [file 12944_2024_2103_MOESM3_ESM.zip › sFigure2∩╝êlipidomes-ER+BC∩╝ë/GCST90277403/scatter.pdf]

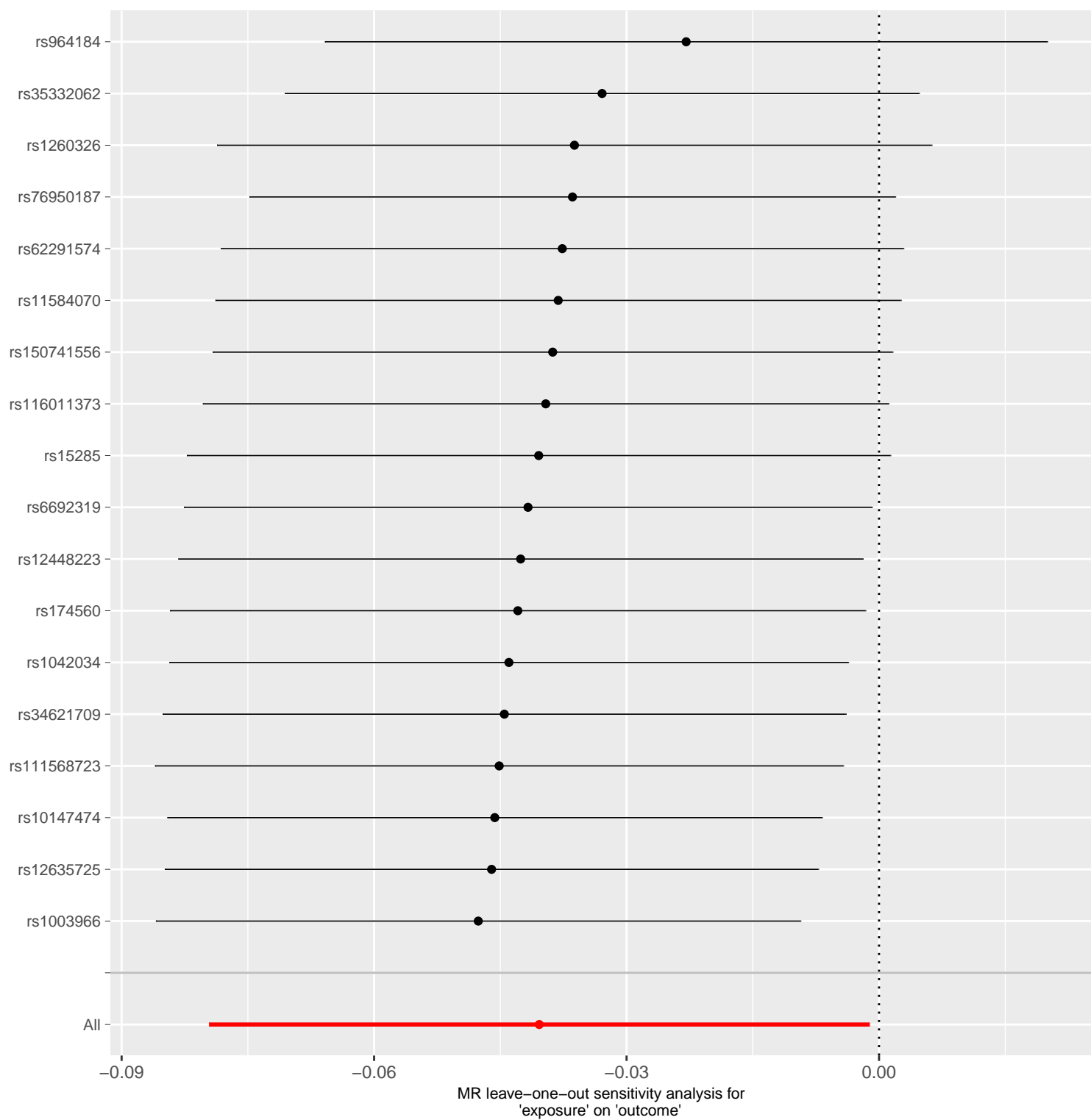

Supplement: Supplementary file 3 — Supplementary Material 3. [file 12944_2024_2103_MOESM3_ESM.zip › sFigure2∩╝êlipidomes-ER+BC∩╝ë/GCST90277402/sensitivity-analysis.pdf]

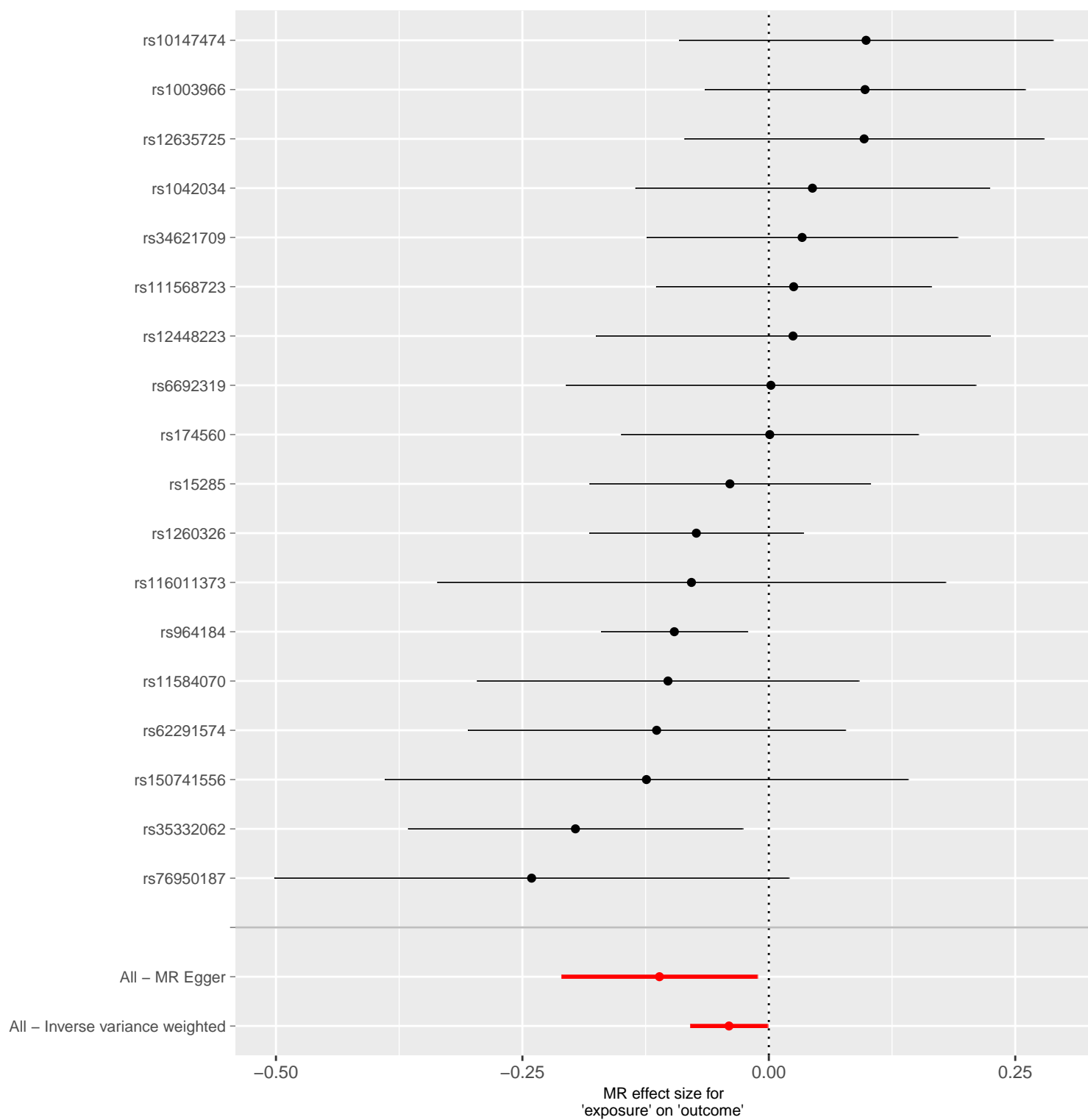

Supplement: Supplementary file 3 — Supplementary Material 3. [file 12944_2024_2103_MOESM3_ESM.zip › sFigure2∩╝êlipidomes-ER+BC∩╝ë/GCST90277402/forest.pdf]

# MR Method

- Inverse variance weighted
- MR Egger

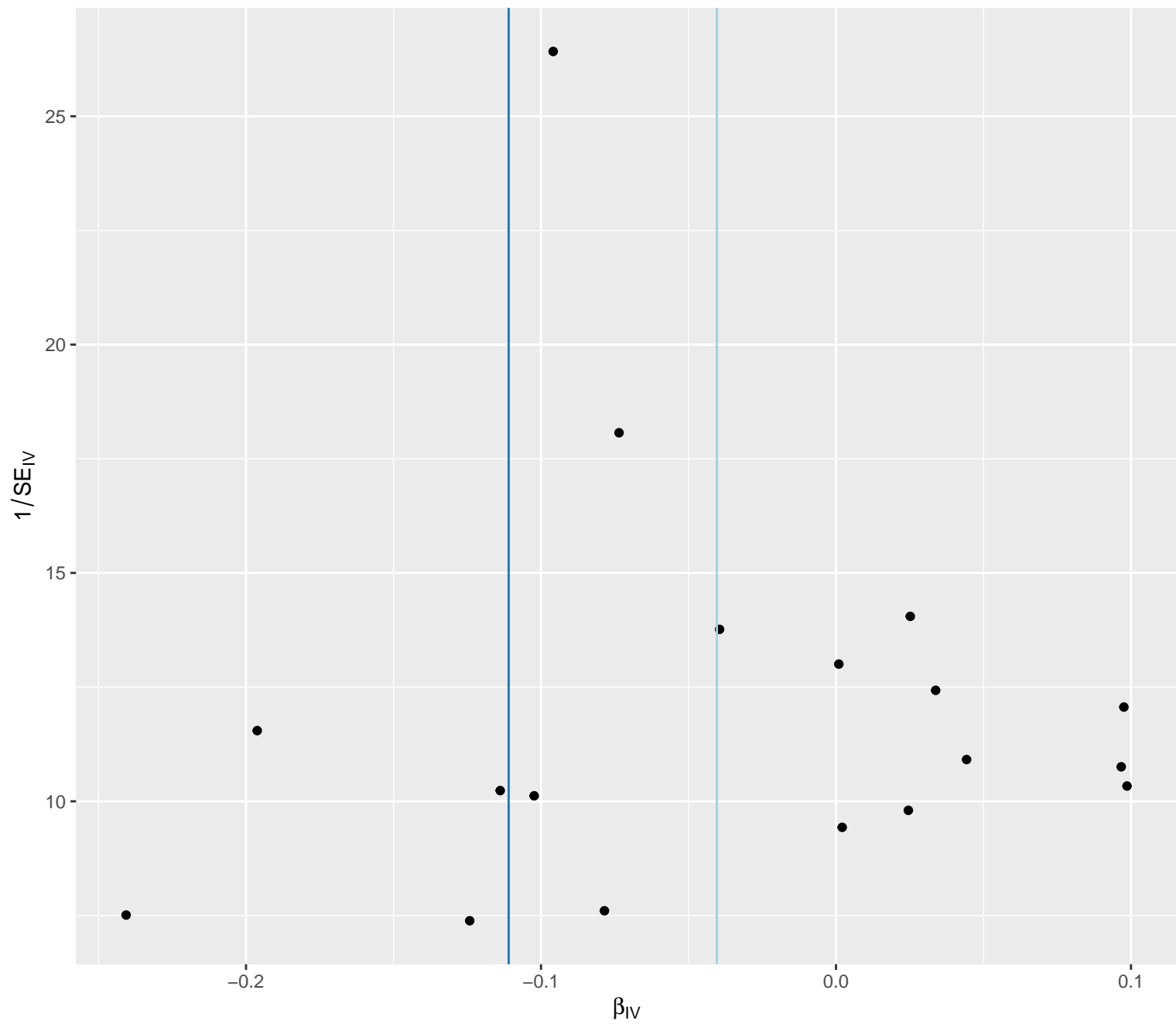

Supplement: Supplementary file 3 — Supplementary Material 3. [file 12944_2024_2103_MOESM3_ESM.zip › sFigure2∩╝êlipidomes-ER+BC∩╝ë/GCST90277402/funnelplot.pdf]

# MR Test

- Inverse variance weighted
- MR Egger
- Simple mode
- Weighted median
- Weighted mode

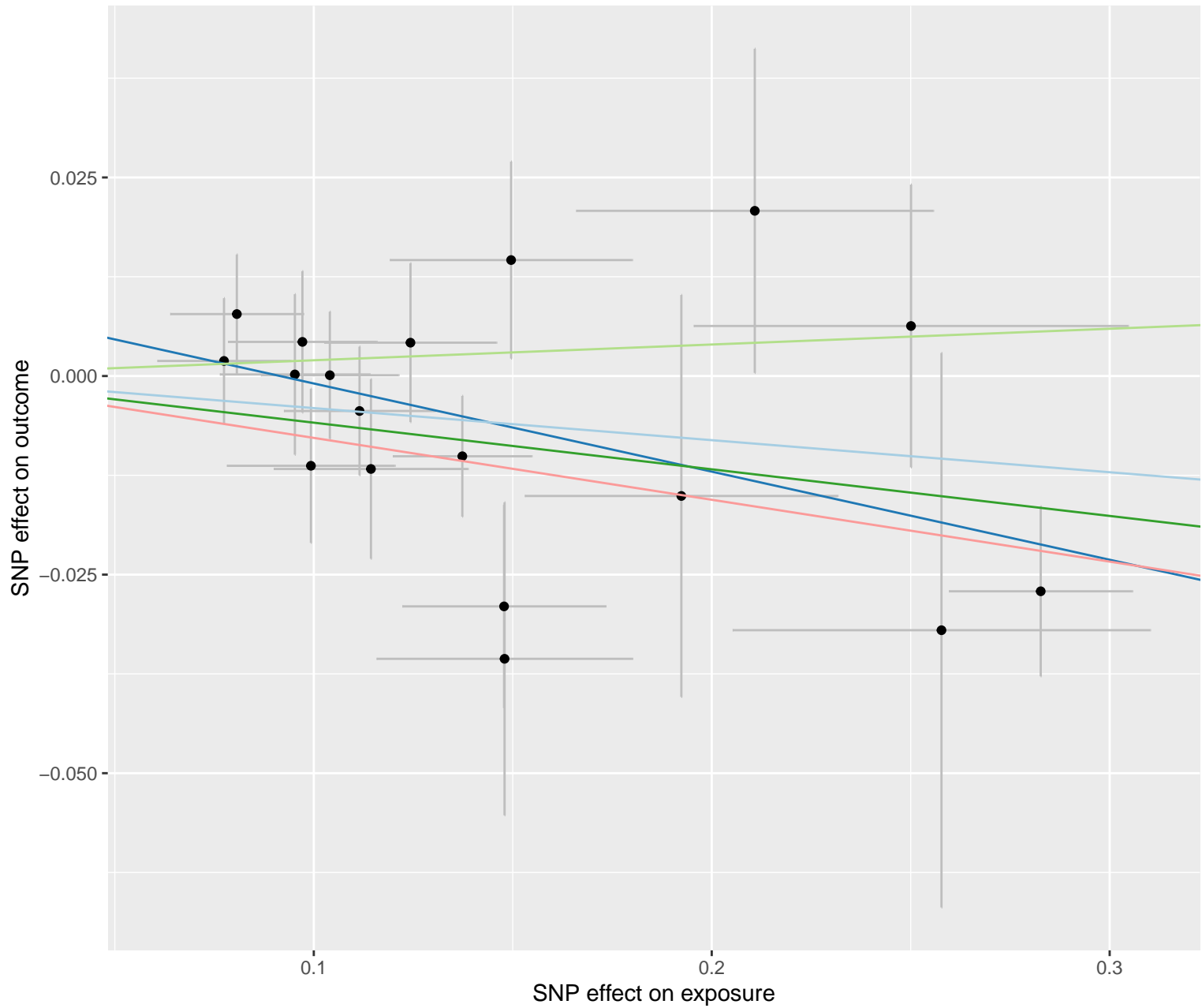

Supplement: Supplementary file 3 — Supplementary Material 3. [file 12944_2024_2103_MOESM3_ESM.zip › sFigure2∩╝êlipidomes-ER+BC∩╝ë/GCST90277402/scatter.pdf]

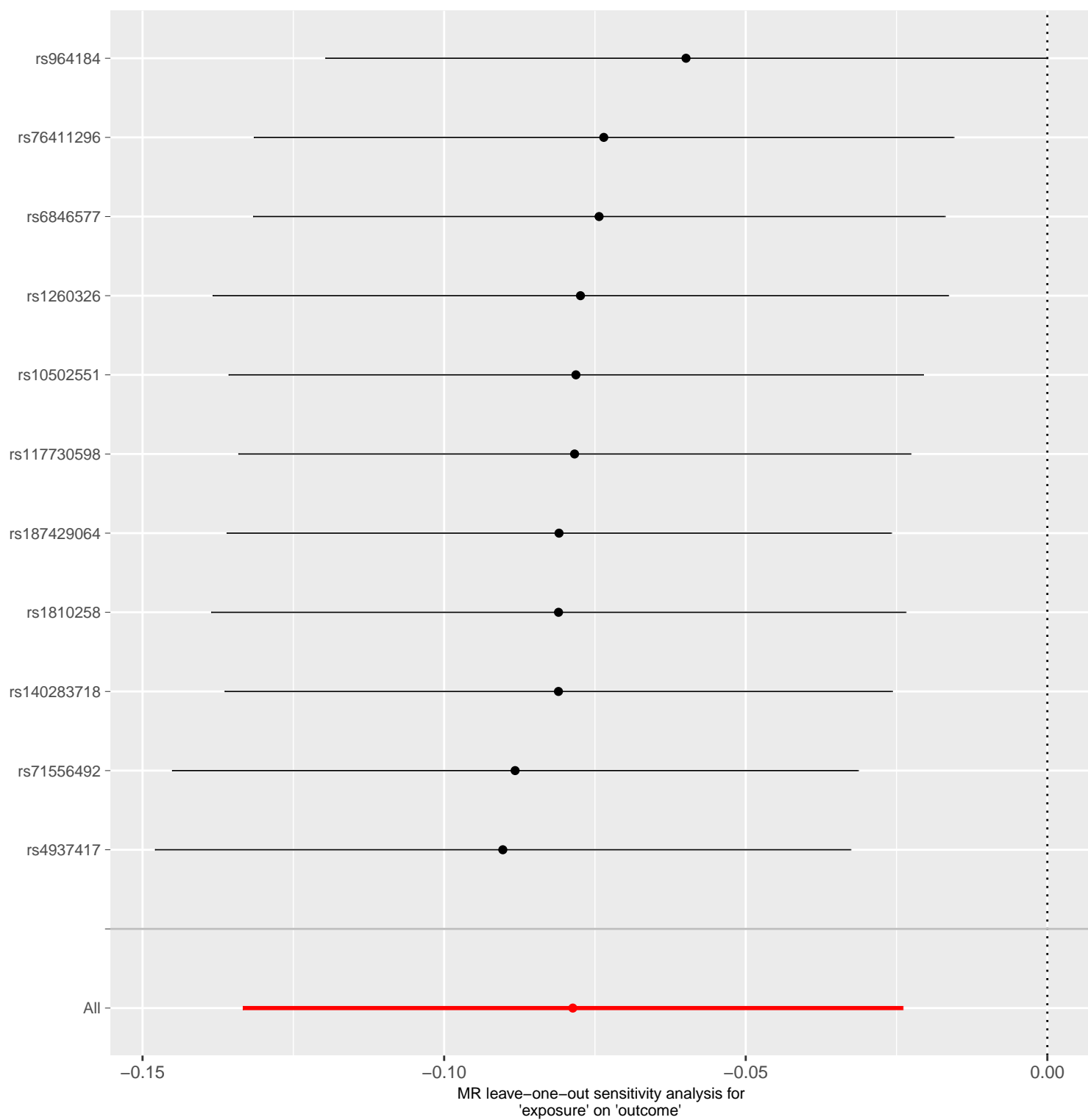

Supplement: Supplementary file 3 — Supplementary Material 3. [file 12944_2024_2103_MOESM3_ESM.zip › sFigure2∩╝êlipidomes-ER+BC∩╝ë/GCST90277386/sensitivity-analysis.pdf]

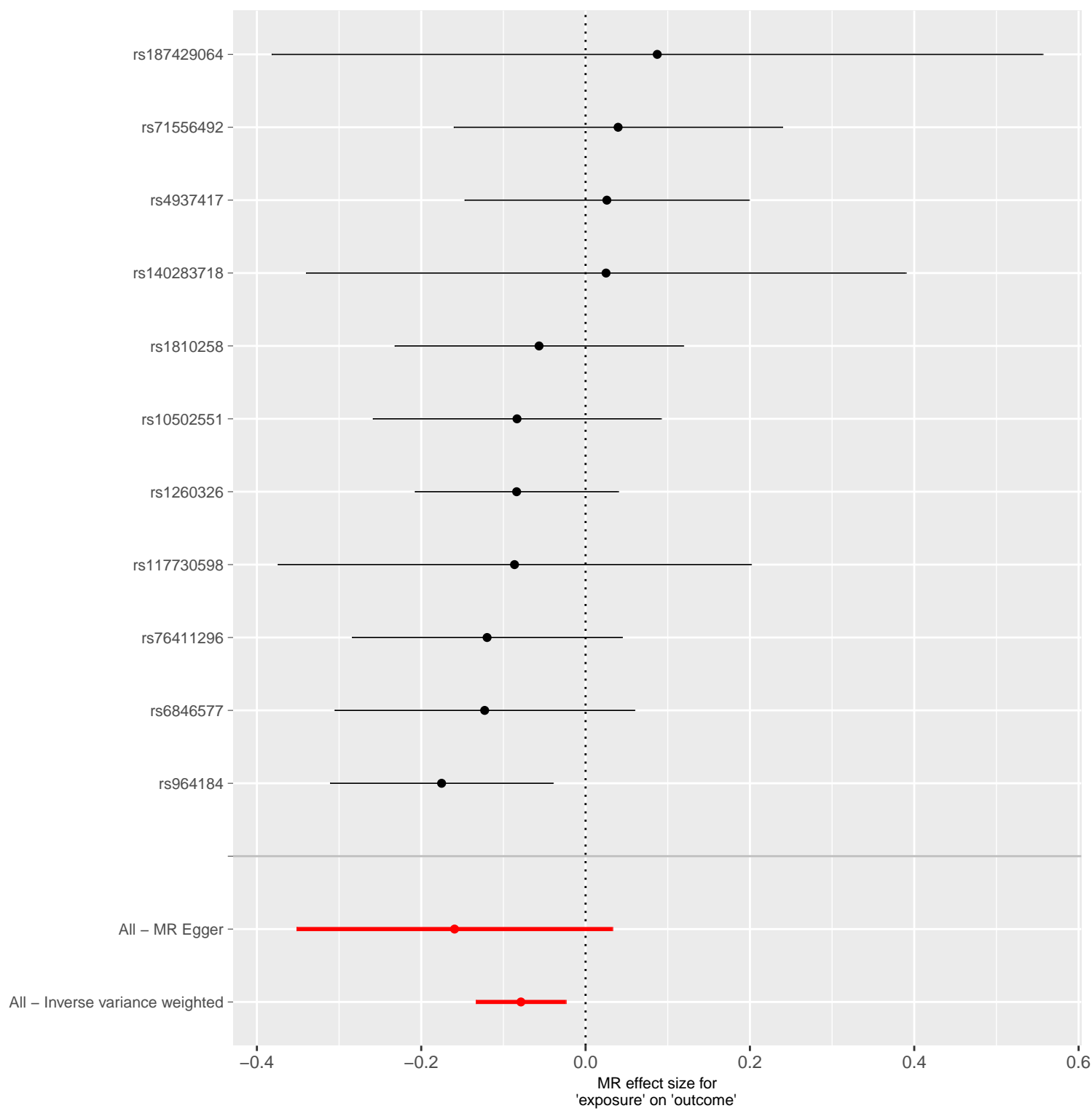

Supplement: Supplementary file 3 — Supplementary Material 3. [file 12944_2024_2103_MOESM3_ESM.zip › sFigure2∩╝êlipidomes-ER+BC∩╝ë/GCST90277386/forest.pdf]

# MR Method

- Inverse variance weighted
- MR Egger

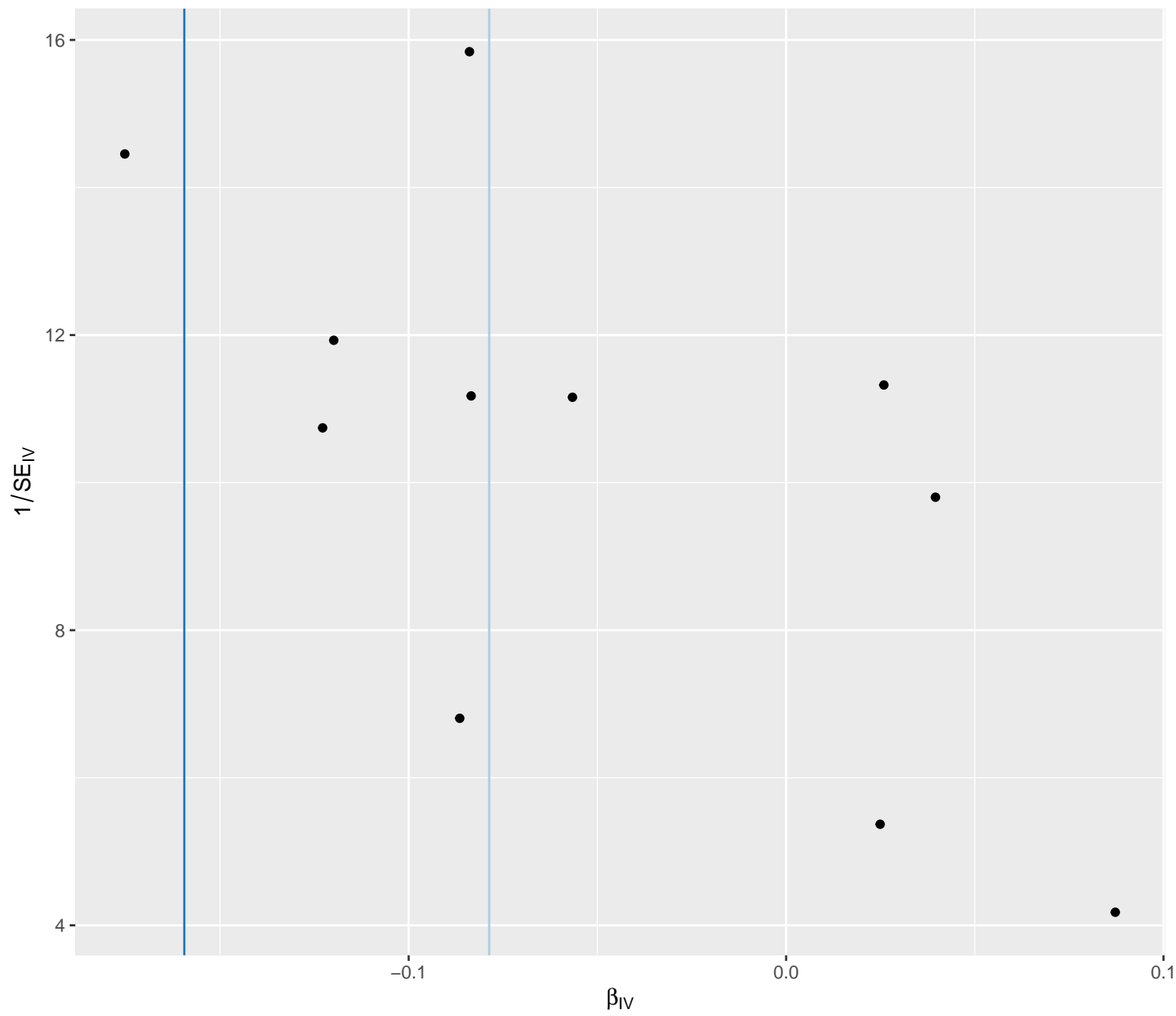

Supplement: Supplementary file 3 — Supplementary Material 3. [file 12944_2024_2103_MOESM3_ESM.zip › sFigure2∩╝êlipidomes-ER+BC∩╝ë/GCST90277386/funnelplot.pdf]

# MR Test

- Inverse variance weighted
- MR Egger
- Simple mode
- Weighted median
- Weighted mode

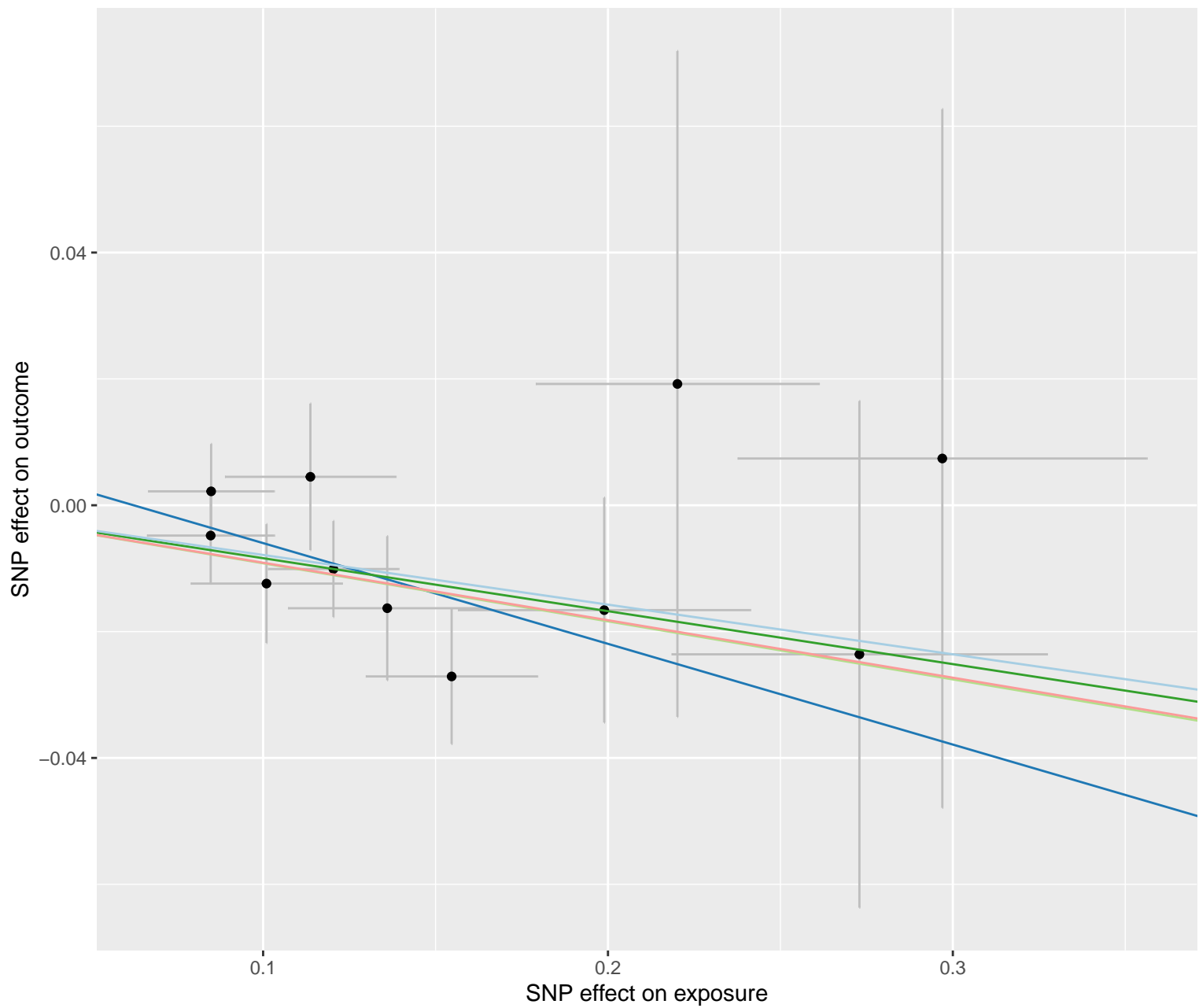

Supplement: Supplementary file 3 — Supplementary Material 3. [file 12944_2024_2103_MOESM3_ESM.zip › sFigure2∩╝êlipidomes-ER+BC∩╝ë/GCST90277386/scatter.pdf]

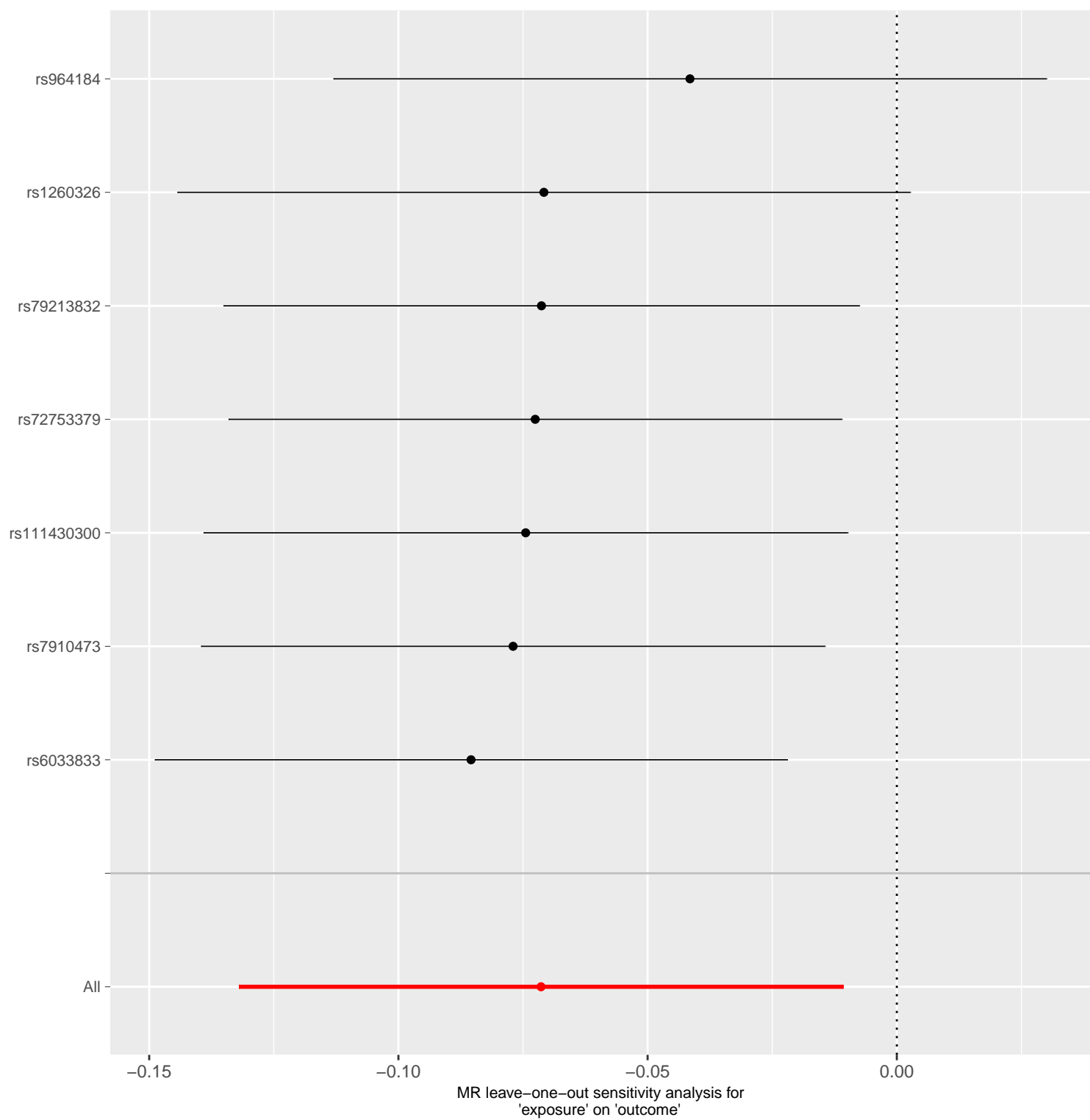

Supplement: Supplementary file 3 — Supplementary Material 3. [file 12944_2024_2103_MOESM3_ESM.zip › sFigure2∩╝êlipidomes-ER+BC∩╝ë/GCST90277388/sensitivity-analysis.pdf]

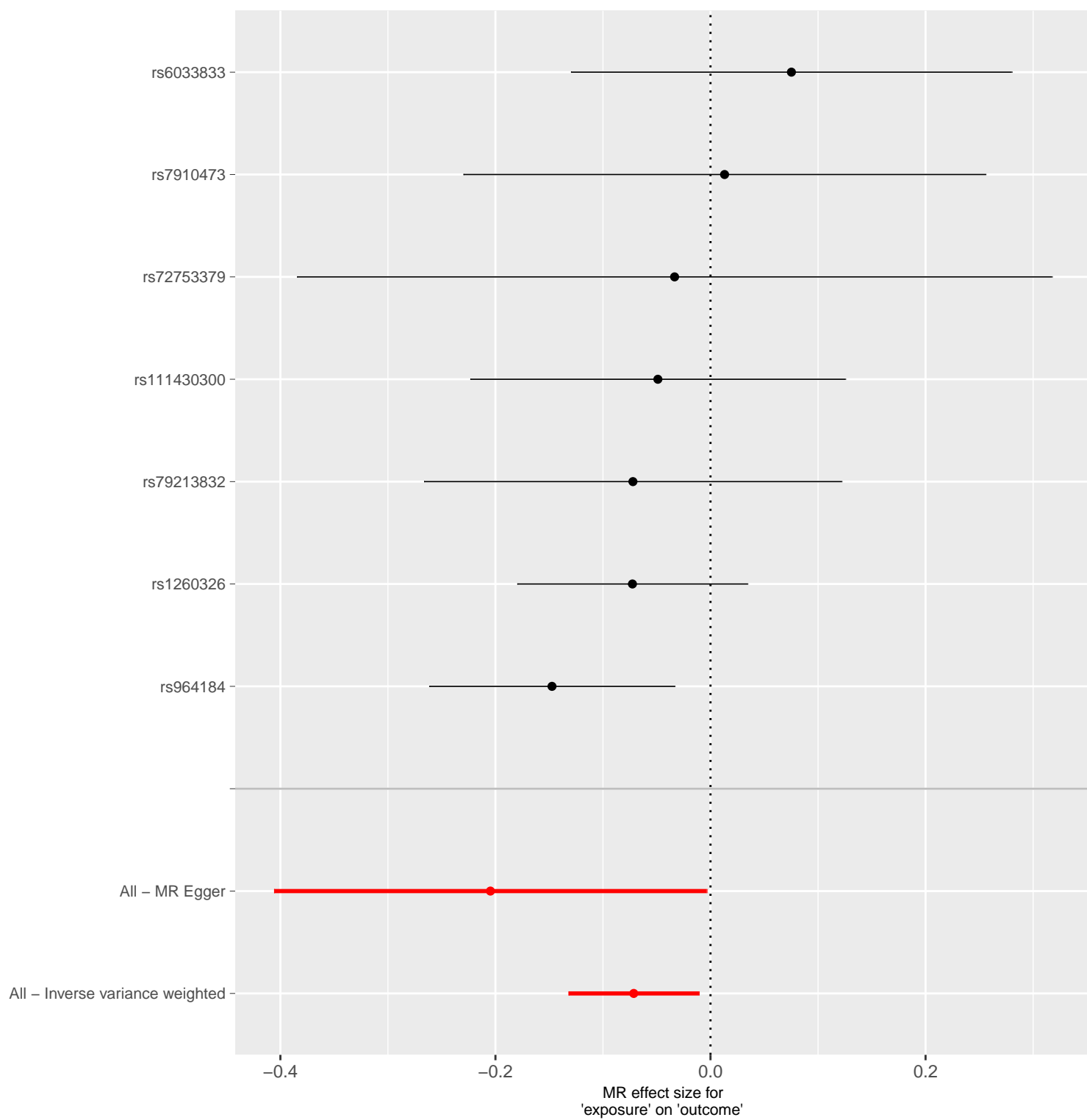

Supplement: Supplementary file 3 — Supplementary Material 3. [file 12944_2024_2103_MOESM3_ESM.zip › sFigure2∩╝êlipidomes-ER+BC∩╝ë/GCST90277388/forest.pdf]

# MR Method

- Inverse variance weighted
- MR Egger

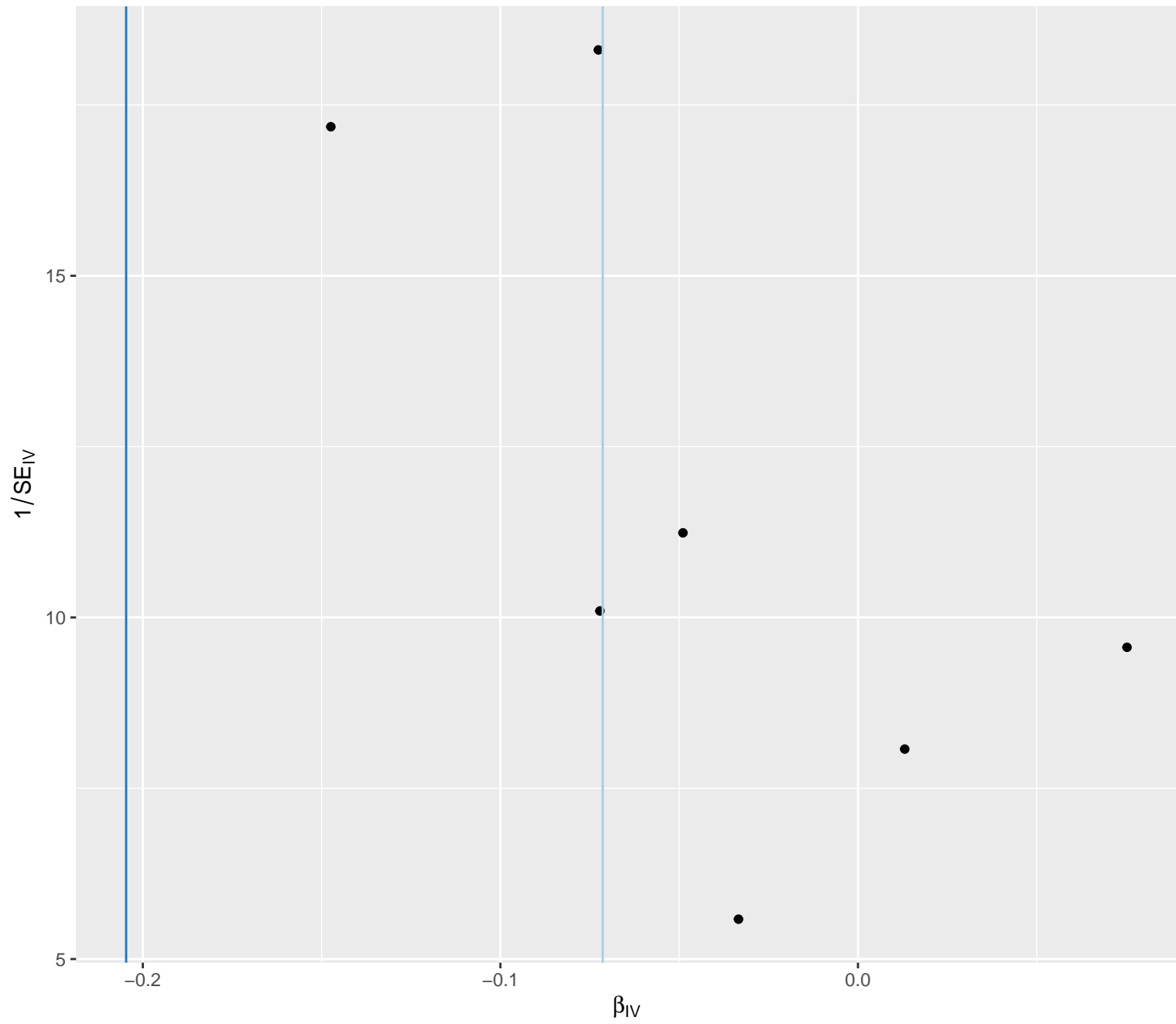

Supplement: Supplementary file 3 — Supplementary Material 3. [file 12944_2024_2103_MOESM3_ESM.zip › sFigure2∩╝êlipidomes-ER+BC∩╝ë/GCST90277388/funnelplot.pdf]

# MR Test

- Inverse variance weighted
- MR Egger
- Simple mode
- Weighted median
- Weighted mode

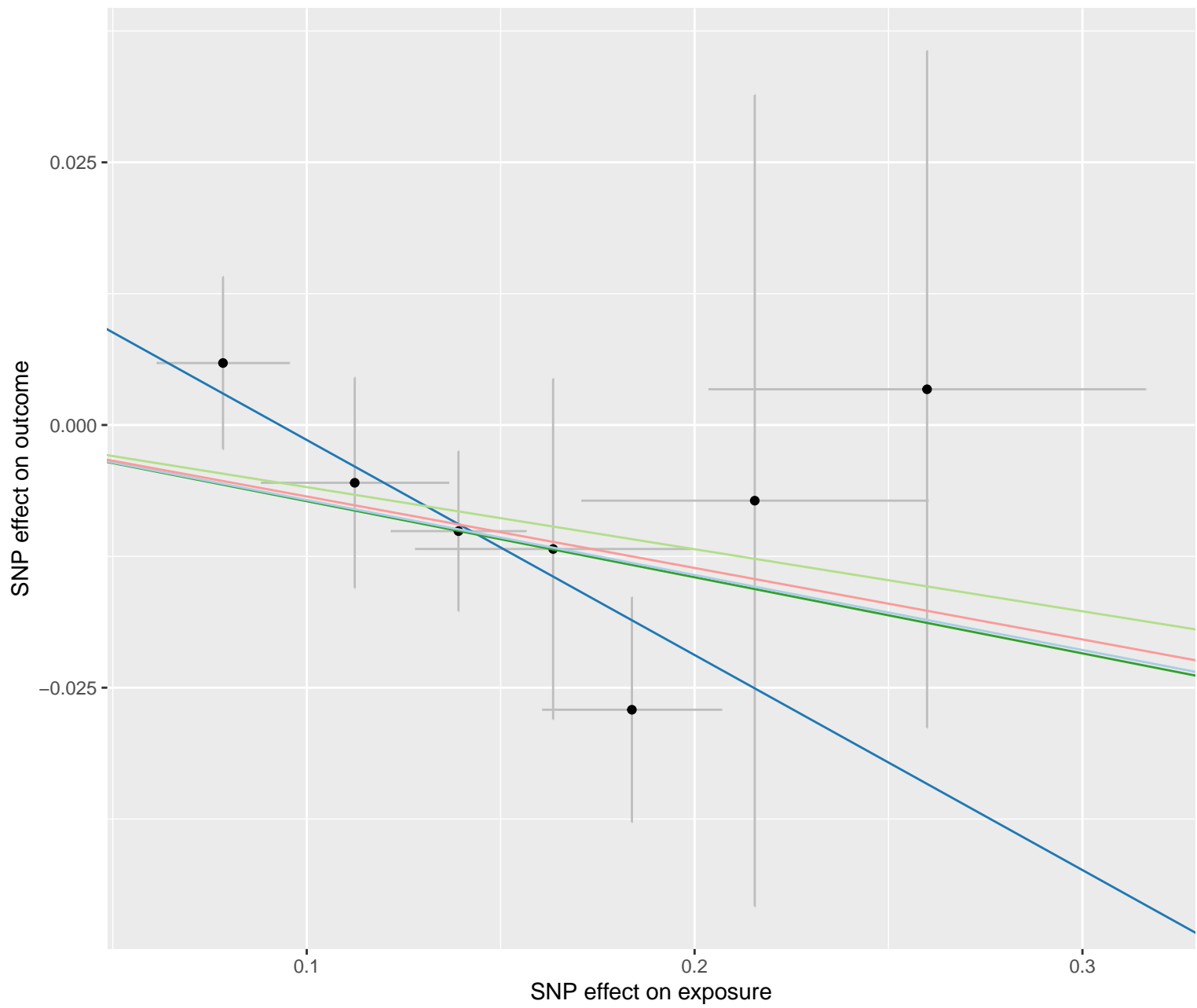

Supplement: Supplementary file 3 — Supplementary Material 3. [file 12944_2024_2103_MOESM3_ESM.zip › sFigure2∩╝êlipidomes-ER+BC∩╝ë/GCST90277388/scatter.pdf]
