# Supplementary figures and images for: The impact of lipidome on breast cancer: a Mendelian randomization study (part 3 of 3)
Source: Lipids Health Dis. 2024 Apr 15;23:109. doi: 10.1186/s12944-024-02103-2 (PMC11017498; doi:10.1186/s12944-024-02103-2)

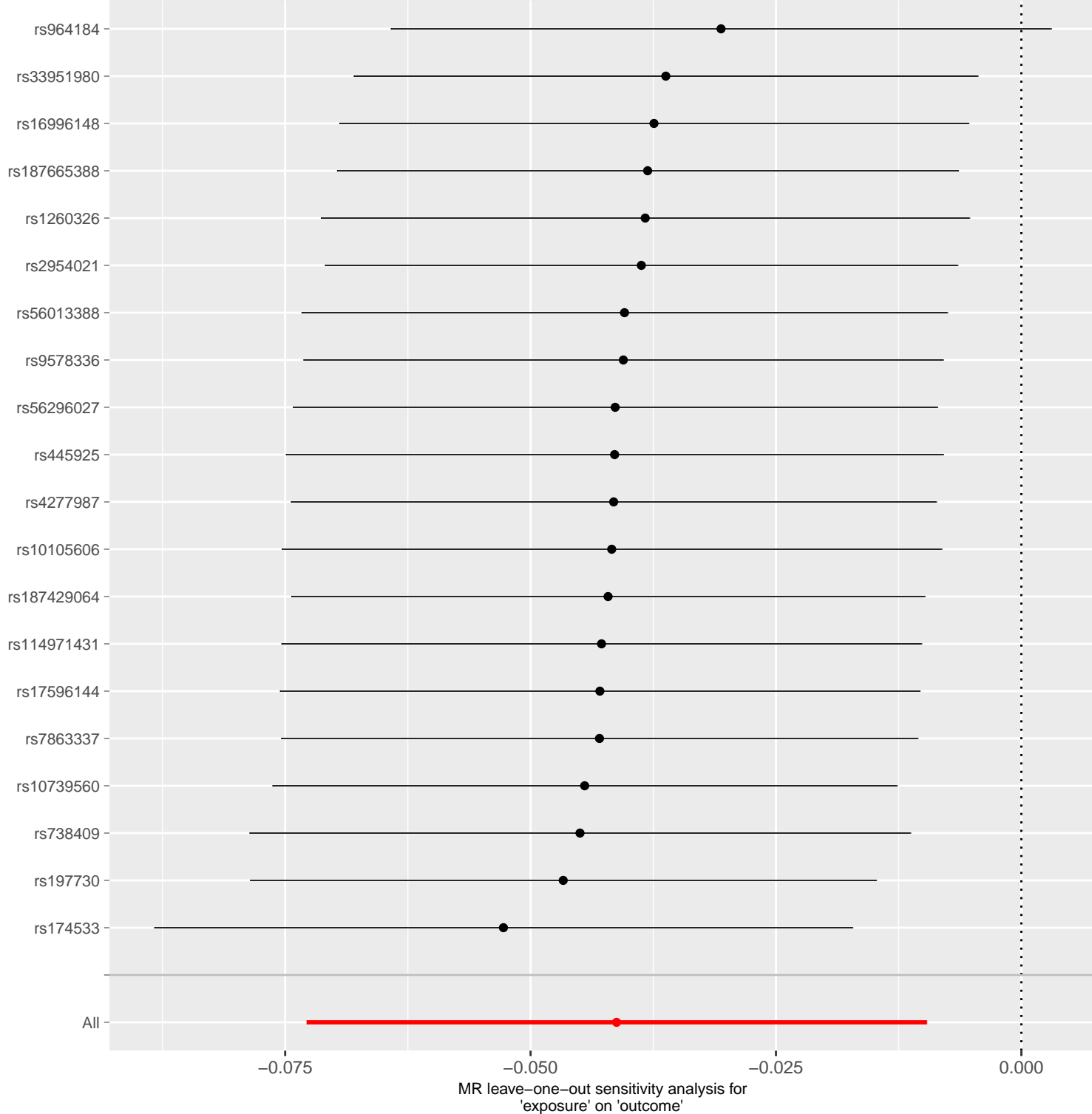

Supplement: Supplementary file 3 — Supplementary Material 3. [file 12944_2024_2103_MOESM3_ESM.zip › sFigure2∩╝êlipidomes-ER+BC∩╝ë/GCST90277412/sensitivity-analysis.pdf]

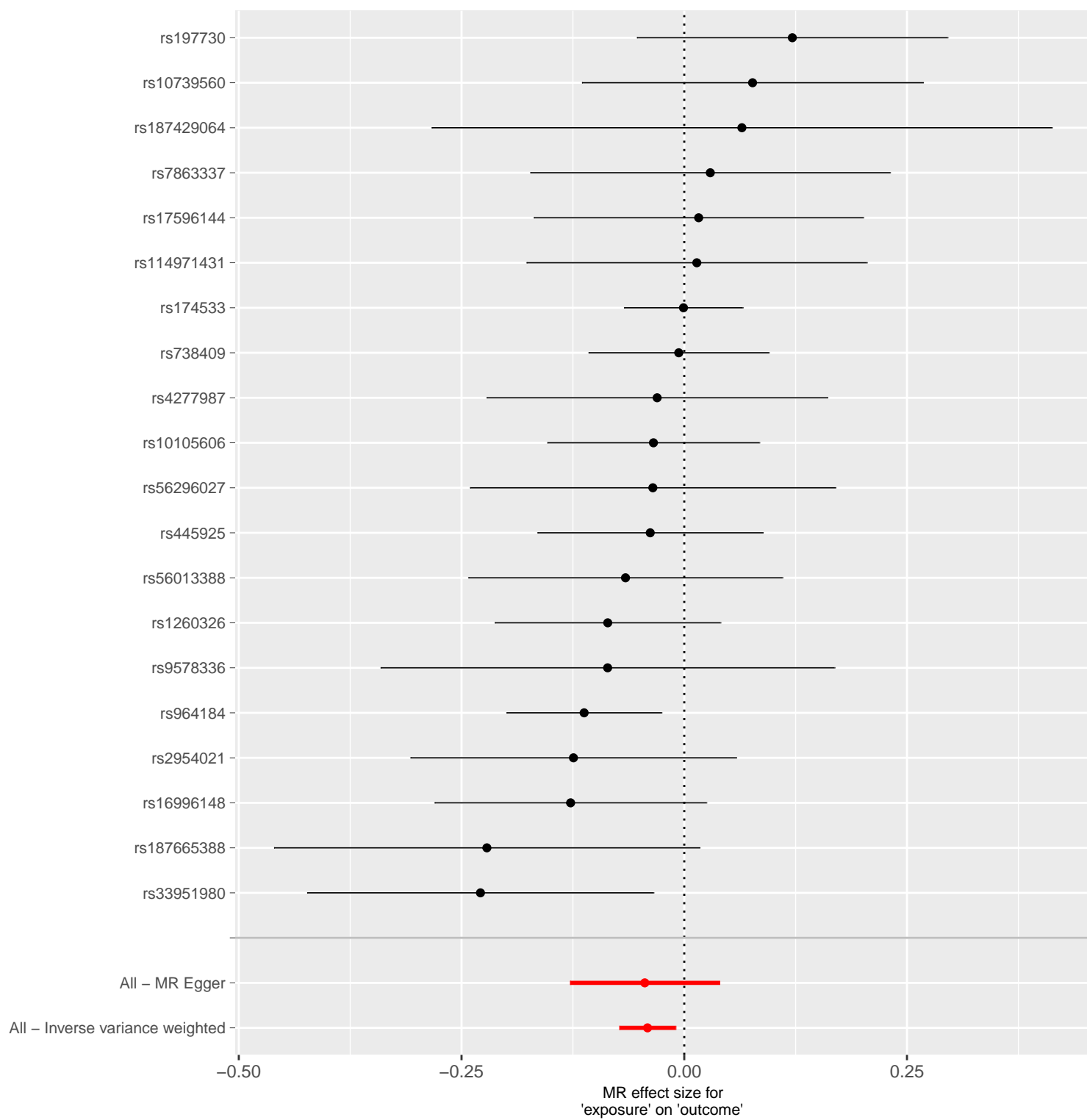

Supplement: Supplementary file 3 — Supplementary Material 3. [file 12944_2024_2103_MOESM3_ESM.zip › sFigure2∩╝êlipidomes-ER+BC∩╝ë/GCST90277412/forest.pdf]

# MR Method

- Inverse variance weighted
- MR Egger

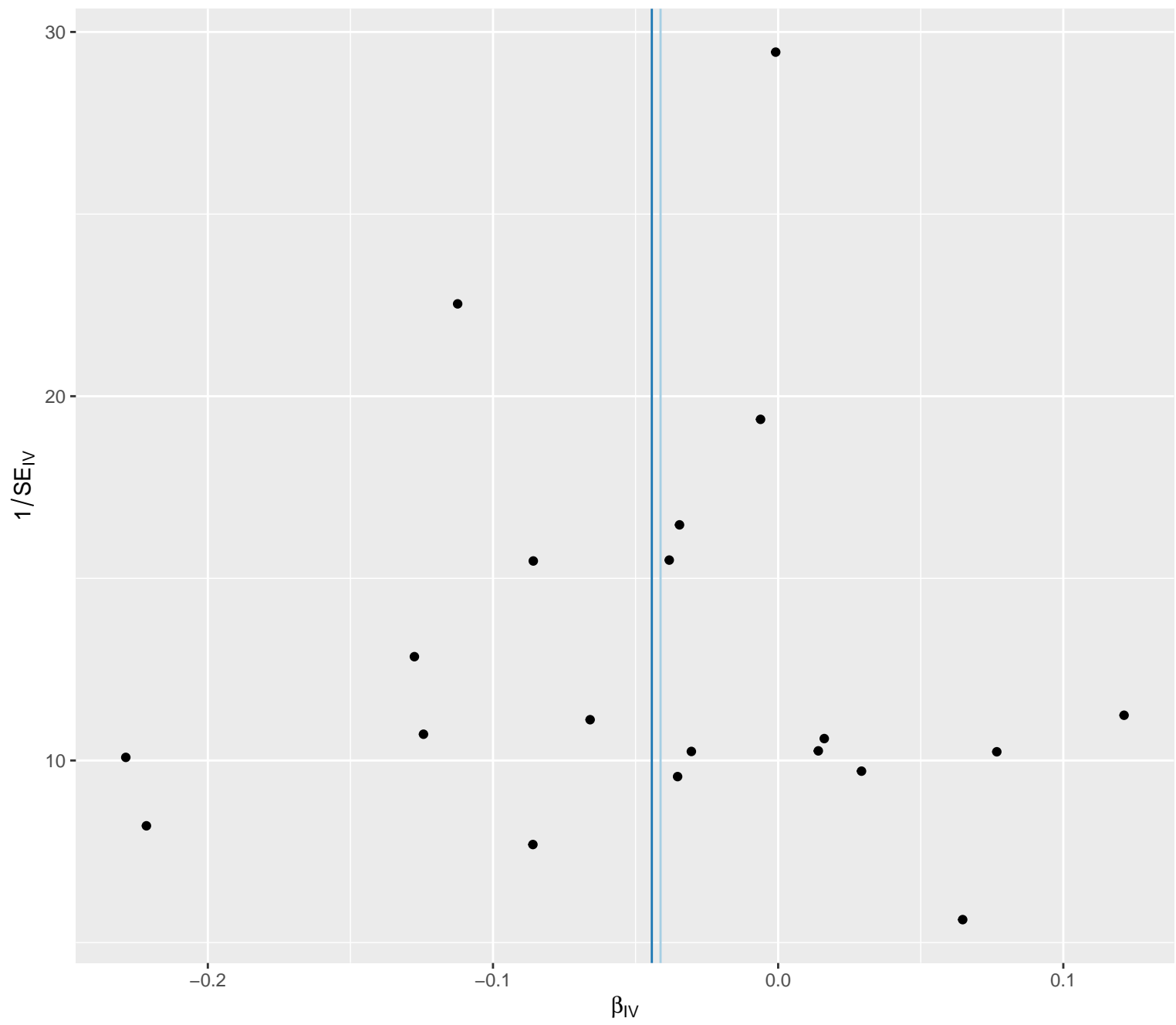

Supplement: Supplementary file 3 — Supplementary Material 3. [file 12944_2024_2103_MOESM3_ESM.zip › sFigure2∩╝êlipidomes-ER+BC∩╝ë/GCST90277412/funnelplot.pdf]

# MR Test

- Inverse variance weighted
- MR Egger
- Simple mode
- Weighted median
- Weighted mode

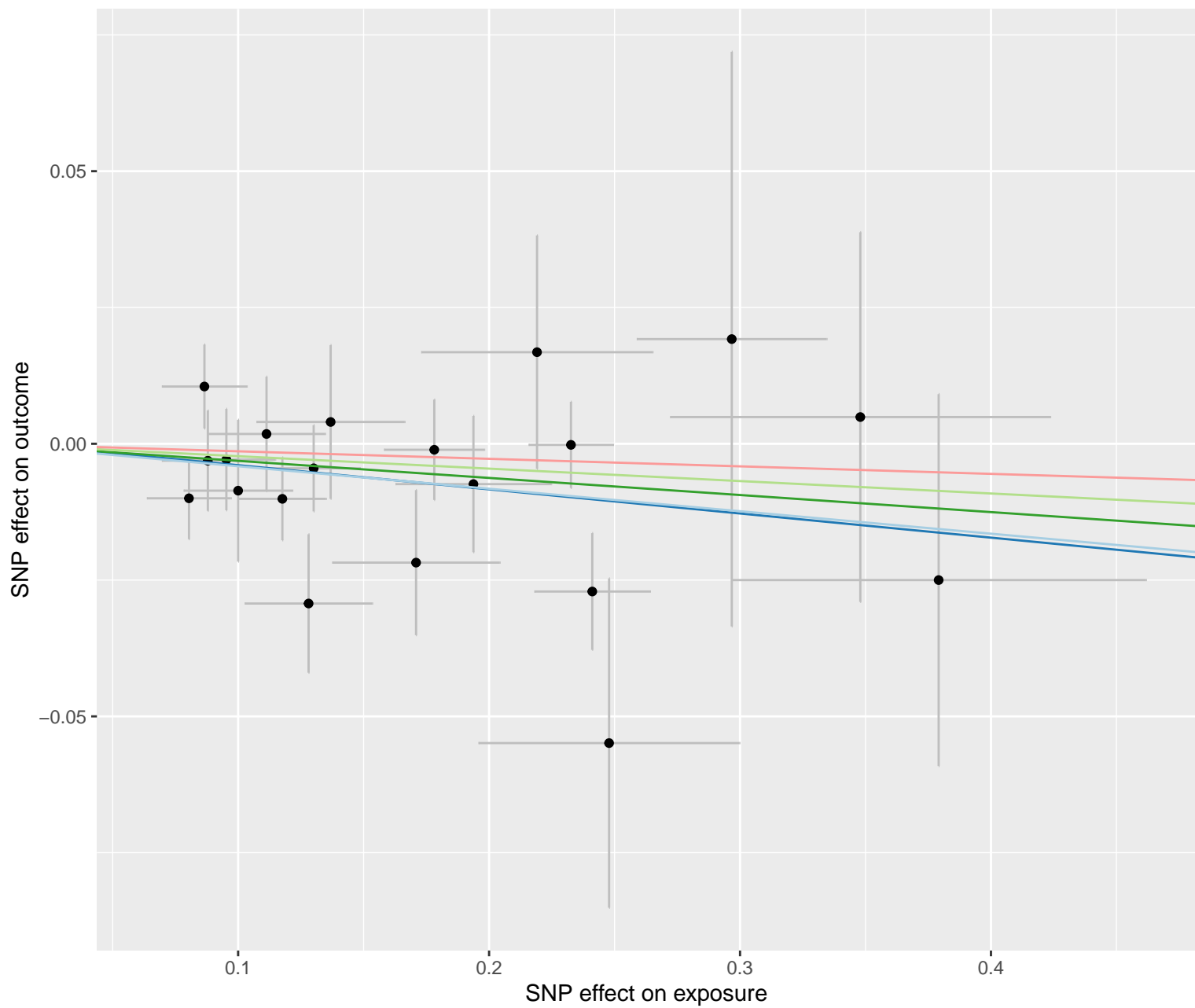

Supplement: Supplementary file 3 — Supplementary Material 3. [file 12944_2024_2103_MOESM3_ESM.zip › sFigure2∩╝êlipidomes-ER+BC∩╝ë/GCST90277412/scatter.pdf]

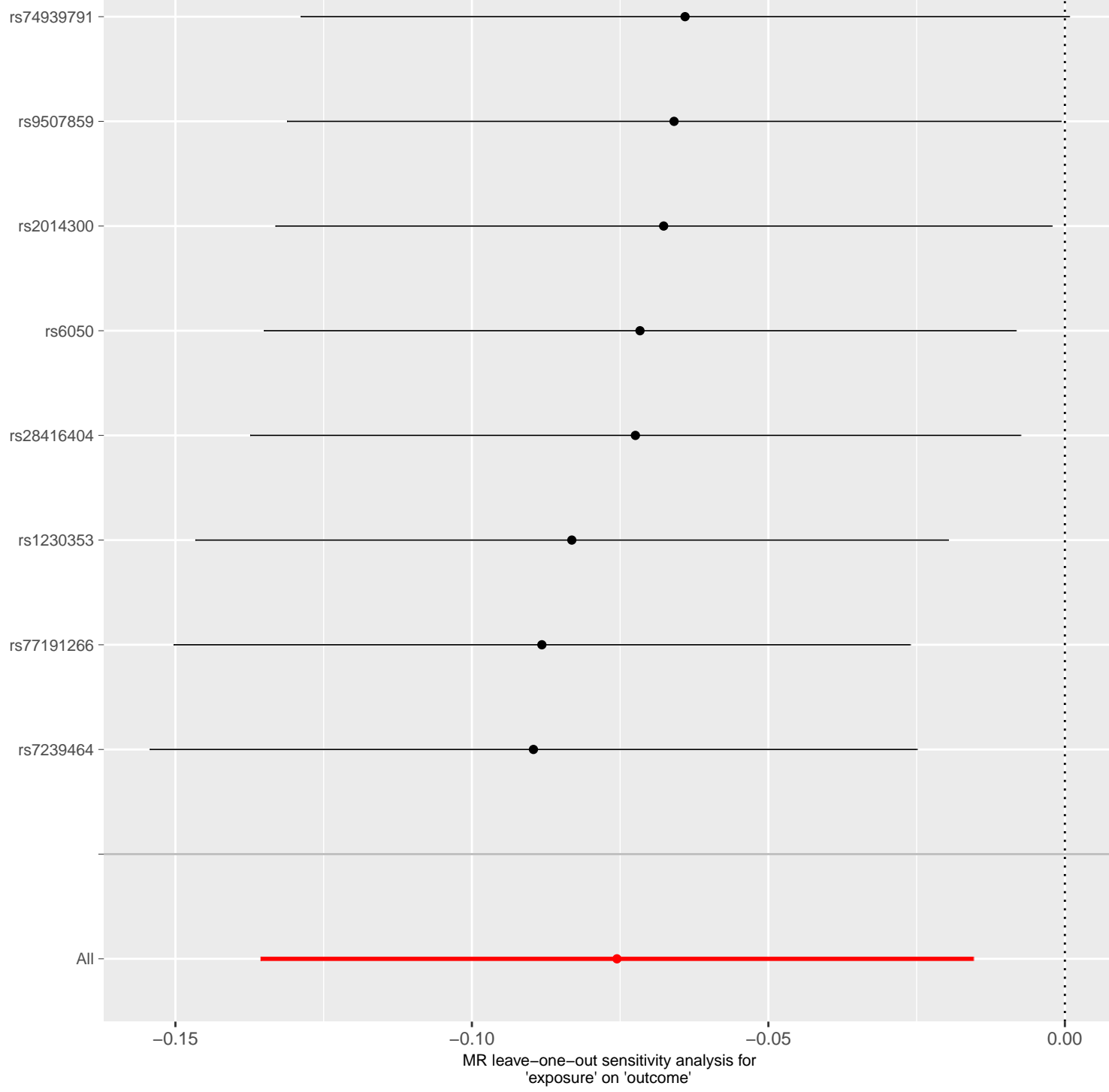

Supplement: Supplementary file 3 — Supplementary Material 3. [file 12944_2024_2103_MOESM3_ESM.zip › sFigure2∩╝êlipidomes-ER+BC∩╝ë/GCST90277272/sensitivity-analysis.pdf]

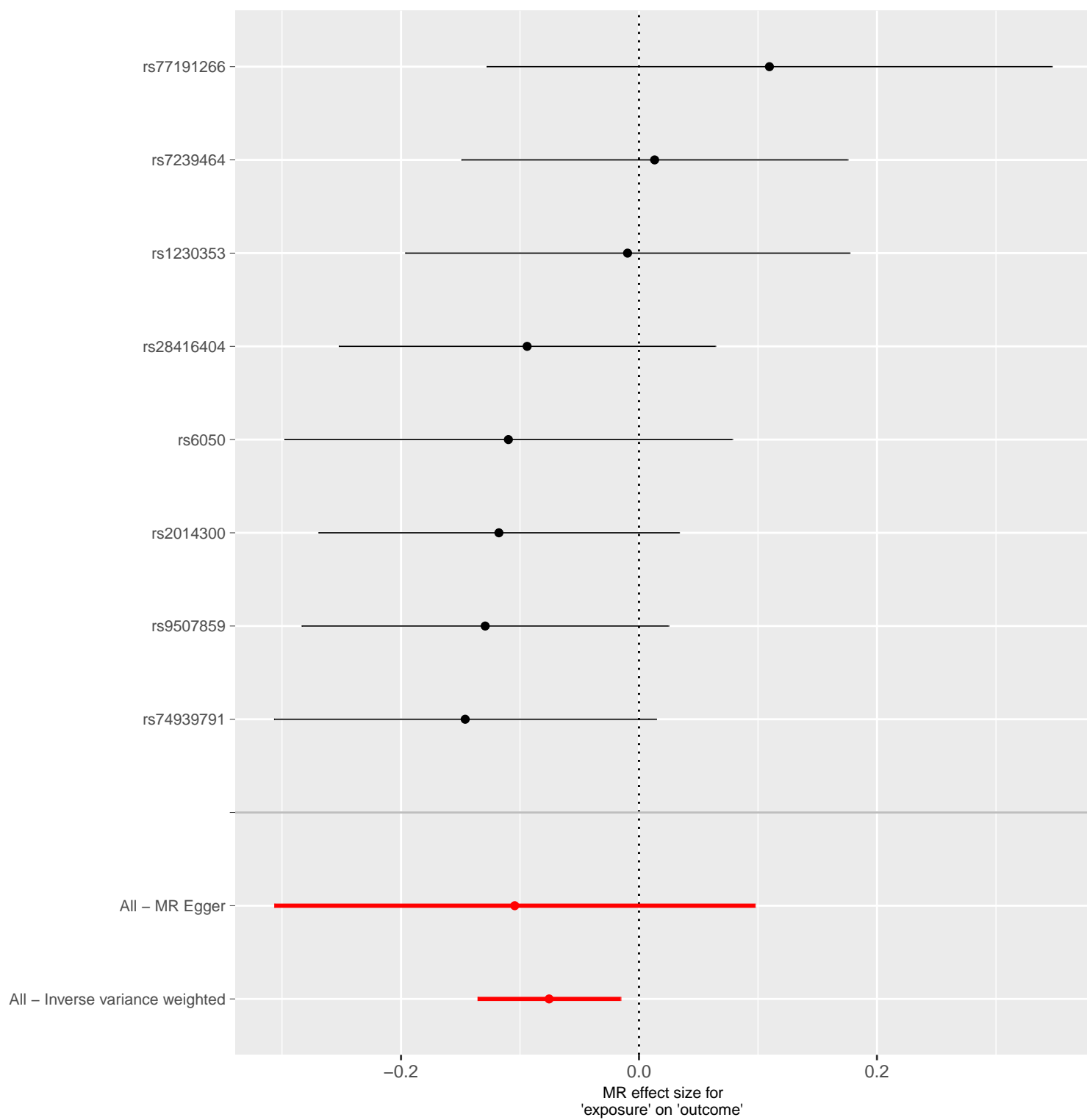

Supplement: Supplementary file 3 — Supplementary Material 3. [file 12944_2024_2103_MOESM3_ESM.zip › sFigure2∩╝êlipidomes-ER+BC∩╝ë/GCST90277272/forest.pdf]

# MR Method

- Inverse variance weighted
- MR Egger

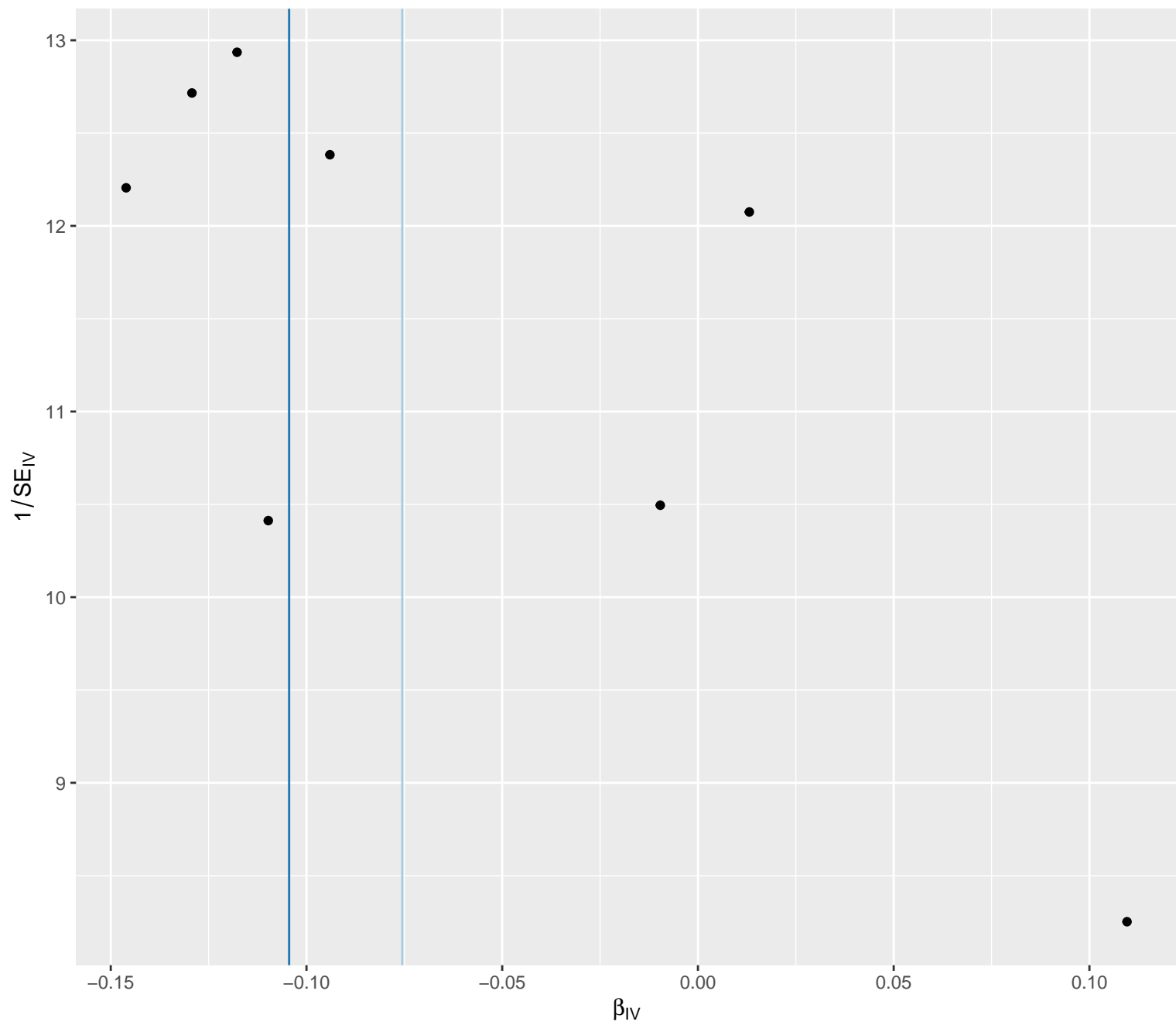

Supplement: Supplementary file 3 — Supplementary Material 3. [file 12944_2024_2103_MOESM3_ESM.zip › sFigure2∩╝êlipidomes-ER+BC∩╝ë/GCST90277272/funnelplot.pdf]

# MR Test

- Inverse variance weighted
- MR Egger
- Simple mode
- Weighted median
- Weighted mode

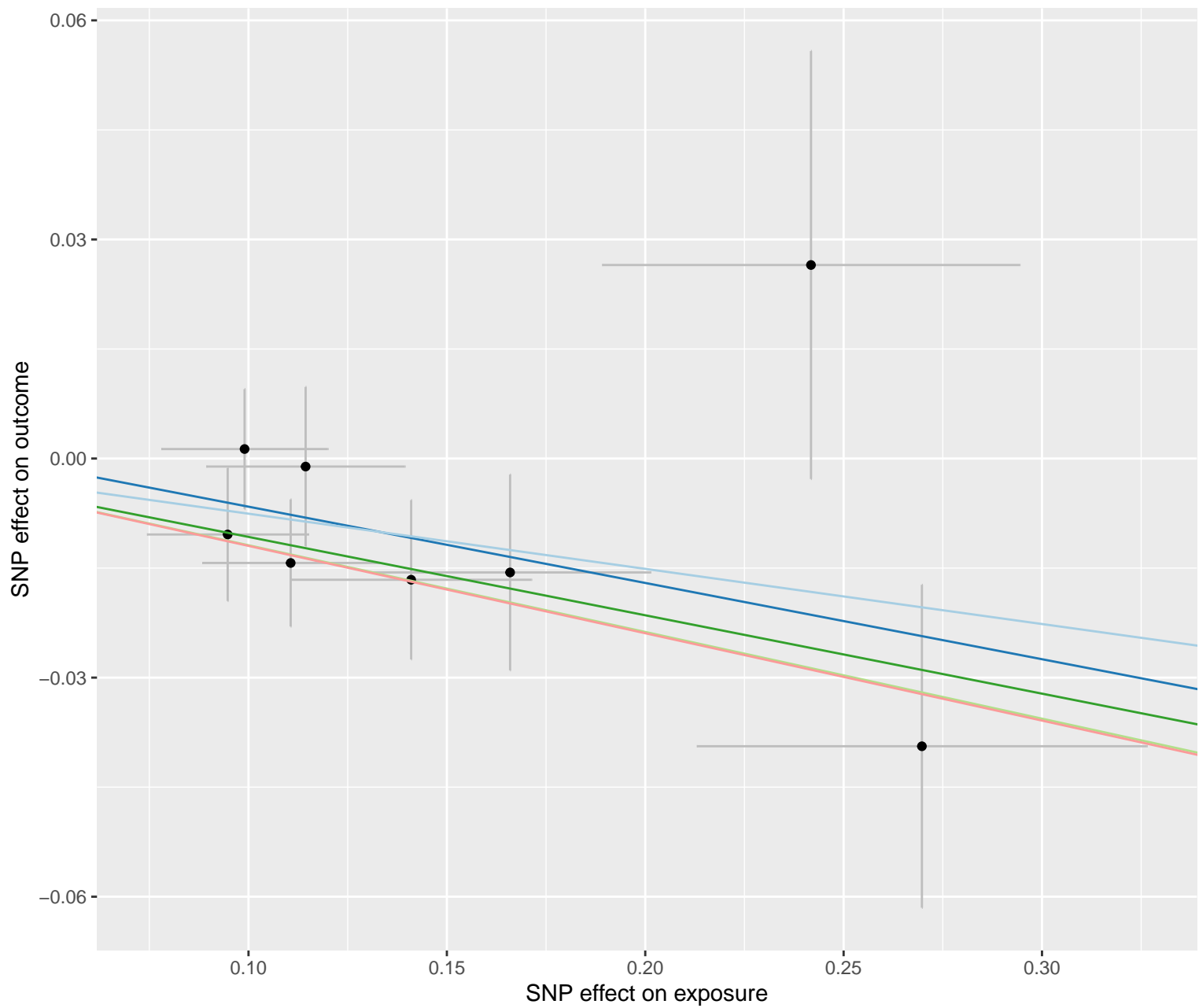

Supplement: Supplementary file 3 — Supplementary Material 3. [file 12944_2024_2103_MOESM3_ESM.zip › sFigure2∩╝êlipidomes-ER+BC∩╝ë/GCST90277272/scatter.pdf]

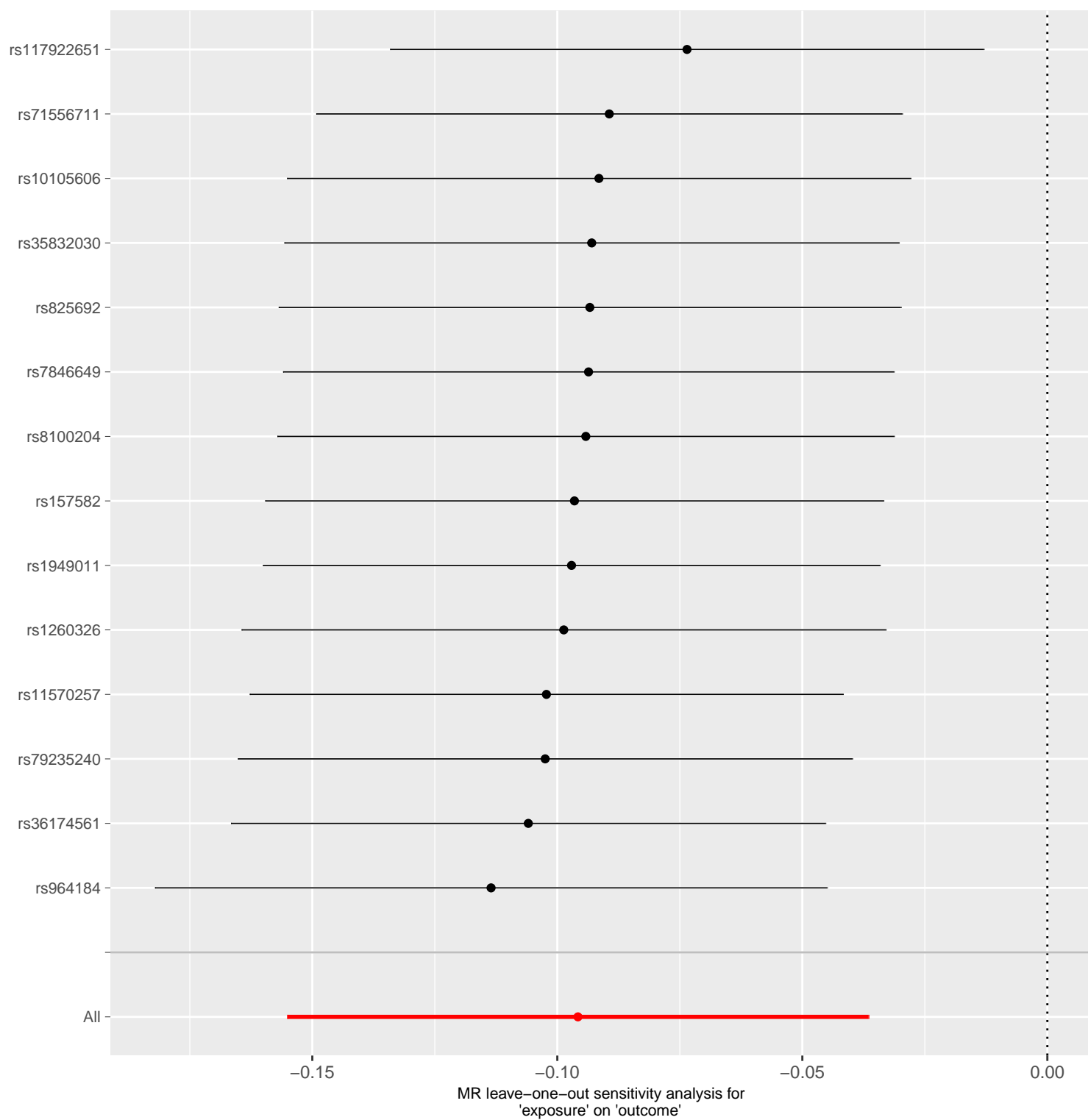

Supplement: Supplementary file 4 — Supplementary Material 4. [file 12944_2024_2103_MOESM4_ESM.zip › sFigure3∩╝êlipidomes-ER-BC∩╝ë/GCST90277403/sensitivity-analysis.pdf]

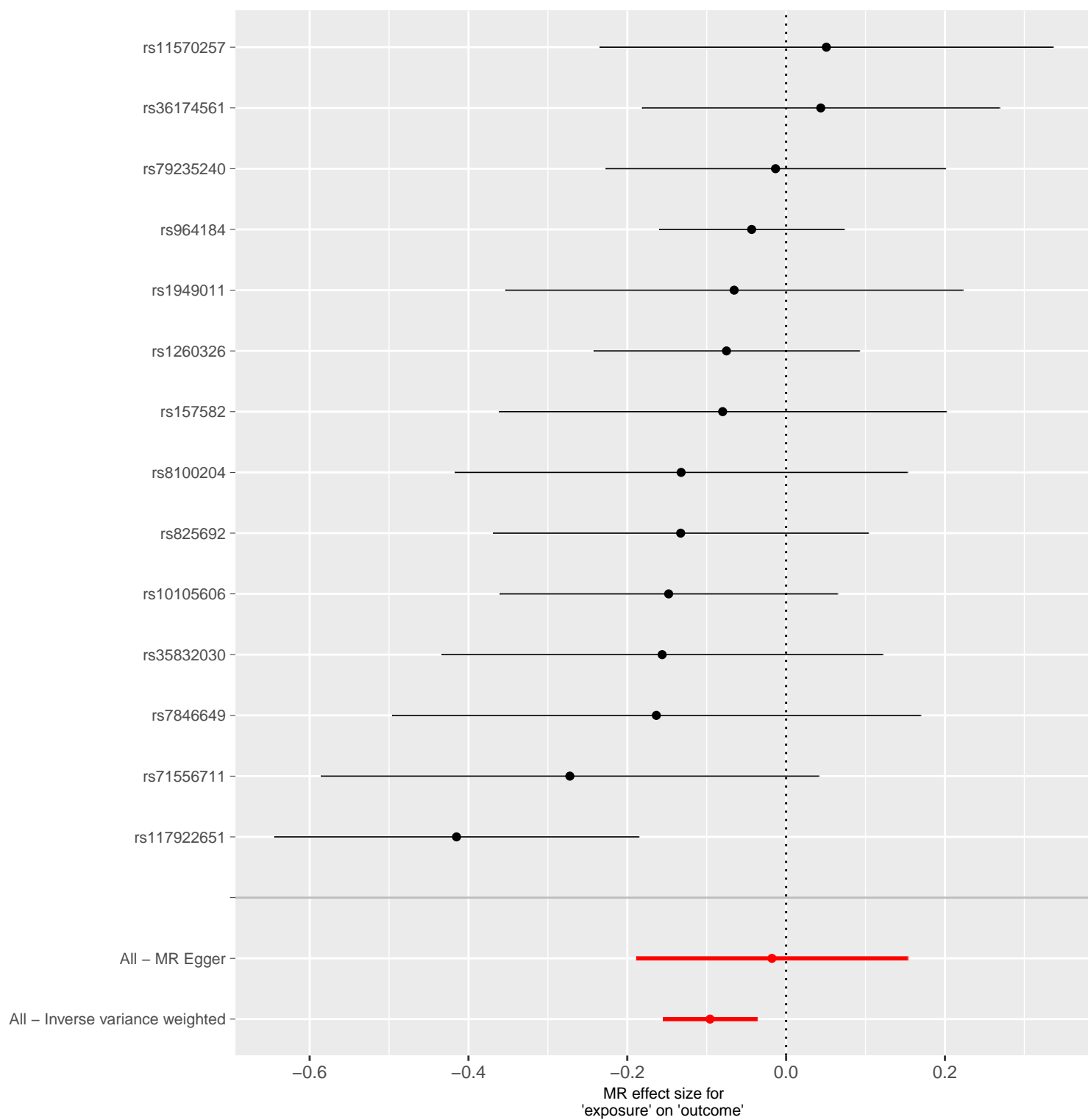

Supplement: Supplementary file 4 — Supplementary Material 4. [file 12944_2024_2103_MOESM4_ESM.zip › sFigure3∩╝êlipidomes-ER-BC∩╝ë/GCST90277403/forest.pdf]

# MR Method

- Inverse variance weighted
- MR Egger

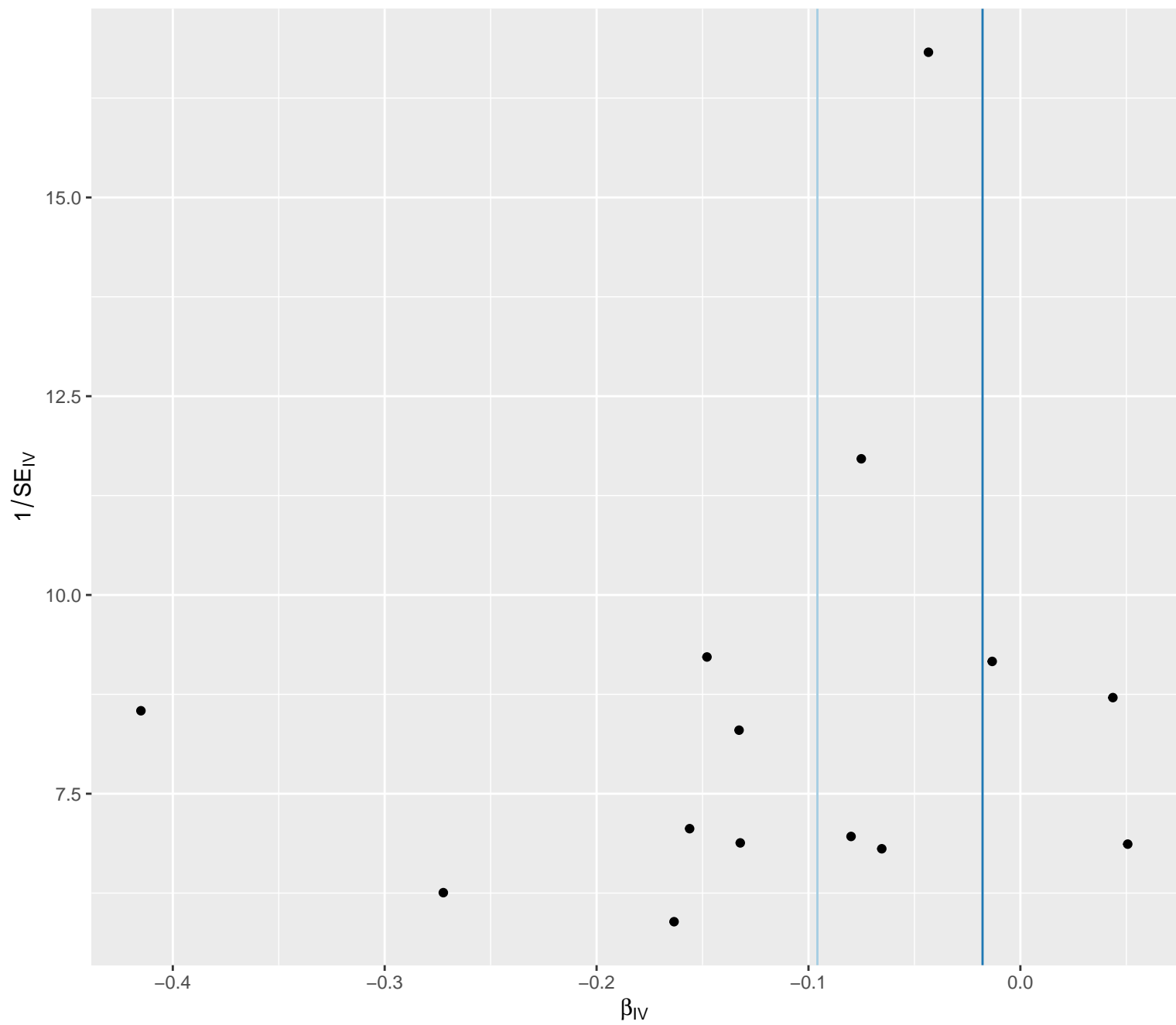

Supplement: Supplementary file 4 — Supplementary Material 4. [file 12944_2024_2103_MOESM4_ESM.zip › sFigure3∩╝êlipidomes-ER-BC∩╝ë/GCST90277403/funnelplot.pdf]

# MR Test

- Inverse variance weighted
- MR Egger
- Simple mode
- Weighted median
- Weighted mode

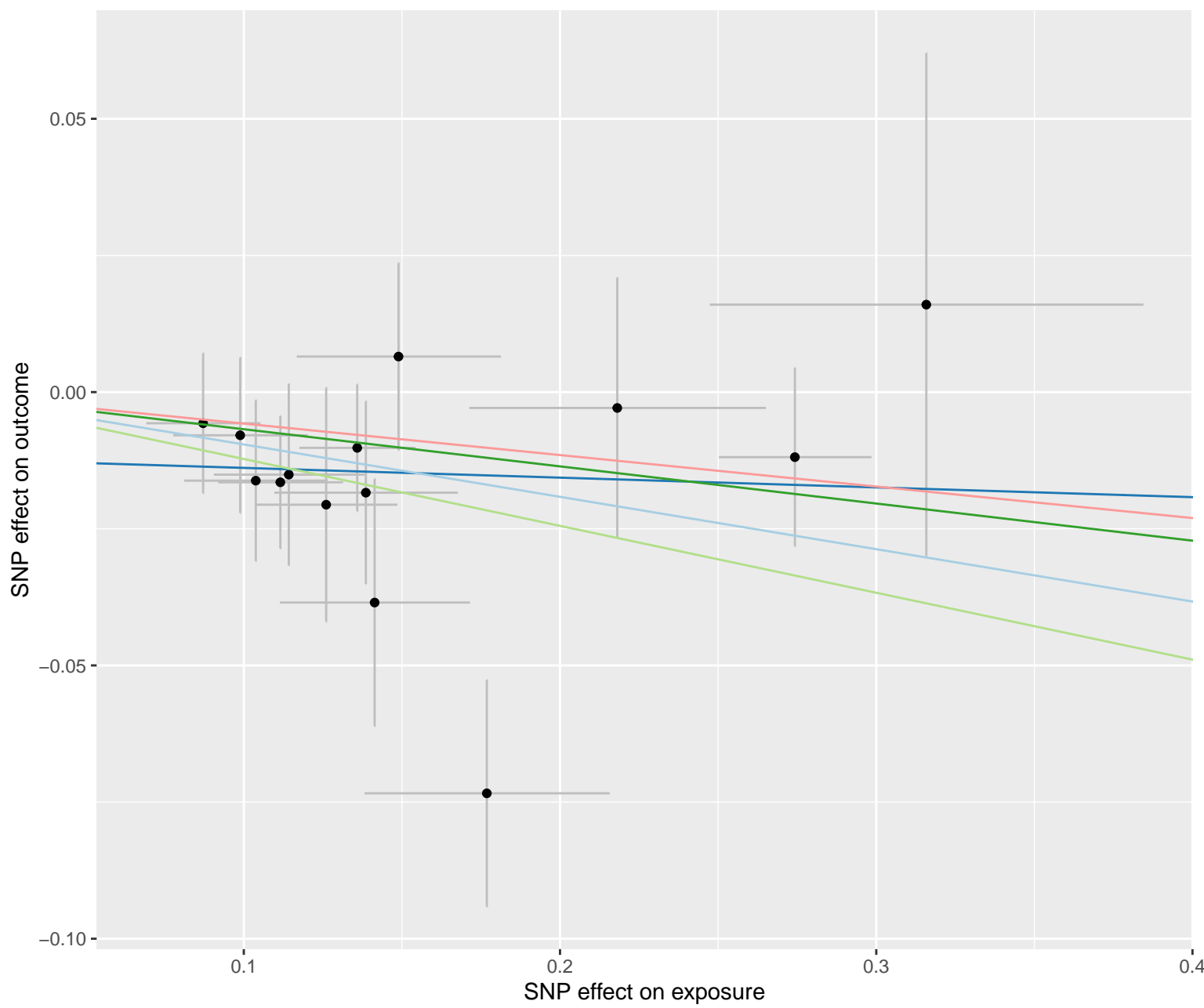

Supplement: Supplementary file 4 — Supplementary Material 4. [file 12944_2024_2103_MOESM4_ESM.zip › sFigure3∩╝êlipidomes-ER-BC∩╝ë/GCST90277403/scatter.pdf]

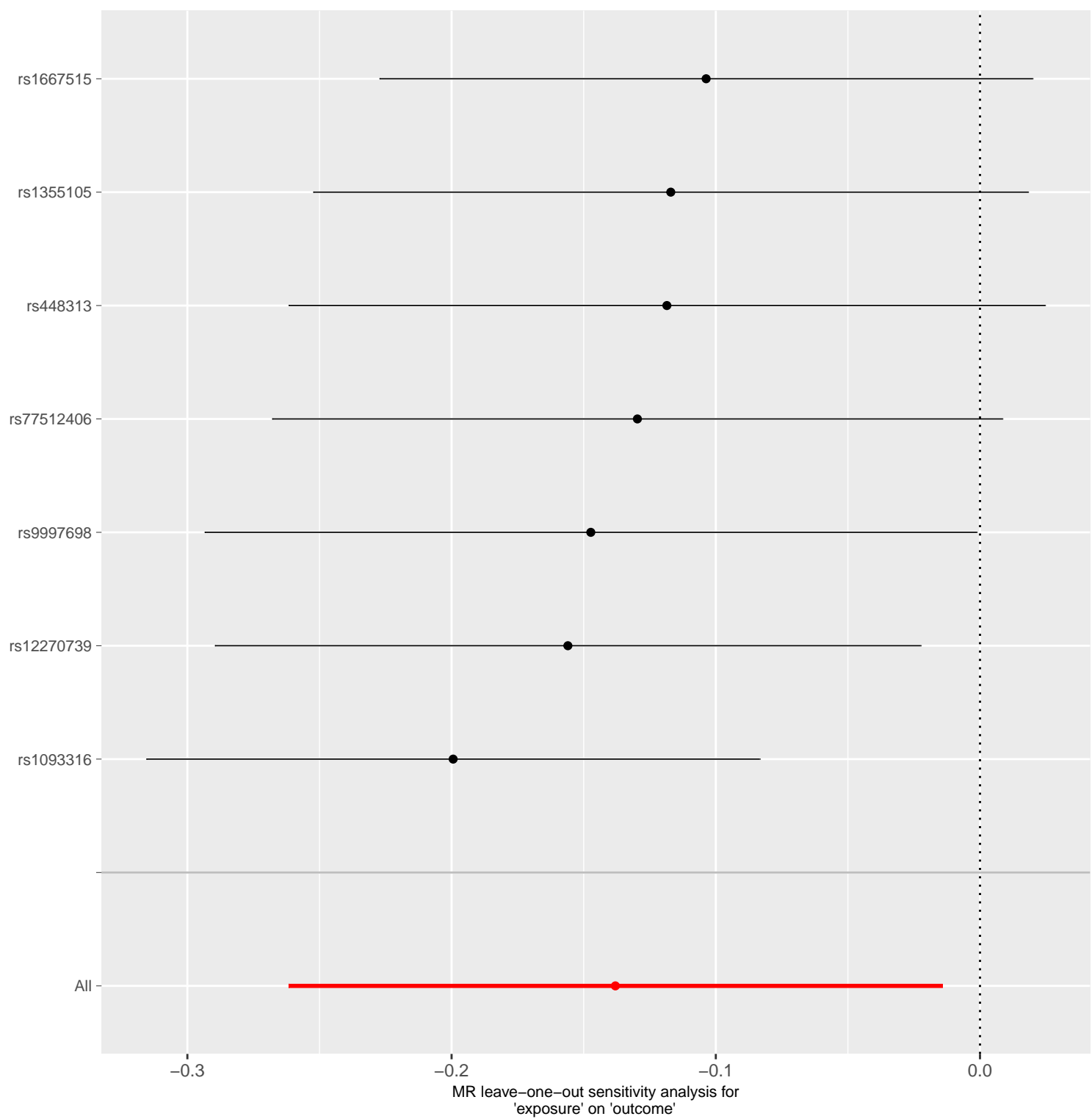

Supplement: Supplementary file 4 — Supplementary Material 4. [file 12944_2024_2103_MOESM4_ESM.zip › sFigure3∩╝êlipidomes-ER-BC∩╝ë/GCST90277326/sensitivity-analysis.pdf]

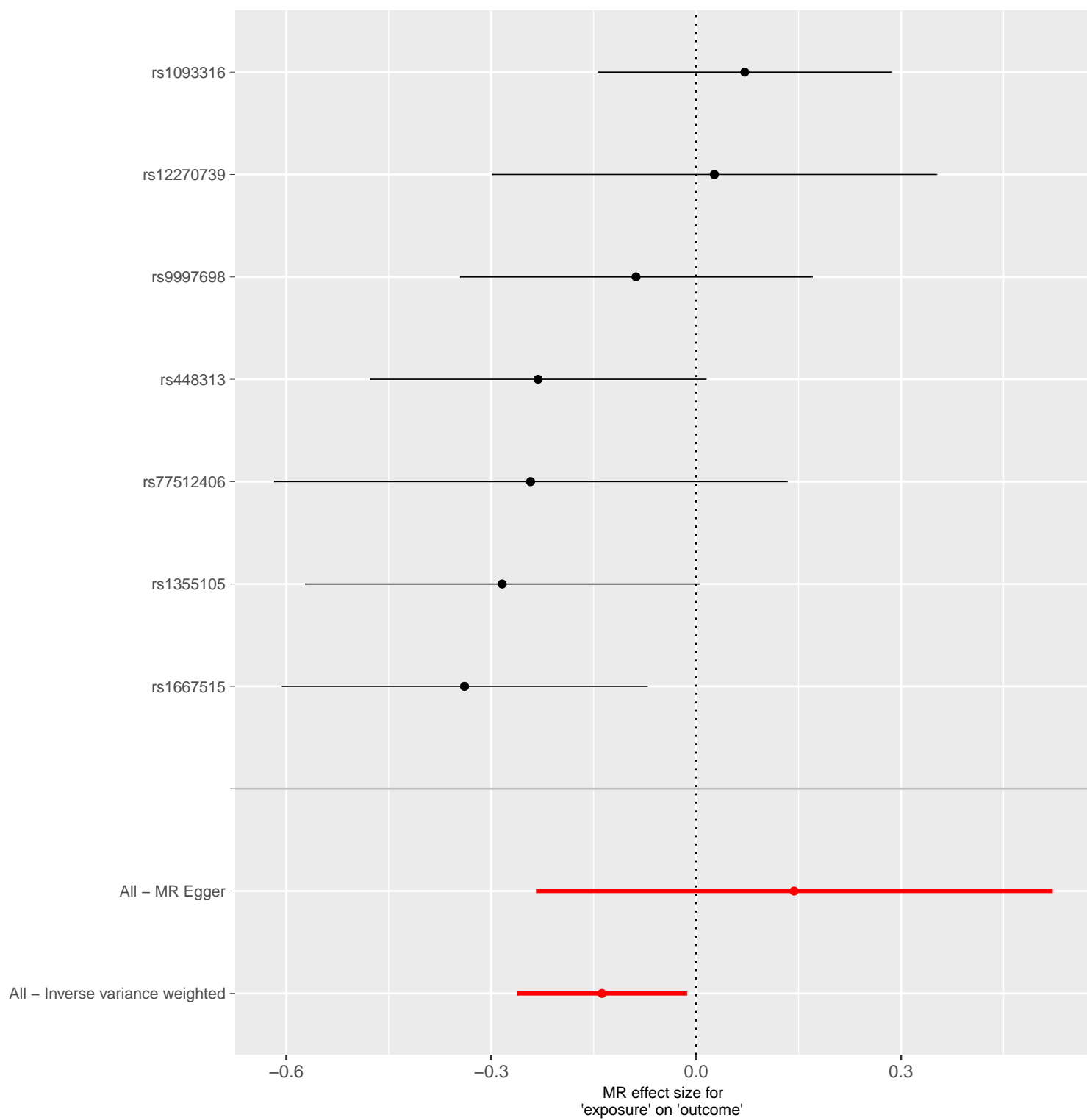

Supplement: Supplementary file 4 — Supplementary Material 4. [file 12944_2024_2103_MOESM4_ESM.zip › sFigure3∩╝êlipidomes-ER-BC∩╝ë/GCST90277326/forest.pdf]

# MR Method

- Inverse variance weighted
- MR Egger

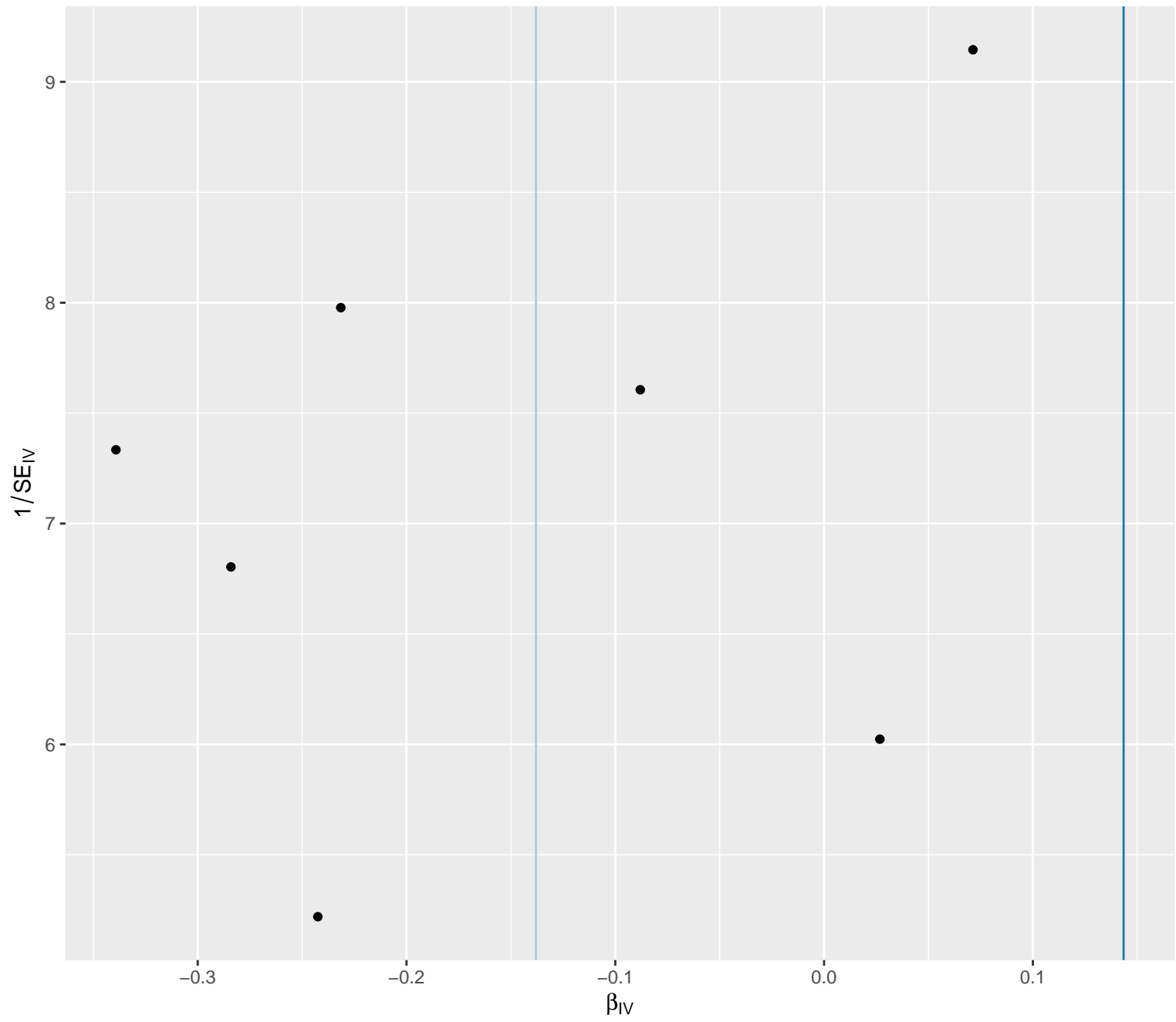

Supplement: Supplementary file 4 — Supplementary Material 4. [file 12944_2024_2103_MOESM4_ESM.zip › sFigure3∩╝êlipidomes-ER-BC∩╝ë/GCST90277326/funnelplot.pdf]

# MR Test

- Inverse variance weighted
- MR Egger
- Simple mode
- Weighted median
- Weighted mode

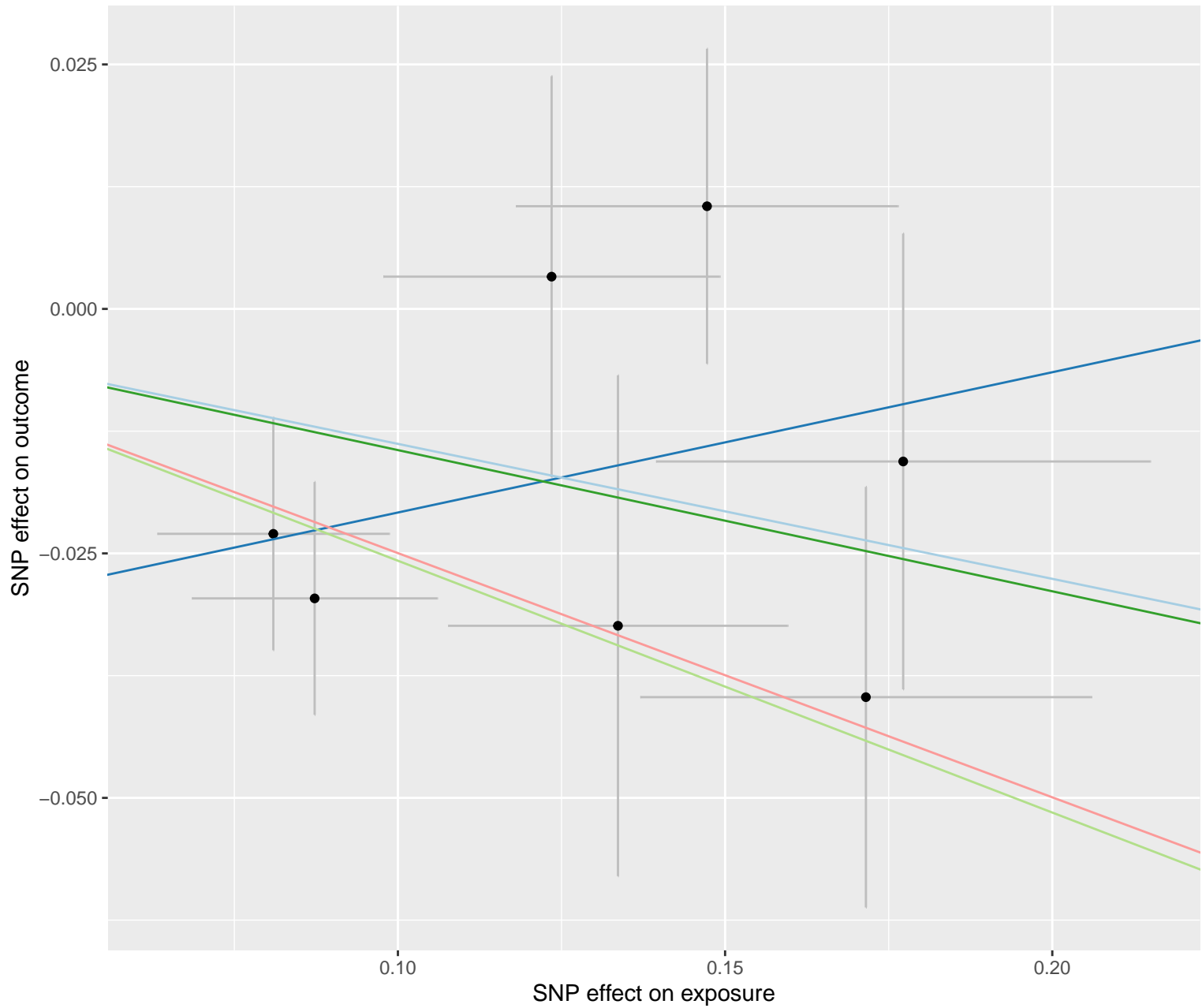

Supplement: Supplementary file 4 — Supplementary Material 4. [file 12944_2024_2103_MOESM4_ESM.zip › sFigure3∩╝êlipidomes-ER-BC∩╝ë/GCST90277326/scatter.pdf]

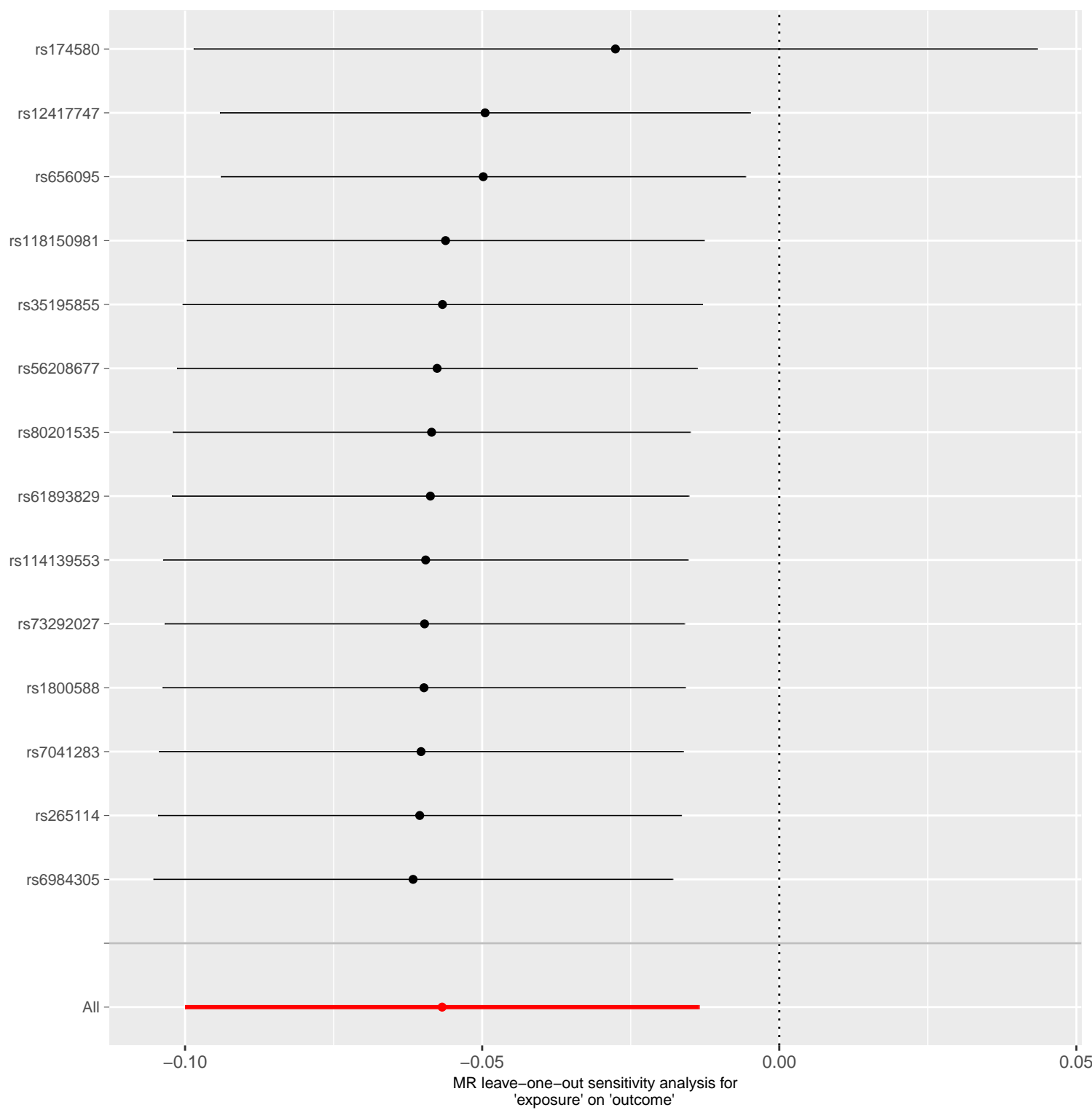

Supplement: Supplementary file 4 — Supplementary Material 4. [file 12944_2024_2103_MOESM4_ESM.zip › sFigure3∩╝êlipidomes-ER-BC∩╝ë/GCST90277311/sensitivity-analysis.pdf]

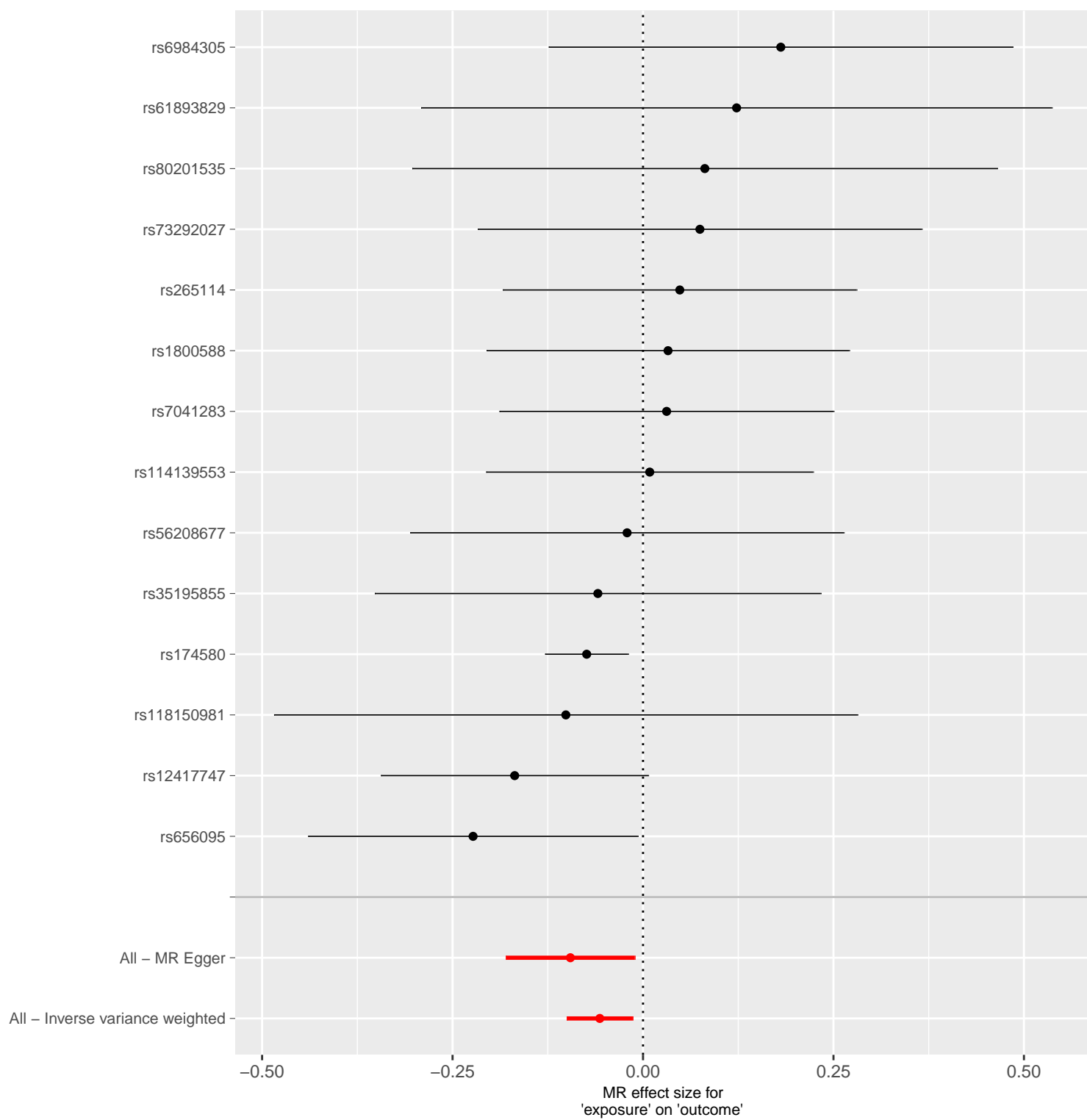

Supplement: Supplementary file 4 — Supplementary Material 4. [file 12944_2024_2103_MOESM4_ESM.zip › sFigure3∩╝êlipidomes-ER-BC∩╝ë/GCST90277311/forest.pdf]

# MR Method

- Inverse variance weighted
- MR Egger

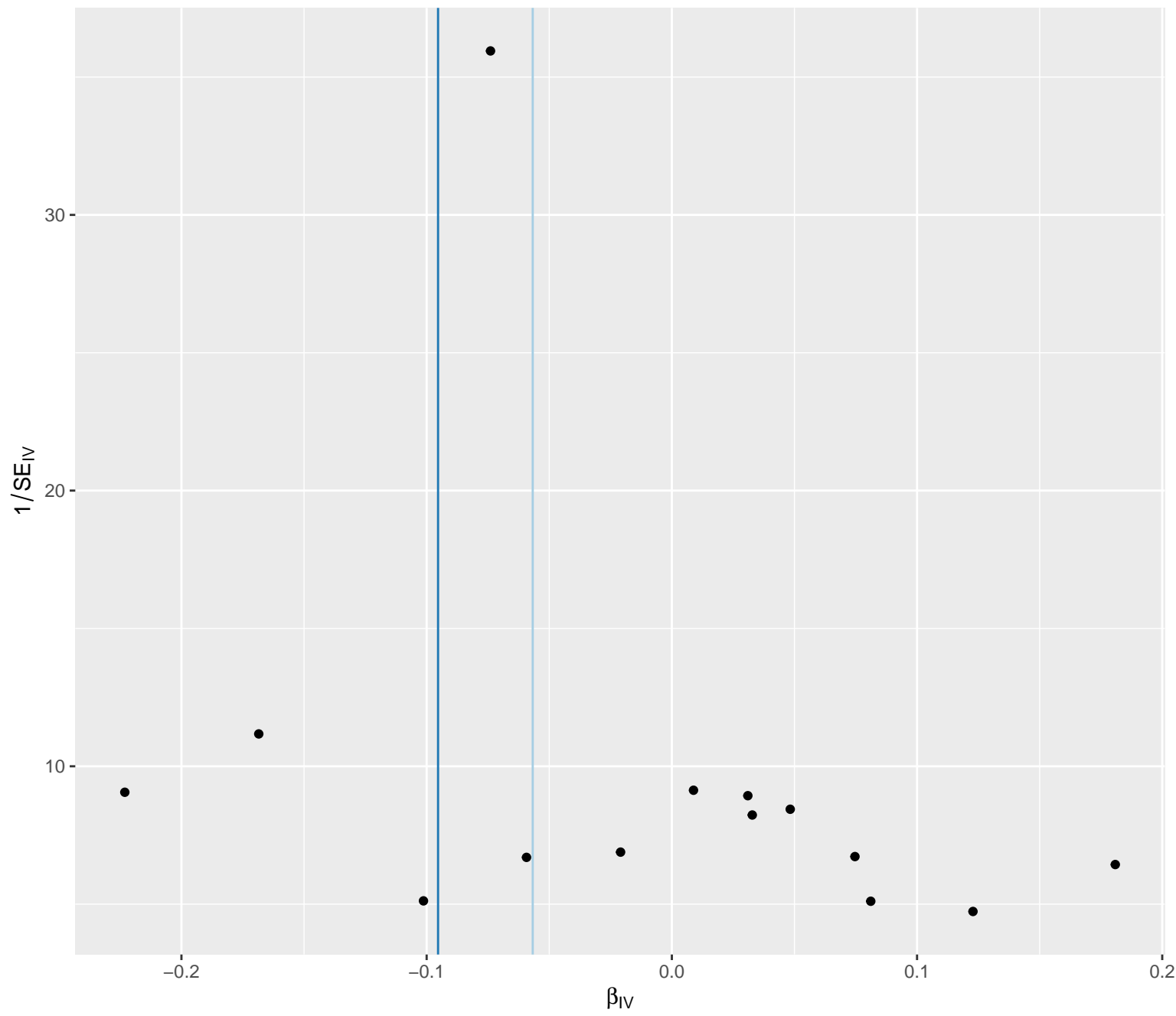

Supplement: Supplementary file 4 — Supplementary Material 4. [file 12944_2024_2103_MOESM4_ESM.zip › sFigure3∩╝êlipidomes-ER-BC∩╝ë/GCST90277311/funnelplot.pdf]

# MR Test

- Inverse variance weighted
- MR Egger
- Simple mode
- Weighted median
- Weighted mode

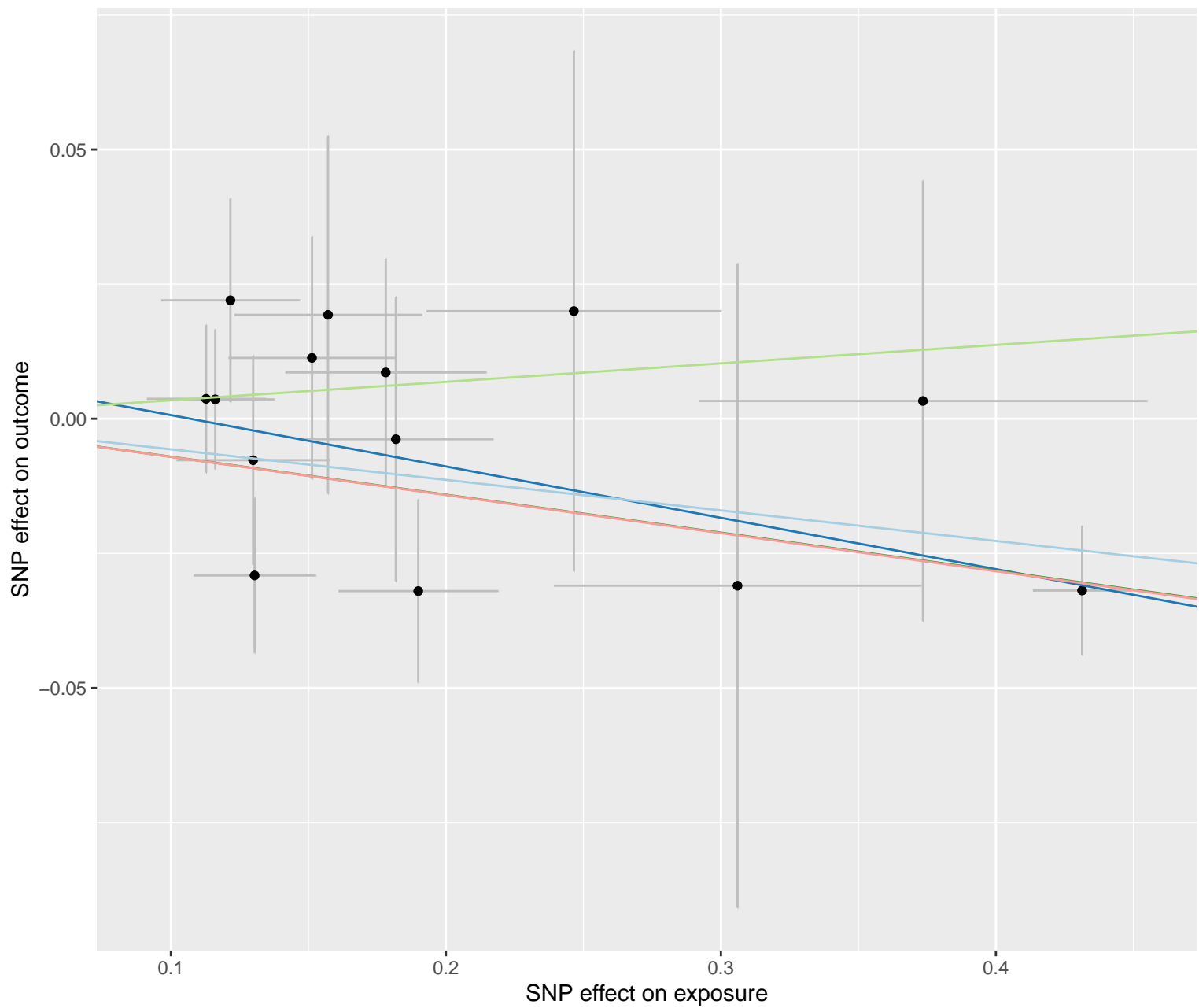

Supplement: Supplementary file 4 — Supplementary Material 4. [file 12944_2024_2103_MOESM4_ESM.zip › sFigure3∩╝êlipidomes-ER-BC∩╝ë/GCST90277311/scatter.pdf]

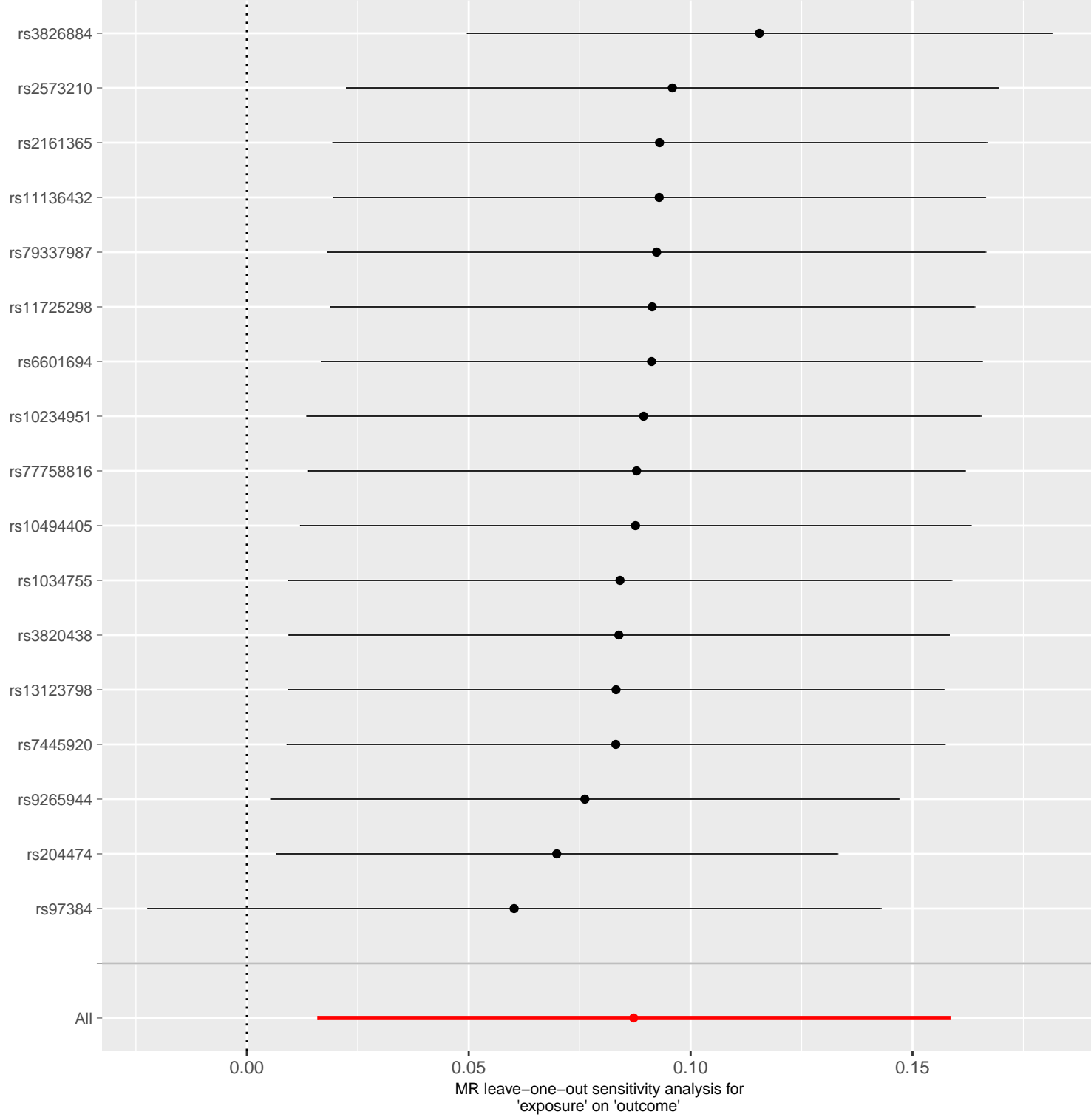

Supplement: Supplementary file 4 — Supplementary Material 4. [file 12944_2024_2103_MOESM4_ESM.zip › sFigure3∩╝êlipidomes-ER-BC∩╝ë/GCST90277351/sensitivity-analysis.pdf]

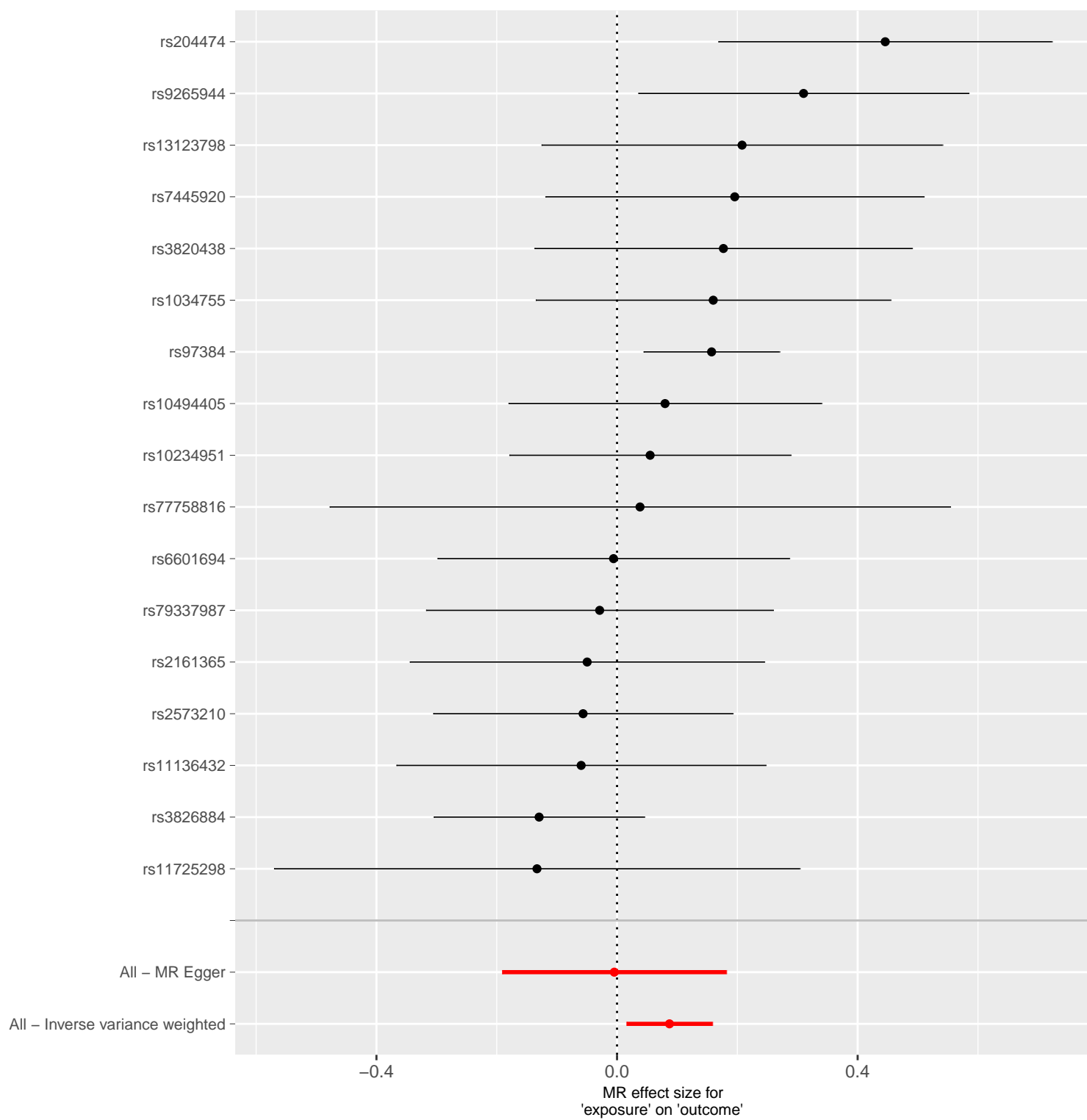

Supplement: Supplementary file 4 — Supplementary Material 4. [file 12944_2024_2103_MOESM4_ESM.zip › sFigure3∩╝êlipidomes-ER-BC∩╝ë/GCST90277351/forest.pdf]

# MR Method

- Inverse variance weighted
- MR Egger

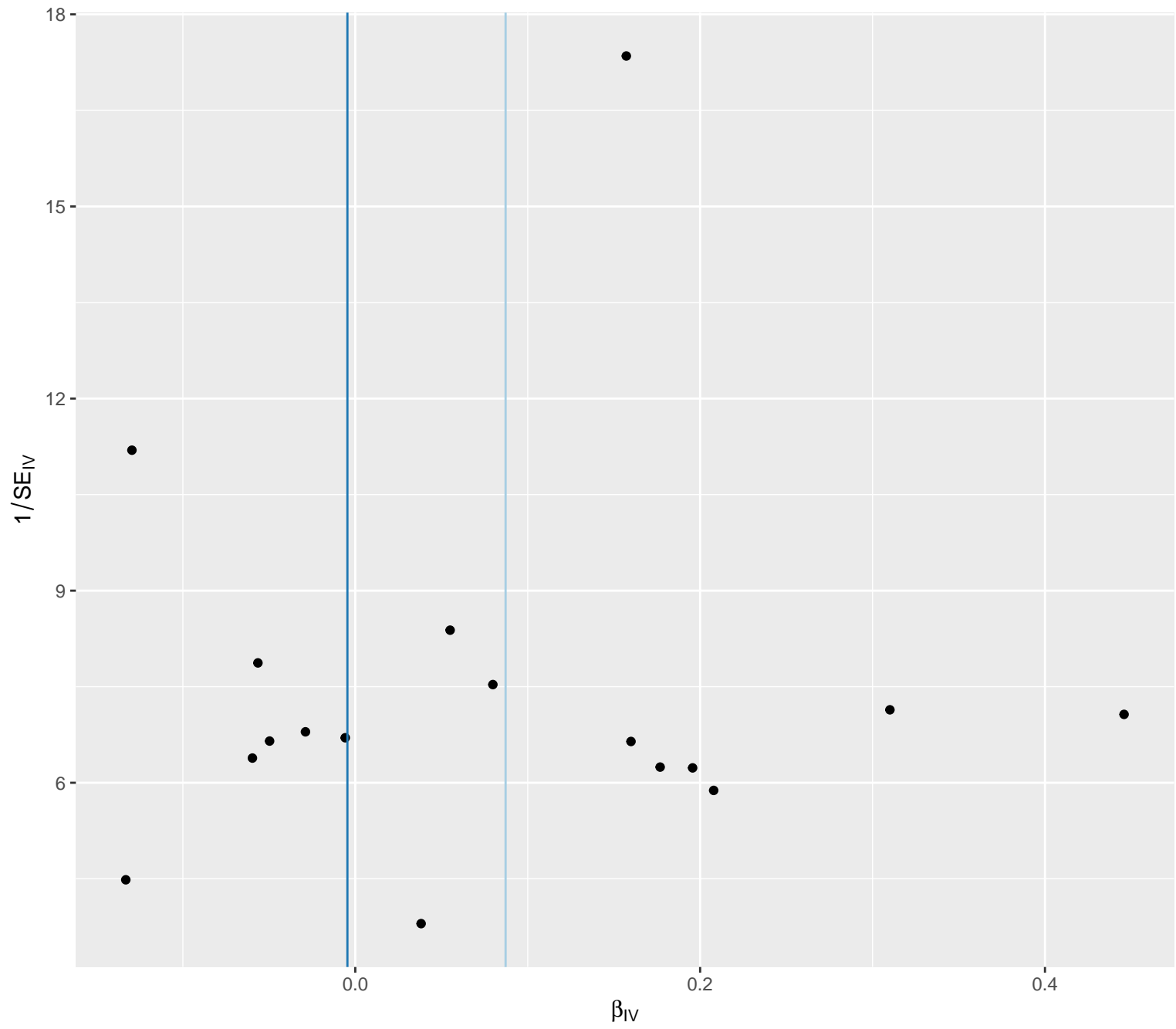

Supplement: Supplementary file 4 — Supplementary Material 4. [file 12944_2024_2103_MOESM4_ESM.zip › sFigure3∩╝êlipidomes-ER-BC∩╝ë/GCST90277351/funnelplot.pdf]

# MR Test

- Inverse variance weighted
- MR Egger
- Simple mode
- Weighted median
- Weighted mode

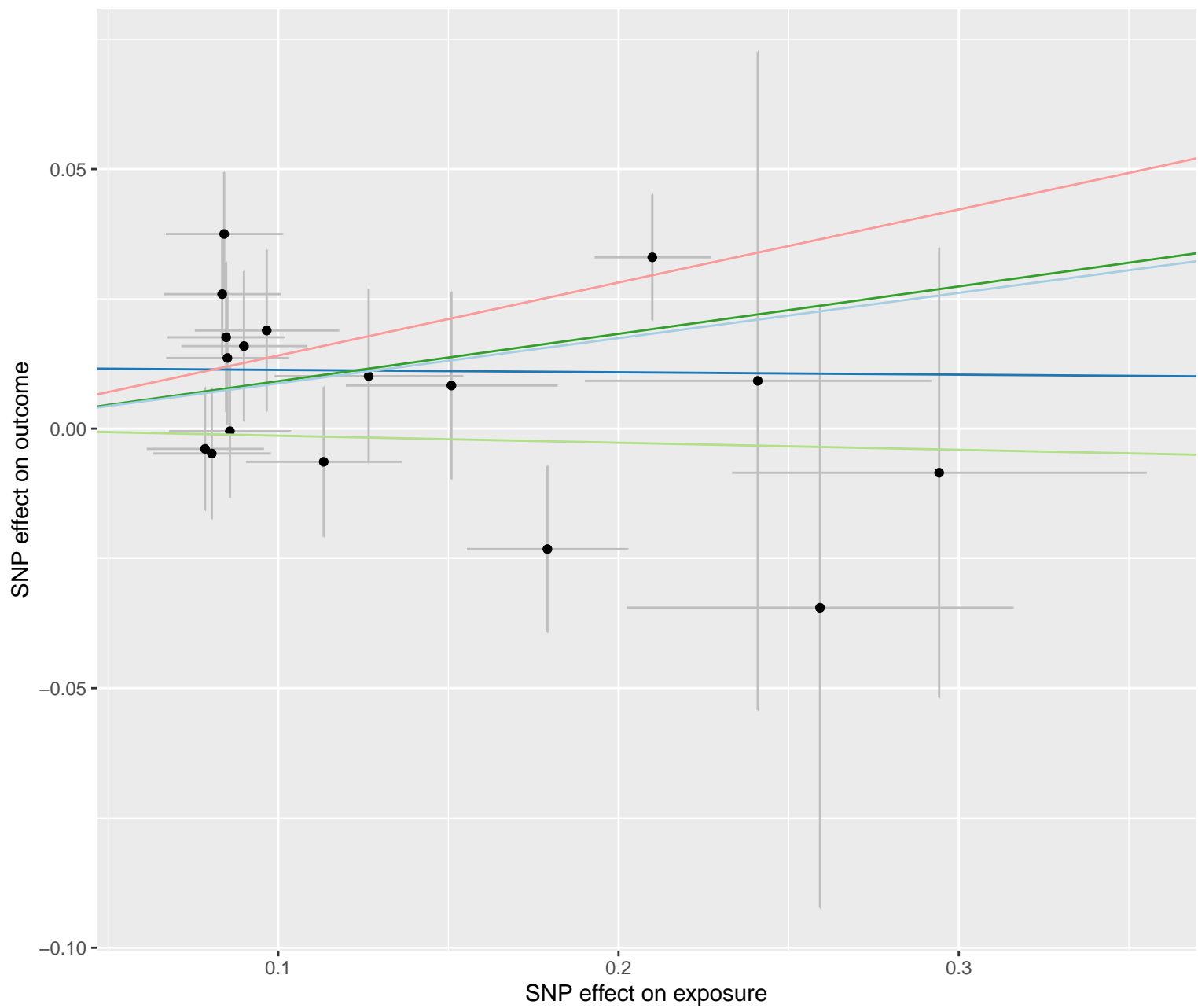

Supplement: Supplementary file 4 — Supplementary Material 4. [file 12944_2024_2103_MOESM4_ESM.zip › sFigure3∩╝êlipidomes-ER-BC∩╝ë/GCST90277351/scatter.pdf]

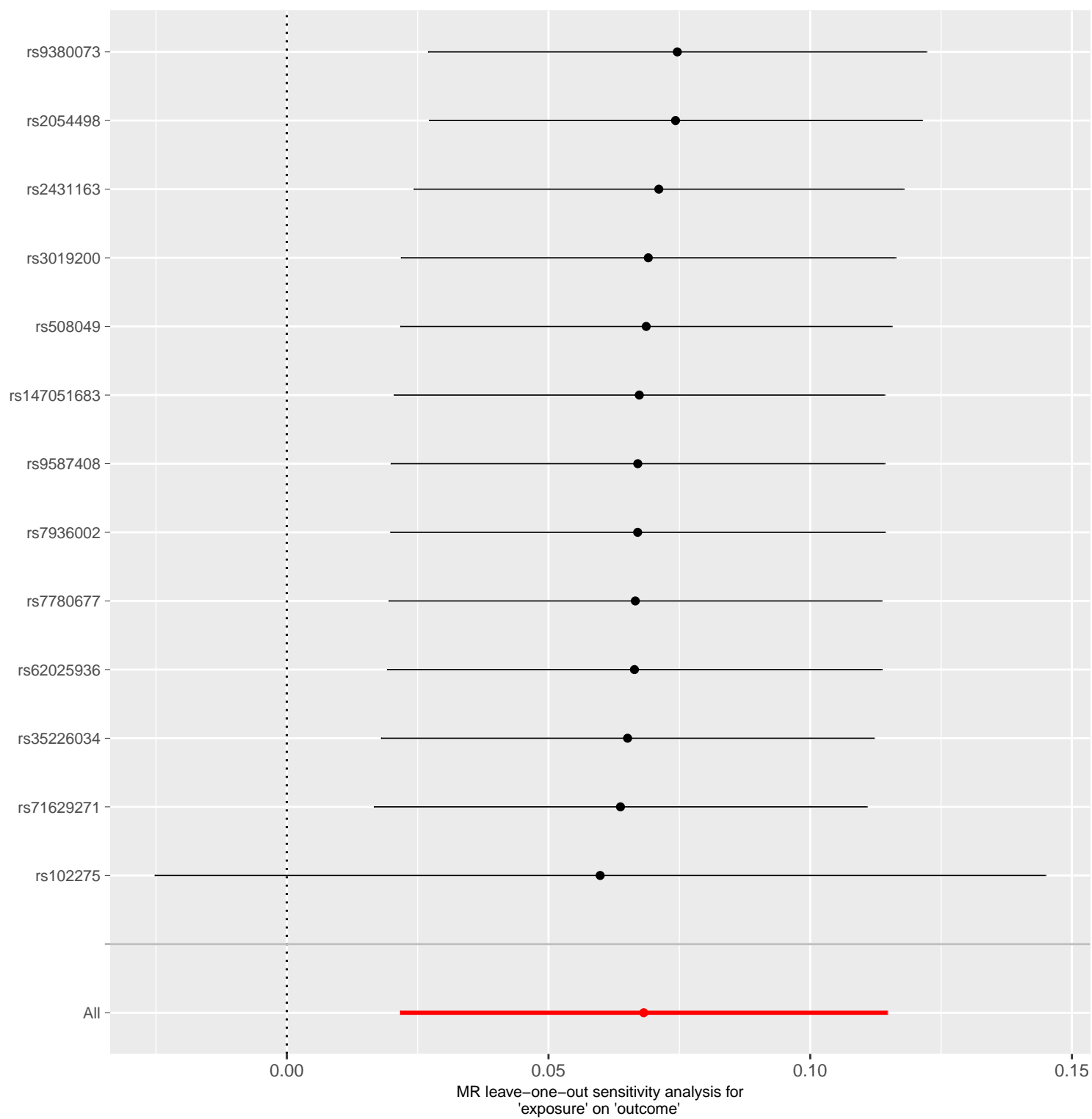

Supplement: Supplementary file 4 — Supplementary Material 4. [file 12944_2024_2103_MOESM4_ESM.zip › sFigure3∩╝êlipidomes-ER-BC∩╝ë/GCST90277305/sensitivity-analysis.pdf]

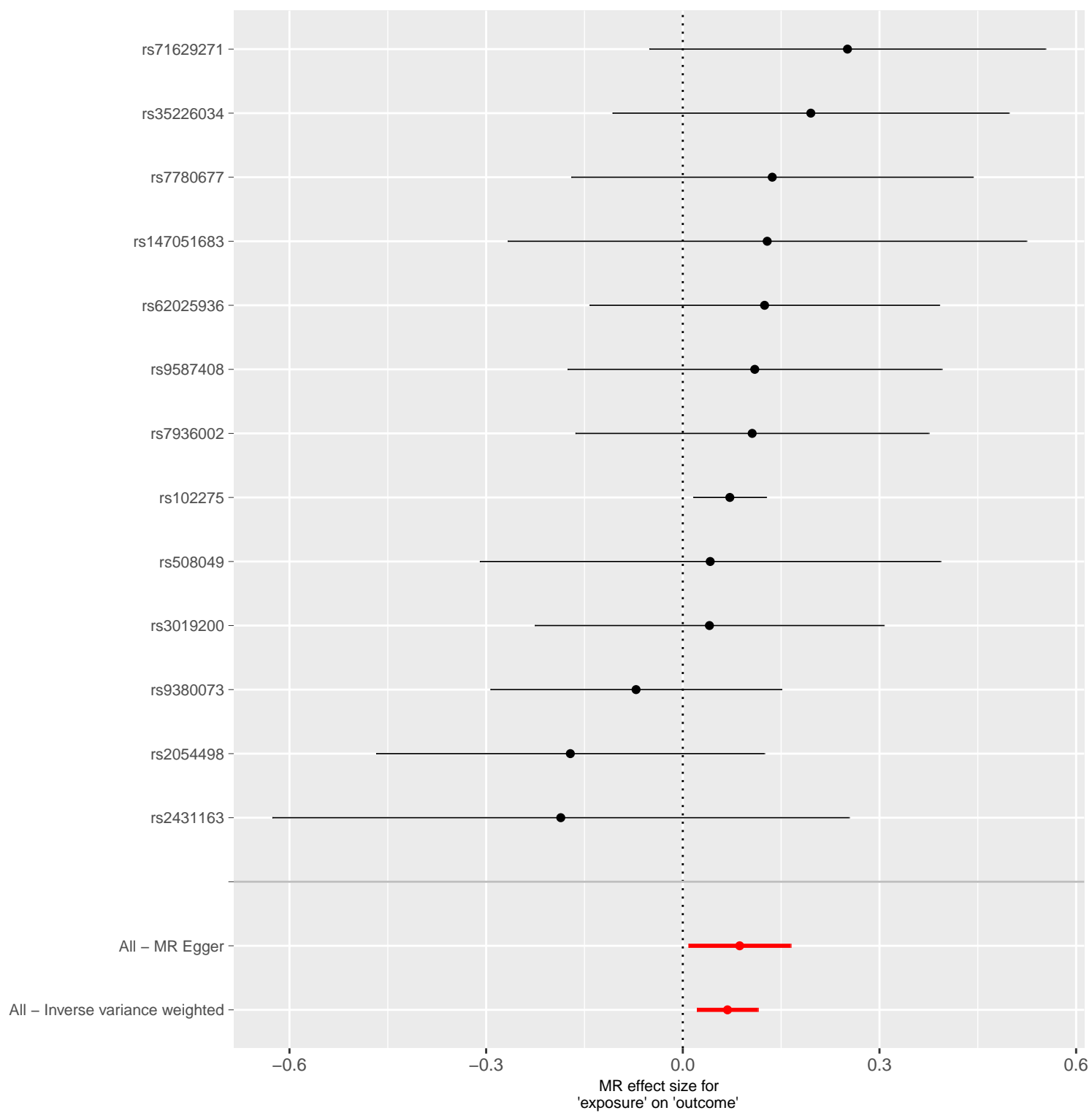

Supplement: Supplementary file 4 — Supplementary Material 4. [file 12944_2024_2103_MOESM4_ESM.zip › sFigure3∩╝êlipidomes-ER-BC∩╝ë/GCST90277305/forest.pdf]

# MR Method

- Inverse variance weighted
- MR Egger

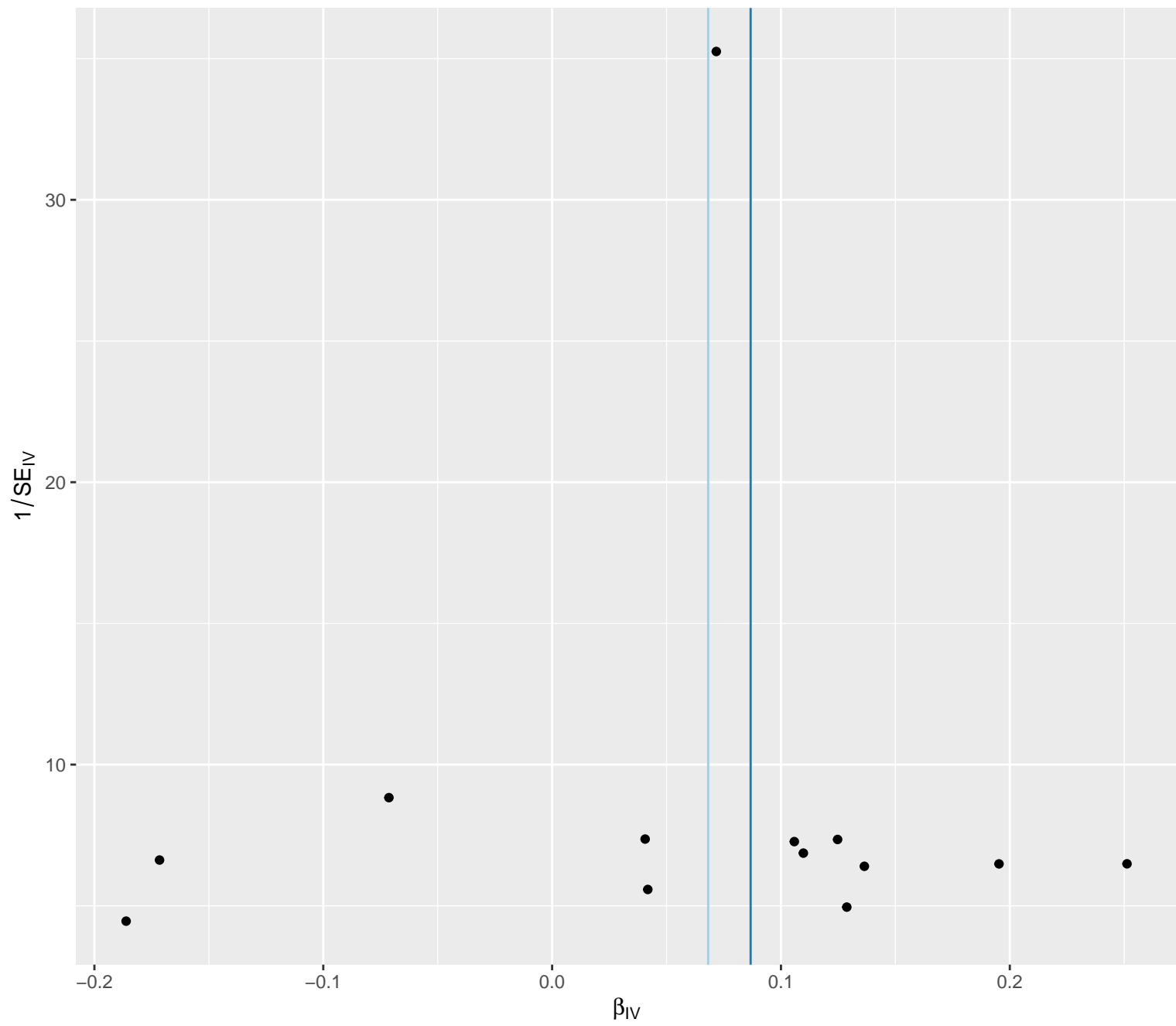

Supplement: Supplementary file 4 — Supplementary Material 4. [file 12944_2024_2103_MOESM4_ESM.zip › sFigure3∩╝êlipidomes-ER-BC∩╝ë/GCST90277305/funnelplot.pdf]

# MR Test

- Inverse variance weighted
- MR Egger
- Simple mode
- Weighted median
- Weighted mode

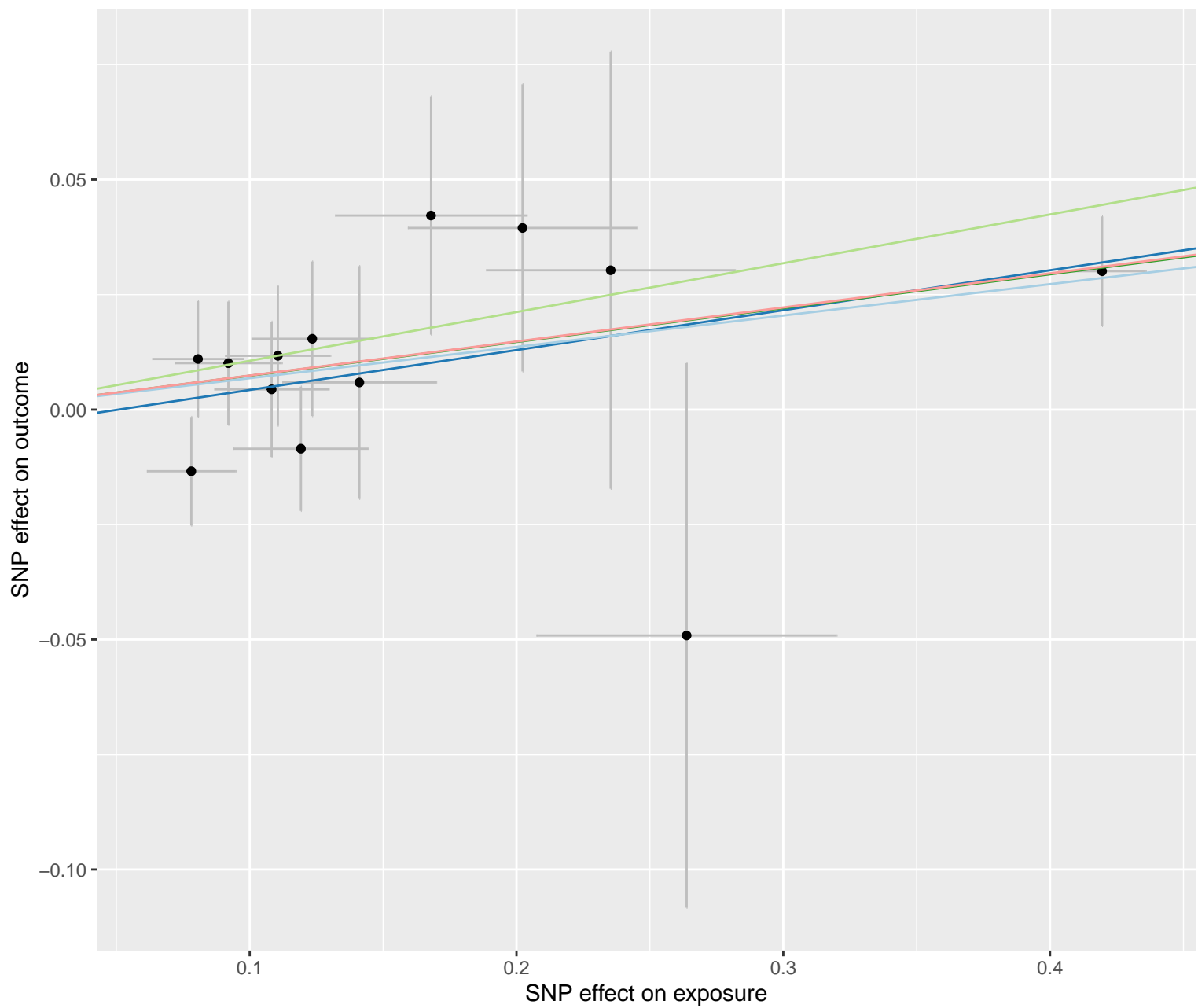

Supplement: Supplementary file 4 — Supplementary Material 4. [file 12944_2024_2103_MOESM4_ESM.zip › sFigure3∩╝êlipidomes-ER-BC∩╝ë/GCST90277305/scatter.pdf]

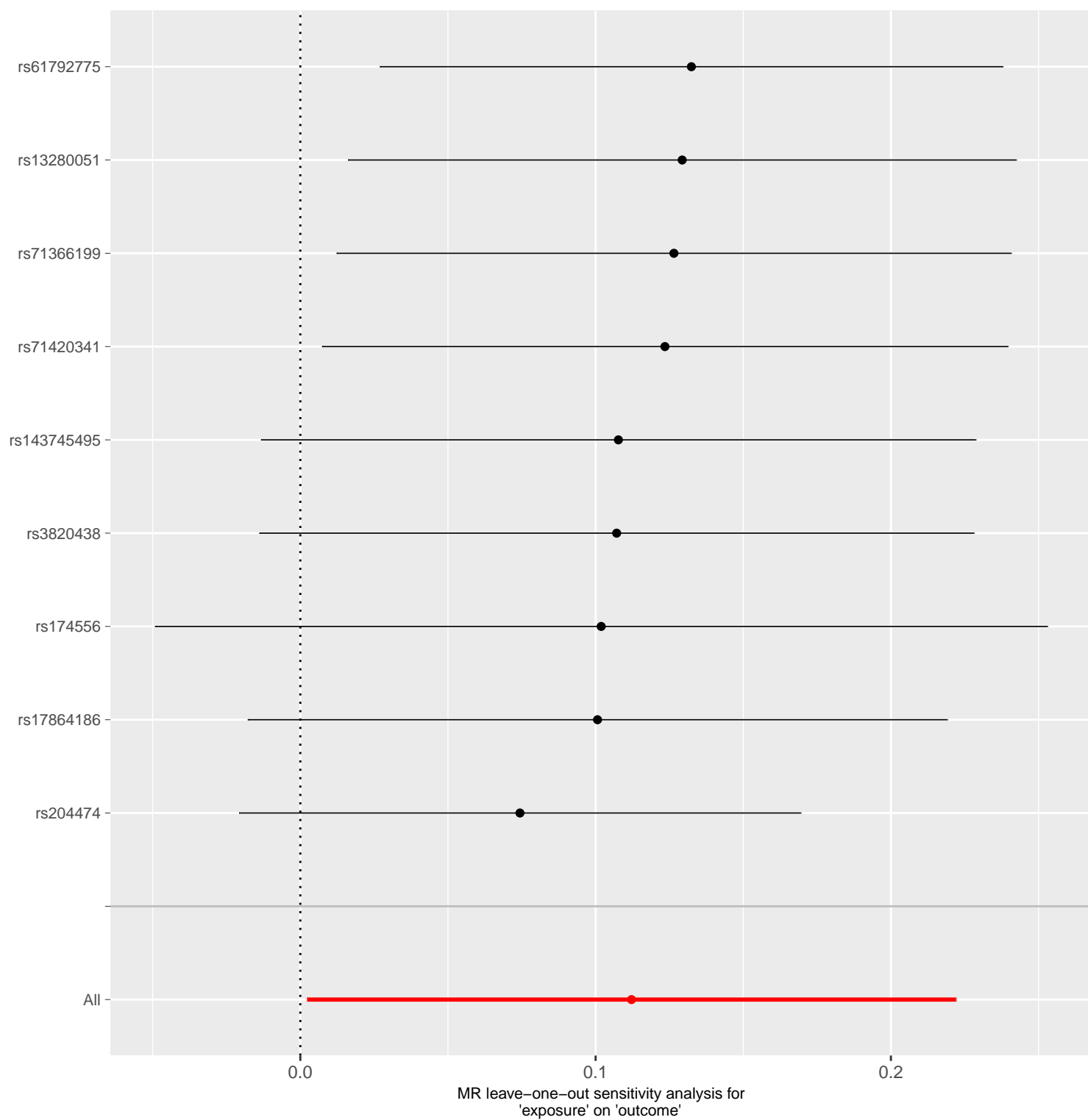

Supplement: Supplementary file 4 — Supplementary Material 4. [file 12944_2024_2103_MOESM4_ESM.zip › sFigure3∩╝êlipidomes-ER-BC∩╝ë/GCST90277357/sensitivity-analysis.pdf]

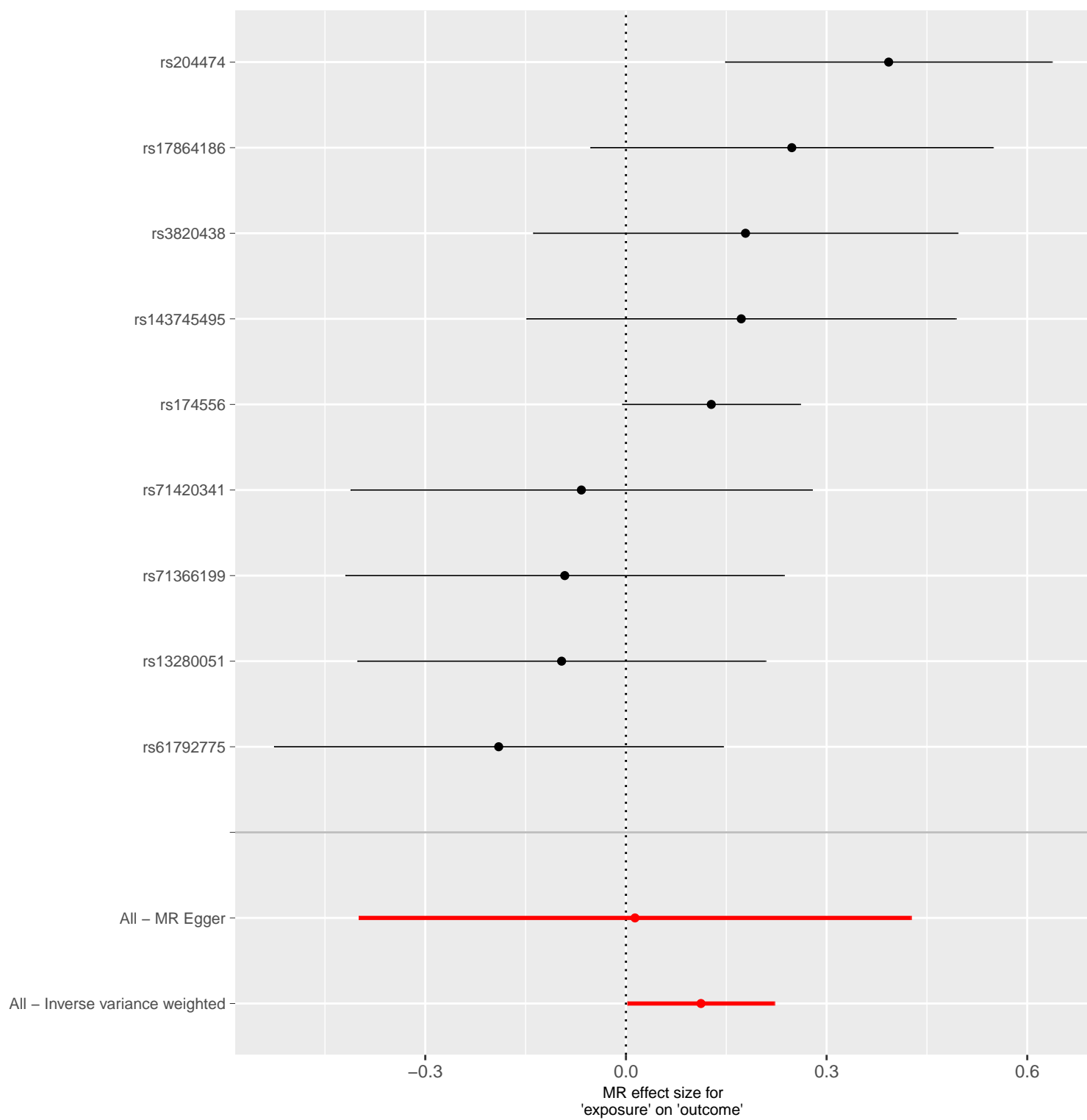

Supplement: Supplementary file 4 — Supplementary Material 4. [file 12944_2024_2103_MOESM4_ESM.zip › sFigure3∩╝êlipidomes-ER-BC∩╝ë/GCST90277357/forest.pdf]

# MR Method

- Inverse variance weighted
- MR Egger

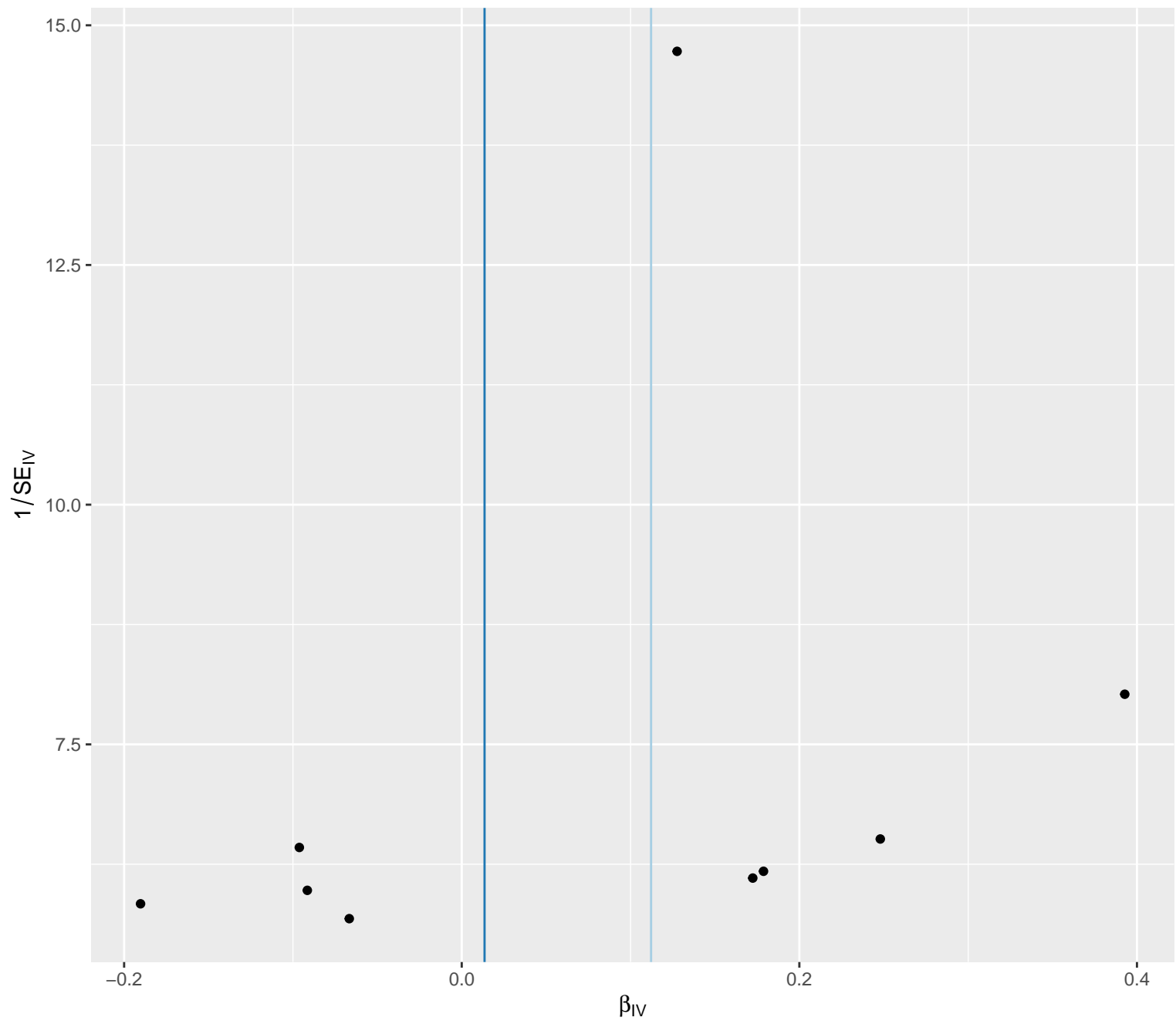

Supplement: Supplementary file 4 — Supplementary Material 4. [file 12944_2024_2103_MOESM4_ESM.zip › sFigure3∩╝êlipidomes-ER-BC∩╝ë/GCST90277357/funnelplot.pdf]

# MR Test

- Inverse variance weighted
- MR Egger
- Simple mode
- Weighted median
- Weighted mode

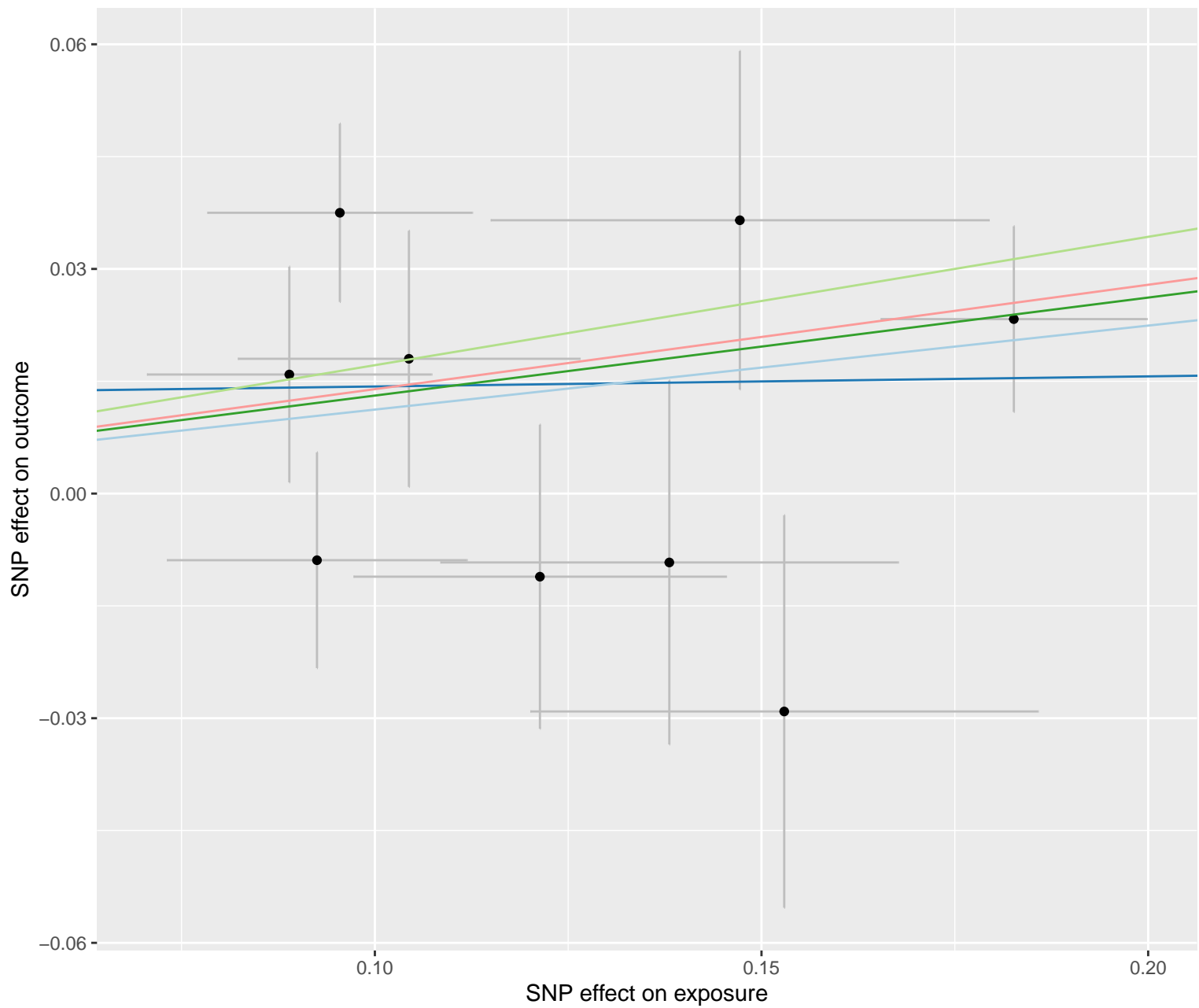

Supplement: Supplementary file 4 — Supplementary Material 4. [file 12944_2024_2103_MOESM4_ESM.zip › sFigure3∩╝êlipidomes-ER-BC∩╝ë/GCST90277357/scatter.pdf]

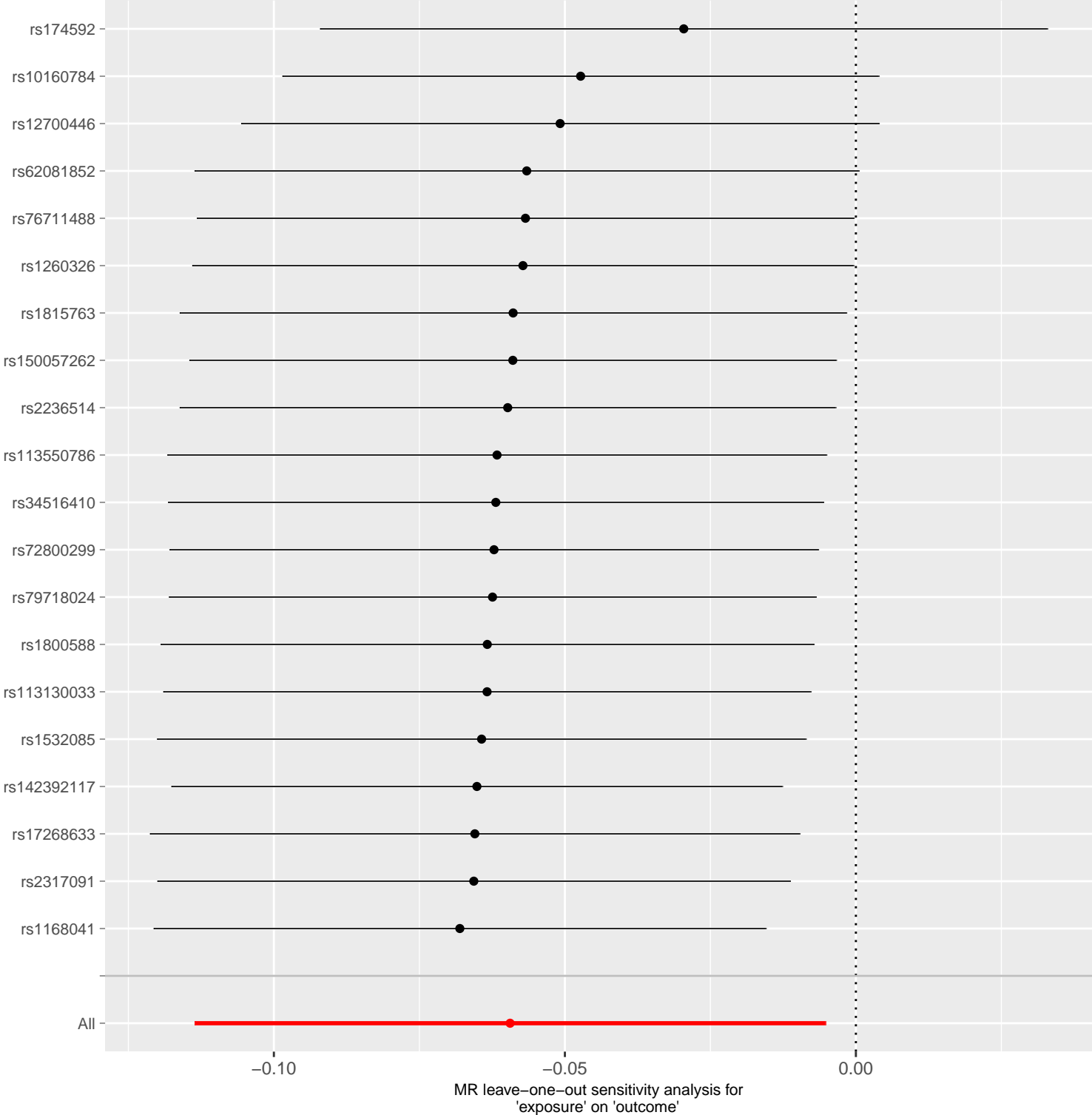

Supplement: Supplementary file 4 — Supplementary Material 4. [file 12944_2024_2103_MOESM4_ESM.zip › sFigure3∩╝êlipidomes-ER-BC∩╝ë/GCST90277285/sensitivity-analysis.pdf]

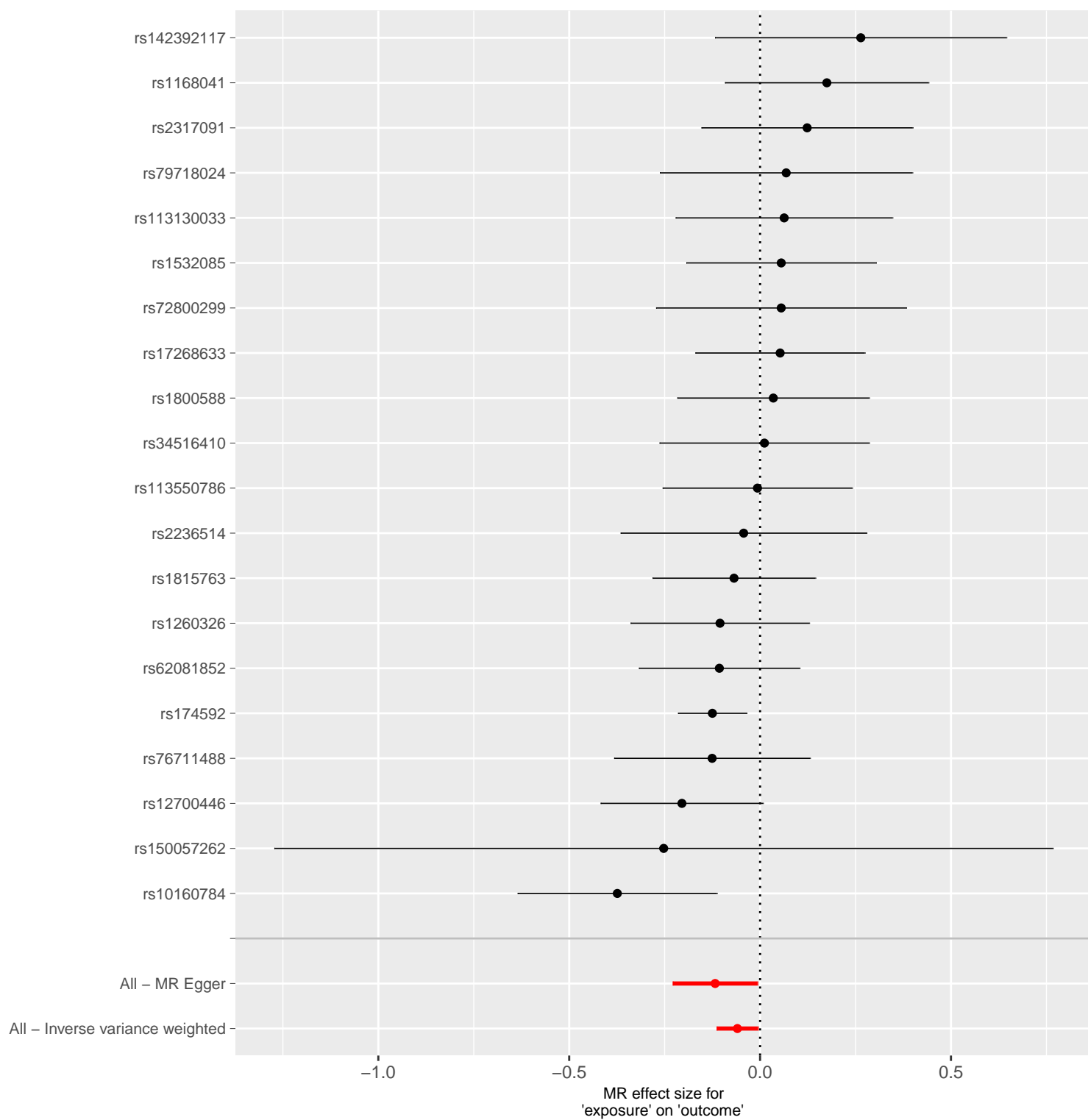

Supplement: Supplementary file 4 — Supplementary Material 4. [file 12944_2024_2103_MOESM4_ESM.zip › sFigure3∩╝êlipidomes-ER-BC∩╝ë/GCST90277285/forest.pdf]

# MR Method

- Inverse variance weighted
- MR Egger

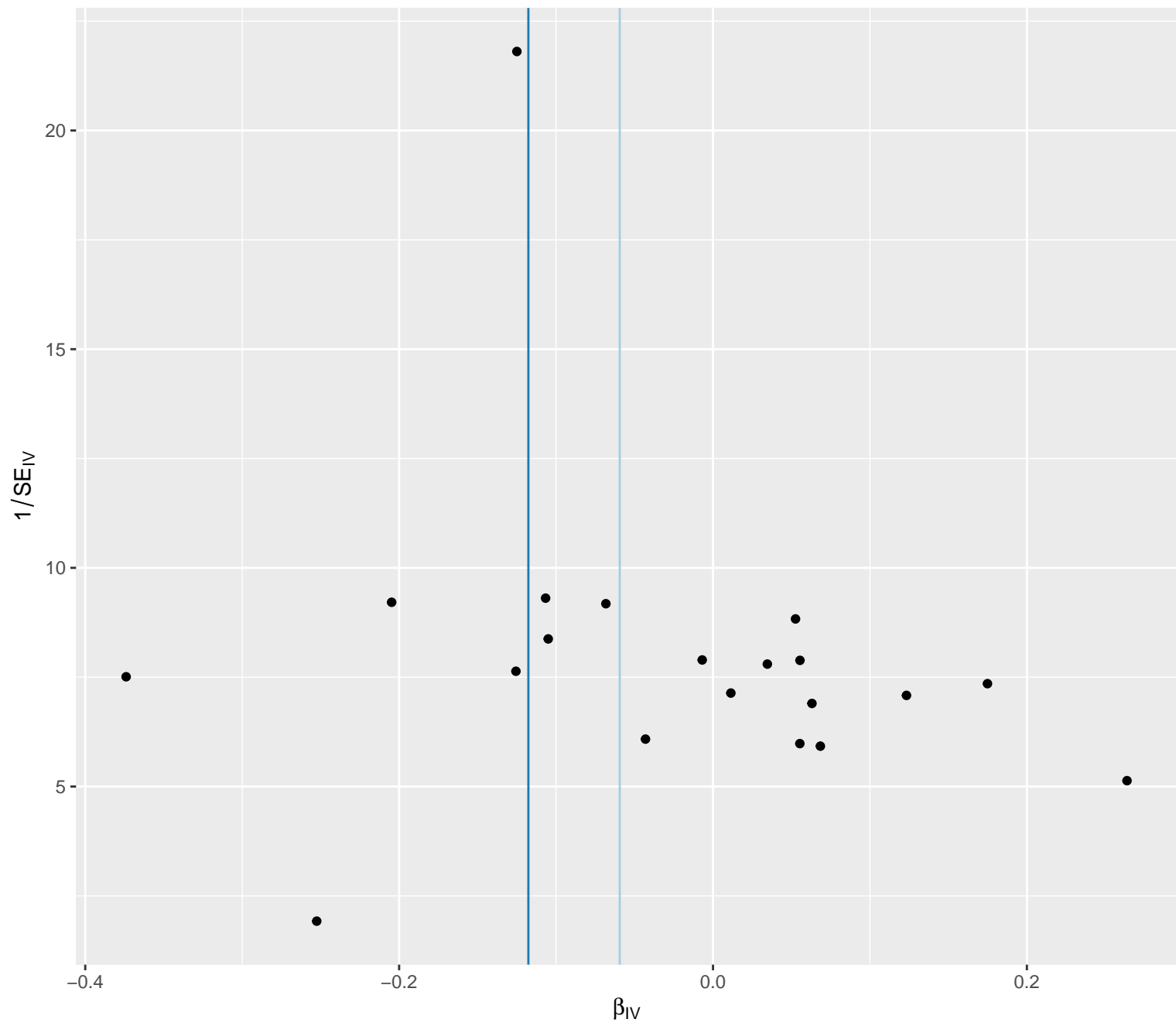

Supplement: Supplementary file 4 — Supplementary Material 4. [file 12944_2024_2103_MOESM4_ESM.zip › sFigure3∩╝êlipidomes-ER-BC∩╝ë/GCST90277285/funnelplot.pdf]

# MR Test

- Inverse variance weighted
- MR Egger
- Simple mode
- Weighted median
- Weighted mode

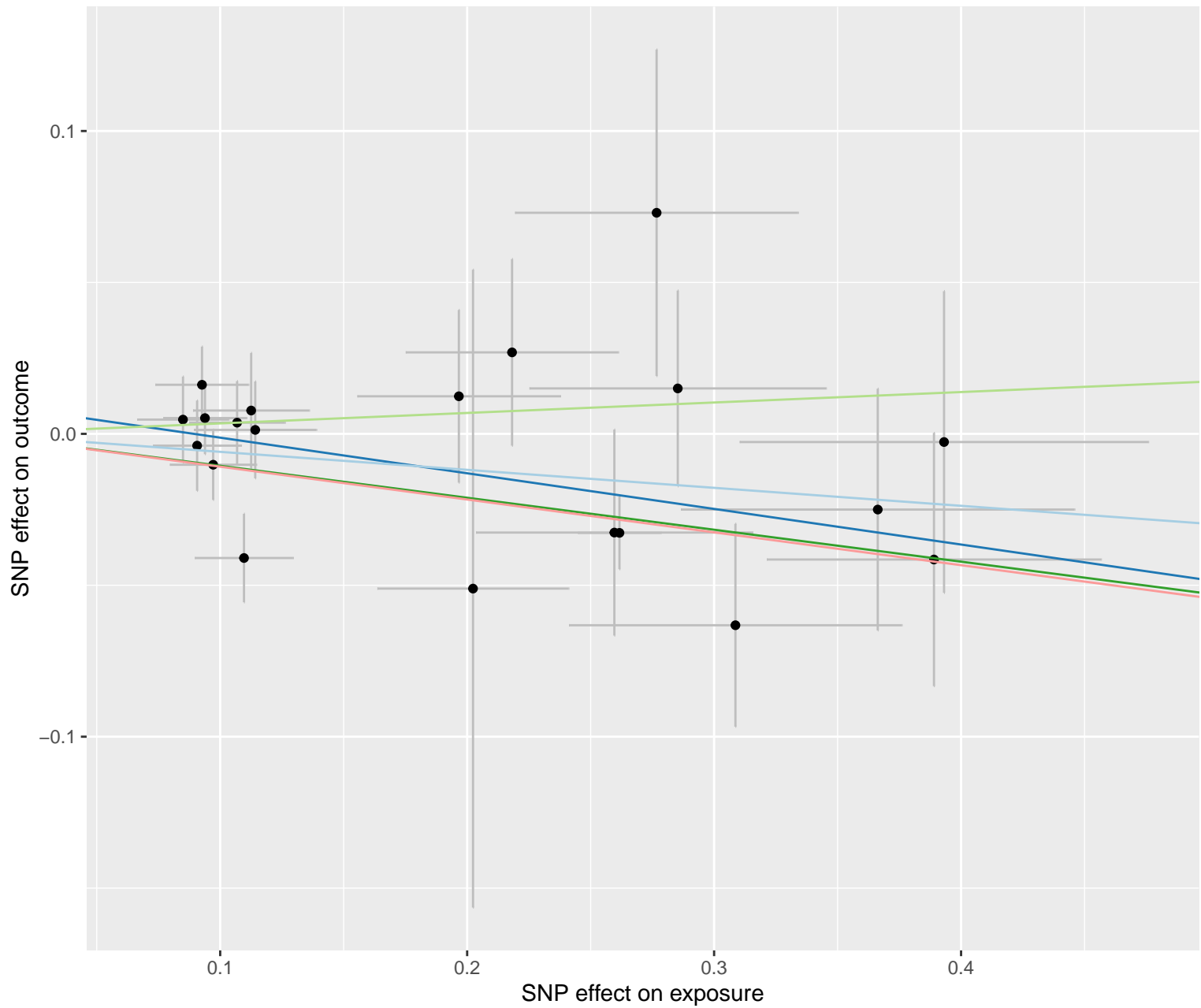

Supplement: Supplementary file 4 — Supplementary Material 4. [file 12944_2024_2103_MOESM4_ESM.zip › sFigure3∩╝êlipidomes-ER-BC∩╝ë/GCST90277285/scatter.pdf]

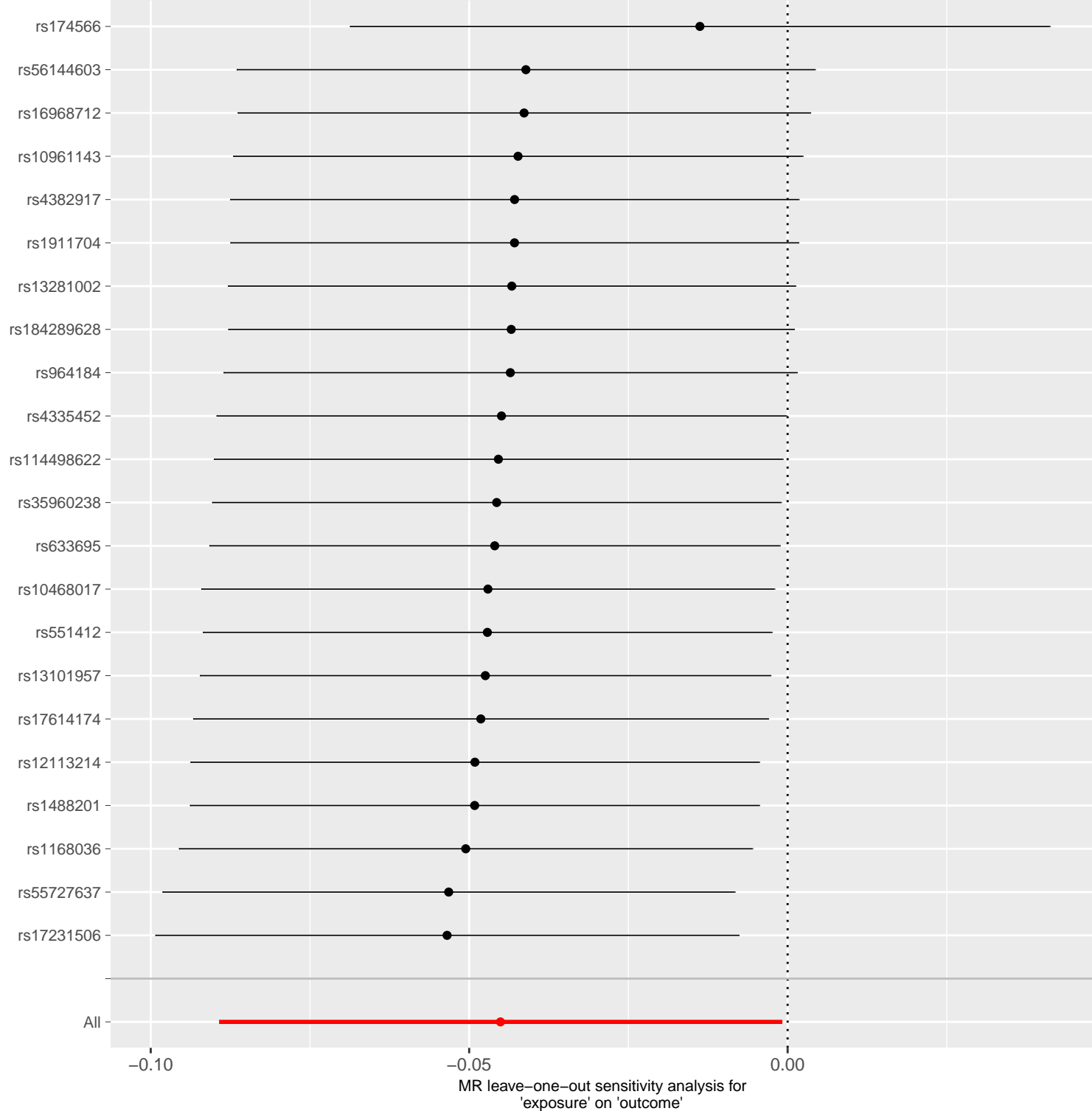

Supplement: Supplementary file 4 — Supplementary Material 4. [file 12944_2024_2103_MOESM4_ESM.zip › sFigure3∩╝êlipidomes-ER-BC∩╝ë/GCST90277276/sensitivity-analysis.pdf]

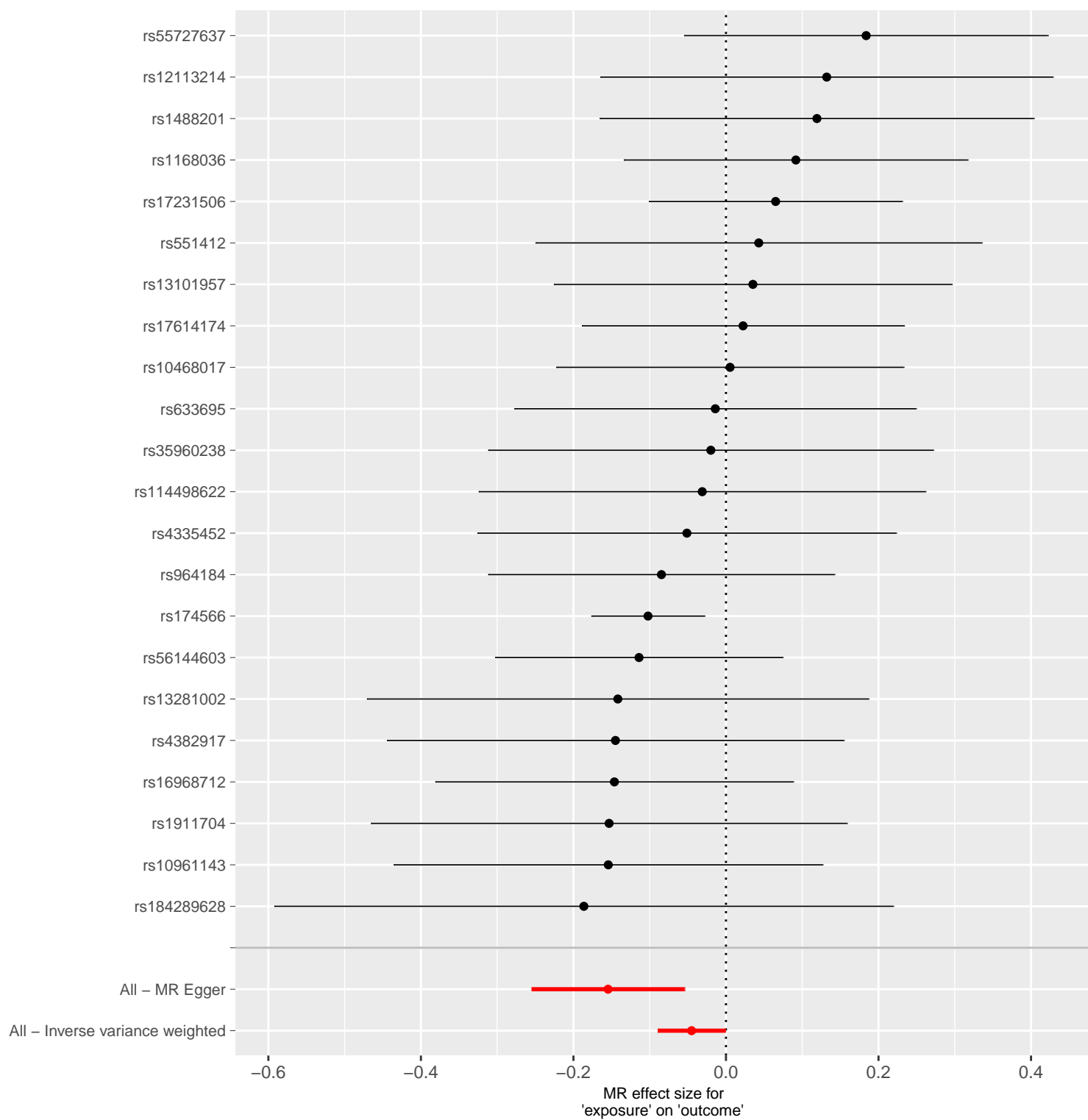

Supplement: Supplementary file 4 — Supplementary Material 4. [file 12944_2024_2103_MOESM4_ESM.zip › sFigure3∩╝êlipidomes-ER-BC∩╝ë/GCST90277276/forest.pdf]

# MR Method

- Inverse variance weighted
- MR Egger

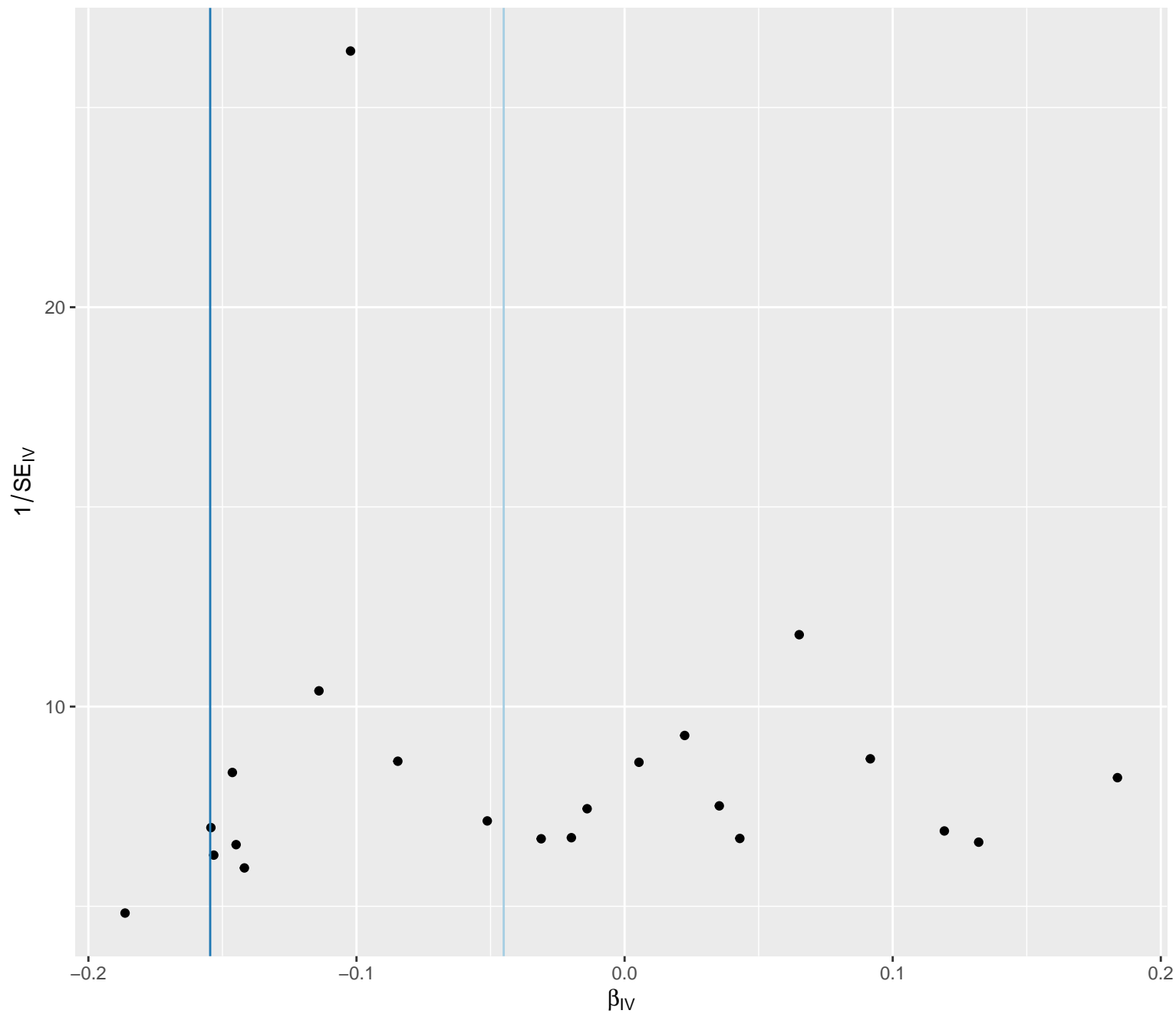

Supplement: Supplementary file 4 — Supplementary Material 4. [file 12944_2024_2103_MOESM4_ESM.zip › sFigure3∩╝êlipidomes-ER-BC∩╝ë/GCST90277276/funnelplot.pdf]

# MR Test

- Inverse variance weighted
- MR Egger
- Simple mode
- Weighted median
- Weighted mode

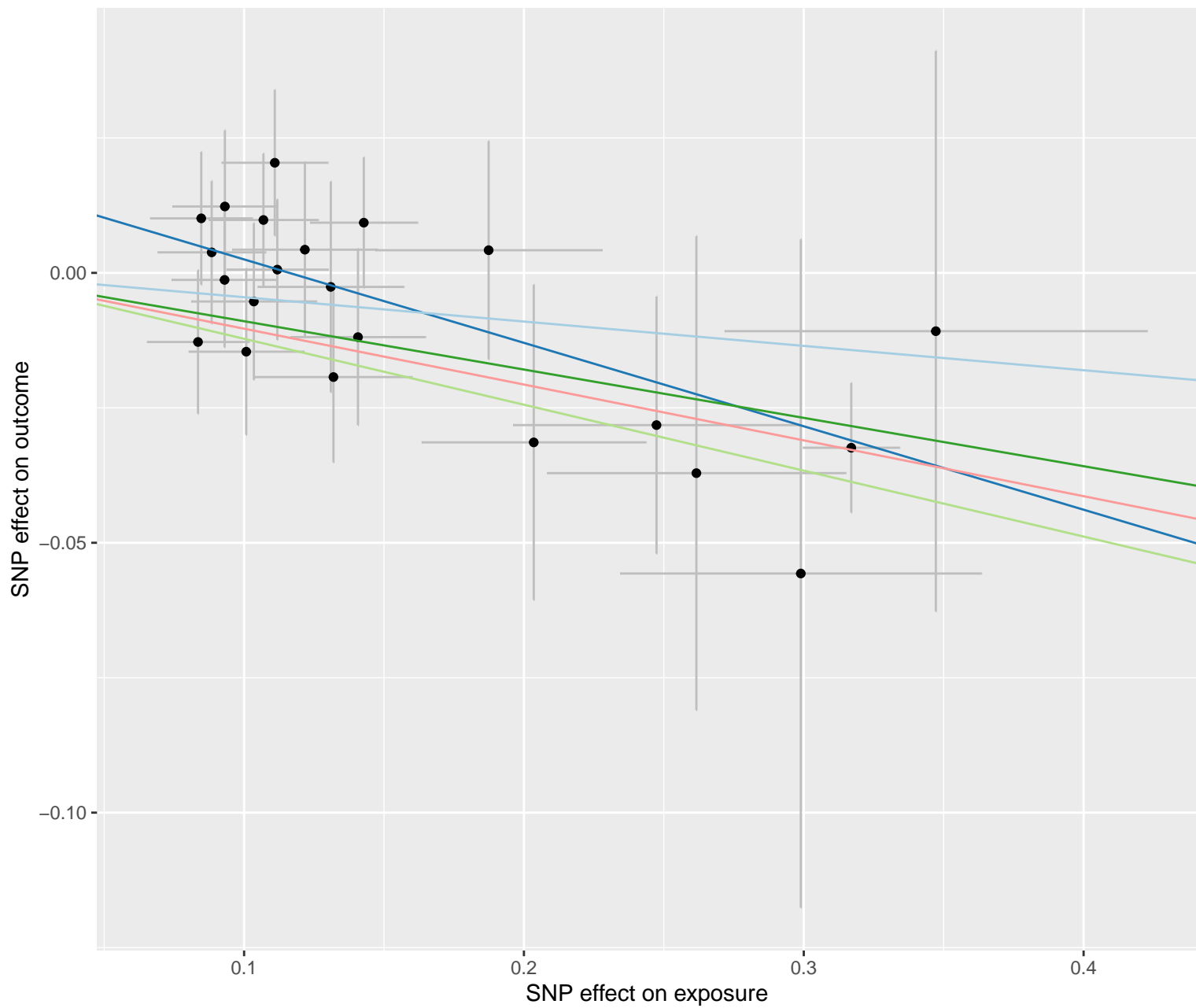

Supplement: Supplementary file 4 — Supplementary Material 4. [file 12944_2024_2103_MOESM4_ESM.zip › sFigure3∩╝êlipidomes-ER-BC∩╝ë/GCST90277276/scatter.pdf]

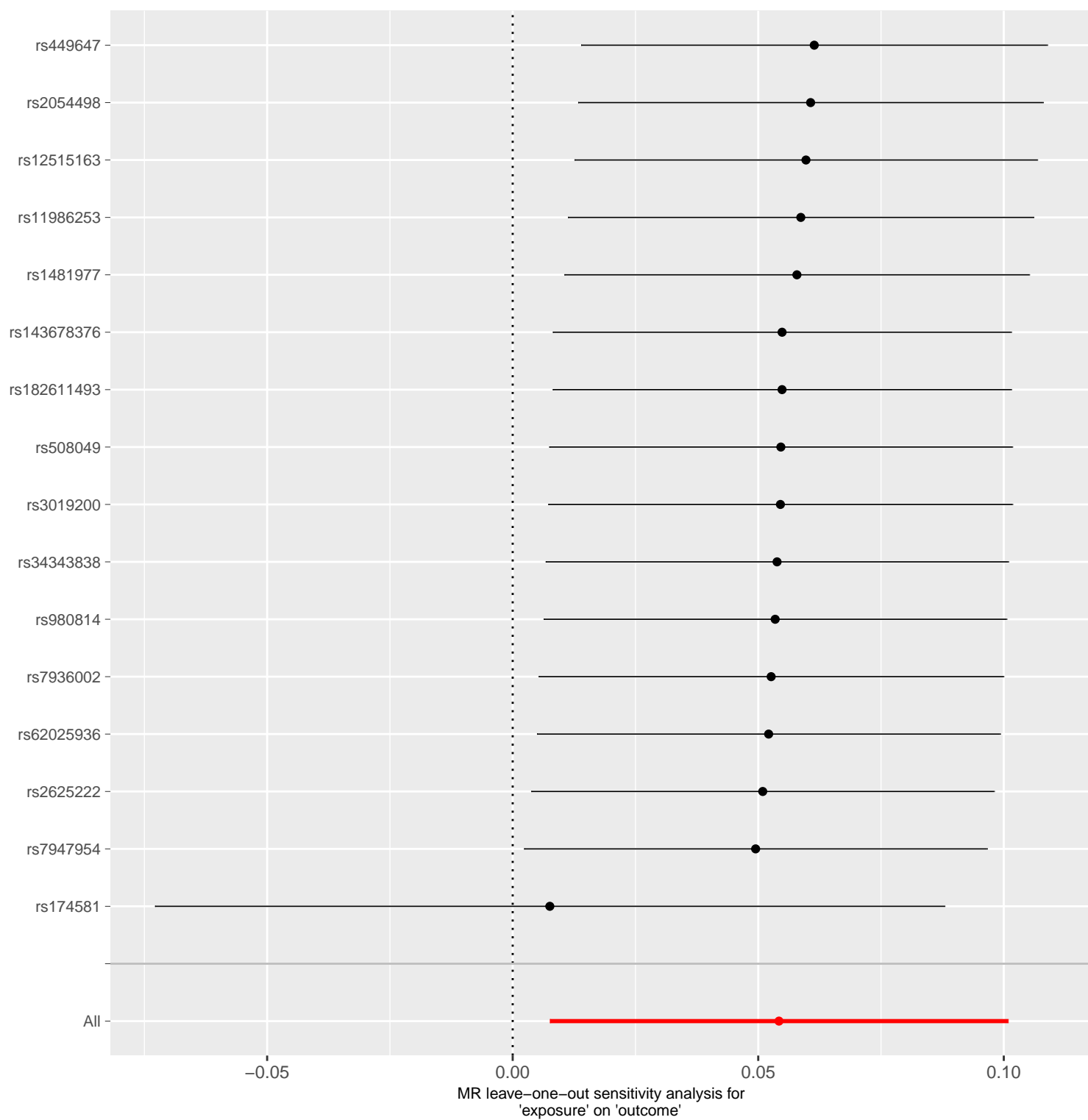

Supplement: Supplementary file 4 — Supplementary Material 4. [file 12944_2024_2103_MOESM4_ESM.zip › sFigure3∩╝êlipidomes-ER-BC∩╝ë/GCST90277251/sensitivity-analysis.pdf]

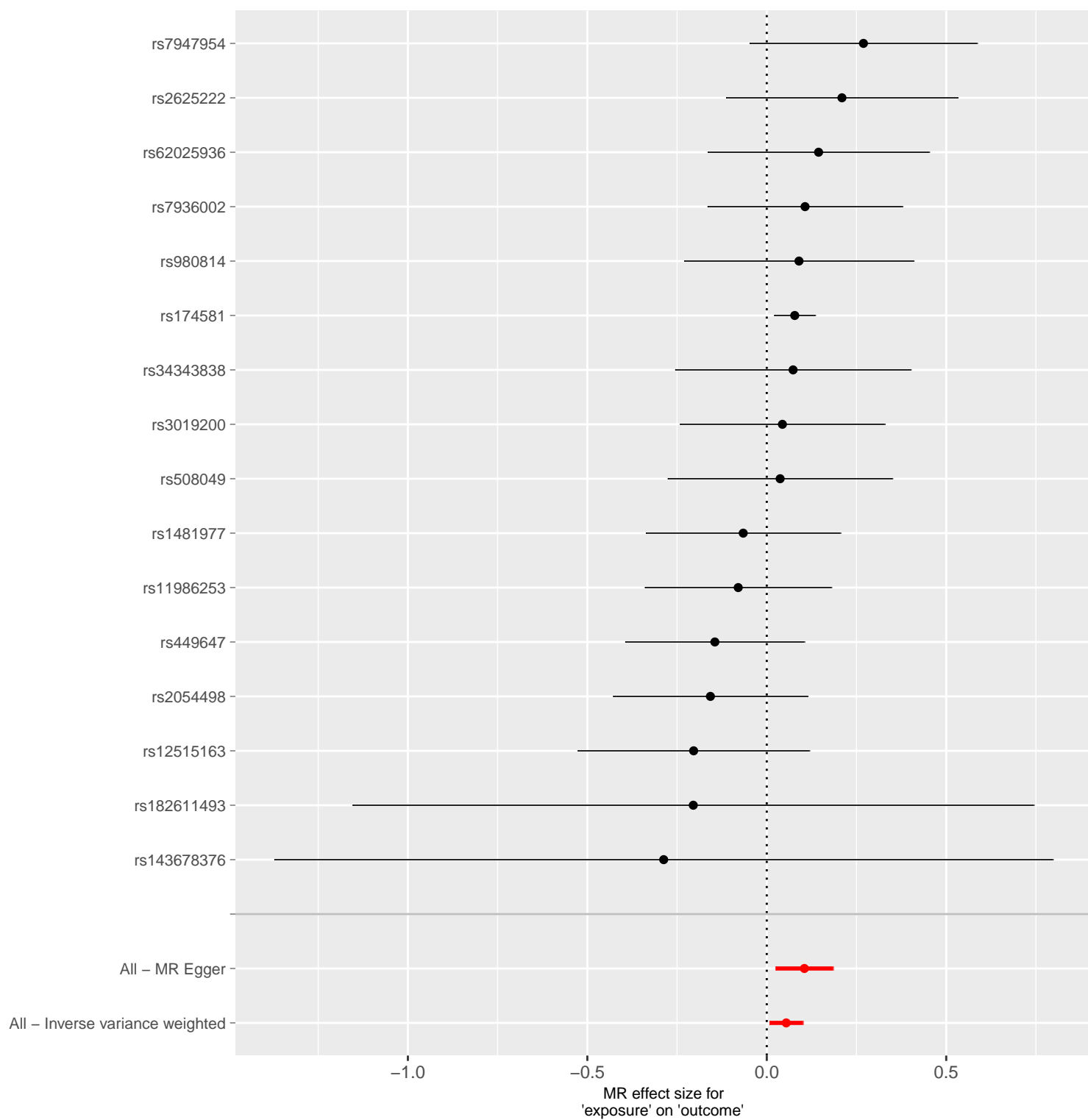

Supplement: Supplementary file 4 — Supplementary Material 4. [file 12944_2024_2103_MOESM4_ESM.zip › sFigure3∩╝êlipidomes-ER-BC∩╝ë/GCST90277251/forest.pdf]

# MR Method

- Inverse variance weighted
- MR Egger

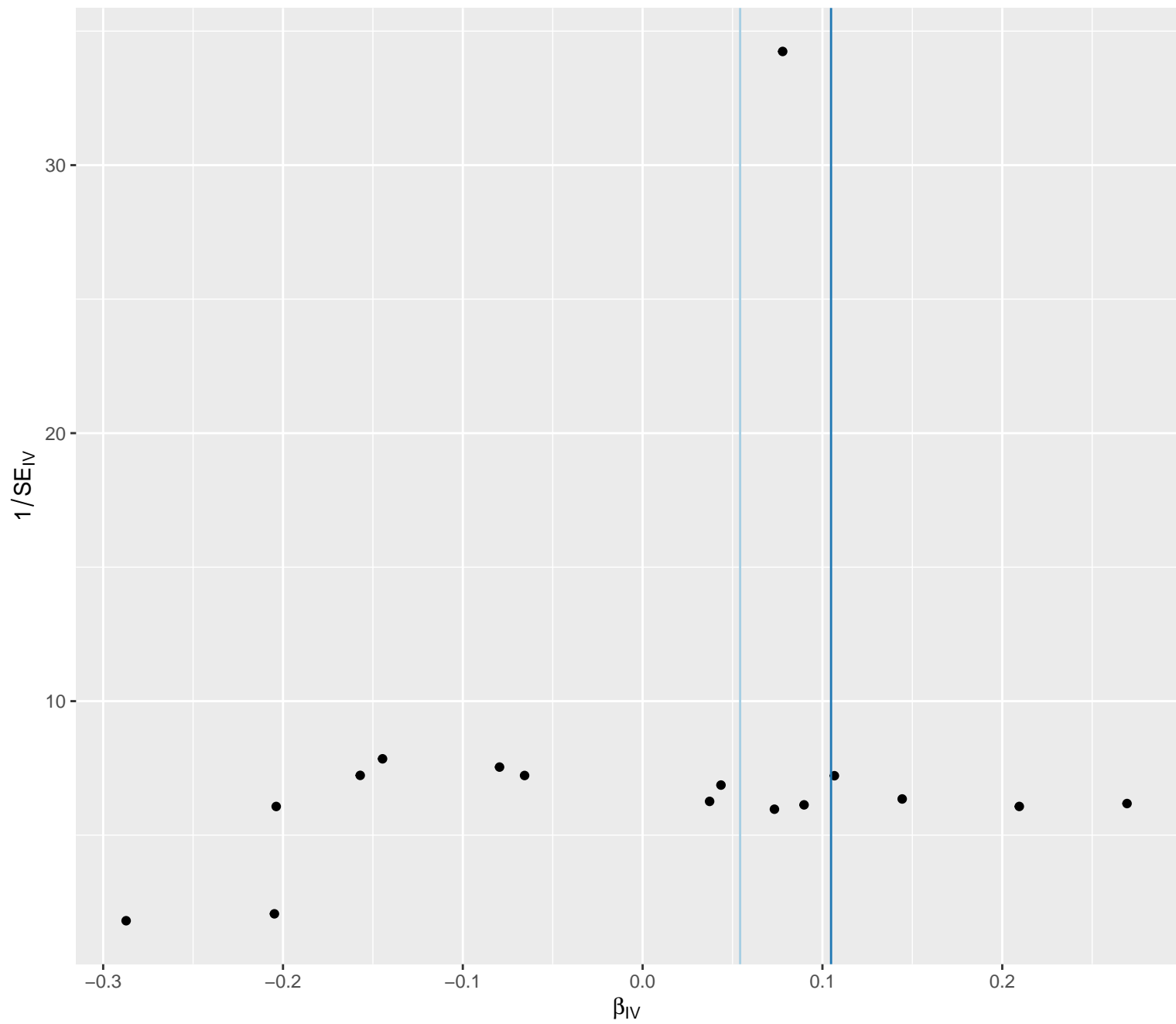

Supplement: Supplementary file 4 — Supplementary Material 4. [file 12944_2024_2103_MOESM4_ESM.zip › sFigure3∩╝êlipidomes-ER-BC∩╝ë/GCST90277251/funnelplot.pdf]

# MR Test

- Inverse variance weighted
- MR Egger
- Simple mode
- Weighted median
- Weighted mode

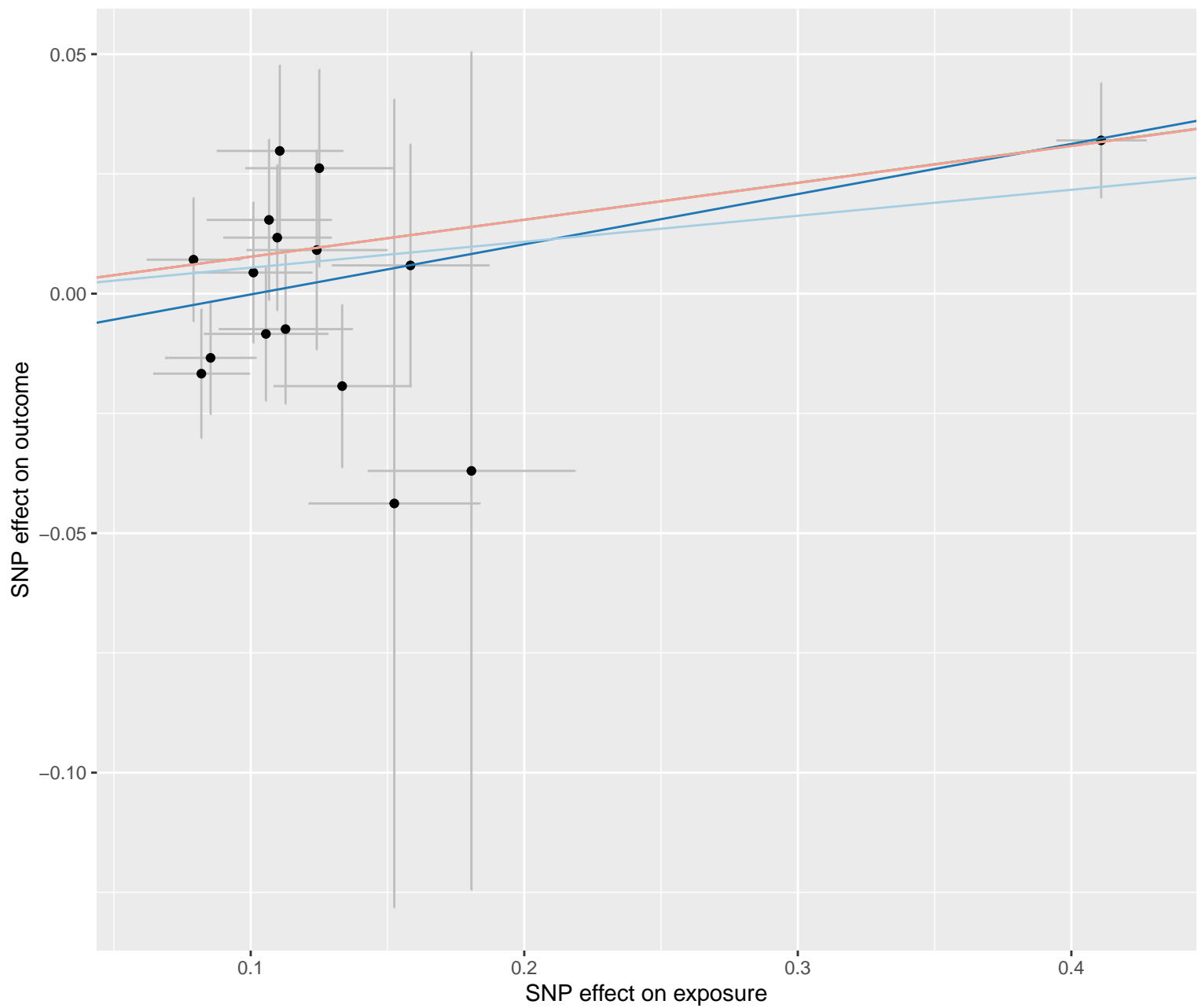

Supplement: Supplementary file 4 — Supplementary Material 4. [file 12944_2024_2103_MOESM4_ESM.zip › sFigure3∩╝êlipidomes-ER-BC∩╝ë/GCST90277251/scatter.pdf]

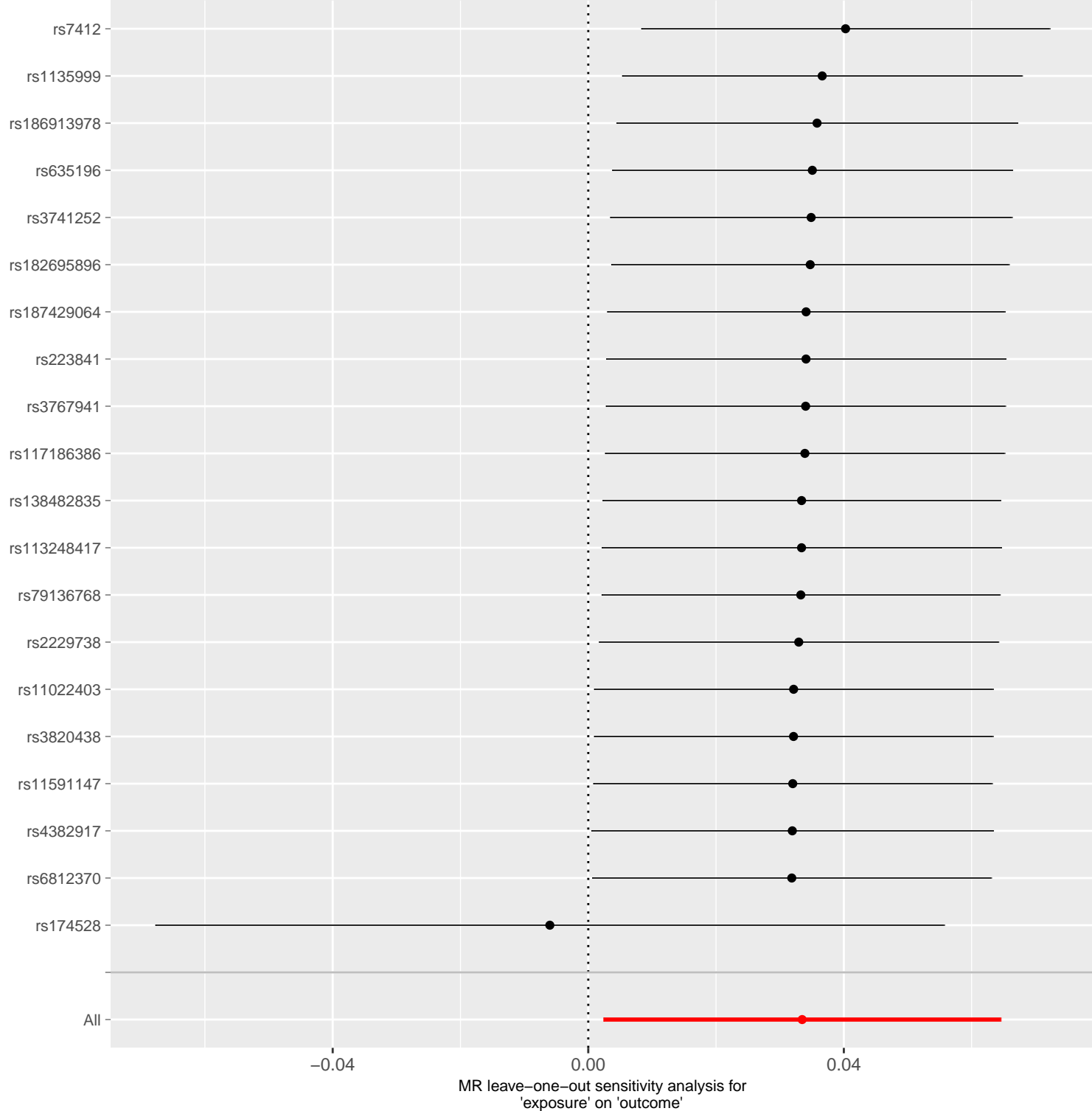

Supplement: Supplementary file 4 — Supplementary Material 4. [file 12944_2024_2103_MOESM4_ESM.zip › sFigure3∩╝êlipidomes-ER-BC∩╝ë/GCST90277250/sensitivity-analysis.pdf]

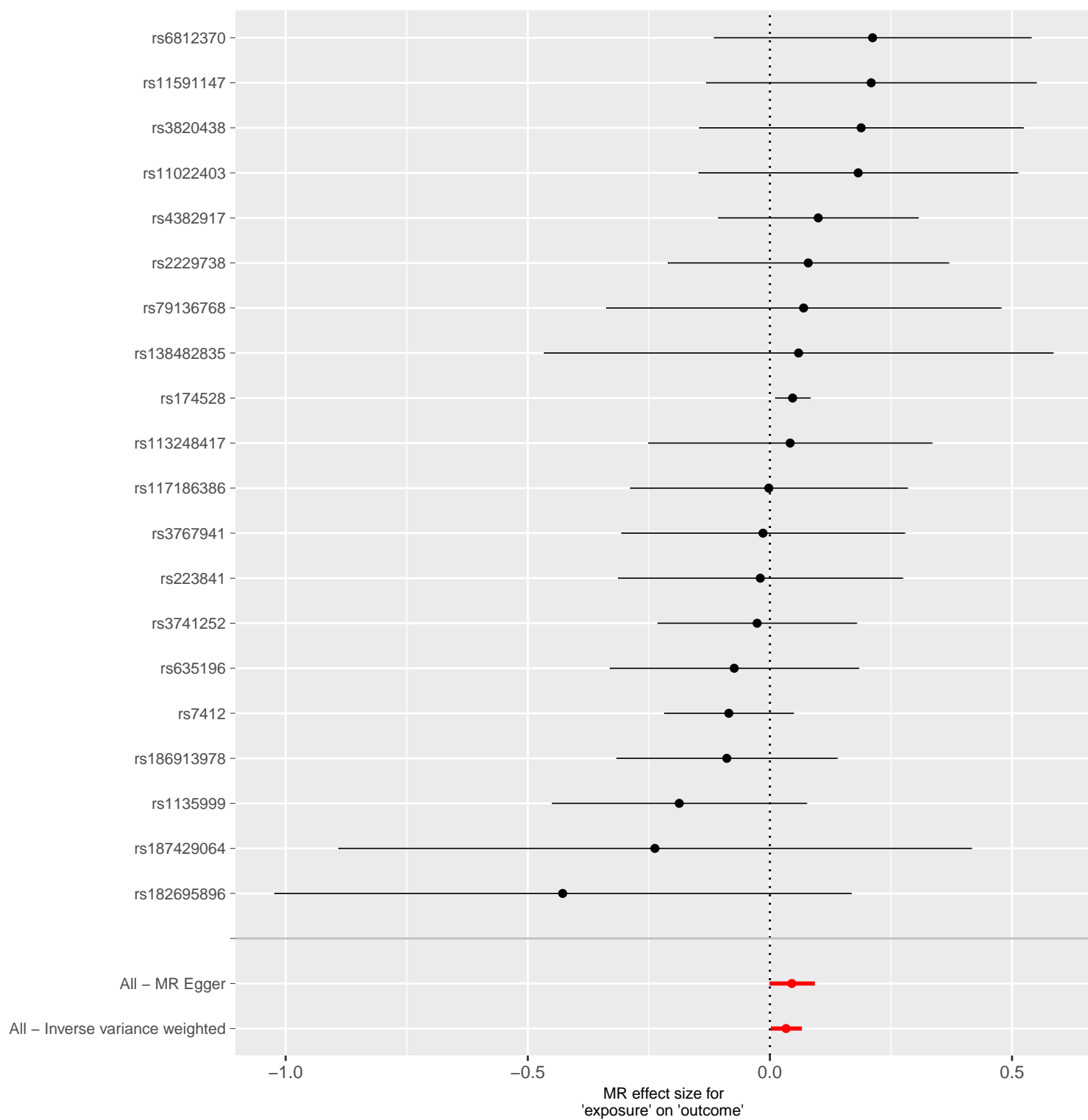

Supplement: Supplementary file 4 — Supplementary Material 4. [file 12944_2024_2103_MOESM4_ESM.zip › sFigure3∩╝êlipidomes-ER-BC∩╝ë/GCST90277250/forest.pdf]

# MR Method

- Inverse variance weighted
- MR Egger

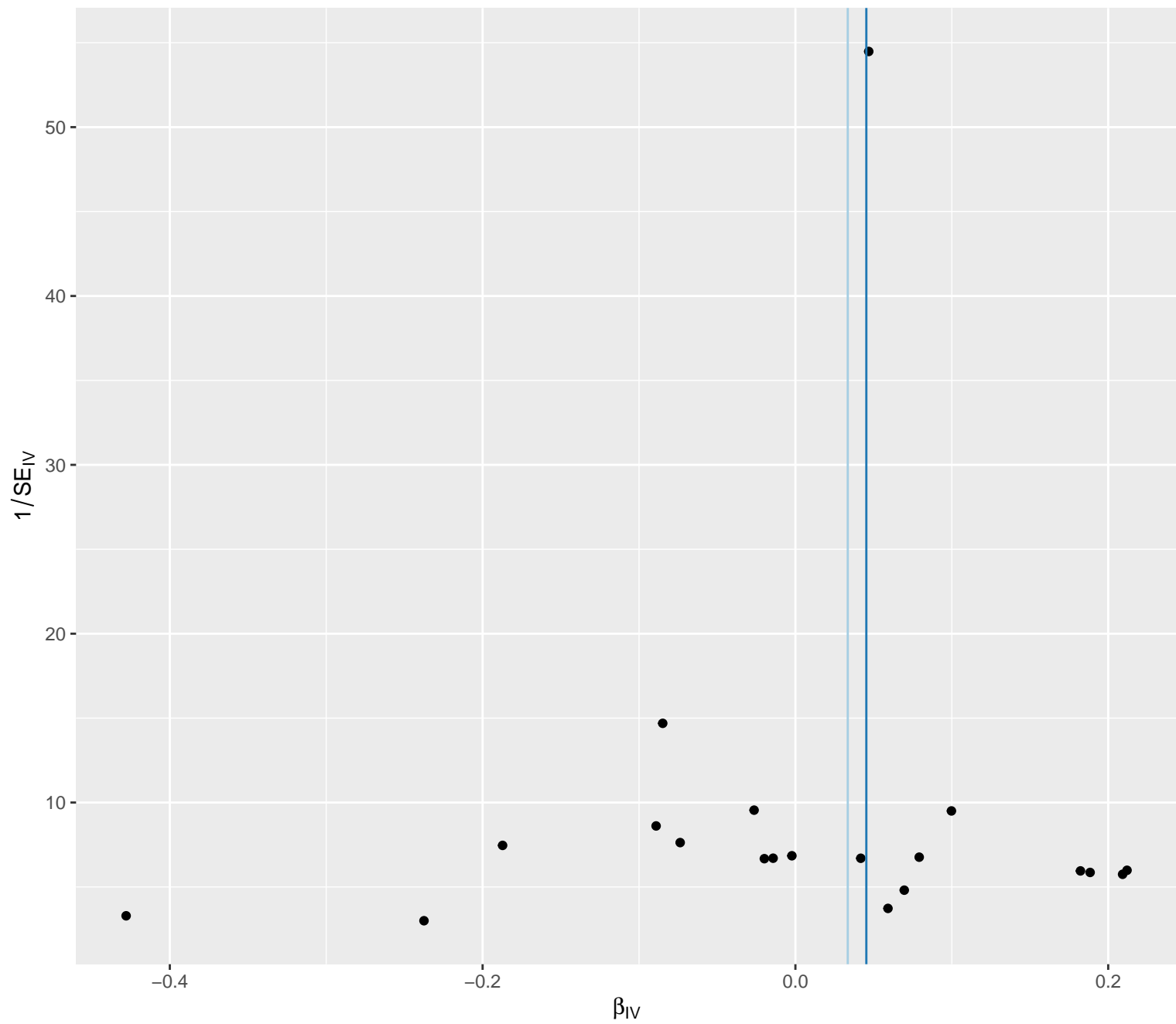

Supplement: Supplementary file 4 — Supplementary Material 4. [file 12944_2024_2103_MOESM4_ESM.zip › sFigure3∩╝êlipidomes-ER-BC∩╝ë/GCST90277250/funnelplot.pdf]

# MR Test

- Inverse variance weighted
- MR Egger
- Simple mode
- Weighted median
- Weighted mode

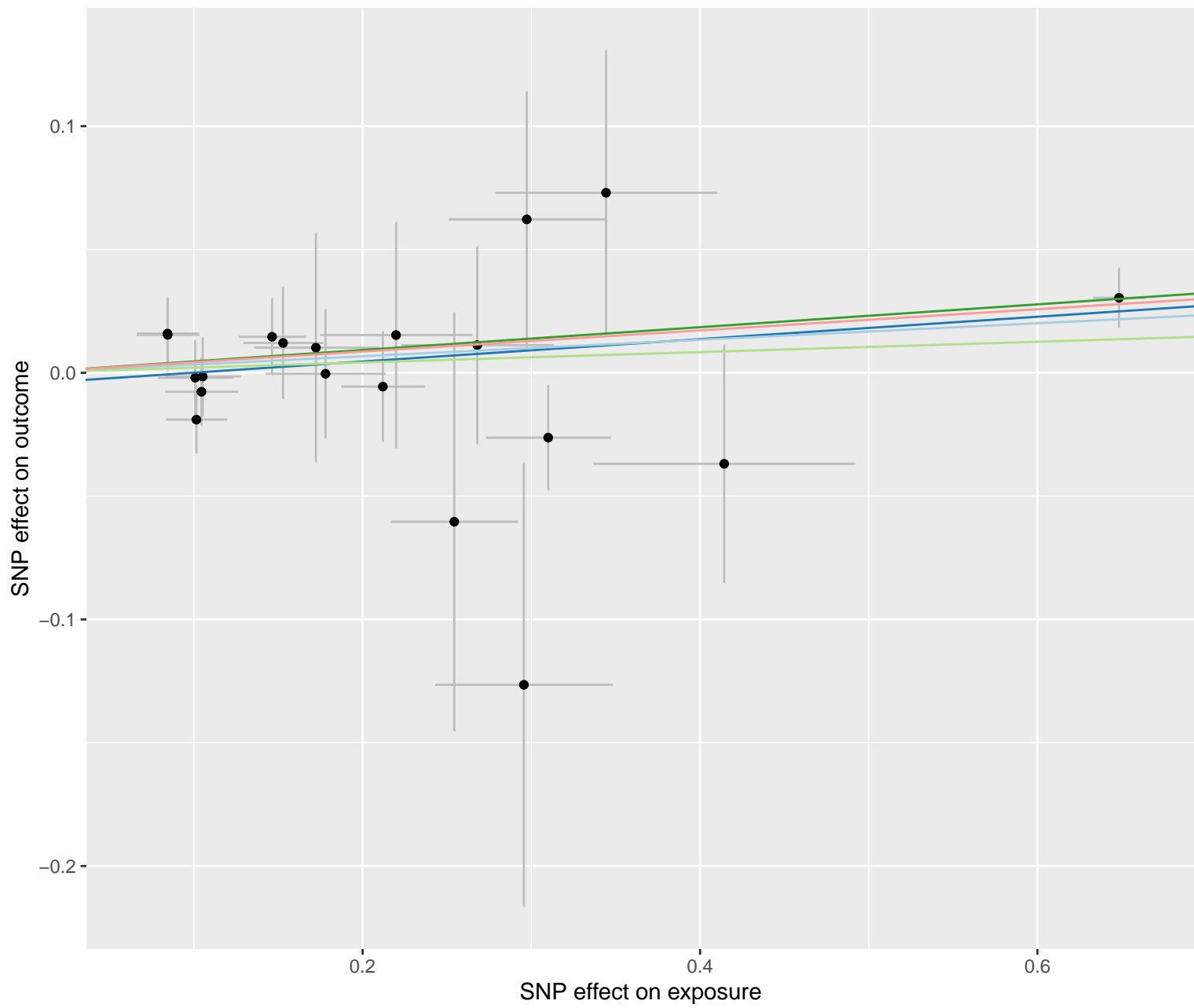

Supplement: Supplementary file 4 — Supplementary Material 4. [file 12944_2024_2103_MOESM4_ESM.zip › sFigure3∩╝êlipidomes-ER-BC∩╝ë/GCST90277250/scatter.pdf]

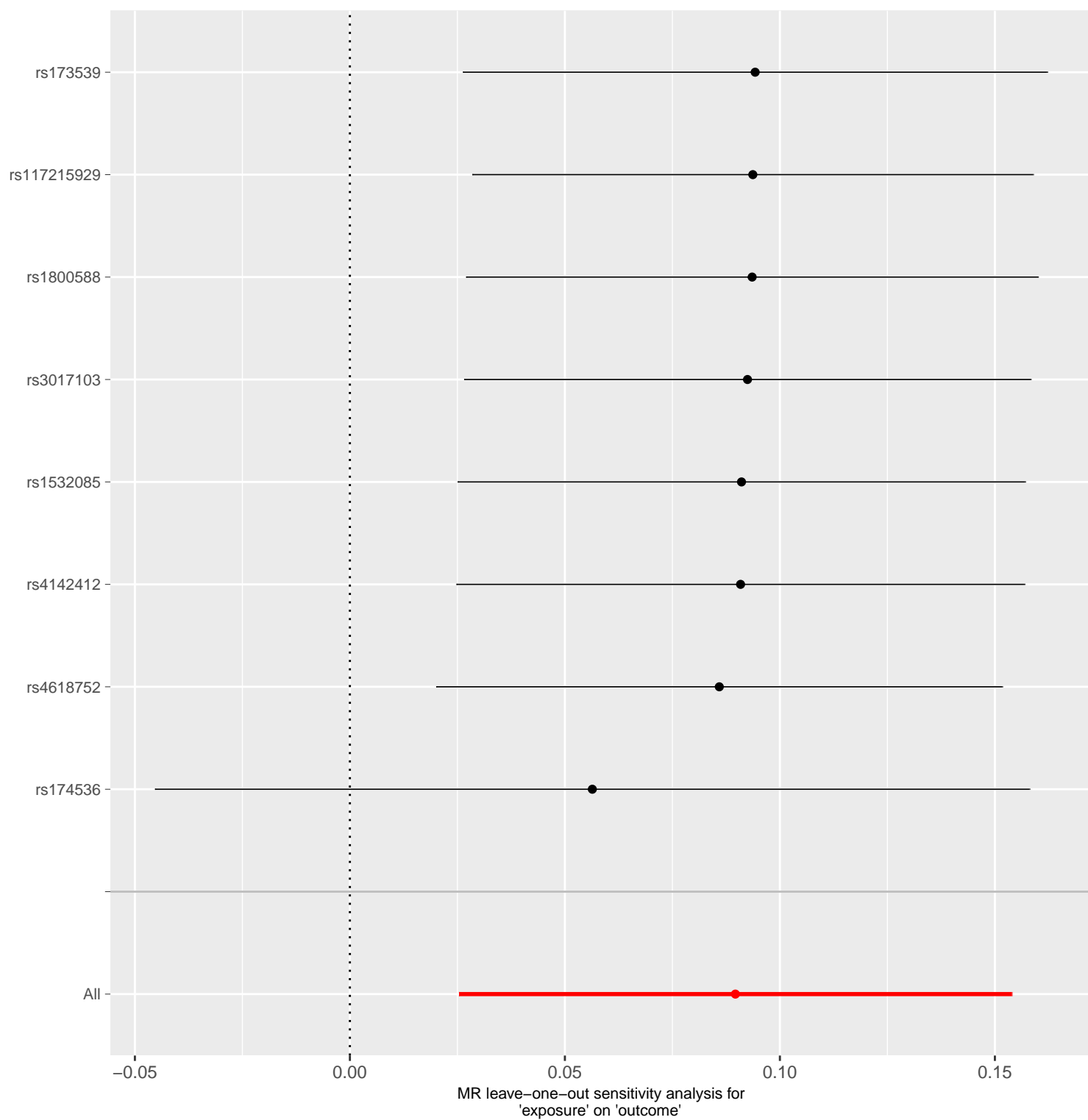

Supplement: Supplementary file 4 — Supplementary Material 4. [file 12944_2024_2103_MOESM4_ESM.zip › sFigure3∩╝êlipidomes-ER-BC∩╝ë/GCST90277340/sensitivity-analysis.pdf]

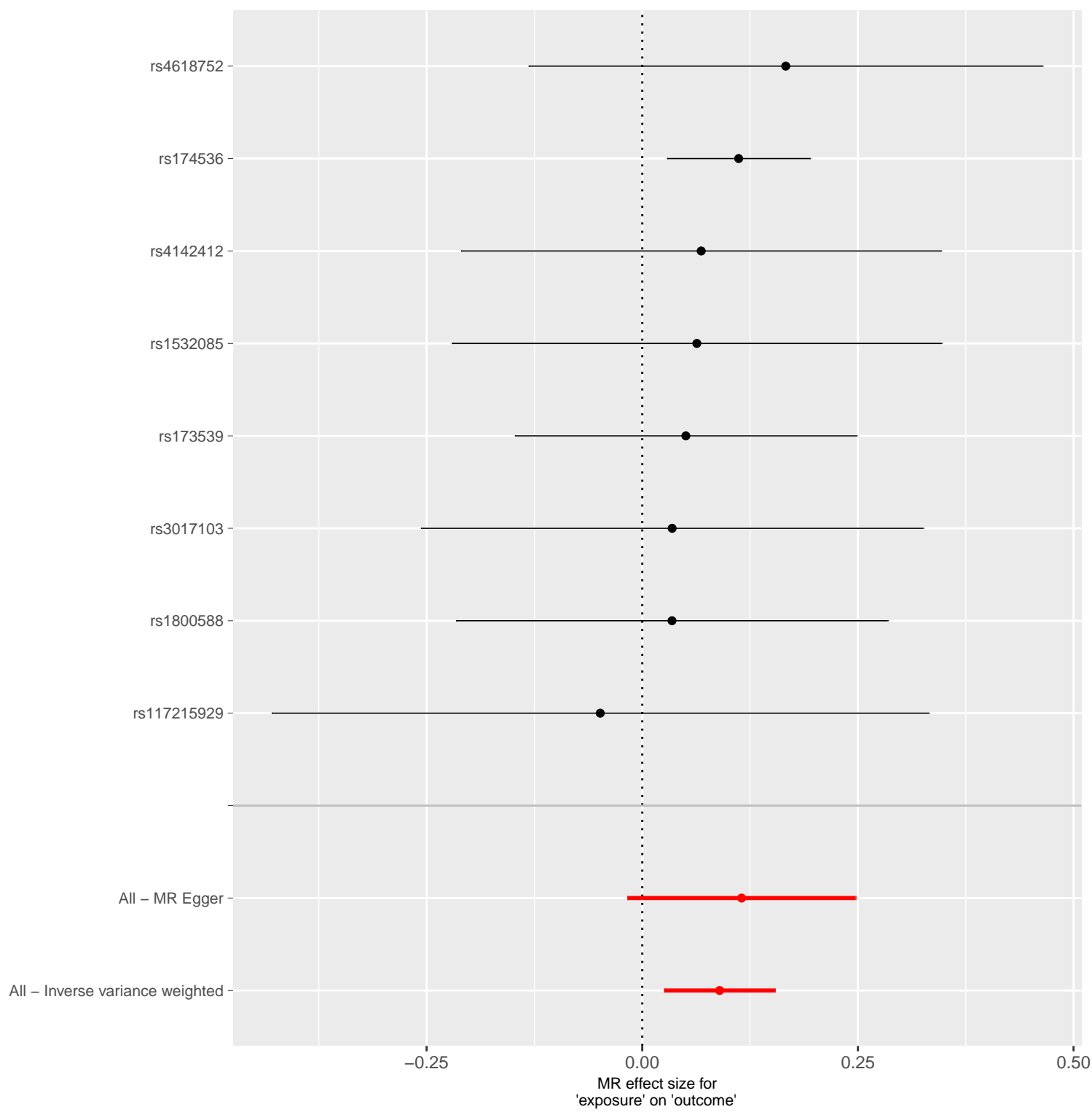

Supplement: Supplementary file 4 — Supplementary Material 4. [file 12944_2024_2103_MOESM4_ESM.zip › sFigure3∩╝êlipidomes-ER-BC∩╝ë/GCST90277340/forest.pdf]

# MR Method

- Inverse variance weighted
- MR Egger

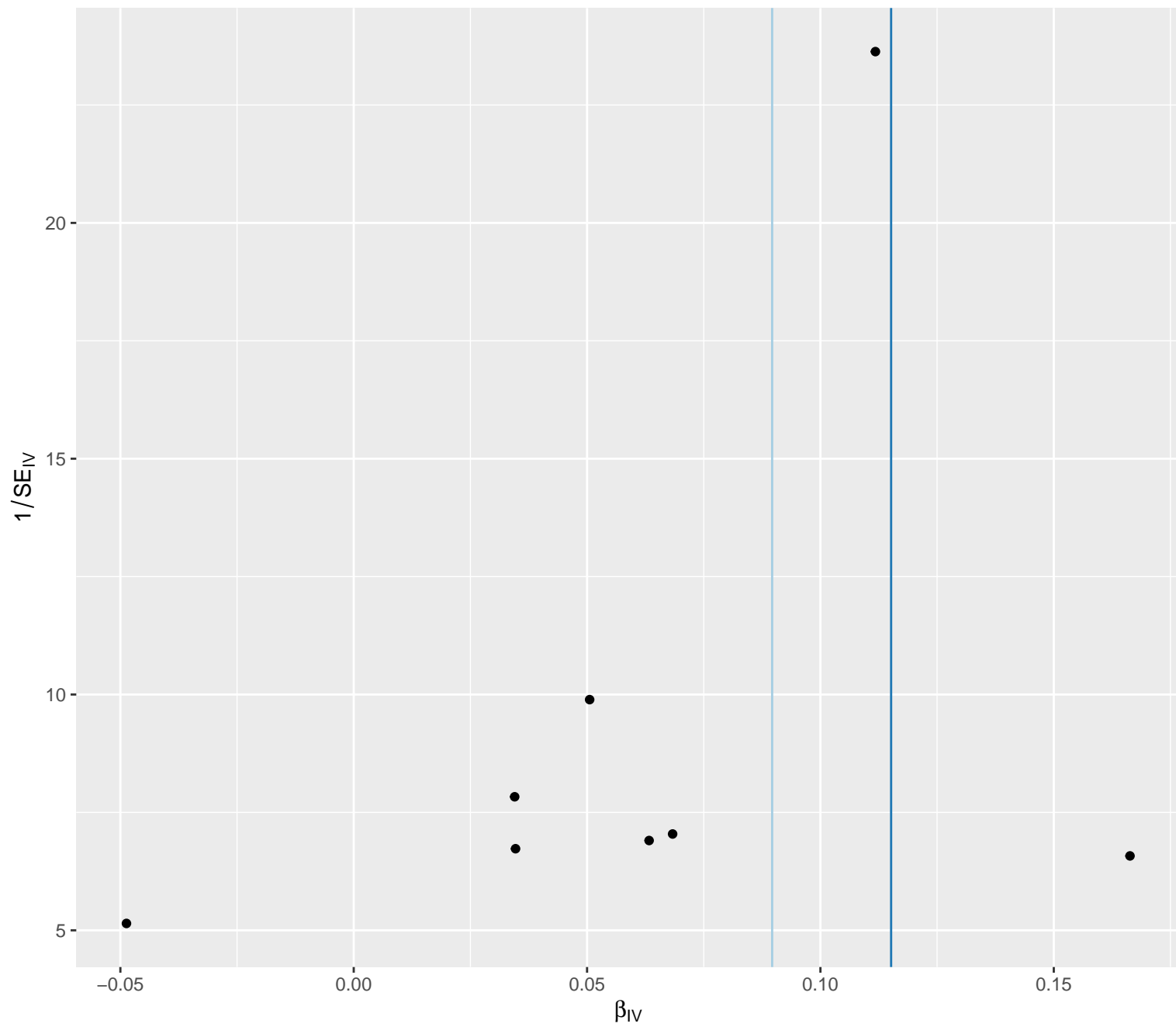

Supplement: Supplementary file 4 — Supplementary Material 4. [file 12944_2024_2103_MOESM4_ESM.zip › sFigure3∩╝êlipidomes-ER-BC∩╝ë/GCST90277340/funnelplot.pdf]

# MR Test

- Inverse variance weighted
- MR Egger
- Simple mode
- Weighted median
- Weighted mode

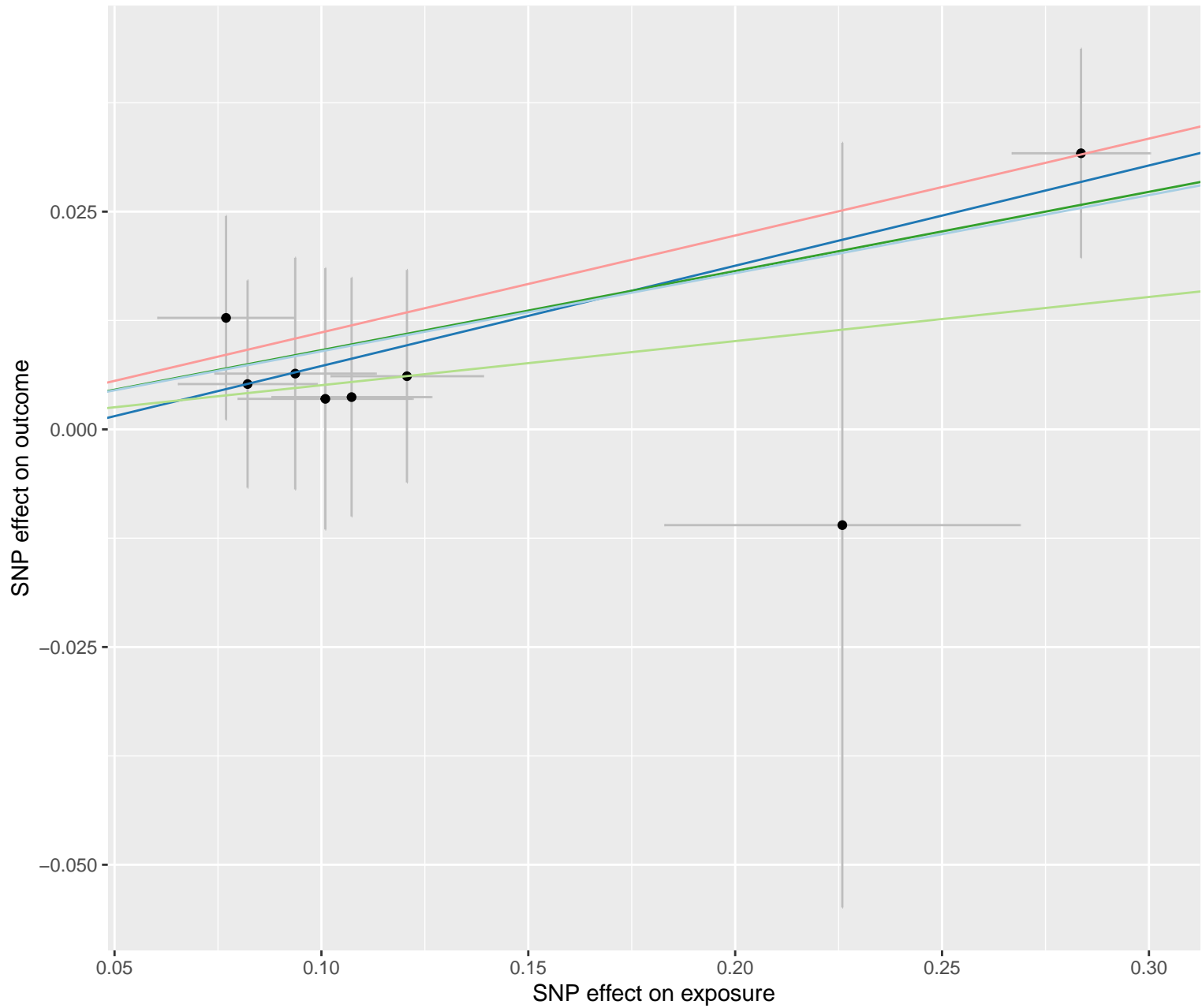

Supplement: Supplementary file 4 — Supplementary Material 4. [file 12944_2024_2103_MOESM4_ESM.zip › sFigure3∩╝êlipidomes-ER-BC∩╝ë/GCST90277340/scatter.pdf]

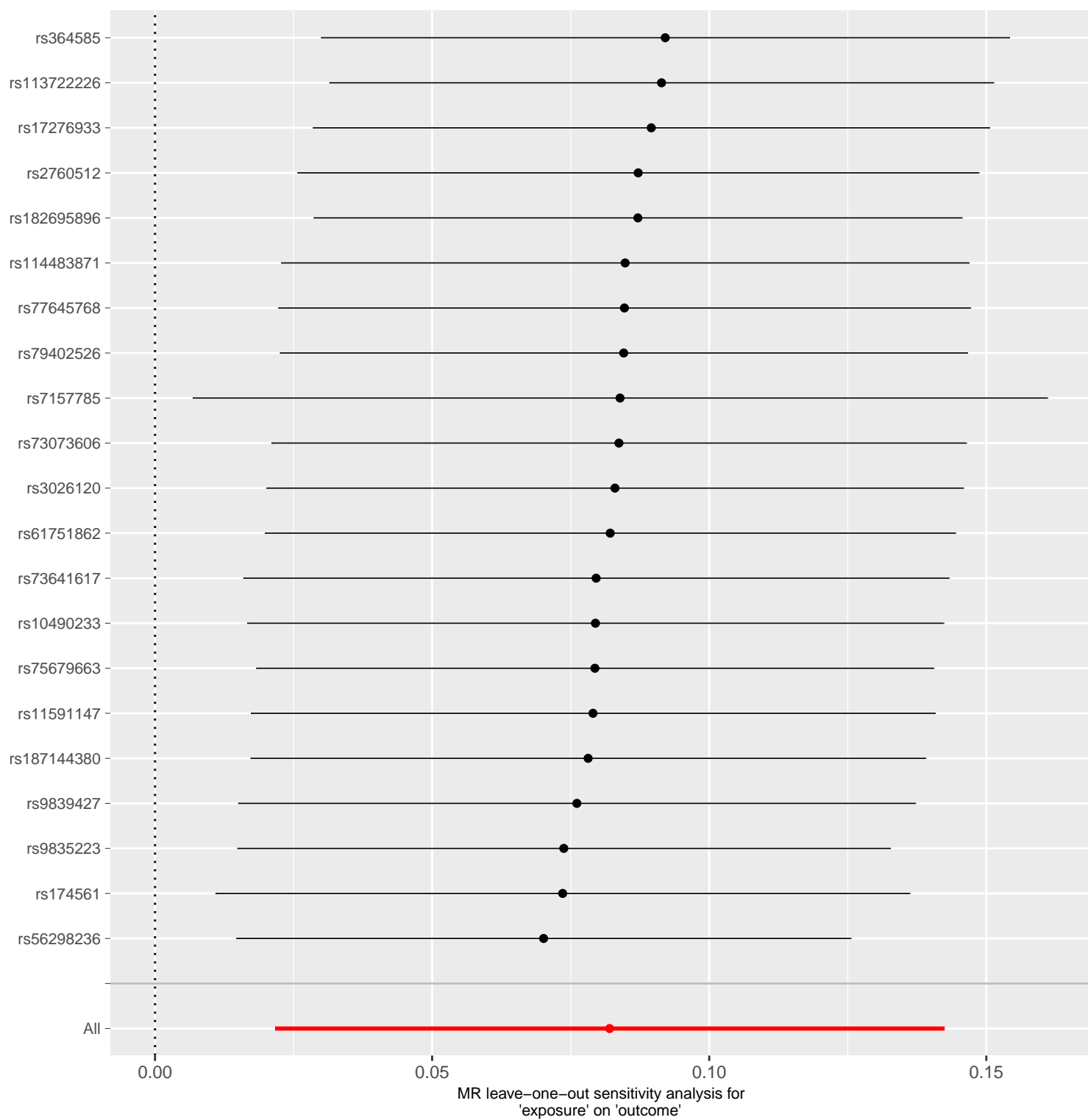

Supplement: Supplementary file 4 — Supplementary Material 4. [file 12944_2024_2103_MOESM4_ESM.zip › sFigure3∩╝êlipidomes-ER-BC∩╝ë/GCST90277377/sensitivity-analysis.pdf]

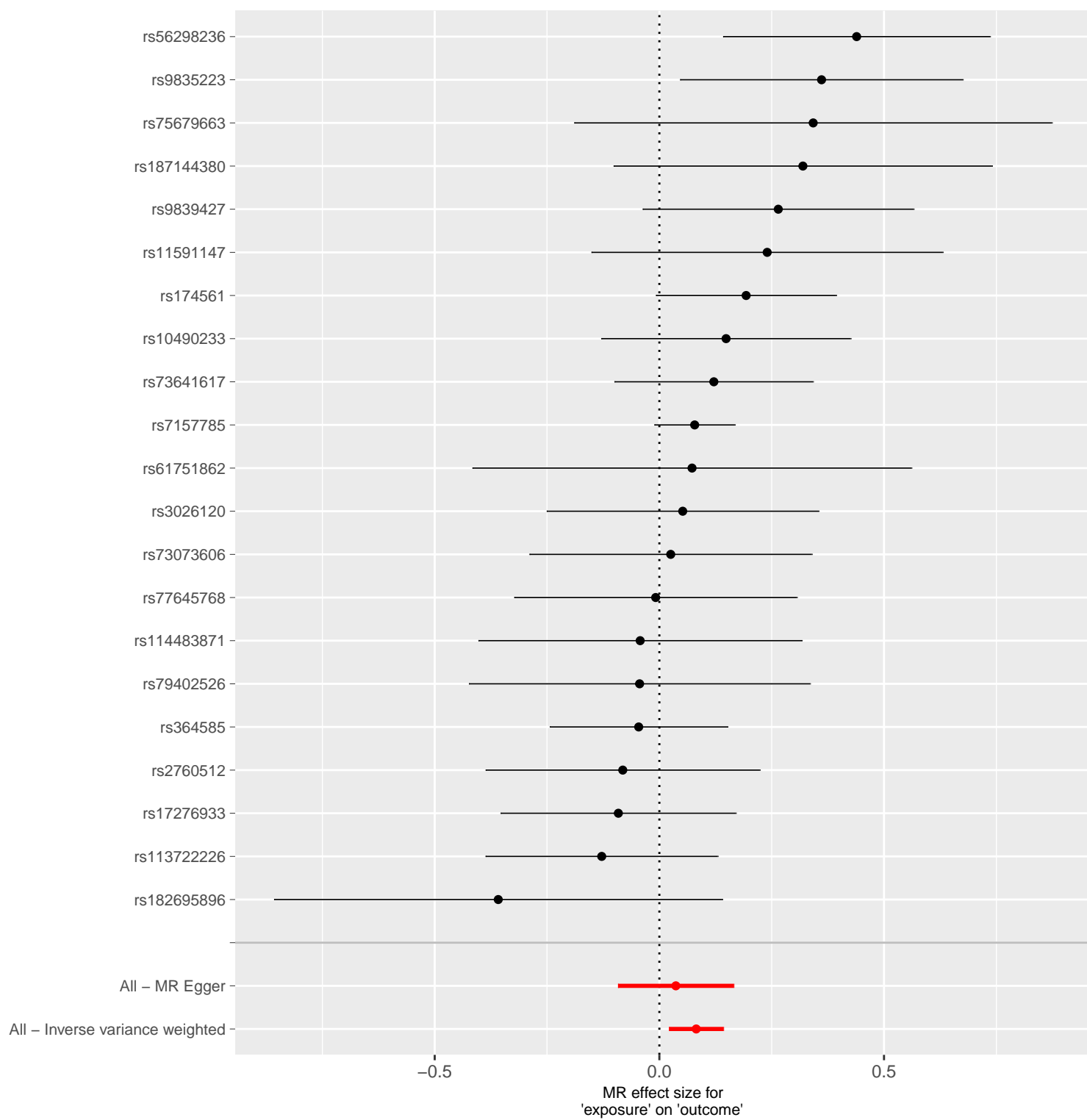

Supplement: Supplementary file 4 — Supplementary Material 4. [file 12944_2024_2103_MOESM4_ESM.zip › sFigure3∩╝êlipidomes-ER-BC∩╝ë/GCST90277377/forest.pdf]

# MR Method

- Inverse variance weighted
- MR Egger

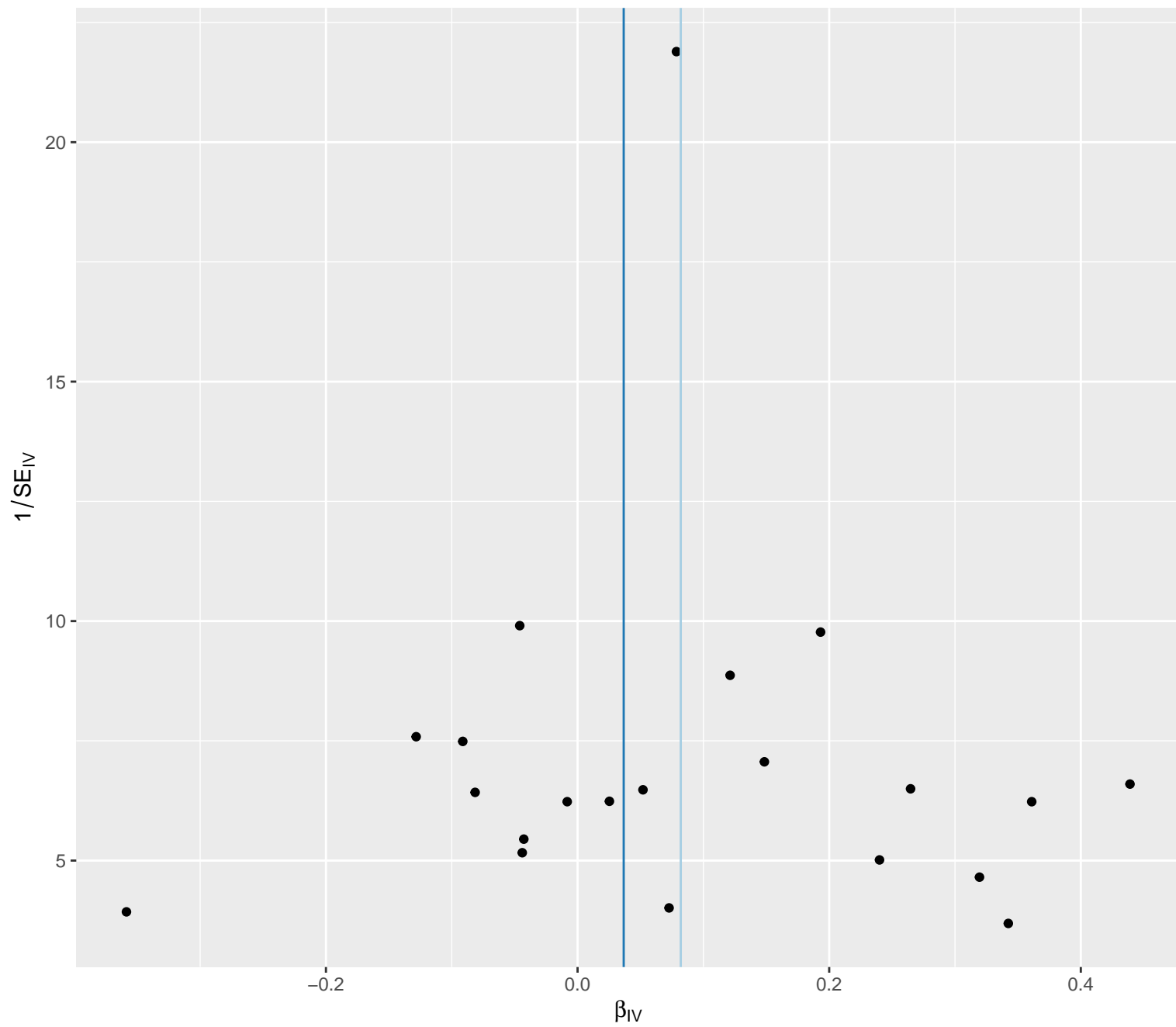

Supplement: Supplementary file 4 — Supplementary Material 4. [file 12944_2024_2103_MOESM4_ESM.zip › sFigure3∩╝êlipidomes-ER-BC∩╝ë/GCST90277377/funnelplot.pdf]

# MR Test

- Inverse variance weighted
- MR Egger
- Simple mode
- Weighted median
- Weighted mode

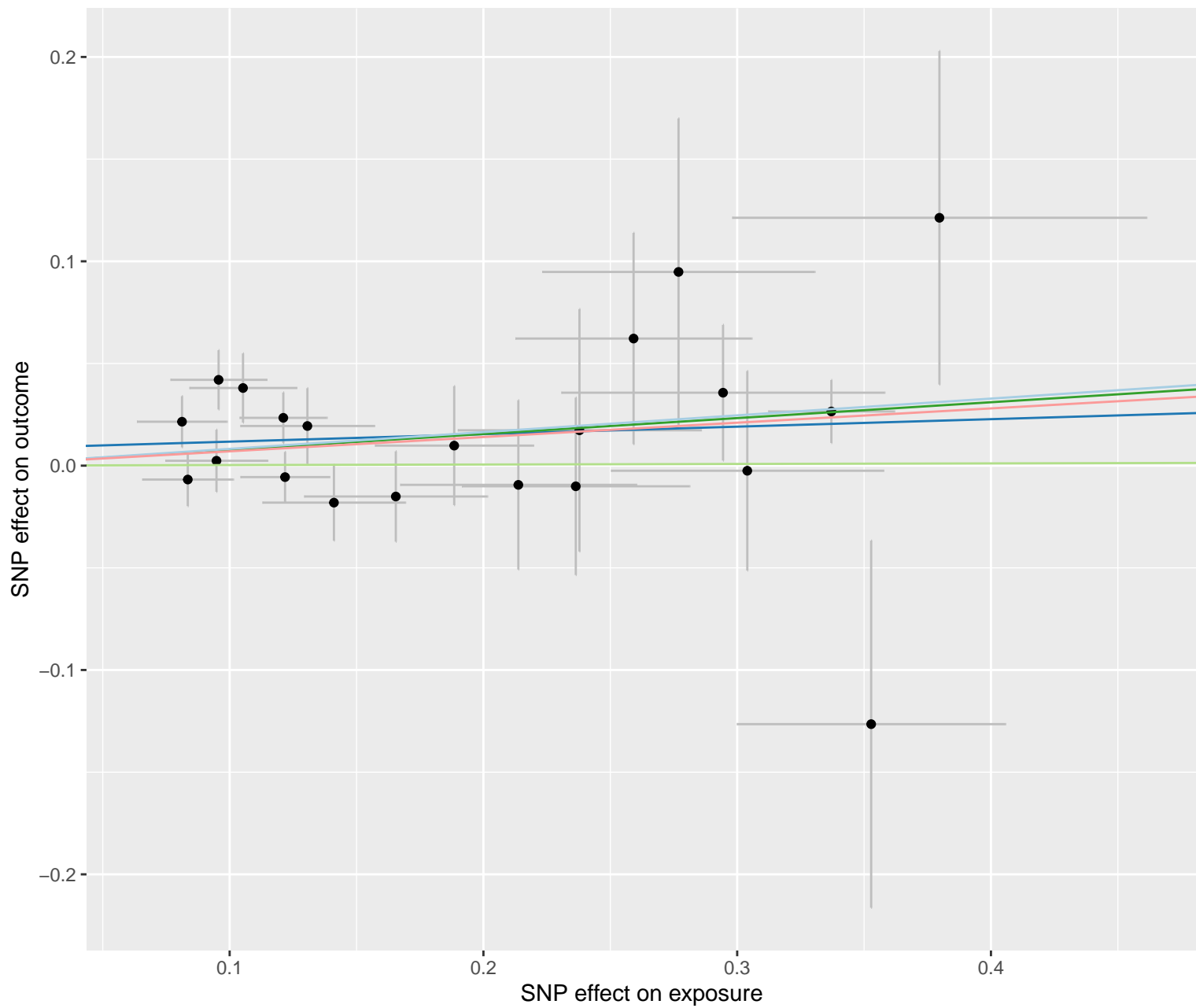

Supplement: Supplementary file 4 — Supplementary Material 4. [file 12944_2024_2103_MOESM4_ESM.zip › sFigure3∩╝êlipidomes-ER-BC∩╝ë/GCST90277377/scatter.pdf]

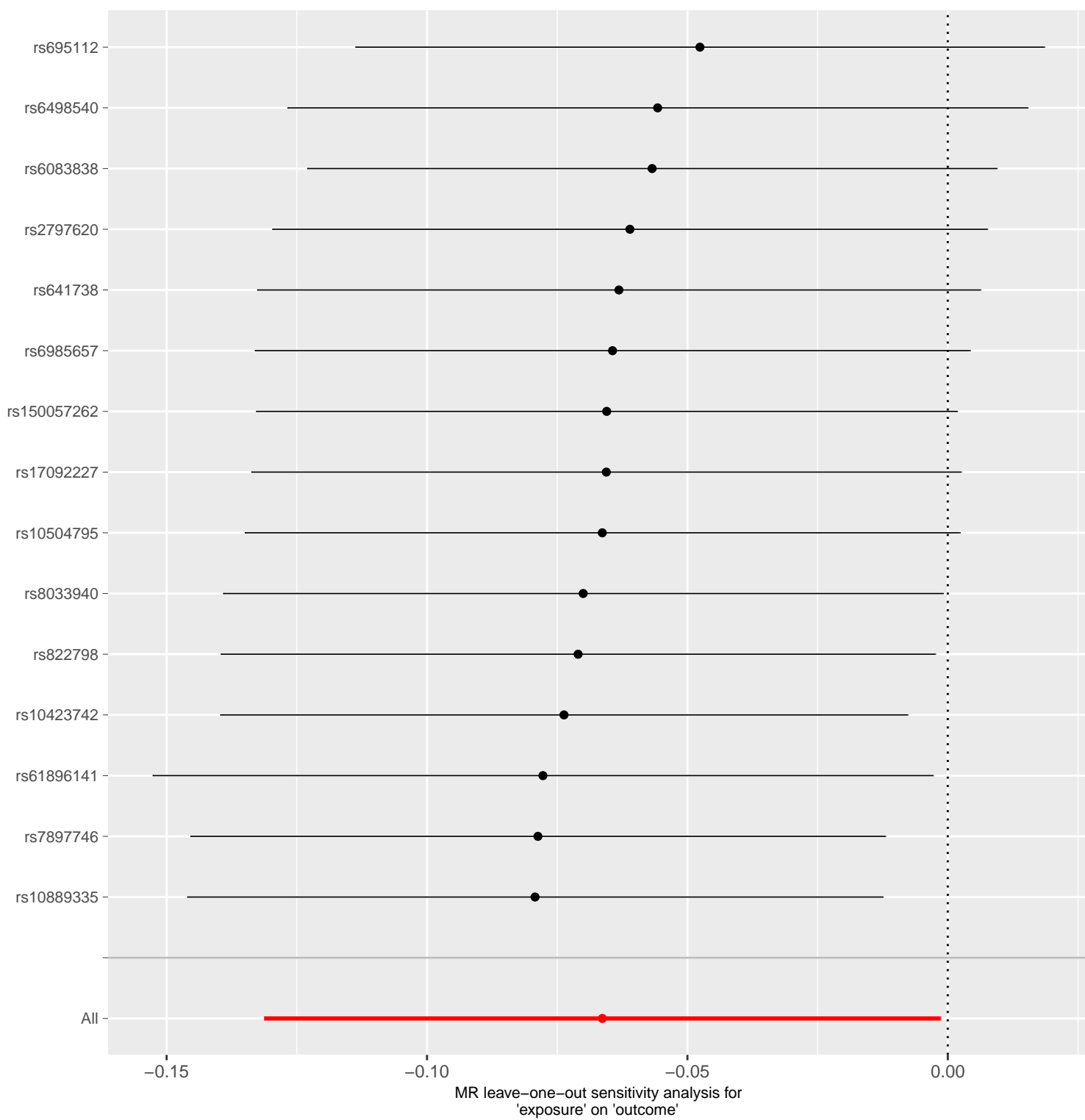

Supplement: Supplementary file 4 — Supplementary Material 4. [file 12944_2024_2103_MOESM4_ESM.zip › sFigure3∩╝êlipidomes-ER-BC∩╝ë/GCST90277312/sensitivity-analysis.pdf]

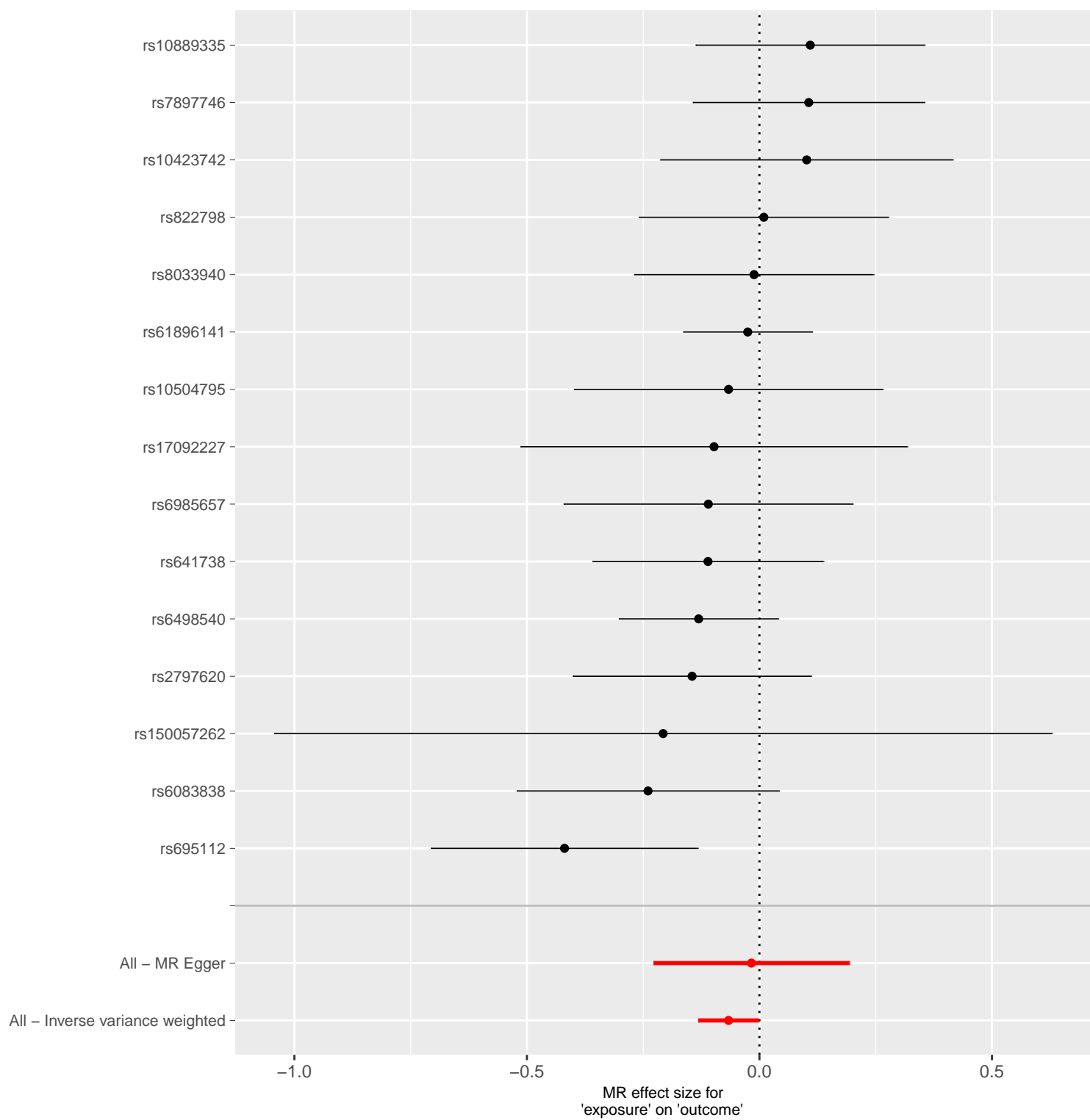

Supplement: Supplementary file 4 — Supplementary Material 4. [file 12944_2024_2103_MOESM4_ESM.zip › sFigure3∩╝êlipidomes-ER-BC∩╝ë/GCST90277312/forest.pdf]

# MR Method

- Inverse variance weighted
- MR Egger

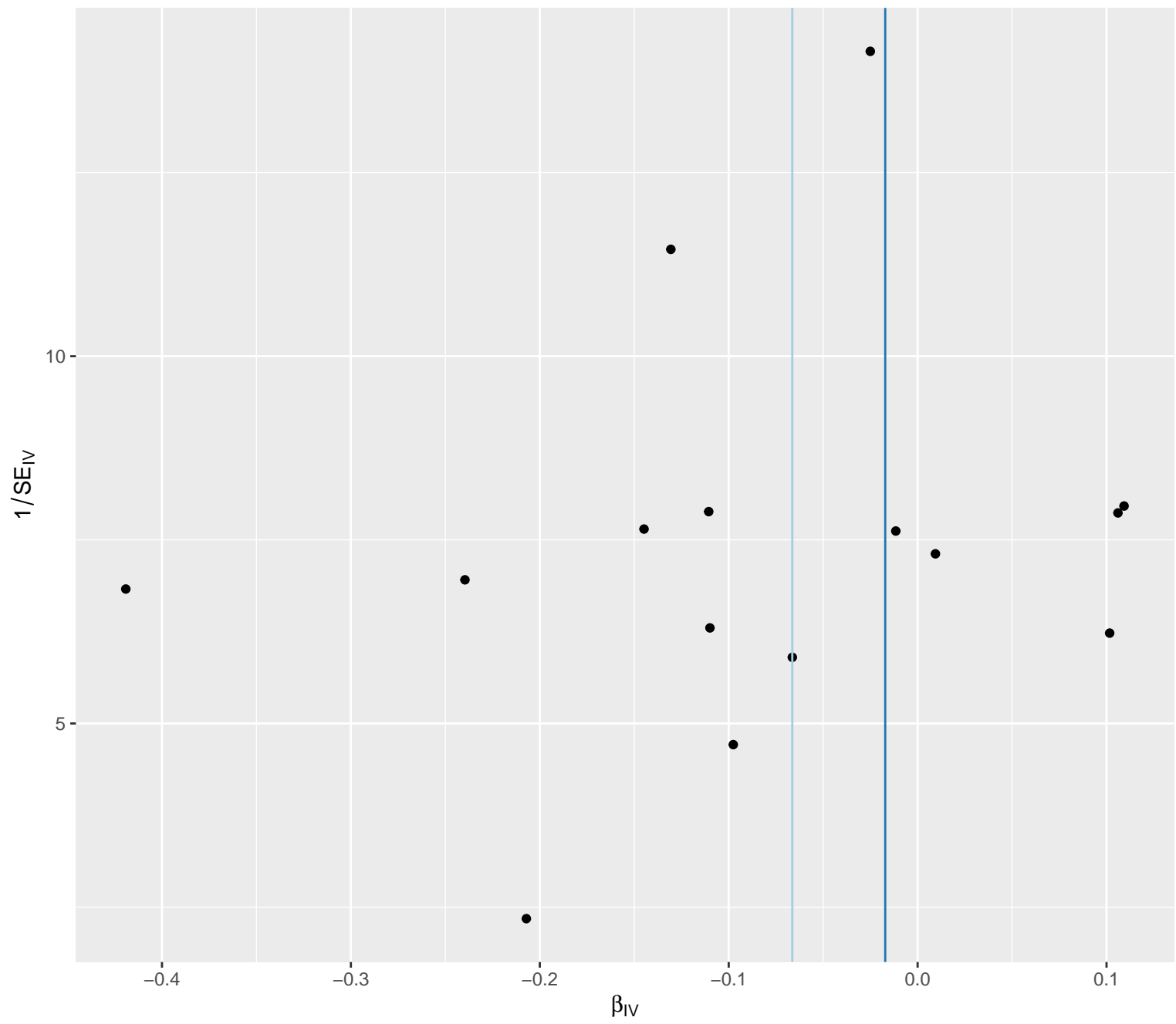

Supplement: Supplementary file 4 — Supplementary Material 4. [file 12944_2024_2103_MOESM4_ESM.zip › sFigure3∩╝êlipidomes-ER-BC∩╝ë/GCST90277312/funnelplot.pdf]

# MR Test

- Inverse variance weighted
- MR Egger
- Simple mode
- Weighted median
- Weighted mode

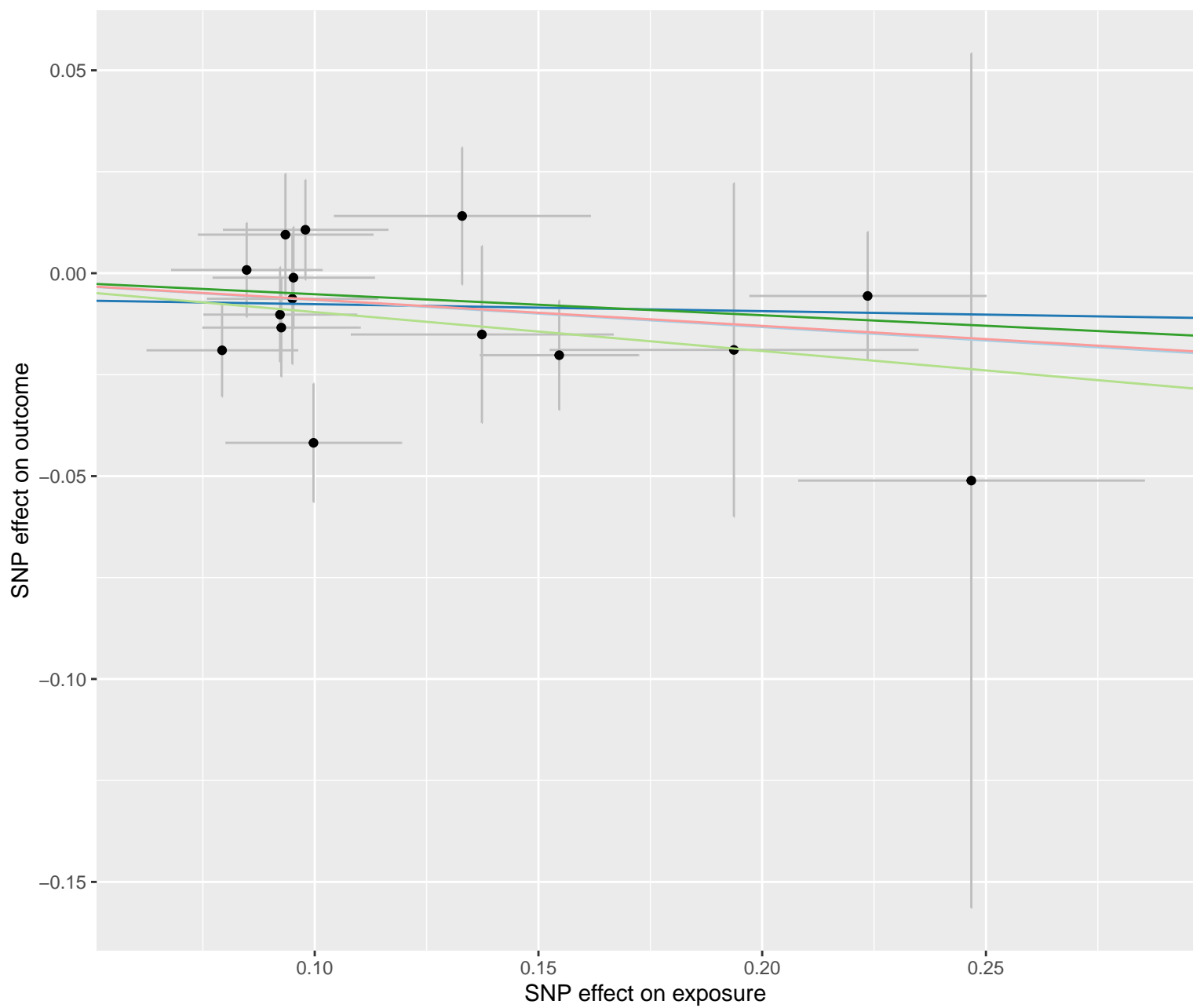

Supplement: Supplementary file 4 — Supplementary Material 4. [file 12944_2024_2103_MOESM4_ESM.zip › sFigure3∩╝êlipidomes-ER-BC∩╝ë/GCST90277312/scatter.pdf]

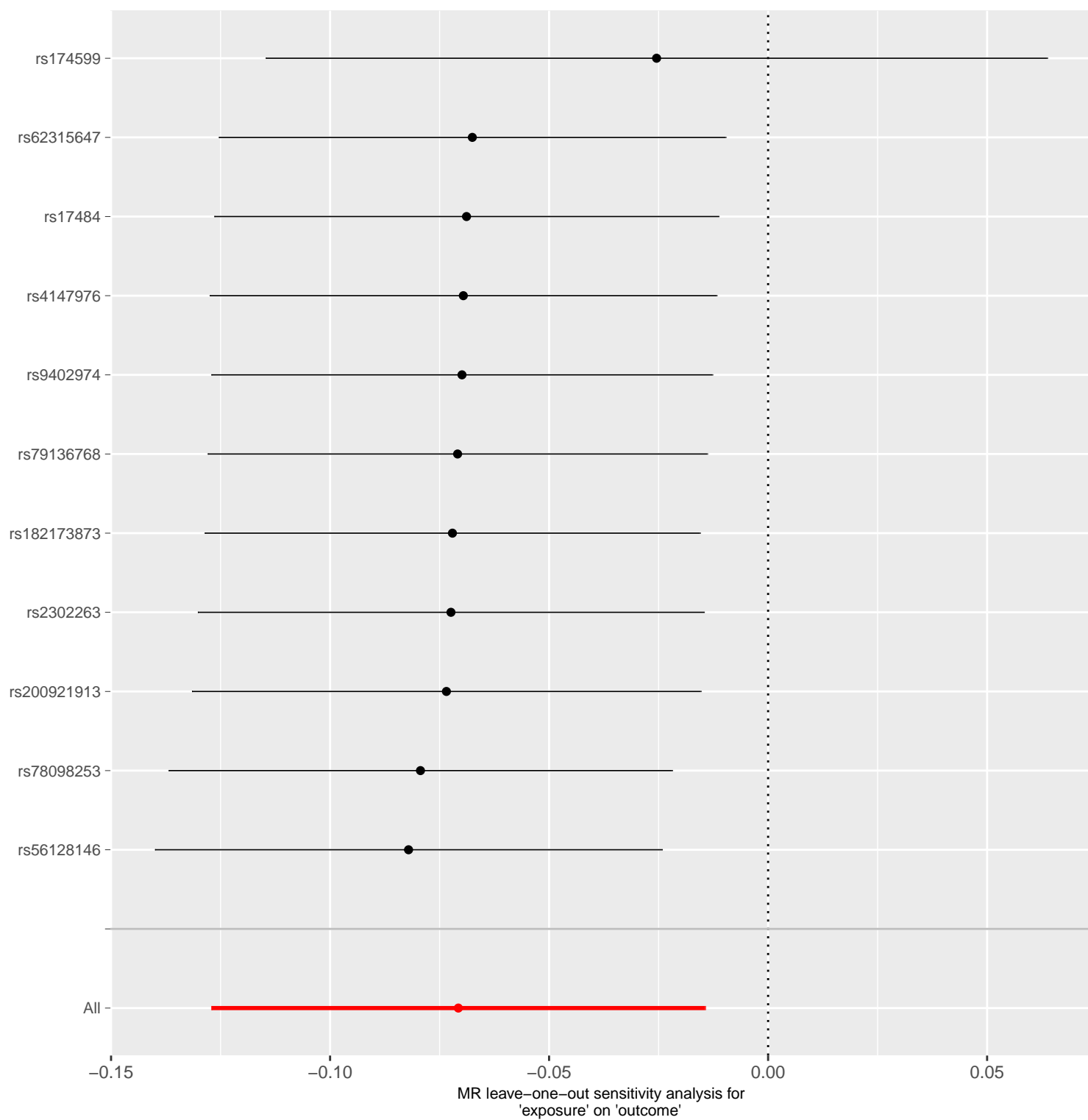

Supplement: Supplementary file 4 — Supplementary Material 4. [file 12944_2024_2103_MOESM4_ESM.zip › sFigure3∩╝êlipidomes-ER-BC∩╝ë/GCST90277315/sensitivity-analysis.pdf]

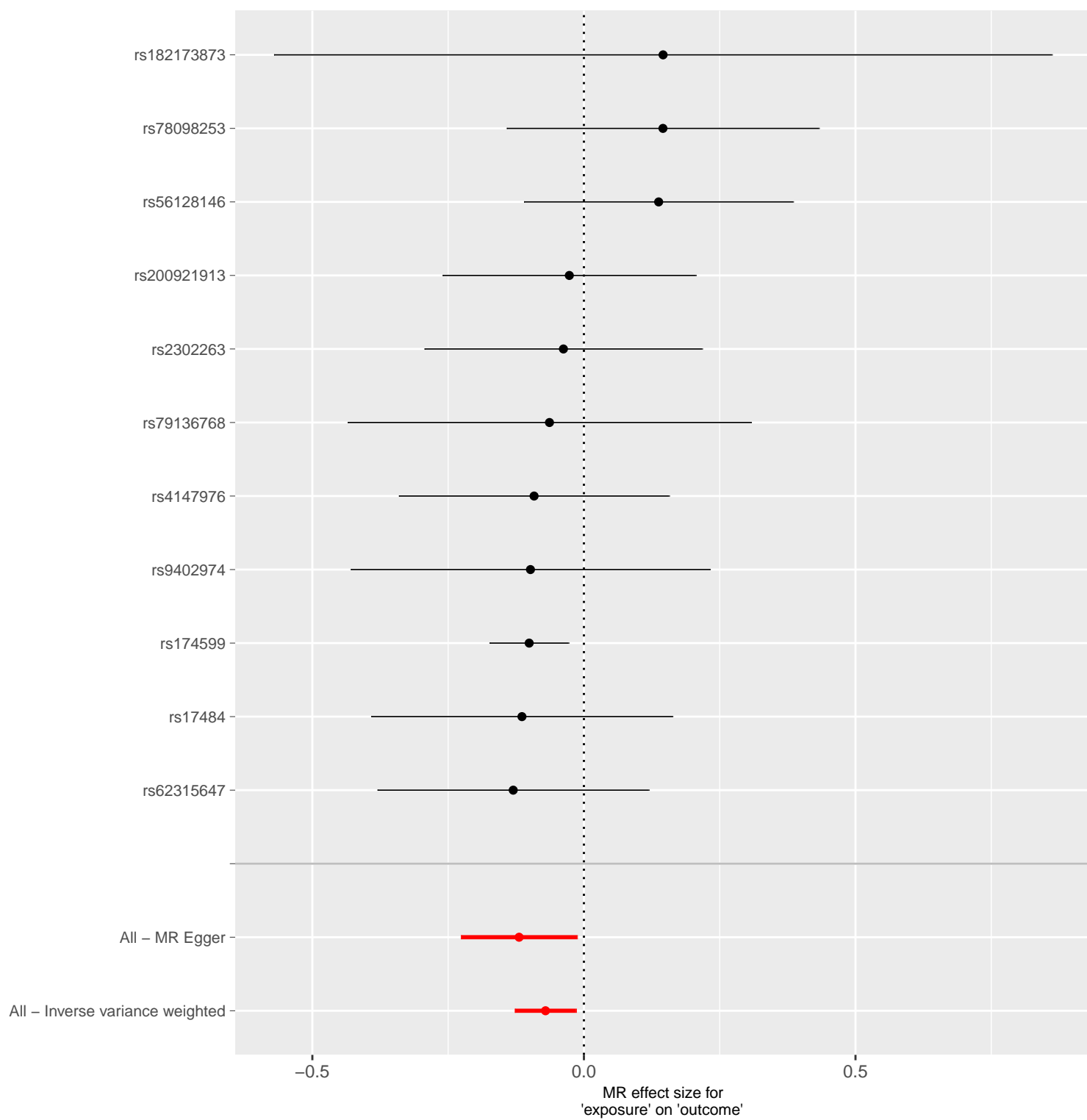

Supplement: Supplementary file 4 — Supplementary Material 4. [file 12944_2024_2103_MOESM4_ESM.zip › sFigure3∩╝êlipidomes-ER-BC∩╝ë/GCST90277315/forest.pdf]

# MR Method

- Inverse variance weighted
- MR Egger

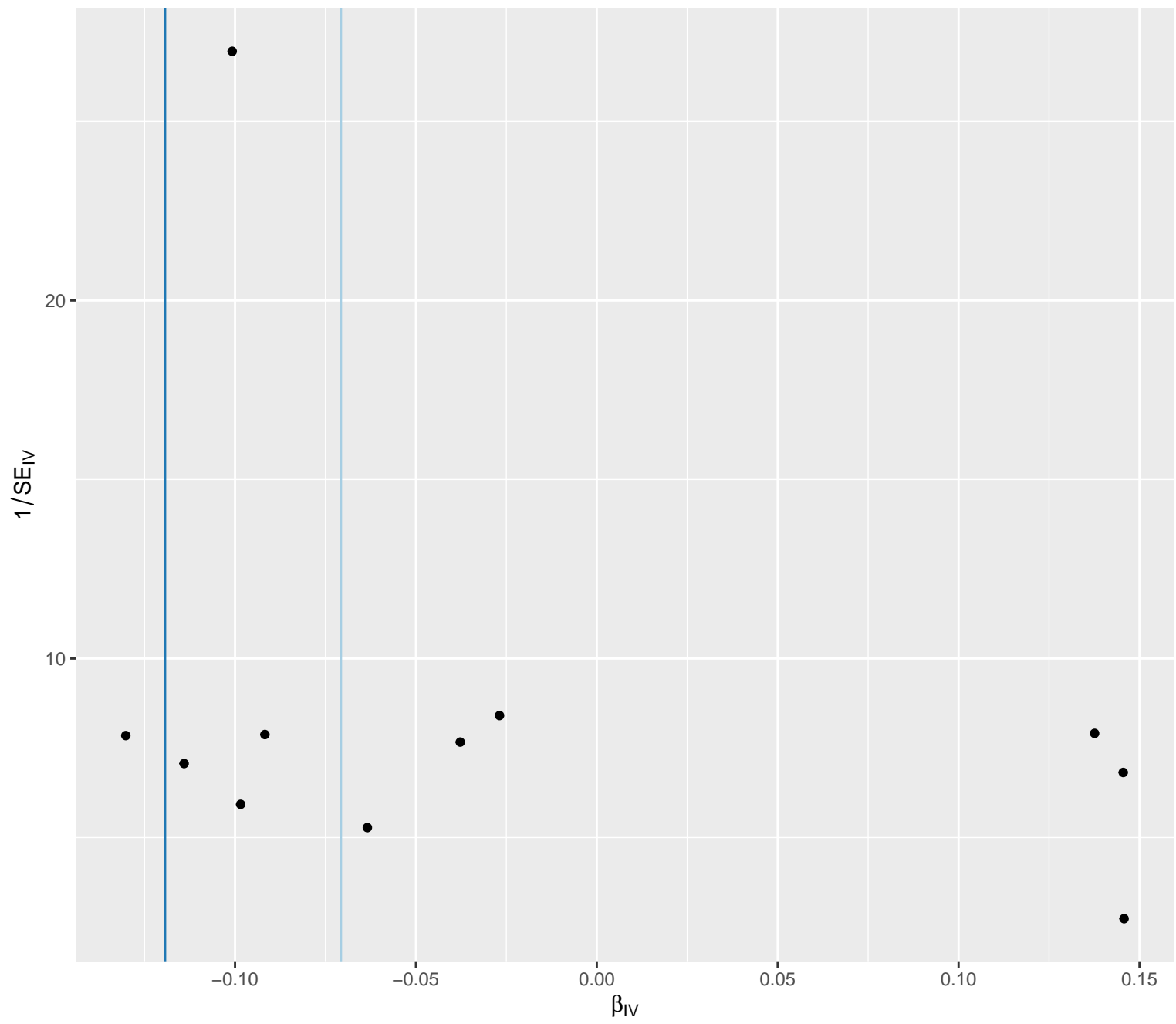

Supplement: Supplementary file 4 — Supplementary Material 4. [file 12944_2024_2103_MOESM4_ESM.zip › sFigure3∩╝êlipidomes-ER-BC∩╝ë/GCST90277315/funnelplot.pdf]

# MR Test

- Inverse variance weighted
- MR Egger
- Simple mode
- Weighted median
- Weighted mode

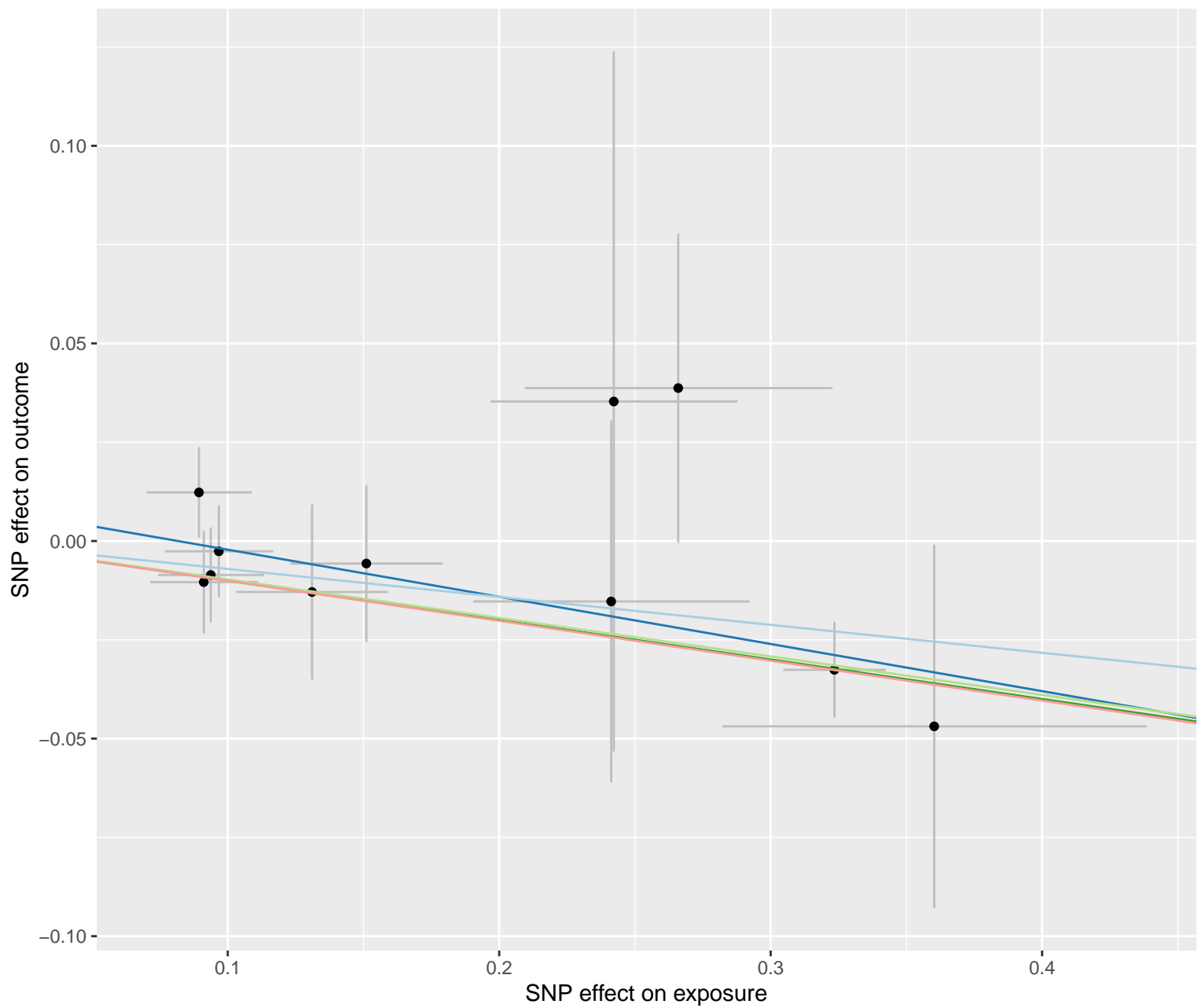

Supplement: Supplementary file 4 — Supplementary Material 4. [file 12944_2024_2103_MOESM4_ESM.zip › sFigure3∩╝êlipidomes-ER-BC∩╝ë/GCST90277315/scatter.pdf]

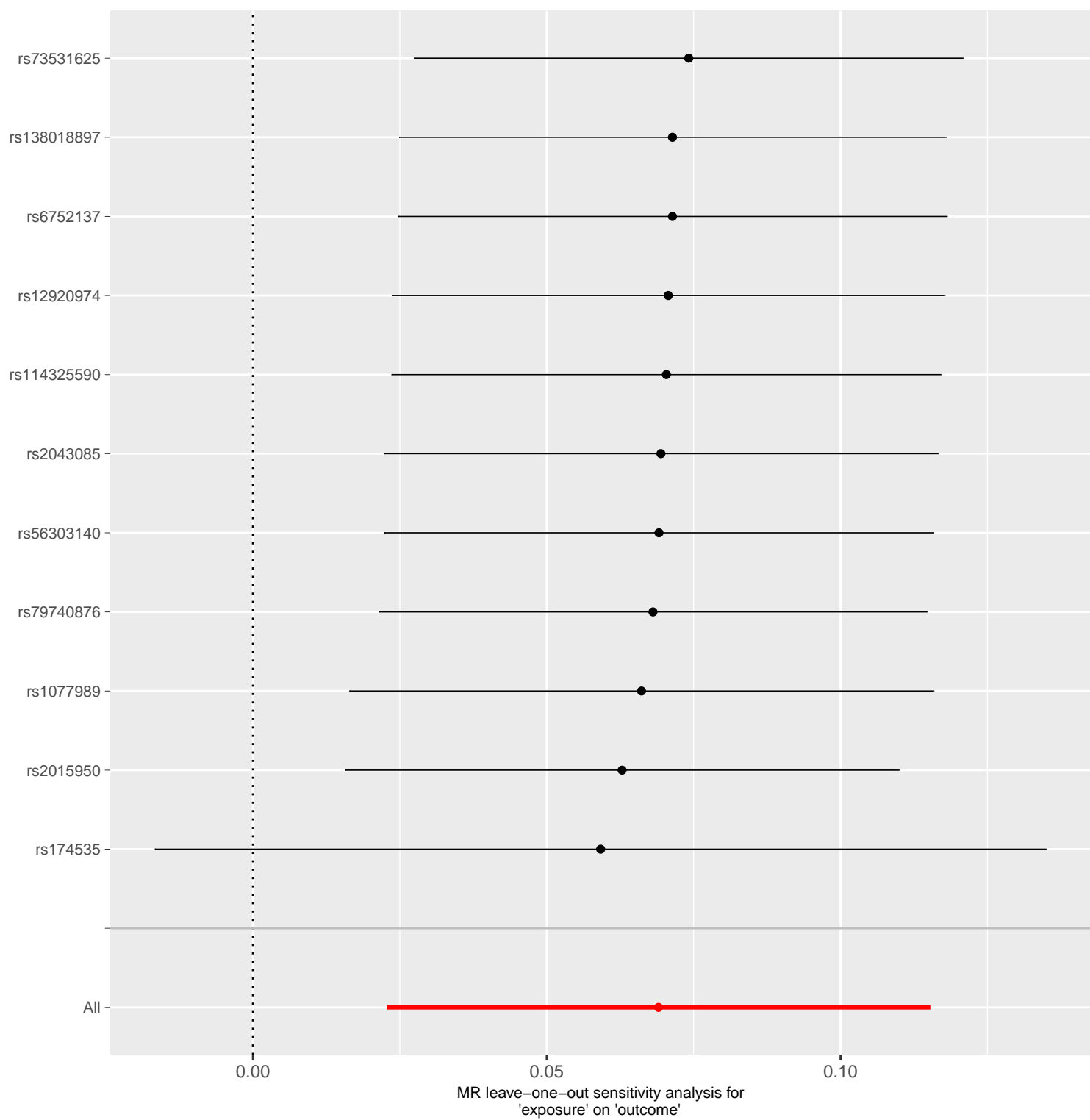

Supplement: Supplementary file 4 — Supplementary Material 4. [file 12944_2024_2103_MOESM4_ESM.zip › sFigure3∩╝êlipidomes-ER-BC∩╝ë/GCST90277330/sensitivity-analysis.pdf]

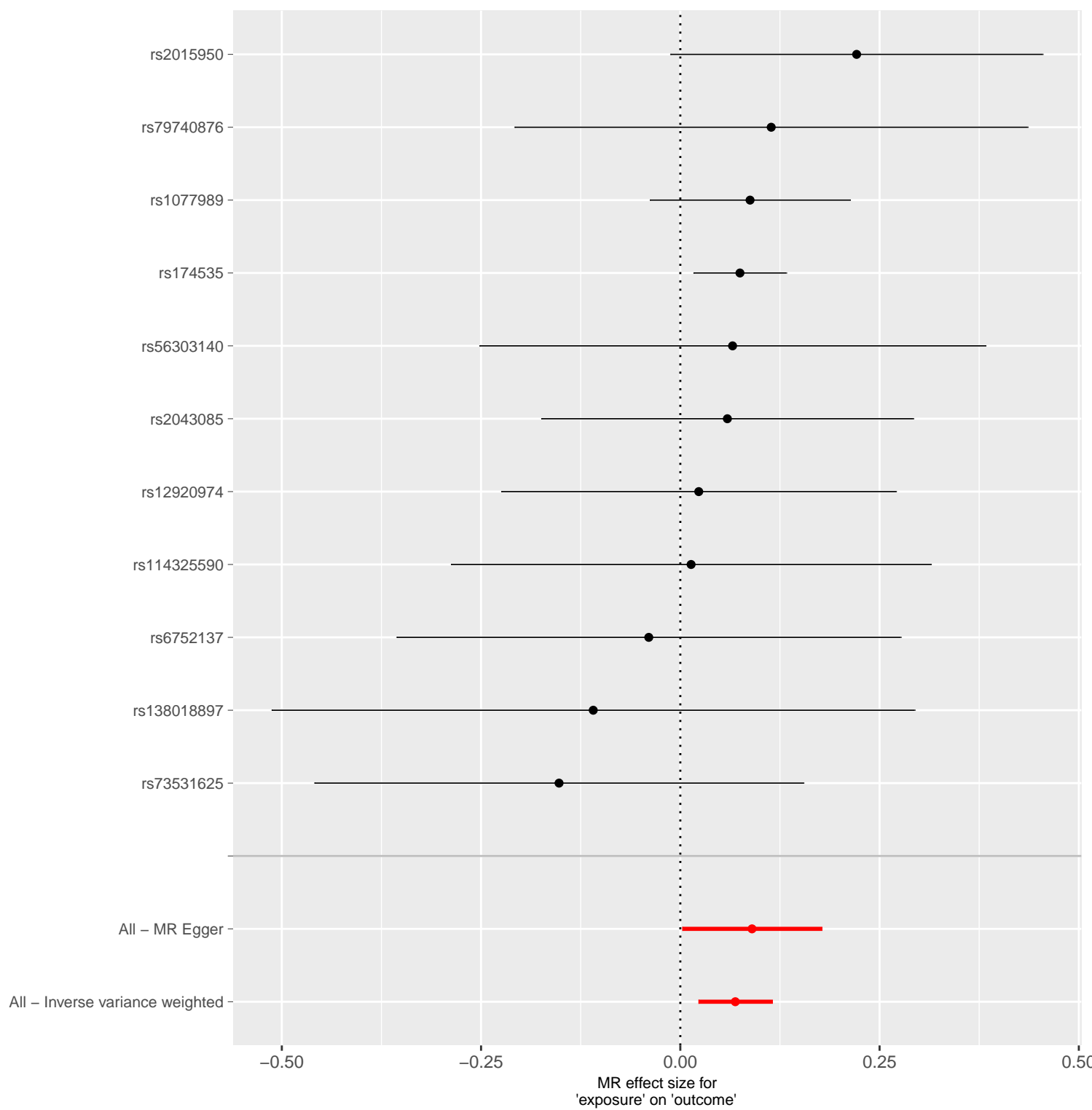

Supplement: Supplementary file 4 — Supplementary Material 4. [file 12944_2024_2103_MOESM4_ESM.zip › sFigure3∩╝êlipidomes-ER-BC∩╝ë/GCST90277330/forest.pdf]

# MR Method

- Inverse variance weighted
- MR Egger

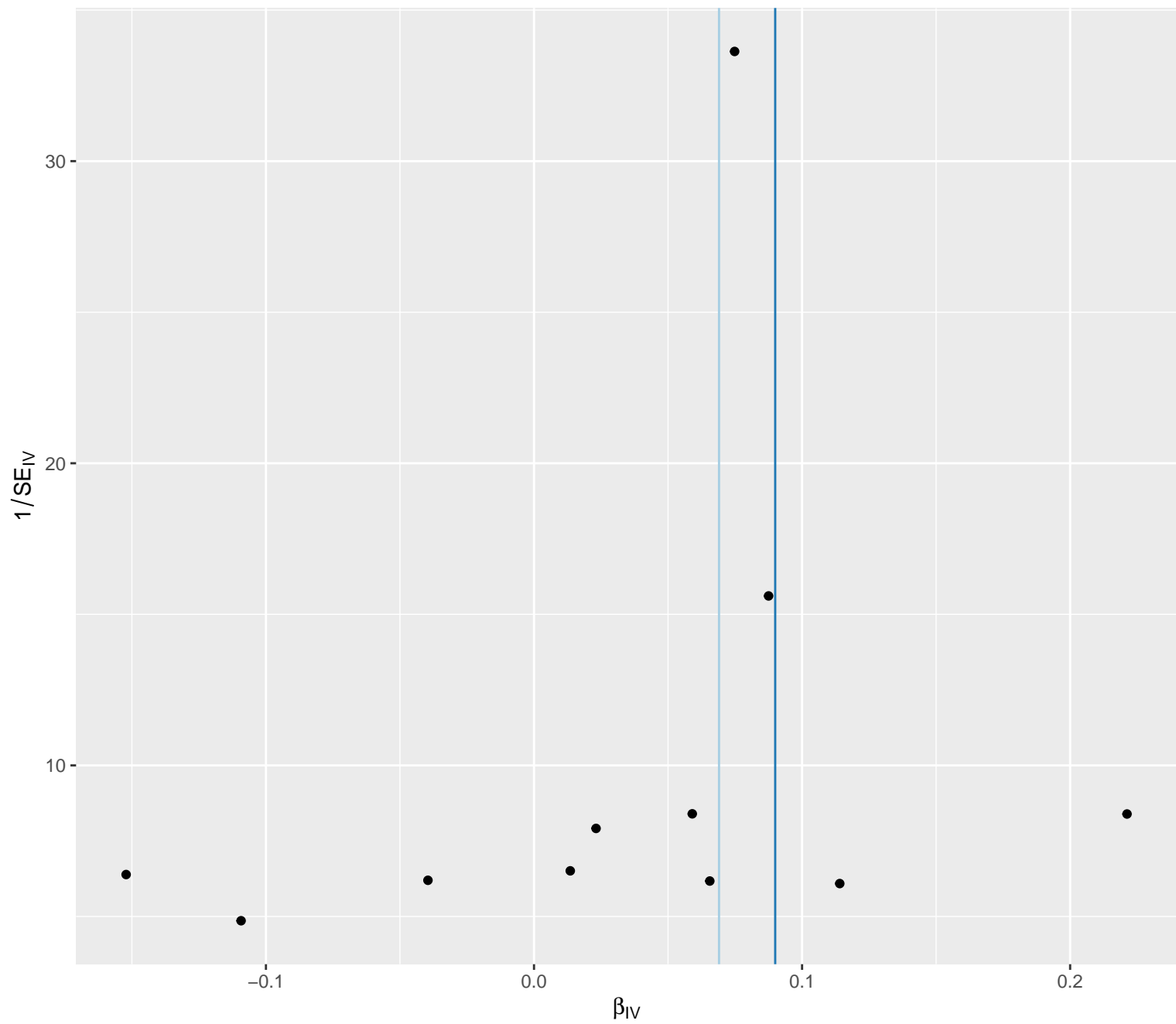

Supplement: Supplementary file 4 — Supplementary Material 4. [file 12944_2024_2103_MOESM4_ESM.zip › sFigure3∩╝êlipidomes-ER-BC∩╝ë/GCST90277330/funnelplot.pdf]

# MR Test

- Inverse variance weighted
- MR Egger
- Simple mode
- Weighted median
- Weighted mode

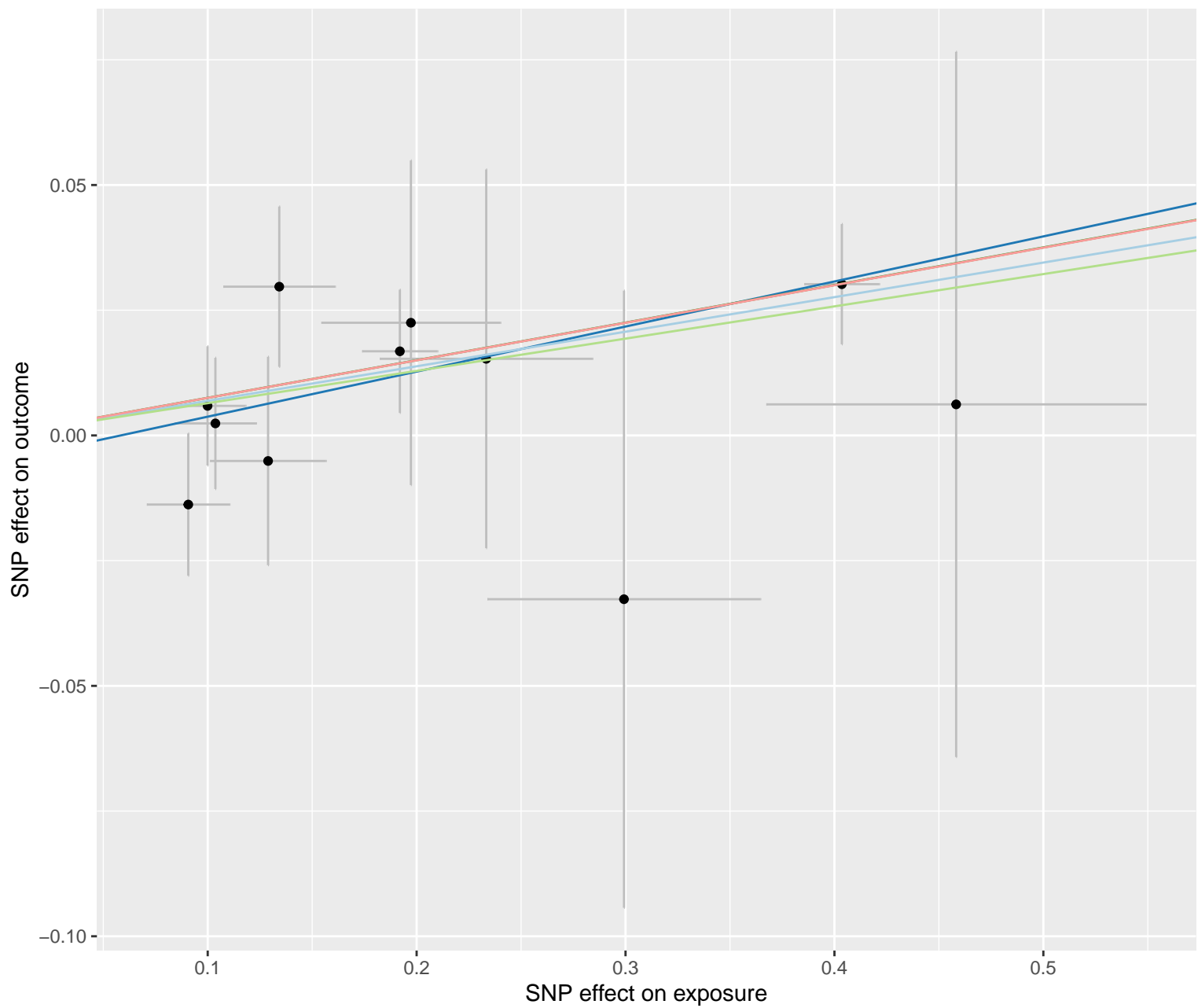

Supplement: Supplementary file 4 — Supplementary Material 4. [file 12944_2024_2103_MOESM4_ESM.zip › sFigure3∩╝êlipidomes-ER-BC∩╝ë/GCST90277330/scatter.pdf]

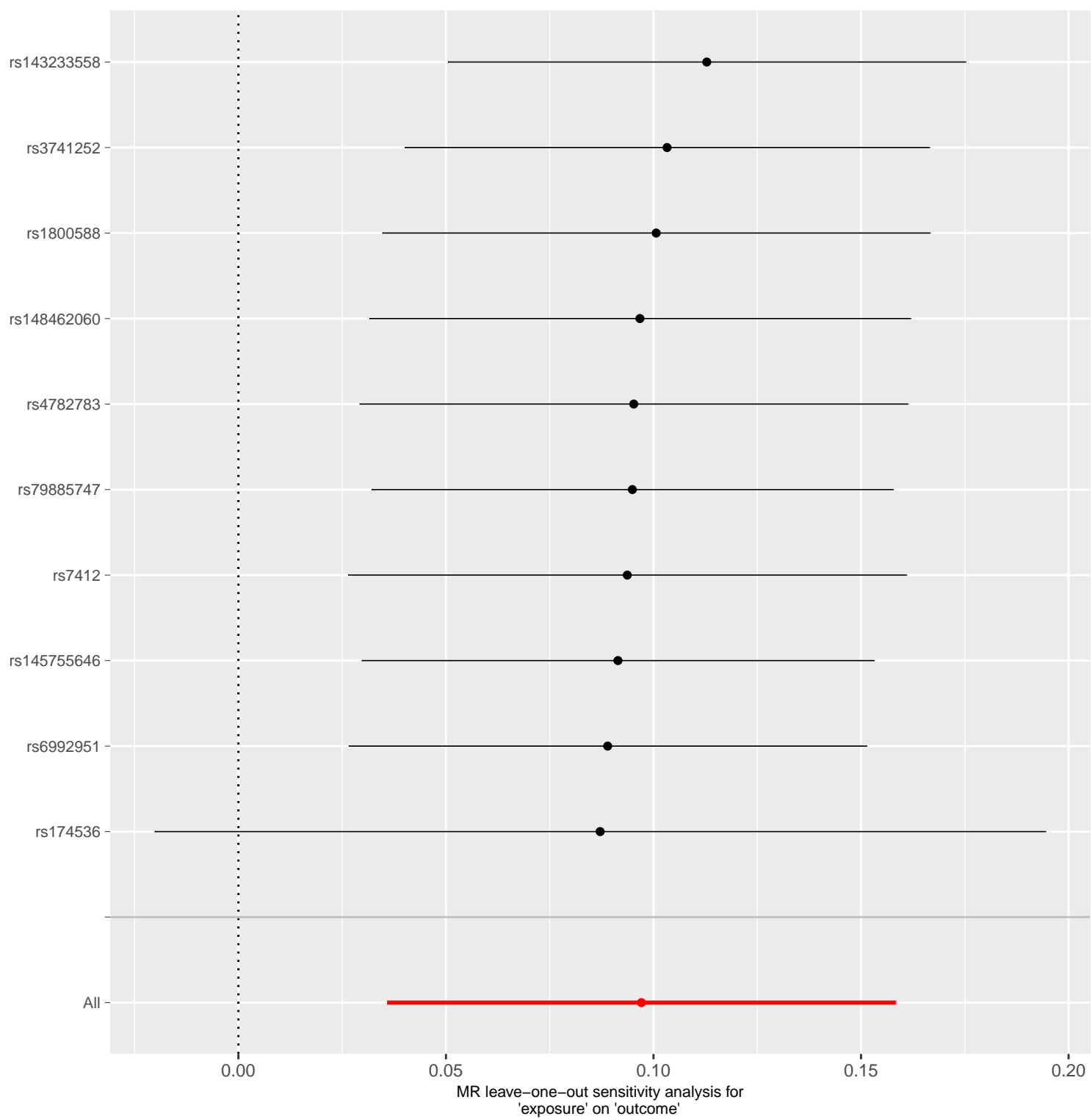

Supplement: Supplementary file 4 — Supplementary Material 4. [file 12944_2024_2103_MOESM4_ESM.zip › sFigure3∩╝êlipidomes-ER-BC∩╝ë/GCST90277336/sensitivity-analysis.pdf]

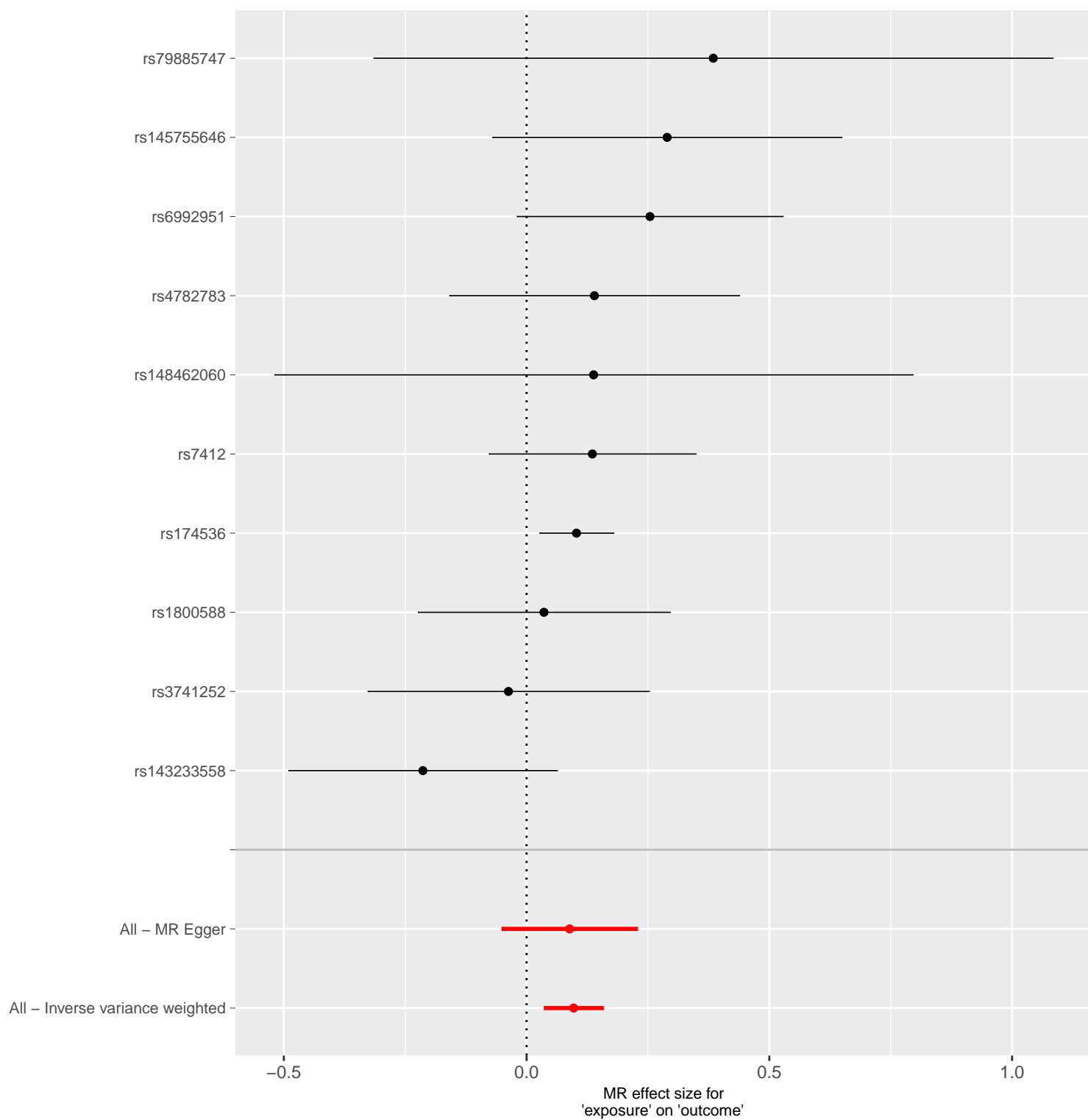

Supplement: Supplementary file 4 — Supplementary Material 4. [file 12944_2024_2103_MOESM4_ESM.zip › sFigure3∩╝êlipidomes-ER-BC∩╝ë/GCST90277336/forest.pdf]

# MR Method

- Inverse variance weighted
- MR Egger

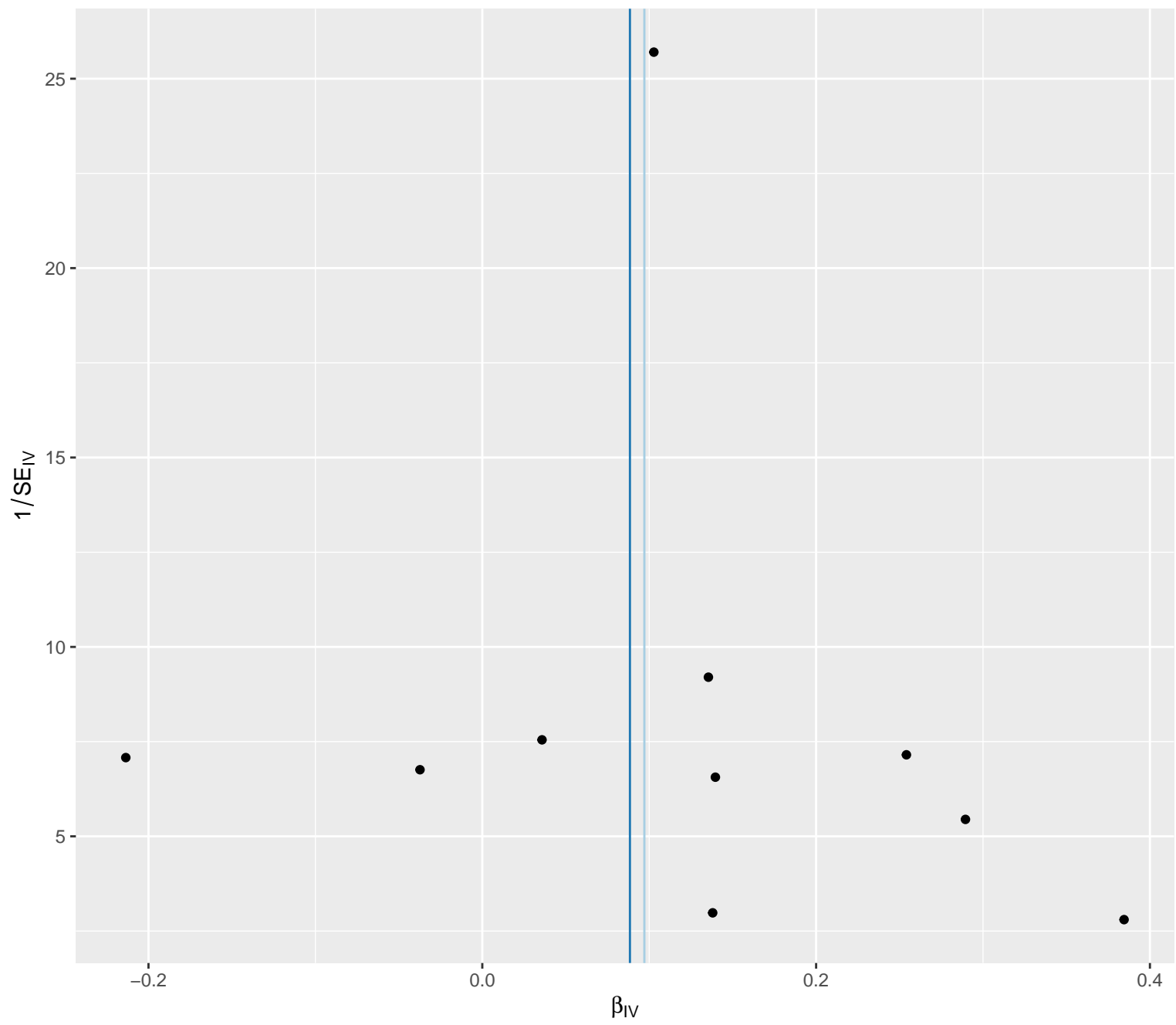

Supplement: Supplementary file 4 — Supplementary Material 4. [file 12944_2024_2103_MOESM4_ESM.zip › sFigure3∩╝êlipidomes-ER-BC∩╝ë/GCST90277336/funnelplot.pdf]

# MR Test

- Inverse variance weighted
- MR Egger
- Simple mode
- Weighted median
- Weighted mode

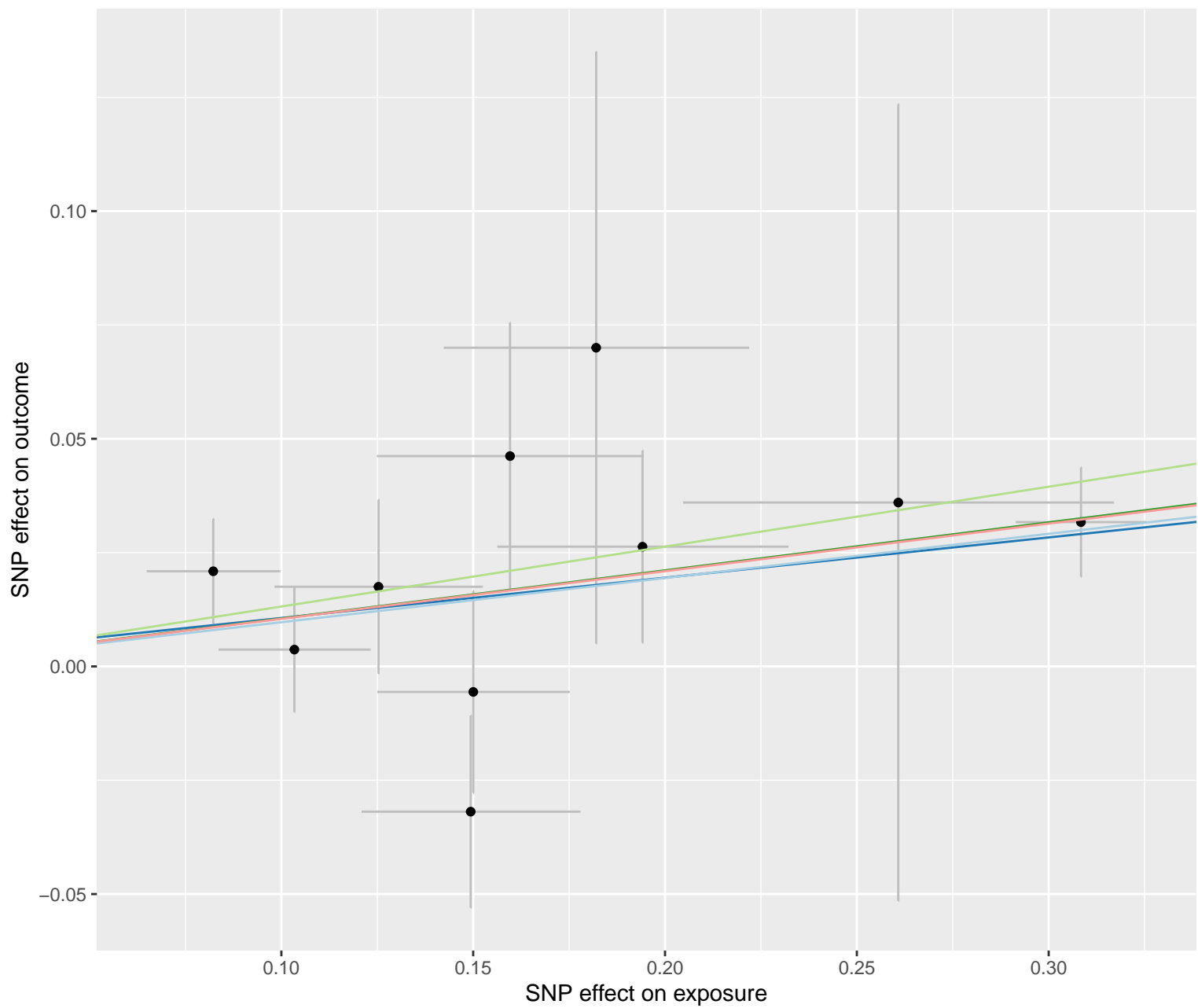

Supplement: Supplementary file 4 — Supplementary Material 4. [file 12944_2024_2103_MOESM4_ESM.zip › sFigure3∩╝êlipidomes-ER-BC∩╝ë/GCST90277336/scatter.pdf]

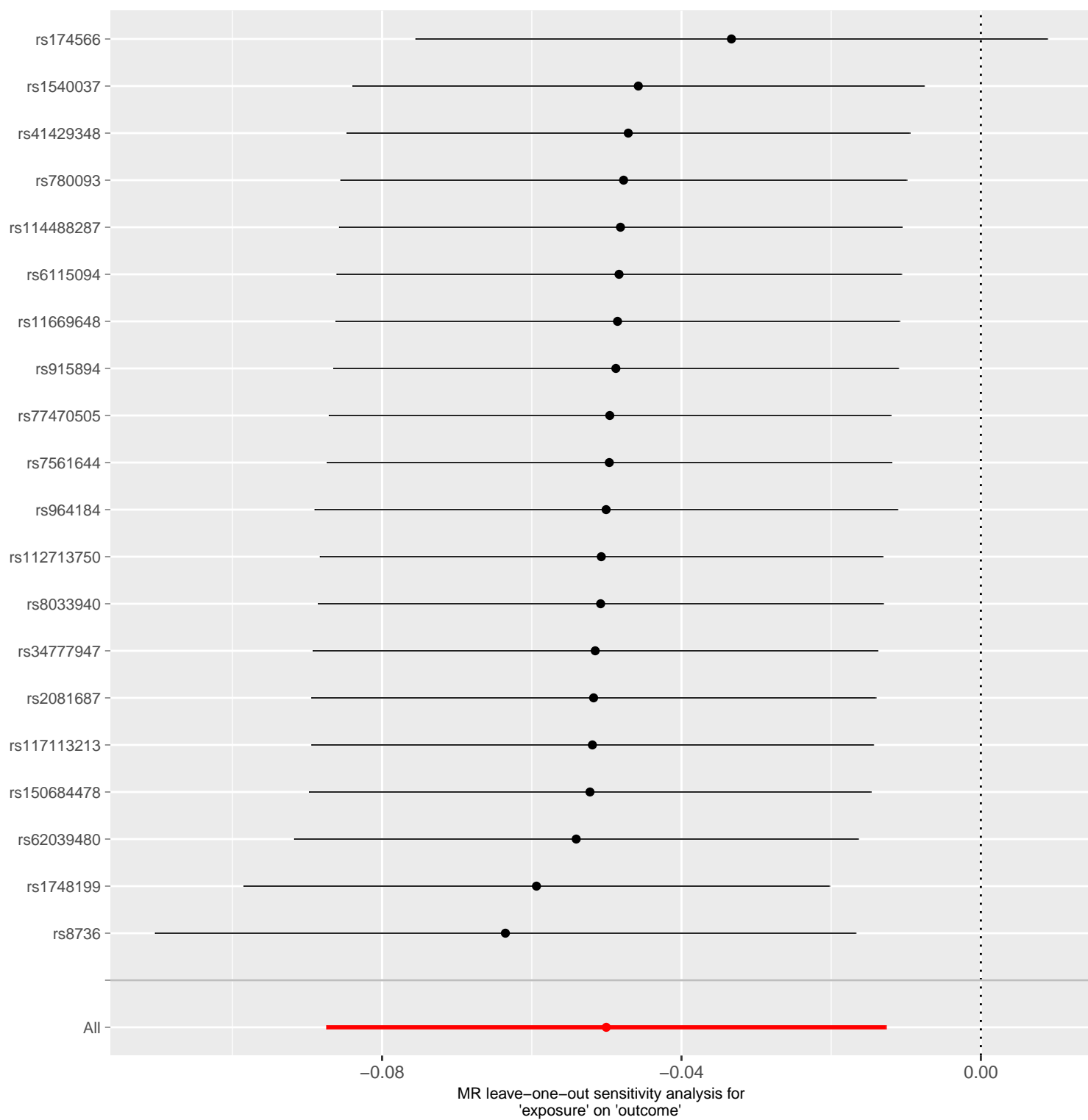

Supplement: Supplementary file 4 — Supplementary Material 4. [file 12944_2024_2103_MOESM4_ESM.zip › sFigure3∩╝êlipidomes-ER-BC∩╝ë/GCST90277362/sensitivity-analysis.pdf]

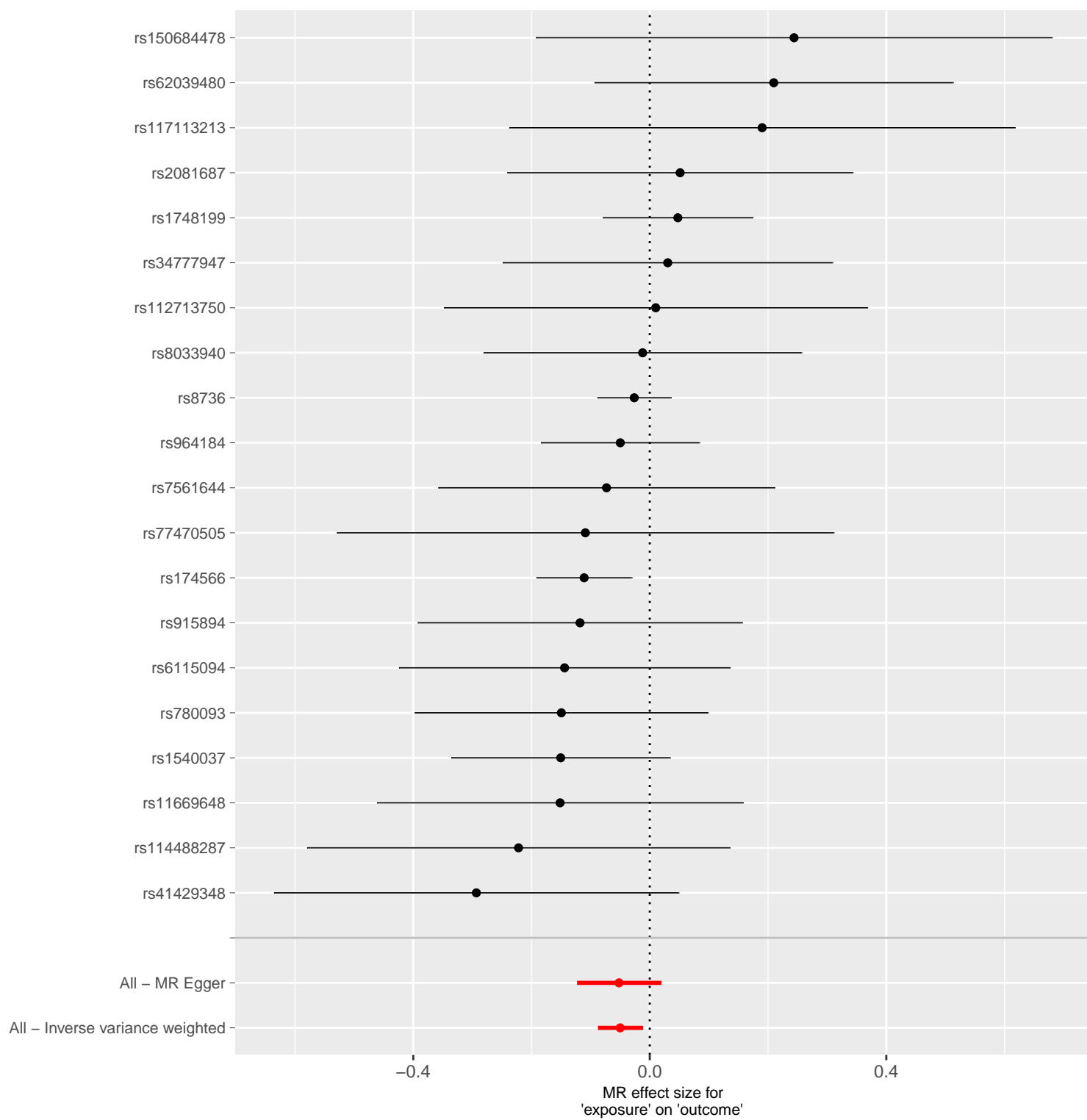

Supplement: Supplementary file 4 — Supplementary Material 4. [file 12944_2024_2103_MOESM4_ESM.zip › sFigure3∩╝êlipidomes-ER-BC∩╝ë/GCST90277362/forest.pdf]

# MR Method

- Inverse variance weighted
- MR Egger

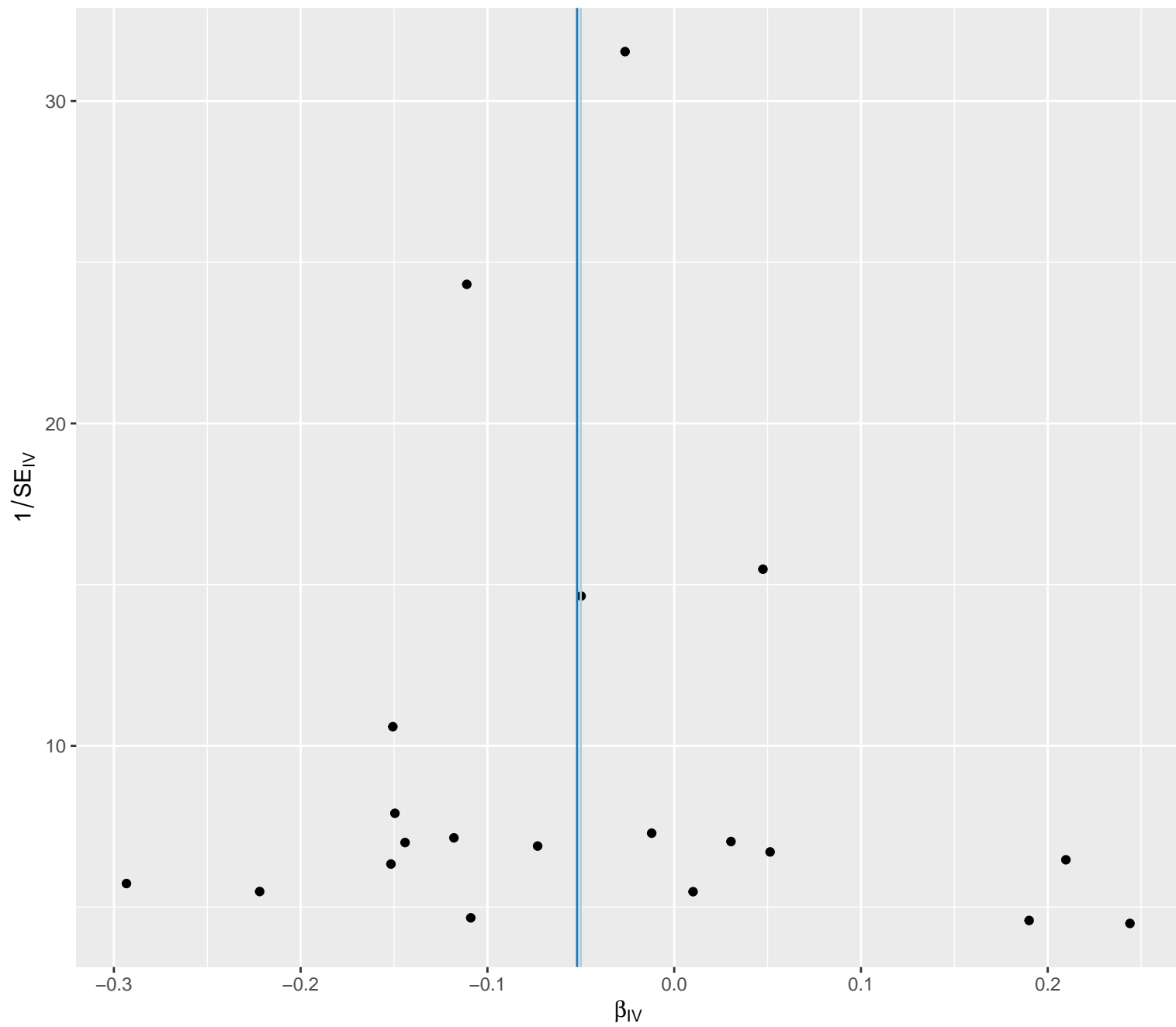

Supplement: Supplementary file 4 — Supplementary Material 4. [file 12944_2024_2103_MOESM4_ESM.zip › sFigure3∩╝êlipidomes-ER-BC∩╝ë/GCST90277362/funnelplot.pdf]

# MR Test

- Inverse variance weighted
- MR Egger
- Simple mode
- Weighted median
- Weighted mode

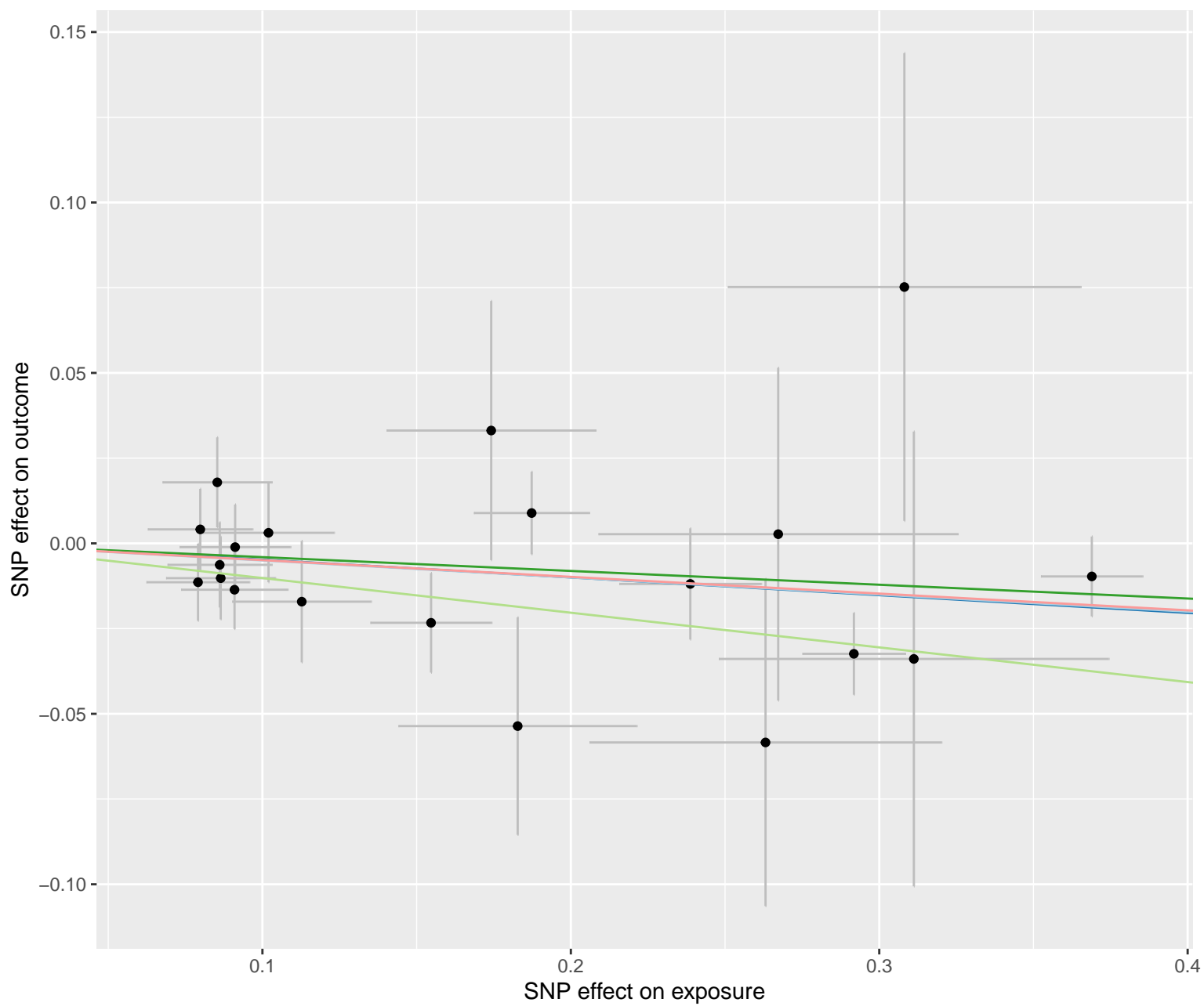

Supplement: Supplementary file 4 — Supplementary Material 4. [file 12944_2024_2103_MOESM4_ESM.zip › sFigure3∩╝êlipidomes-ER-BC∩╝ë/GCST90277362/scatter.pdf]

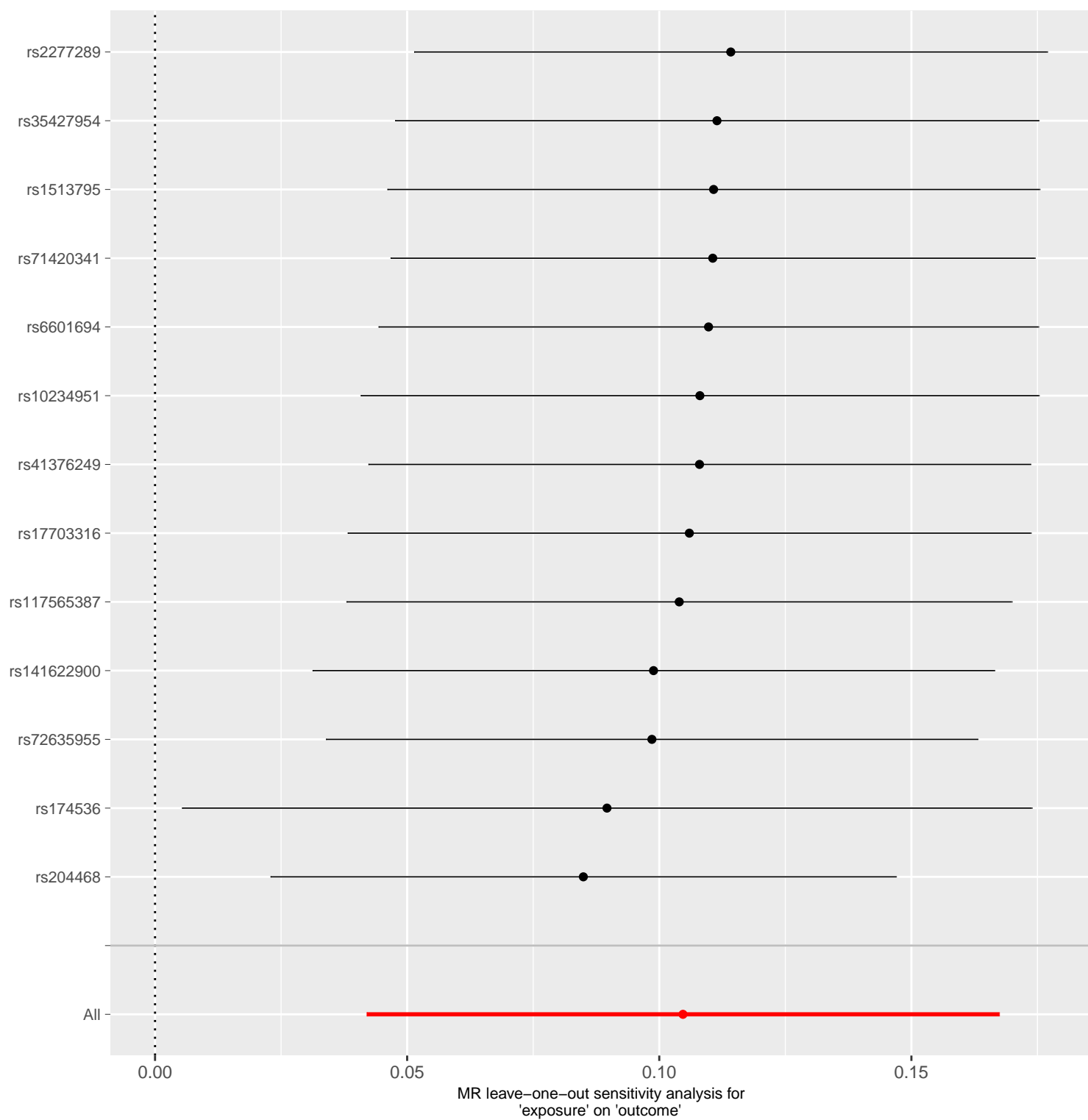

Supplement: Supplementary file 4 — Supplementary Material 4. [file 12944_2024_2103_MOESM4_ESM.zip › sFigure3∩╝êlipidomes-ER-BC∩╝ë/GCST90277354/sensitivity-analysis.pdf]

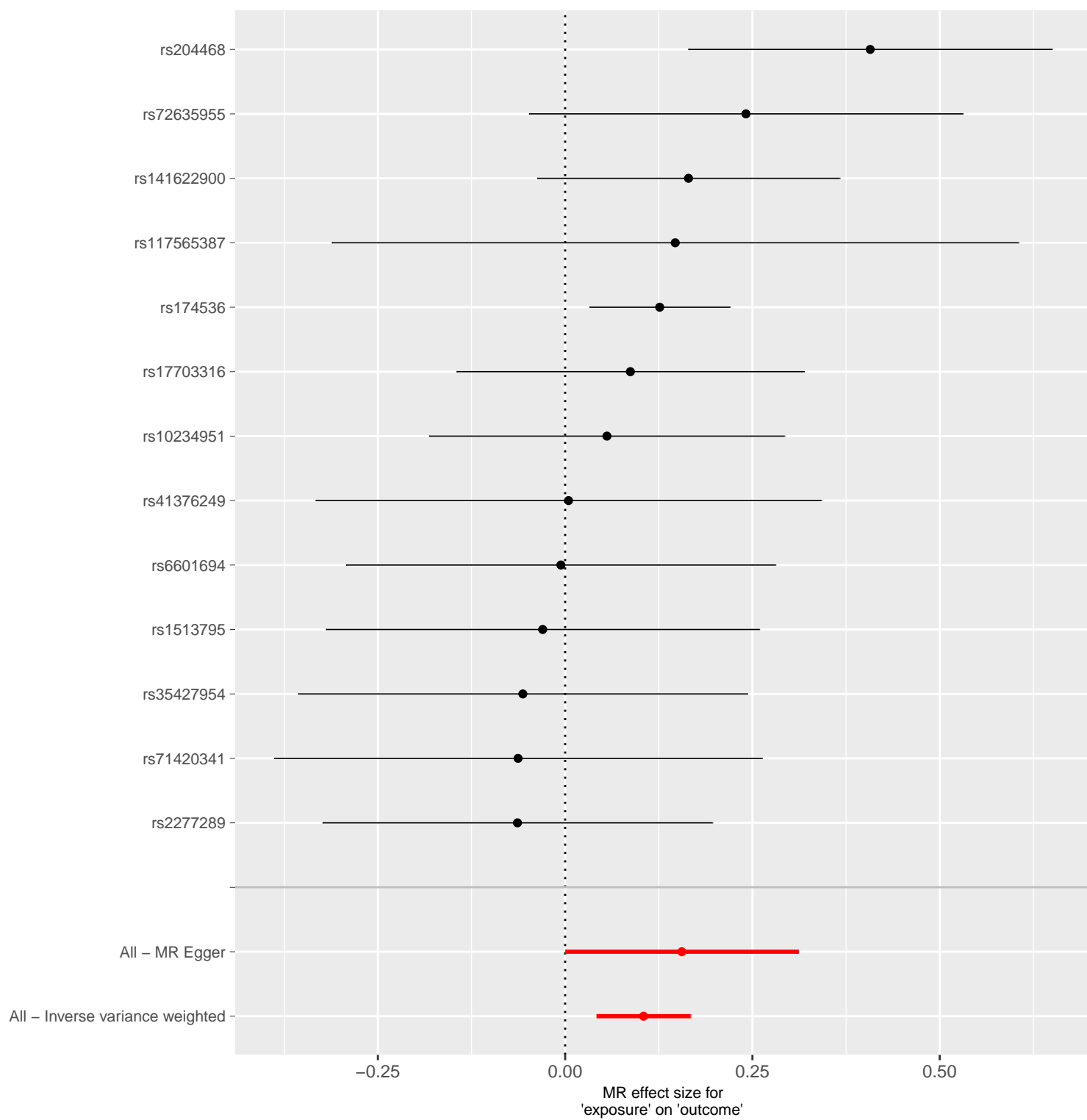

Supplement: Supplementary file 4 — Supplementary Material 4. [file 12944_2024_2103_MOESM4_ESM.zip › sFigure3∩╝êlipidomes-ER-BC∩╝ë/GCST90277354/forest.pdf]

# MR Method

- Inverse variance weighted
- MR Egger

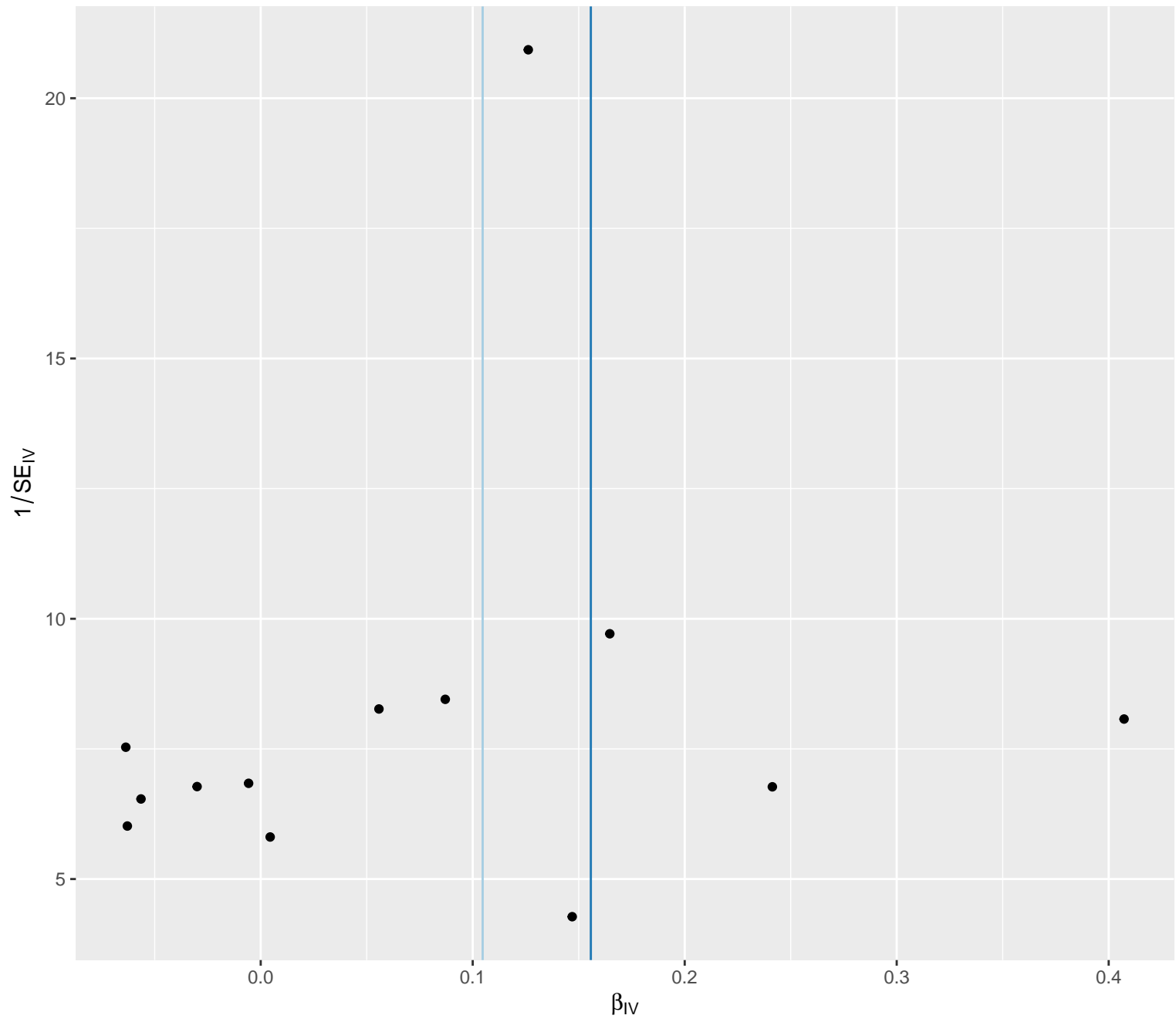

Supplement: Supplementary file 4 — Supplementary Material 4. [file 12944_2024_2103_MOESM4_ESM.zip › sFigure3∩╝êlipidomes-ER-BC∩╝ë/GCST90277354/funnelplot.pdf]

# MR Test

- Inverse variance weighted
- MR Egger
- Simple mode
- Weighted median
- Weighted mode

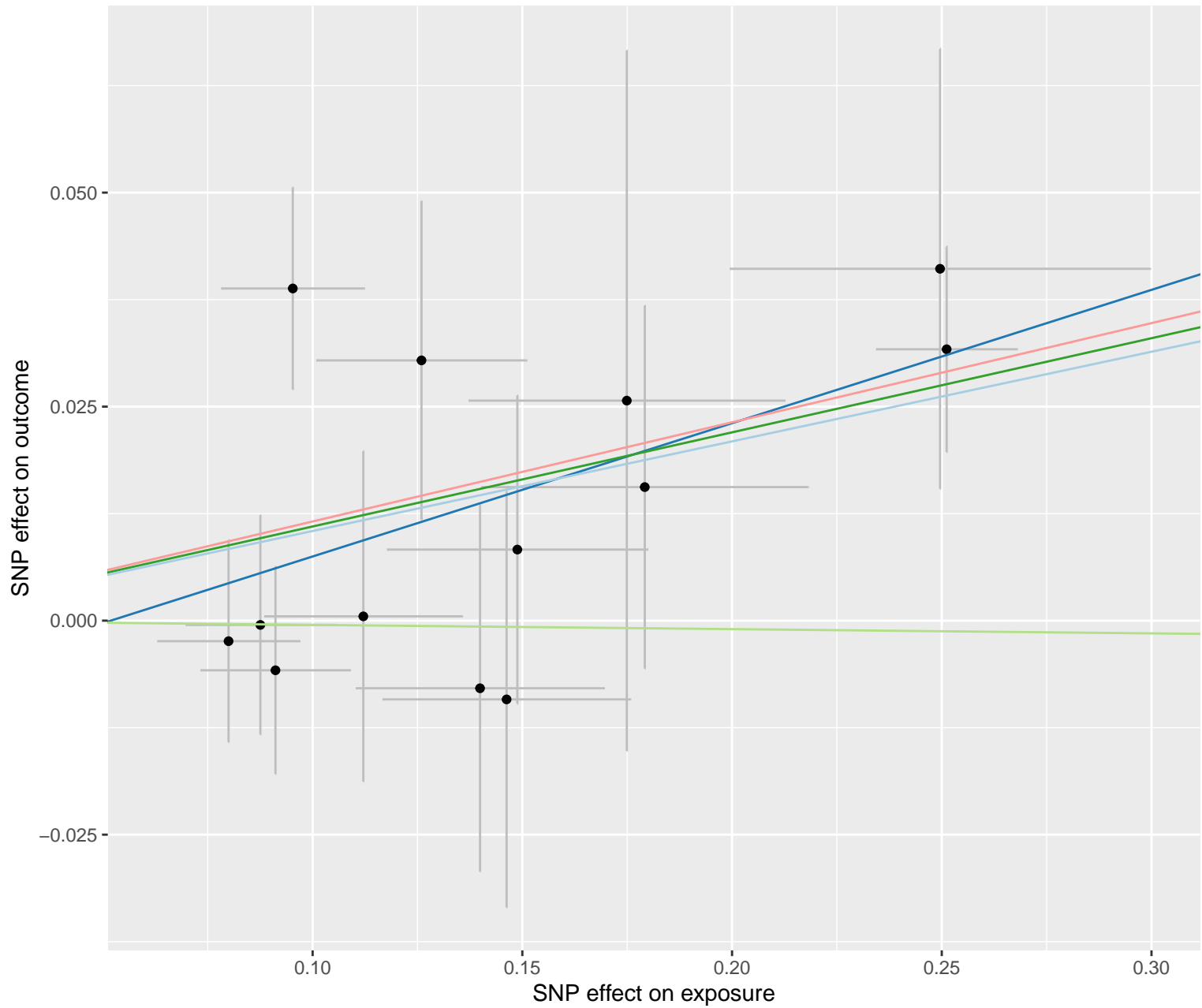

Supplement: Supplementary file 4 — Supplementary Material 4. [file 12944_2024_2103_MOESM4_ESM.zip › sFigure3∩╝êlipidomes-ER-BC∩╝ë/GCST90277354/scatter.pdf]

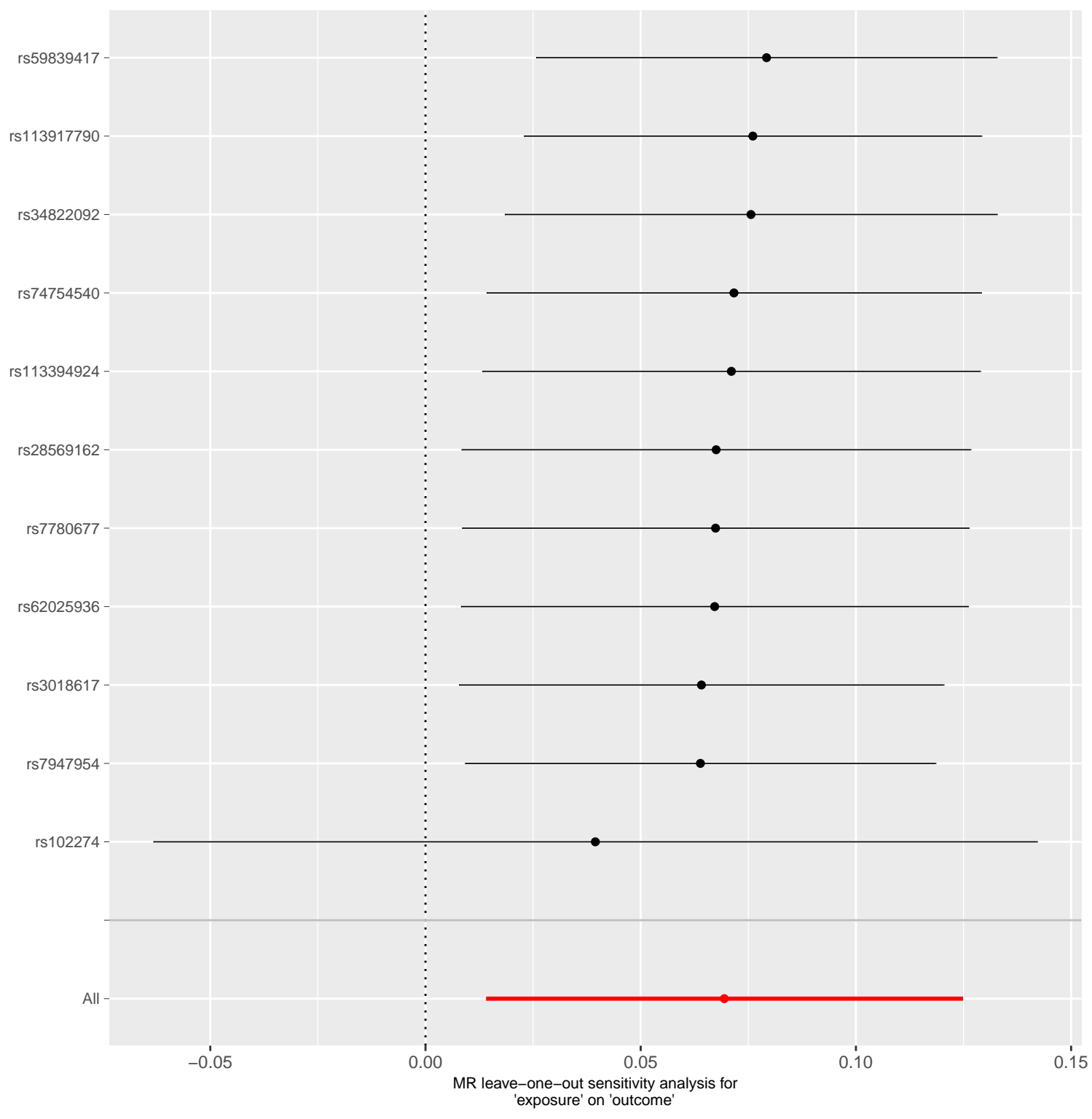

Supplement: Supplementary file 4 — Supplementary Material 4. [file 12944_2024_2103_MOESM4_ESM.zip › sFigure3∩╝êlipidomes-ER-BC∩╝ë/GCST90277288/sensitivity-analysis.pdf]

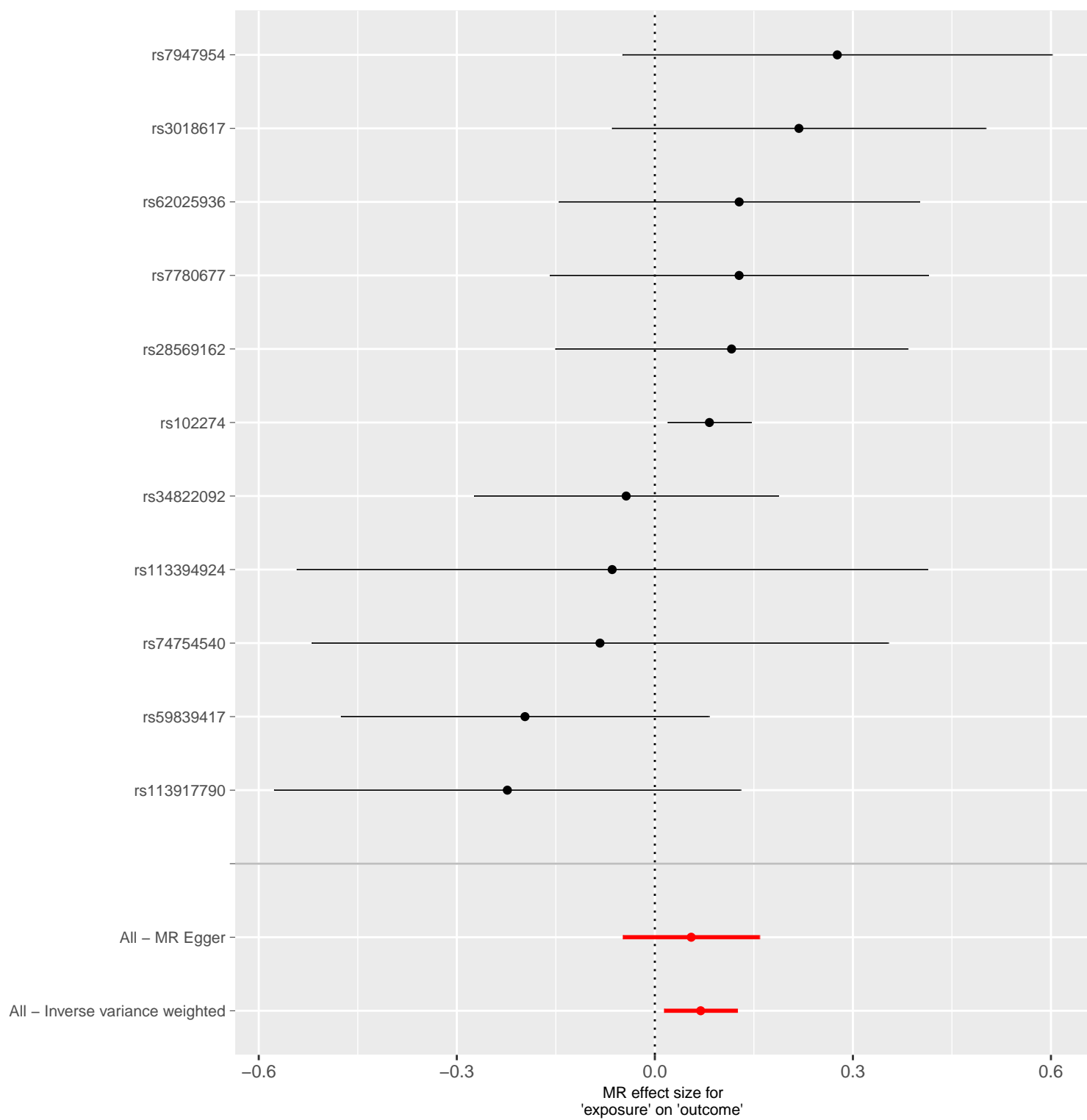

Supplement: Supplementary file 4 — Supplementary Material 4. [file 12944_2024_2103_MOESM4_ESM.zip › sFigure3∩╝êlipidomes-ER-BC∩╝ë/GCST90277288/forest.pdf]

# MR Method

- Inverse variance weighted
- MR Egger

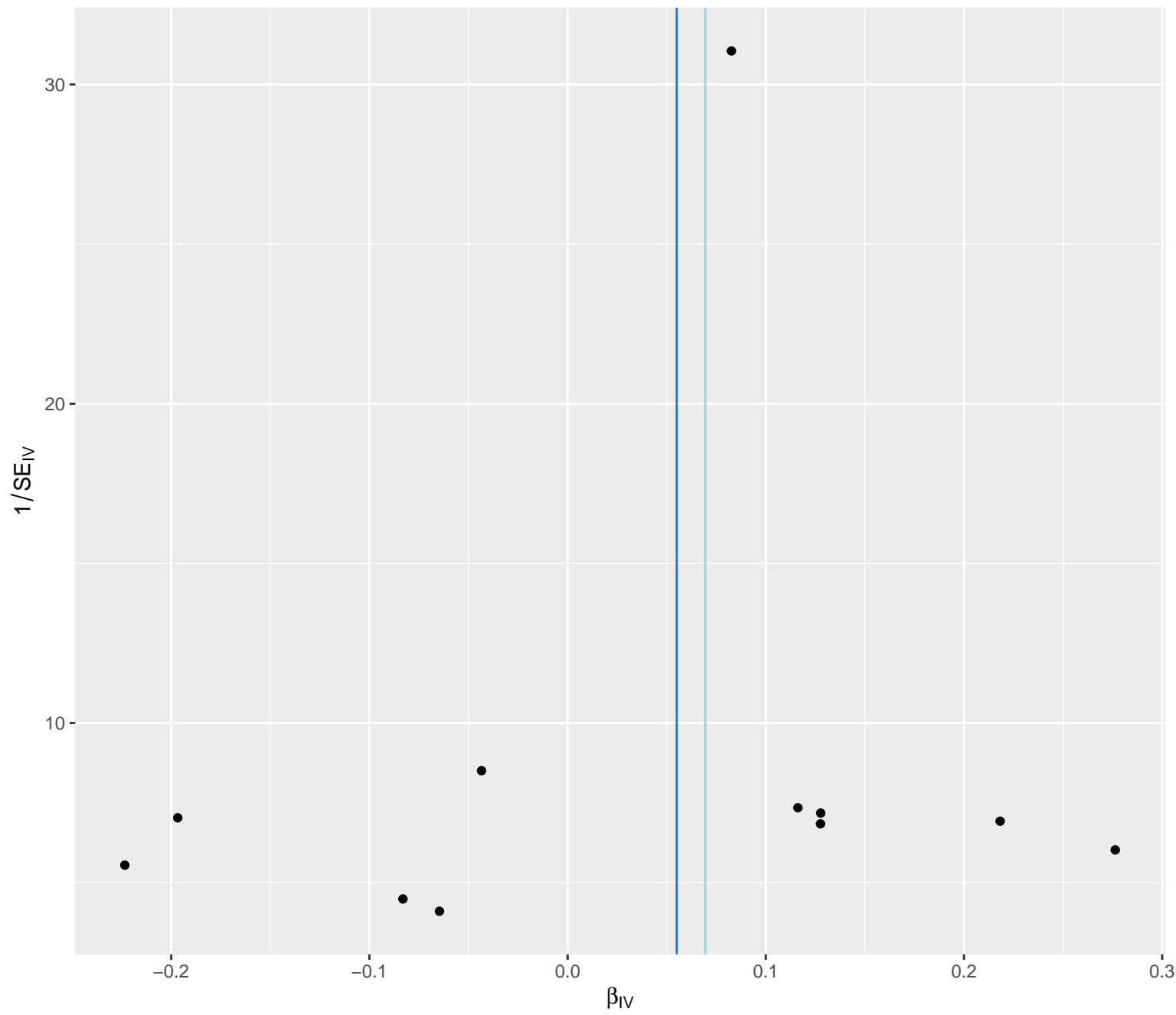

Supplement: Supplementary file 4 — Supplementary Material 4. [file 12944_2024_2103_MOESM4_ESM.zip › sFigure3∩╝êlipidomes-ER-BC∩╝ë/GCST90277288/funnelplot.pdf]

# MR Test

- Inverse variance weighted
- MR Egger
- Simple mode
- Weighted median
- Weighted mode

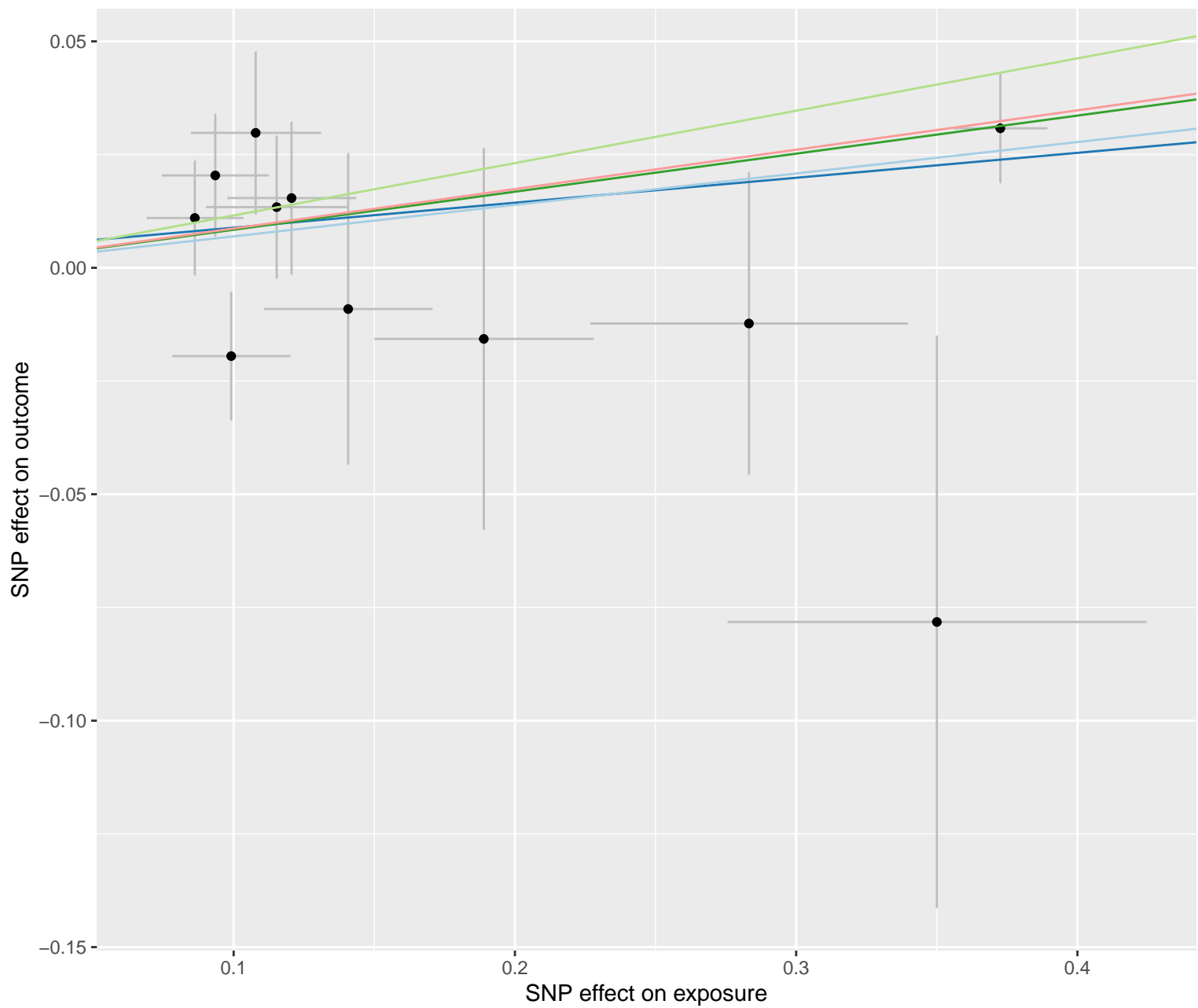

Supplement: Supplementary file 4 — Supplementary Material 4. [file 12944_2024_2103_MOESM4_ESM.zip › sFigure3∩╝êlipidomes-ER-BC∩╝ë/GCST90277288/scatter.pdf]
