# Supplementary material for: The impact of lipidome on breast cancer: a Mendelian randomization study
Source: Lipids Health Dis. 2024 Apr 15;23:109. doi: 10.1186/s12944-024-02103-2 (PMC11017498; doi:10.1186/s12944-024-02103-2)
Supplement: Supplementary file 5 — Supplementary Material 5. [file 12944_2024_2103_MOESM5_ESM.rtf]

install.packages("devtools")devtools::install_github("MRCIEU/TwoSampleMR")install.packages("ggplot2")rm(list = ls())#librarylibrary(TwoSampleMR)library(ggplot2)library(foreach)setwd("/Users/yuchen/Downloads/lipidomes/clump”)lipidome<-data.table::fread("xxxx")lipidome<-subset(lipidome,select=c("rsid","effect_allele","other_allele","effect_allele_frequency","beta",                                     "standard_error","neg_log_10_p_value","n"))colnames(lipidome)<-c("rsid","A2","A1","FRQ","BETA",                        "SE","P","N")lipidome <- subset(lipidome, P<5E-6)lipidome$"F" <- (lipidome$BETA/lipidome$SE)^2lipidome <- subset(lipidome, F>10)lipidome <- subset(lipidome, select = -F)lipidome$MAF <- ifelse(lipidome$FRQ<0.5,lipidome$FRQ,1-lipidome$FRQ)lipidome <- subset(lipidome,MAF>0.01)lipidome <- subset(lipidome,select = -MAF)exp_data <- TwoSampleMR::format_data( lipidome, type = "exposure", snp_col = "rsid", chr_col = "CHR", pos_col = "BP", phenotype_col = "phenotype", effect_allele_col = "A2", other_allele_col = "A1", eaf_col = "FRQ", beta_col = "BETA", se_col = "SE", pval_col = "P",samplesize_col = "N")exp_data <- TwoSampleMR::clump_data(exp_data,clump_kb=10000,clump_r2=0.001,pop="EUR")##change the 179 lipidome id one by oneexpo_rt<-read_exposure_data(filename = “id.csv”, sep = ", “, snp_col = "SNP", beta_col = "beta.exposure",    se_col = "se.exposure", effect_allele_col = "effect_allele.exposure", other_allele_col = "other_allele.exposure", eaf_col = "eaf.exposure", pval_col = "pval.exposure", samplesize_col = "samplesize.exposure")outc_rt = extract_outcome_data(snps=exposure_dat$SNP, outcomes="ieu-a-1126") ##change the outcome id into ieu-a-1126/ ieu-a-1127/ieu-a-1128TSMR <- harmonise_data(    exposure_dat =  expo_rt,     outcome_dat = outc_rt,action=2)  TSMR$R2 <- (2 * (TSMR$beta.exposure^2) * TSMR$eaf.exposure * (1 - TSMR$eaf.exposure)) /    (2 * (TSMR$beta.exposure^2) * TSMR$eaf.exposure * (1 - TSMR$eaf.exposure) +       2 * TSMR$samplesize.exposure*TSMR$eaf.exposure * (1 - TSMR$eaf.exposure) * TSMR$se.exposure^2)  TSMR$f <- TSMR$R2 * (TSMR$samplesize.exposure - 2) / (1 - TSMR$R2)  TSMR$meanf<- mean( TSMR$f)  TSMR<-TSMR[TSMR$f>10,]  mr_result<- mr(TSMR)result_or=generate_odds_ratios(mr_result) #if P<0.05, continue to the next stepswrite.table(TSMR, file ="harmonise.txt",row.names = F,sep = "\t",quote = F)write.table(result_or[,5:ncol(result_or)],file =“OR.txt",row.names = F,sep = "\t",quote = F)pleiotropy=mr_pleiotropy_test(TSMR)write.table(pleiotropy,file = "pleiotropy.txt",sep = "\t",quote = F)heterogeneity=mr_heterogeneity(TSMR)write.table(heterogeneity,file = "heterogeneity.txt",sep = "\t",quote = F)p1 <- mr_scatter_plot(mr_result, TSMR)ggsave(p1[[1]], file="scatter.pdf", width=8, height=8)singlesnp_res<- mr_singlesnp(TSMR)singlesnpOR=generate_odds_ratios(singlesnp_res)write.table(singlesnpOR,file="singlesnpOR.txt"),row.names = F,sep = "\t",quote = F)p2 <- mr_forest_plot(singlesnp_res)ggsave(p2[[1]], file="forest.pdf"), width=8, height=8)sen_res<- mr_leaveoneout(TSMR)p3 <- mr_leaveoneout_plot(sen_res)ggsave(p3[[1]], file="sensitivity-analysis.pdf"), width=8, height=8)res_single <- mr_singlesnp(TSMR)p4 <- mr_funnel_plot(singlesnp_res)ggsave(p4[[1]], file="funnelplot.pdf"), width=8, height=8)presso=run_mr_presso(TSMR,NbDistribution = 1000)capture.output(presso,file = "presso.txt")library(TwoSampleMR)library(ggplot2)library(foreach)source("BWMR_updated.R") #download from: https://github.com/jiazhao97/BWMRBW <- BWMR(gammahat = TSMR$beta.exposure,                 Gammahat = TSMR$beta.outcome,                 sigmaX = TSMR$se.exposure,                 sigmaY = TSMR$se.outcome) beta=BW[["beta"]]  lci95 <- BW[["beta"]]-1.96*BW[["se_beta"]]  uci95 <- BW[["beta"]]+1.96*BW[["se_beta"]]  or <- exp(BW[["beta"]])  or_lci95 <- exp(lci95)  or_uci95 <- exp(uci95)  pval<-BW[["P_value"]]  result=rbind(result,cbind(id=i,method="BWMR",beta,lci95,uci95,or,or_lci95,or_uci95,pval))
